# Gene-encoding DNA origami for mammalian cell expression

**DOI:** 10.1038/s41467-023-36601-1

**Published:** 2023-02-23

**Authors:** Jessica A. Kretzmann, Anna Liedl, Alba Monferrer, Volodymyr Mykhailiuk, Samuel Beerkens, Hendrik Dietz

**Affiliations:** 1 Department of Biosciences, School of Natural Sciences, Technical University of Munich, Am Coulombwall 4a, 85748 Garching, Germany; 2 Munich Institute of Biomedical Engineering, Technical University of Munich, Boltzmannstraße 11, 85748 Garching, Germany

**Keywords:** Biotechnology, DNA nanotechnology

## Abstract

DNA origami may enable more versatile gene delivery applications through its ability to create custom nanoscale objects with specific targeting, cell-invading, and intracellular effector functionalities. Toward this goal here we describe the expression of genes folded in DNA origami objects delivered to mammalian cells. Genes readily express from custom-sequence single-strand scaffolds folded within DNA origami objects, provided that the objects can denature in the cell. We demonstrate enhanced gene expression efficiency by including and tuning multiple functional sequences and structures, including virus-inspired inverted-terminal repeat-like (ITR) hairpin motifs upstream or flanking the expression cassette. We describe gene-encoding DNA origami bricks that assemble into multimeric objects to enable stoichiometrically controlled co-delivery and expression of multiple genes in the same cells. Our work provides a framework for exploiting DNA origami for gene delivery applications.

## Abstract (web summary)

DNA origami may enable more versatile gene delivery applications through its ability to create custom nanoscale objects. Here the authors show that genes folded in DNA origami with custom scaffolds express efficiently when delivered to mammalian cells and can be assembled into multimeric arrays to deliver and express defined ratios of multiple genes simultaneously.

## Introduction

The delivery and expression of custom genes in cells drive major scientific discoveries and underpin a growing number of medical and technical applications^1^. The successful delivery of genetic material to specific cells or tissues continues to be a significant challenge. Packaging, delivering, and expressing the desired nucleic acids often must be addressed in an application-specific manner^2^. Additional challenges arise for the simultaneous delivery and expression of multiple custom genes to cells, which could be desirable for yet more advanced genome or epigenome editing applications, for transcriptional modulation, and/or the programming of new genetic circuits^3–7^.

The methods of DNA nanotechnology enable the rational design of custom-shaped objects that self-assemble in solution from sets of DNA molecules^8^. DNA origami is a popular design approach^9–11^, in which a long template DNA single strand termed “scaffold” is folded into custom shapes by sets of DNA oligonucleotides with designed sequences (termed “staples”). DNA origami objects can comprise hundreds of unique DNA strands and thousands of DNA base pairs, and can form with high-yield and high-quality^12–15^. DNA origami methodologies also allow making well-defined higher-order macromolecular objects with dimensions up to the size of viruses^14,16,17^. DNA origami objects can be site-selectively functionalized with proteins such as antibodies, aptamers, and organic small molecules but also with inorganic particles^18,19^. DNA nanostructures can also deform lipid membranes^20,21^, they can form channels in lipid membranes^22^, and they can be enveloped within lipid membranes^23^. SNARE-protein-based fusion of solid-supported lipid membranes with lipid vesicles has been induced in vitro with a DNA origami platform^24^. DNA origami methods also allow the rational design of a great variety of stimuli-dependent reconfigurable assemblies^16,25^, that is, objects where the type of conformational change such as opening/closing of a cavity, piston-like actuation, rotation, and also the type of stimulus (hydrophobicity^26^, ionic strength and temperature changes^16^, pH changes^27^, RNA detection^28^, antigen detection^29^) can be defined by the user.

The capability for designing shapes and controlling their conformations and functionalization, and the option for positioning non-DNA components with nanometer-scale precision has popularized DNA origami and led to the pursuit of applications in a variety of fields^25^ including drug delivery^30–32^, immunotherapy^33–35^, sensing and imaging^36–39^. Commonly, the sequences of the DNA molecules in DNA origami are designed for purely structural purposes, that is, to enable folding of the target object and for positioning guest molecules via site-specific DNA hybridization reactions. DNA origami objects are also often designed around generic single-stranded “scaffolds” derived from bacteriophage genomes that are not suited for gene expression in mammalian cells. With the development of techniques for building fully sequence programmable synthetic scaffolds for DNA origami^40^, however, these limitations can be overcome and designing synthetic mammalian-cell expressible gene cassettes and folding them into custom DNA origami objects has become more accessible.

Gene therapeutic applications have been approached thus far with DNA origami hybrid objects featuring additional RNA or proteins, which bypasses the need for expression from nucleic acids^41–44^. Lin-Shiao et al.^44^ recently demonstrated delivery and integration of template DNA for gene editing structured within a DNA origami, bound together with Cas9 ribonuclear proteins (RNPs). Here we investigate the determinants for designing the sequences of synthetic DNA origami scaffolds and the structures of DNA origami objects, so that genes encoded within DNA molecules in these objects become expressible by mammalian cells. We establish a functional end-point—the expression of a target gene —to help exploit DNA origami for future gene delivery and potential gene therapeutic applications.

We find that a prerequisite for expression is that DNA origami objects unfold within the intracellular environment. Genes may then express from the single-stranded DNA (ssDNA) scaffold strand laid bare. We further found that the efficiency of gene expression can be enhanced with targeted design of helper staple strands and by the inclusion of several features, such as a Kozak sequence^45^, woodchuck post-transcriptional regulatory element^46^, and at least one inverted terminal repeat (ITR)^47^, preferably upstream of the expression cassette in the synthetic scaffold. Exemplarily, we demonstrate multiplexed gene expression from higher-order DNA origami assemblies, where each of the DNA origami subunits encoded for a specific gene of interest. These “click-and-express” objects enable co-delivery and expression of an array of genes with improved stoichiometric control relative to traditional methods such as the Poisson-distribution-limited co-transfection with plasmids.

## Results and discussion

### Genes are expressed from DNA origami independent of gene position or origami shape

**Fig. 1:**
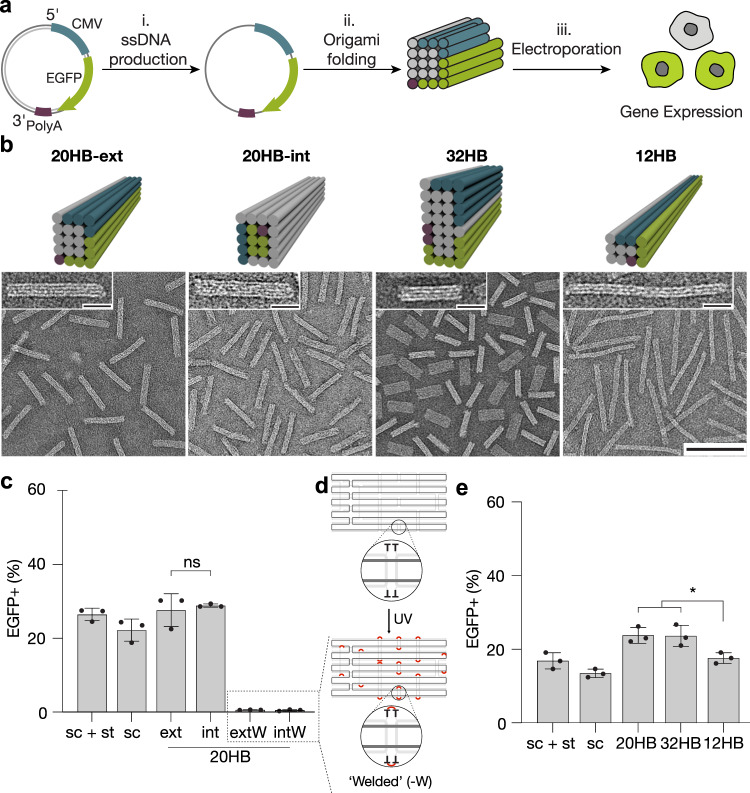
Folding and expressing genes from origami structures. **a** Schematics of the overall workflow: ssDNA is produced from plasmid DNA via phagemids (i), and then folded into 20 helix bundle (20HB) DNA origami objects (ii). Objects were delivered to cells, and gene expression from the origami structure was assessed by detection of positive fluorescence read-out (iii). CMV promoter sequence is shown in blue, gene encoding for enhanced green fluorescent protein (EGFP) in green, and the bGH polyadenylation signal (polyA) in purple. **b** Cylinder models and negative-staining transmission electron micrographs of the 20HB, 12HB and 32 HB are given in the upper and lower panels, respectively (scale bar 100 nm, insets 20 nm). Coloring demonstrates the positions of the scaffold features, for example 20HB-ext demonstrates the CMV (blue), EGFP (green) and polyA (purple) encoding sequences present along exterior helices, while 20HB-int presents the sequences encoding for EGFP and polyA within interior helices. **c** Transfection efficiency in HEK293T cells. **d** Schematics explaining internal crosslinking via UV irradiation. For UV-welded structures 20HB-ext-W and 20HB-int-W, EGFP expression was silenced. **e** Transfection efficiency in HEK293T cells by electroporation seen for 20HB(-ext), 32HB and 12HB structures. 12HB structure demonstrated lower transfection efficiency (EGFP + cells) than 20HB (*p* = 0.0287) and 32HB structures (*p* = 0.0325). Data collected in **c** and **e** were quantified using flow cytometry and are presented as mean ± standard deviation (s.d.) for *n* = 3 biologically independent experiments, source data provided. Individual data points are overlaid, controls are unfolded scaffold and staple mixture ‘sc + st’ and scaffold only ‘sc’, 0.5 µg total DNA per condition. Statistical analysis was performed using one-way ANOVA with Tukey’s multiple comparison (*p ≤ 0.05, ns *p* > 0.05). Corresponding EGFP-encoding plasmid was used as a positive control, given in Supplementary Fig. 6.

To determine the basic parameters of DNA origami designs that mammalian cells will express, we created a customized circular ssDNA scaffold termed sc_EGFP1 which encoded for an enhanced green fluorescent protein (EGFP) in the 5’ to 3’ direction as coding strand (Fig. 1a, Supplementary Fig. 1). Cells which successfully express EGFP from the scaffold can then be identified via fluorescence detection. We use two observables in this study: the fraction of cells showing fluorescence (termed transfection efficiency), and the mean fluorescence intensity (MFI) per cell which we use as a proxy for gene expression efficiency. We used electroporation to deliver the DNA origami objects directly to the cell to focus on parameters directly affecting the expression. Since electroporation in a traditional cuvette caused DNA origami aggregation, consistent with previous studies^48^, we used electroporation via the Neon^TM^ transfection system (Supplementary Fig. 2). We utilized human embryonic kidney 293T (HEK293T) cells as a model cell line due to their ease of transfection.

Our custom EGFP scaffold expressed in high yield and purity via phagemid production, and the scaffold folded efficiently into the designed target objects (Fig. 1b and Supplementary Fig. 3a (left)). To address whether the spatial position of the gene within the DNA origami object affects expression, we designed two 20-helix bundles (20HBs) variants where the EGFP gene was positioned either on the exterior (20HB-ext) or in the interior (20HB-int) of the multi-layer DNA origami (Fig. 1b, first two panels, Supplementary Fig. 3a (right)). Gene expression occurred from both 20HB variants in HEK293T cells after electroporation (Fig. 1c, Supplementary Fig. 4a). There was no statistically significant difference in either the transfection or expression efficiency between the two objects.

The aspect ratio of DNA origami has previously been reported to influence cellular uptake^49,50^. To elucidate whether aspect ratio influences expression, we designed a ~114 nm long twelve-helix bundle (12HB), a ~69 nm long 20HB-ext, and a ~42 nm long 32HB with the EGFP gene and recognition sequences presented in all cases on the exterior of the bundles (Fig. 1b, Supplementary Fig. 3b). These objects have aspect ratios of ~15, 5 and 2 for the 12HB, 20HB, and 32HB, respectively. The EGFP and associated genes are presented in the 12HB as long continuous regions with minimal scaffold crossovers, while the 32HB presents them within the shortest continuous regions. When delivered to HEK293T cells we found no statistically significant difference in transfection and expression efficiency from the 20HB and the 32HB samples (Fig. 1e, Supplementary Fig. 4). A small decrease in transfection efficiency was seen from the 12HB sample (p ≤ 0.05) relative to 20HB and 32HB. However, the cell density after electroporation was also lower for the 12HB object (Supplementary Fig. 5).

Hence, for both transfection and expression efficiency it did not matter how the gene of interest was packaged among our panel of test DNA origami. This observation suggested that unfolding of the DNA origami is a requirement for gene expression. We tested this hypothesis with EGFP encoding objects that cannot unfold. To this end, we included extra thymidine residues in the staple strands for 20HB-ext and 20HB-int to enable internal crosslinking via UV point welding^51^. The objects were then internally stabilized by several hundred UV-induced cyclobutane pyrimidine dimer bonds between staple strands and at crossovers, which topologically prevents strand dissociation (Fig. 1d). When we delivered the UV point welded 20HB variants to the HEK293T cells, we found almost complete suppression of the EGFP signal (Fig. 1c). While exposure to UV radiation can also have a inhibitory effect on gene expression from plasmids^52,53^, the pronounced, near-complete inhibition of gene expression from the covalently crosslinked DNA origami supports our hypothesis that DNA origami must unfold prior for gene expression.

### Targeted design in promoter region enhances gene expression

**Fig. 2:**
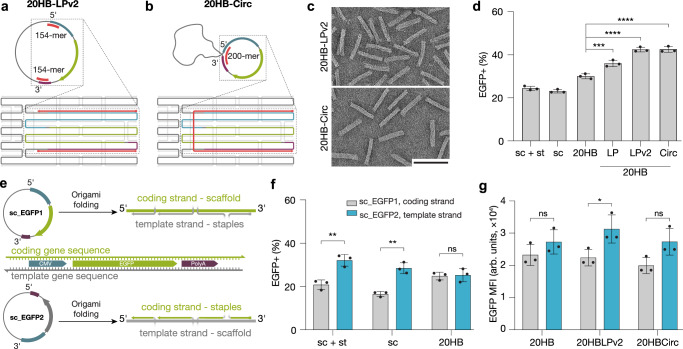
Optimization of gene expression through staple design and scaffold orientation. Scaffold routing and schematics of unfolded scaffold for 20HB-LPv2 and 20HB-Circ designs, **a** and **b** respectively. 20HB-LPv2 incorporates two continuous 154-mer staples (pink), and 20HB-Circ design has been routed so that the 200-mer staple (pink), which acts as a splint, brings together the 5’ start of the CMV, and the 3’ of the polyA. **c** TEM micrographs of 20HB-LPv2 and 20HB-Circ, scale bar 100 nm. **d** Transfection efficiency into HEK293T cells via electroporation revealed higher transfection efficiency for samples 20HB-LP (*p* = 0.00004), 20HB-LPv2 (*p* = 1.87 × 10^−7^) and 20HB-Circ (*p* = 1.82 × 10^−7^), when compared to the standard 20HB. **e** Scaffold used encoded for the ‘coding strand’, where the expression cassette is present in the 5’ to 3’ direction (sc_EGFP1, upper panel). Scaffold encoding for the reverse complementary sequence of the expression cassette, thus the ‘template strand’ (sc_EGFP2, lower panel) was designed and produced. **f** Transfection efficiency into HEK293T cells by electroporation with either sc_EGFP2 scaffold + staple mixture, scaffold only, or folded 20HB objects. Electroporation with the template strand, sc_EGFP2, demonstrated higher transfection efficiencies for sc + st (*p* = 0.0051) and sc (*p* = 0.0018) conditions, compared to sc_EGFP1. **g** EGFP mean fluorescence intensity (MFI) for 20HB, 20HB-LPv2 and 20HB-Circ structures folded with either the coding or the template strand as the scaffold, transfected into HEK293T cells. Structure 20HB-LPv2 demonstrated higher MFI when folded with the template strand (*p* = 0.0388). Data collected in **d**, **f** and **g** were quantified using flow cytometry and are presented as mean ± s.d. for *n* = 3 biologically independent experiments, individual data points are overlaid, controls are unfolded scaffold and staple mixture ‘sc + st’ and scaffold only ‘sc’, 0.5 µg total DNA per condition, source data provided. Statistical analysis in **d** was performed using one-way ANOVA with Tukey’s multiple comparison, while statistical analysis for **f** and **g** was performed using two-tailed Student’s *t*-test (*p ≤ 0.05, **p ≤ 0.01, ***p ≤ 0.001, ****p ≤ 0.0001, ns *p* > 0.05).

We observed that electroporating a premixed, non-annealed cocktail of ssDNA scaffold and staple strands, which do not form structured objects (Supplementary Fig. 7), resulted in slightly higher transfection efficiency compared to administering the ssDNA scaffold alone (Fig. 1c, e). We hypothesized that partial association occurs between the staple and scaffold strands, resulting in double-stranded DNA regions around the promoter regions that enhance gene expression. We thus tested whether simply increasing the average staple length in a DNA origami object would lead to enhanced expression, which was not the case (Supplementary Fig. 8), suggesting that a more targeted design is required. We redesigned the 20HB object to incorporate long continuous staple segments with no crossovers in the promoter region, resulting in a structure with continuous 93-mer and 154-mer staples flanking the expression region at the 5’ start region of the CMV promoter, and at the 3’ end of the polyA sequence (20HB-LP and 20HB-LPv2, respectively). A schematic of 20HB-LPv2 design and staple localization is given in Fig. 2a. Inspired by the partially double-stranded hepatitis B genome^54^, we also prepared a design in which a 200-mer staple acts as a splint between the 5’ start of the CMV and the 3’ end of the polyA to form a partially double-stranded circular structure when unfolded (20HB-Circ) (Fig. 2b). All designs folded readily into defined 20HBs, as seen by direct imaging by TEM (Fig. 2c, Supplementary Fig. 9). Delivery of these objects into cells resulted in up to 50% enhancement of gene expression efficiency for objects 20HB-LP, 20HB-LPv2, and 20HB-Circ when compared to the standard 20HB staple routing (Fig. 2d).

Next, we determined whether the orientation of the target gene on the scaffold impacts gene expression. As the scaffold is ssDNA, delivery of the coding strand requires synthesis of the complementary sequence (template strand) prior to transcription. We created a ‘template strand’ scaffold with the reverse complementary gene sequences (sc_EGFP2, Fig. 2e). Delivering these scaffolds with and without staples demonstrated significantly increased transfection efficiency for the template strand compared to the coding strand (Fig. 2f). However, when folded into the 20HB DNA origami object, the difference in transfection efficiency disappeared (Fig. 2f, right, Supplementary Fig. 11). The overall transfection efficiency of the 20HBs thus does not depend on having either the coding or template strand as the scaffold. Yet we observed a minor trend of increased mean fluorescent intensity (MFI) of EGFP in EGFP-positive cells for all objects with the template-strand scaffold relative to those where the coding strand was used as scaffold (Fig. 2g). Characterization of all sc_EGFP2 structures is given in Supplementary Fig. 10.

### Enhancing gene expression with scaffold sequence features

**Fig. 3:**
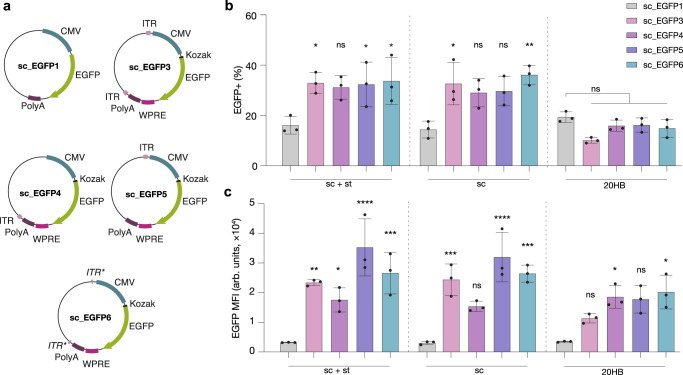
Enhanced gene expression from alternative scaffold sequences. **a** Scaffold designs where sc_EGFP1 represents the initial scaffold design, and sc_EGFP3/4/5/6 include additional sequence features such as ITRs (light pink), or ITR binding domains (ITR*), Kozak sequence (black), and WPRE (dark pink). **b**, **c** Comparison of transfection efficiency in HEK293T cells as determined by EGFP+ cells (**b**) and mean fluorescent intensity of EGFP+ cells (**c**). For condition sc + st, scaffold sc_EGFP3 (*p* = 0.0267), sc_EGFP5 (*p* = 0.0361) and sc_EGFP6 (*p* = 0.0168) demonstrated a higher percentage of EGFP+ cells, while scaffolds sc_EGFP3 (*p* = 0.0011), sc_EGFP4 (*p* = 0.0499), sc_EGFP5 (*p* = 3.10 × 10^−7^) and sc_EGFP6 (*p* = 0.0001) demonstrated higher MFI compared to sc_EGFP1. For condition sc, scaffold sc_EGFP3 (*p* = 0.0118) and sc_EGFP6 (*p* = 0.0014) demonstrated higher percentage of EGFP+ cells, while scaffolds sc_EGFP3 (*p* = 0.0005), sc_EGFP5 (*p* = 2.52 × 10^−6^) and sc_EGFP6 (*p* = 0.0001) demonstrated higher MFI compared to sc_EGFP1. For 20HB structures, only those folded with sc_EGFP4 (*p* = 0.0317) and sc_EGFP6 (*p* = 0.0113) demonstrated higher EGFP MFI than 20HBs folded with sc_EGFP1. Data is replotted in Supplementary Fig. 14. Data collected in **b** and **c** were quantified using flow cytometry and are presented as mean ± s.d. for *n* = 3 biologically independent experiments, individual data points are overlaid, controls are unfolded scaffold and staple mixture ‘sc + st’ and scaffold only ‘sc’, 0.5 µg total DNA per condition, source data provided. Statistical analysis was performed using one-way ANOVA with Tukey’s multiple comparison (*p ≤ 0.05, **p ≤ 0.01, ***p ≤ 0.001, ****p ≤ 0.0001, ns *p* > 0.05).

To further enhance the gene expression, we included additional features in the scaffold sequence based on ssDNA AAV2 expression cassettes (Fig. 3a). We placed a Kozak sequence, which functions as a protein translation site^45^, and a chimeric intron, upstream of EGFP; and a woodchuck hepatitis virus post-transcriptional regulatory element (WPRE) downstream of EGFP (before the polyA). The WPRE is thought to improve mRNA stability and protein yield^46^. In addition, we included inverted terminal repeats (ITRs) flanking the expression cassette. ITRs are palindromic sequences which form a T-shaped hairpin, and are used by adeno-associated viruses as an origin of replication for their ssDNA genome, in addition to other functions^47,55^. We attempted producing a synthetic scaffold ssDNA containing all of these features (sc_EGFP3, Fig. 3a). The scaffold was produced at low yield and low quality, which we attributed to the repetitive ITR structures. To improve scaffold yield and quality we produced a further series of scaffolds that included only a single ITR downstream or upstream of the expression cassette (sc_EGFP4 and sc_EGFP5, respectively). In addition, we created a scaffold that includes partial sequences of both ITRs, but where the ITR hairpin would be provided by a complementary staple oligonucleotide during DNA origami folding (schematic Supplementary Fig. 12, sc_EGFP6). 20HBs with standard staple designs were produced for all these scaffold variants, however, the origami objects folded with low yields and purity (Supplementary Fig. 13).

We observed a trend of increased transfection efficiency and enhanced gene expression in the scaffold-only controls in all cases relative to the original sc_EGFP1 scaffold (Fig. 3b, c, Supplementary Fig. 14). The 20HB samples folded from the “enhanced” scaffolds demonstrated a similar range of transfection efficiencies relative to those observed for 20HB folded from the sc_EGFP1 scaffold but the MFI was significantly increased in the positive cells for 20HBs folded from the “enhanced” scaffolds sc_EGFP3/4/5/6 relative to the original sc_EGFP1 (Fig. 3b), meaning that the additional features in the scaffolds enhanced intracellular gene expression.

**Fig. 4:**
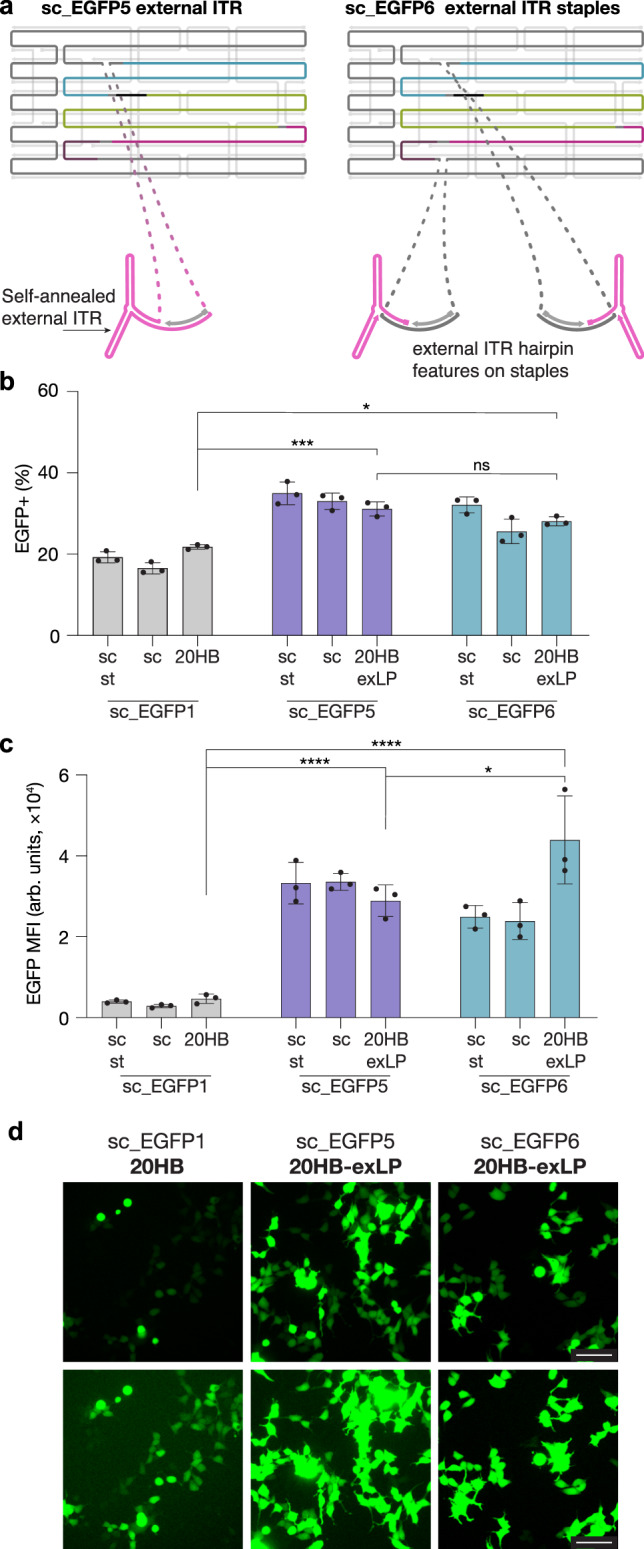
Enhancing gene expression through scaffold sequence design. **a** 20HB design for the sc_EGFP5 structure included an external single-stranded loop to allow the ITR sequence to self-anneal and form the hairpin structure. 20HB design for the sc_EGFP6 scaffold included two external loops to expose the ITR binding domain, enabling the ITR hairpin staples to anneal. **b**, **c** Transfection efficiency and MFI in HEK293T cells seen for 20HB-exLP structures folded with sc_EGFP5 and sc_EGFP6. Higher transfection efficiencies and MFI were observed for 20HB-exLP objects folded with sc_EGFP5 (*p* = 0.0002, *p* = 7.99 × 10^−5^) and sc_EGFP6 (*p* = 0.0134, *p* = 4.6 × 10^−8^) compared to sc_EGFP1 20HB. In addition, 20HB-exLP folded with sc_EGFP6 demonstrated higher MFI than 20HB-exLP folded with sc_EGFP5 (*p* = 0.0169). Data collected in **b** and **c** were quantified using flow cytometry and are presented as mean ± s.d. for *n* = 3 biologically independent experiments, individual data points are overlaid, controls are unfolded scaffold and staple mixture ‘sc + st’ and scaffold only ‘sc’, 0.5 µg total DNA per condition, source data provided. Statistical analysis in **b** and **c** was performed using one-way ANOVA with Tukey’s multiple comparison, (*p ≤ 0.05, ***p ≤ 0.001, ****p ≤ 0.0001, ns *p* > 0.05). **d** Representative epifluorescence microscopy images showing EGFP expression from cells transfected with DNA origami objects folded with sc_EGFP5 and sc_EGFP6 relative to sc_EGFP1. Images in the bottom row have been purposely contrast enhanced to reveal EGFP positive cells that have poor EGFP intensity in the sc_EGFP1 sample. The images are representative of one of *n* = 3 biologically independent experiments; similar results were observed each time. Scale bar 100 µm.

In our designs discussed thus far, the ITR sequences were masked within double-helical DNA domains in the object, to become available only once the object denatures within the cell. We hypothesized that the gene expression from the 20HB may be further improved by positioning the ITR sequence motif such that it can already assemble into its hairpin secondary structure during folding of the object (Fig. 4a, design 20HB-exL). In addition, we included a continuous 154-mer staple at the 5’ region of the promoter for design 20HB-exLP, encoded with both sc_EGFP5 and 6 scaffolds (Supplementary Fig. 15 for object characterization). Indeed, delivery of these designs demonstrated up to twofold transfection enhancement (Fig. 4b) and up sixfold and ninefold increased expression efficiency, respectively, as measured by MFI (Fig. 4c, d) compared to the original 20HB design using the sc_EGFP1 scaffold.

**Fig. 5:**
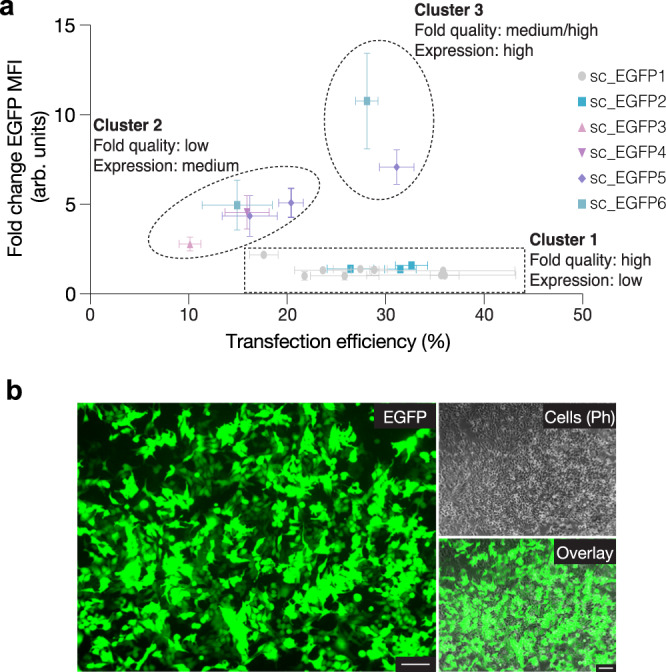
Transfection summary and optimization. **a** Comparison summary of transfection efficiency (%) and EGFP MFI (arb. units) across all structures investigated in HEK293T cells, grouped by scaffold. sc_EGFP1 20HB-ext was used as an internal control in all experiments, and EGFP MFI is represented as fold change compared to this sample. For each sample *n* ≥ 3 biologically independent experiments, and is represented as mean ± s.d, source data provided. Three clusters are highlighted: 1, structures with high folding quality but low overall gene expression; 2, structures with low folding quality and medium expression levels; 3, structures with medium to high folding quality and high expression levels. **b** Representative epifluorescent microscopy images showing the expression of EGFP by successfully transfected HEK293T cells after optimization of electroporation settings, where EGFP expression (green), cell (phase contrast) and the overlay are given. The images are representative of one of *n* = 2 biologically independent experiments; similar results were observed each time. Scale bar 100 µm.

We plotted the transfection efficiency achieved against the relative EGFP MFI (arb. units) for all designs, normalized to the sc_EGFP1 20HB-ext as an internal control (Fig. 5a). The samples separate into three clusters. Cluster 1 includes objects built from sc_EGFP1/2 scaffolds, which have a high quality of folding and high transfection efficiencies but featured low overall gene expression. Cluster 2 includes objects based on sc_EGFP3/4/5/6 scaffolds which had low quality of folding and low transfection efficiencies but enhanced gene expression. Cluster 3 then had objects with improved folding quality, as is the case with 20HB-exLP for sc_EGFP5 and 6 scaffolds, and improved transfection efficiency and improved gene expression. Finally, we further optimized the transfection efficiency by titrating the amount of material administered and by varying the electroporation conditions, resulting in even higher transfection efficiencies (~80%) and MFIs (Fig. 5b, Supplementary Fig. 16, 17 and Supplementary Table 4).

### Multiplexed gene assemblies for cotransfection

**Fig. 6:**
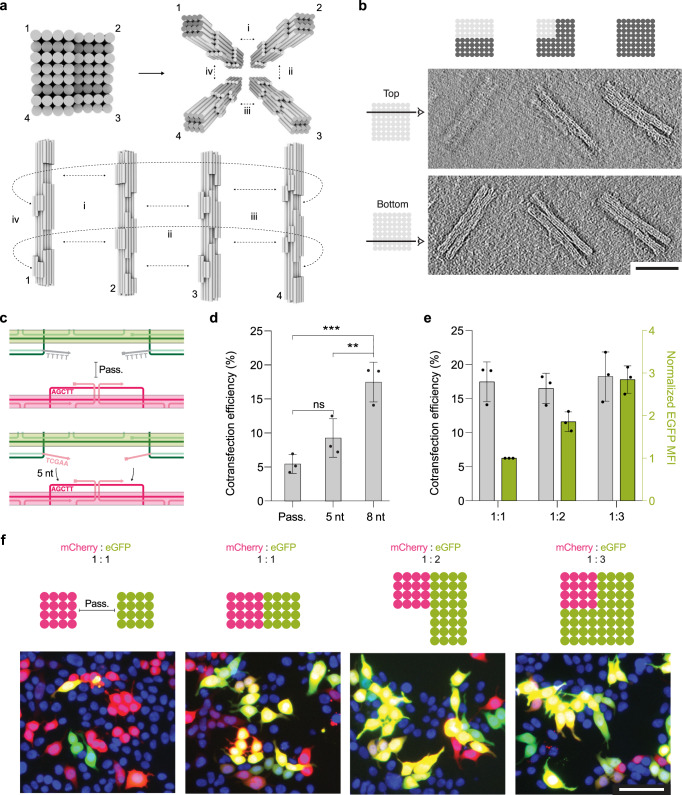
Delivery of multimeric origami assemblies enables codelivery of genes in defined ratios. **a** Cylindrical models of DNA origami objects for programmed assembly via shape-complementary protrusions and recesses. Schematic demonstrates unique interaction patterns to build dimer (i), trimer (i and ii) and tetramer (i, ii, iii and iv) higher-order assemblies. **b** Representative comparison tomogram slice through dimer, trimer, and tetramer structures, scale bar 100 nm, taken from a sample with mixed assembly products. **c** Schematic demonstrating passivated overhangs for inhibiting assembly, and assembly assisted via complementary 5 nt sticky ends. **d** Cotransfection (mCherry+/EGFP+) efficiency in HEK293T cells after delivery of mCherry and EGFP as individual monomers (Pass.), or as a dimer connected through 5 nt or 8 nt sticky ends. Dimers with 8 nt sticky ends demonstrated higher cotransfection efficiency than passivated dimers (*p* = 0.0002) and dimers with 5 nt sticky ends (*p* = 0.0074). **e** Cotransfection (mCherry+/EGFP+) efficiency in HEK293T cells with assembled multimeric DNA origami structures including mCherry and EGFP-encoded monomers in ratios of 1:1, 1:2, and 1:3 mCherry:EGFP. EGFP MFI (arb. units) is given on the right *y*-axis. Data collected in **d** and **e** were quantified using flow cytometry and are presented as mean ± s.d. for *n* = 3 biologically independent experiments, individual data points are overlaid, source data provided. 0.125 µg of each DNA origami monomer was used per condition. Statistical analysis in **d** was performed using one-way ANOVA with Tukey’s multiple comparison, (**p ≤ 0.01, ***p ≤ 0.001, ns *p* > 0.05). **f** Top: schematic design of mCherry and EGFP monomer blocks for no assembly (passivated), or assembly into dimer, trimer or tetramer structures in the ratio of 1:1, 1:2, and 1:3 mCherry:EGFP, from left to right. Bottom: representative epifluorescent microscopy images demonstrated the expression of mCherry (red), EGFP (green), or coexpression (yellow) by successfully transfected HEK293T cells. Cell nuclei are given in blue, scale bar 100 µm. The images are representative of one of *n* = 3 biologically independent experiments; similar results were observed each time.

We designed DNA origami objects encoding for either mCherry or EGFP expression to enable assembly and delivery in stoichiometric ratios of 1:1, 1:2, and 1:3 mCherry to EGFP (Fig. 6a, b, using scaffolds sc_mCherry5 and sc_EGFP5). The individual gene “blocks” were programmed to interact with each other via shape-complementary docking sites^16^ lined with sequence-complementary sticky-ends which were either five or eight base pairs long (referred to as 5 nt or 8 nt sticky ends, respectively). We also made control objects that had deactivated docking sites passivated with five thymidine long single-strand overhangs (Fig. 6c). Gene assemblies of either dimer, trimer or tetramers formed as designed, as we saw by negative-staining TEM tomography and agarose gel electrophoresis (AGE, Fig. 6b, Supplementary Fig. 18). The cotransfection efficiency, measured as the cell population positive for both EGFP and mCherry expression, for a 1:1 stoichiometric mixture of passivated (non-connected) mCherry and EGFP monomers was ~5.4 ± 1.4%. By contrast, we observed ~17.5 ± 2.9% cotransfection efficiency when using the pre-assembled dimer object (8 nt sticky ends) that included both mCherry and EGFP as expressible genes (Fig. 6d, f). The near four-fold increase in cotransfection relative to when delivering the genes in separate objects indicates that the delivery and expression of both components is now linked, and no longer occurs at random.

Finally, we delivered the multimeric origami objects in the form of a dimer, trimer, and tetramer with the ratios of 1:1, 1:2, and 1:3 mCherry:EGFP. The molar concentration of the multimeric origami objects was conserved across samples and the total cotransfection efficiency thus remained comparable (Fig. 6e, gray bars). However, the expression level of EGFP was directly proportional to the number of monomers present within the object (Fig. 6e (green bars)). Direct imaging of cells using fluorescence microscopy agreed with the observations made in flow cytometry (Fig. 6f). Therefore, we succeeded delivering and expressing genes in a designed, stoichiometric ratio simply by “clicking” the genes together in a higher-order DNA origami assembly.

In summary, we investigated the expression of genes folded into DNA origami objects and determined scaffold and structural design features to enable efficient gene expression. We also demonstrated co-delivery of genes in controlled stoichiometric ratios. The scaffolds designed herein provide a reporter gene framework to quantify the functionality of a DNA origami-based gene delivery systems, and to probe the intracellular fate of DNA origami objects. By replacing the fluorescent reporter protein genes with sequences encoding for custom proteins, our scaffolds can also be adapted for custom delivery purposes. Inclusion of responsive crosslinkers, such as pH switchable triplex structures or photocleavable linkers, may enable spatio- and/or temporal control over DNA object unfolding and expression. In addition, the controlled co-delivery of custom genes via higher-order DNA origami assemblies could facilitate for example the delivery of synergistic genes for therapeutics and/or gene circuits. Traditional means of co-delivery would involve mixing the genetic materials in the desired ratio, but then having them distributed randomly either upon complexation with the delivery agent (e.g., liposome, polymer, or inorganic nanoparticle), or via direct cellular delivery (electroporation), which faces important challenges in an in vivo context. Future work will include further optimizing the multi-component origami gene assembly, which will be an important step towards creating a gene editing or modulation origami platform. Further, HEK293T cells were utilized as a model cell line due to ease of transfection, however further optimization may be required when translating the technology to other cell lines, or primary cells. Nevertheless, with our system, a set of “clickable” DNA origami bricks can now be envisioned that include, e.g., a CRISPR-based expression cassette^4^, which could be multiplexed with different combinations of guide RNAs or other helper genes in controlled stoichiometric ratios for codelivery.

## Methods

### Scaffold production

The design and cloning methods of our customized scaffolds are given in detail in Supplemental Information. In brief, gene fragments from EGFP-containing plasmids (Addgene plasmids #13031 and #105530, with and without ITR sequences respectively) were assembled with a fragment for phage origin of replication bacterial resistance (Addgene plasmid #126854) using either Golden Gate or digestion ligation cloning. Plasmids were verified using restriction digests and DNA sequencing (Eurofins genomics, Ebersberg Germany). Exact primer sequences and methods can be found in Supplementary Table 1, and sequences of the custom scaffolds can be found in Section 2.2 in Supplemental Information.

For the production of the ssDNA custom scaffolds^40,56^, chemically competent DH5α E. coli cells were cotransformed with the plasmid of interest, and a helper plasmid (Addgene plasmid #120346). Single colonies were picked and grown for ~10 h in a 5 mL pre-culture (2×YT, 30 µg/mL kanamycin, 30 µg/mL carbenicillin) before being transferred to 750 mL of 2×YT (30 µg/mL kanamycin, 30 µg/mL carbenicillin, 5 mM MgCl_2_) in Ultra Yield flasks (Thomson). Cells then were grown in a shaking incubator at 37 °C overnight. Bacteria were pelleted by centrifugation (45 min, 4500 × *g*, and the supernatant collected. Phagemid particles were precipitated from the supernatant with addition of polyethylene glycol 8000 (PEG-8000, final concentration 3% w/w) and NaCl (final concentration 0.5 M) and incubated with stirring for 1 h at rt, before being collected by centrifugation (45 min, 4500 × *g*, 4 °C). The pellet was resuspended in 4 mL of 1× TE buffer (10 mM Tris, 1 mM EDTA, pH 8) and centrifuged again (15 min, 16,000 × *g*, 4 °C) to remove residual bacterial components. The ssDNA scaffold was then extracted via phagemid lysis and purified via ethanol precipitation. Representative AGE for all custom ssDNA scaffolds is given in Supplementary Fig. 1.

### DNA origami design, folding, and purification

DNA origami objects were designed using caDNANo sq v0.1 and caDNAno v2. All origami objects were folded in standardized ‘folding buffers’ containing *x* mM MgCl_2_ in addition to 5 mM Tris base, 1 mM EDTA and 5 mM NaCl, pH 8 (FoB*x*). All reactions were subjected to thermal annealing ramps in Tetrad (Bio-Rad) thermal cycling devices. Exact folding conditions for each structure is given in Supplementary Tables 2 and 3. Staple strands were purchased from Integrated DNA Technologies, listed in Supplementary Tables 5–28, and used with standard desalting unless stated otherwise. Origami scaffold and staple routing are given in Supplementary Figs. 20–41. Origami objects were purified by either PEG precipitation, or gel extraction^57,58^.

### Assembly of multi-component DNA origami objects

To assemble the origami subunits to form the dimer, trimer, and tetramer samples, monomers were mixed in molar ratios in 1 × FoB5 buffer and incubated for 48 h at 37 °C. Passivated samples were treated identically.

### UV welding

UV weldable samples were designed with additional thymine bases located at all potential staple crossover position, and UV-crosslinked with UV light (310 nm, 2 h) using Asahi Spectra Xenon Light source (300 W, MAX-303) with a high transmission bandpass filter centered around 310 nm (XAQA310, Asahi Spectra)^51^. Samples were in FoB10 buffer at the time of UV-crosslinking.

### Urea-PAGE purification of ultramers

Long staple oligomers (93-mers, 154-mers, and 200-mers) were purchased from IDT as ultramers and purified in-house via denaturing urea polyacrylamide gel electrophoresis (Urea-PAGE). Bands corresponding to the correct MW were cut away and crushed prior to the addition of 1 × TEN buffer (10 mM Tris-HCl, 1 mM EDTA, 100 mM NaCl, pH 8.00). Pure ultramers were recovered via EtOH precipitation, redissolved in MilliQ H_2_O, and stored at 4 °C.

### Gel electrophoresis

For characterization of PCR products and plasmids, 1% agarose gels containing 0.5 × TBE buffer (22 mM tris base, 22 mM boric acid, 0.5 mM EDTA) were used. Gel electrophoresis was performed with an identical buffer solution for 1 h at a voltage of 110 V. To characterize assembled origami and scaffolds, we used 2% agarose gels containing 0.5 × TBE buffer and 5.5 mM MgCl_2_. Gel electrophoresis was performed with an identical buffer solution for 1–2 h at a voltage of 90 V; gels were placed in a water bath for cooling. All gels were imaged using a Typhoon FLA 9500 laser scanner (GE Healthcare) with a pixel size of 50 µm/pixel.

### Negative staining TEM

Samples were incubated on glow-discharged copper TEM grids (FCF400-CU, Electron Microscopy Sciences), for 30–60 s. Grids were then stained for 30 s (2% aqueous uranyl formate, 25 mM NaOH). Imaging was performed at magnifications of 21,000–42,000×. Data was acquired with SerialEM software, using a FEI Tecnai T12 microscope (120 kV, Tietz TEMCAM-F416 camera). Images were processed using ImageJ^59^. TEM micrographs were high-pass filtered to remove long-range staining gradients and the contrast was auto-leveled using Adobe Photoshop CS5.

The tilt series were performed from −50° to +50° and micrographs were acquired in 2° increments, the tomogram was then generated using a filtered back-projection, processed with Etomo (IMOD) to acquire tomograms^60^. The Gaussian-Filter used a cutoff between 0.25 and 0.5, and a fall-off of 0.035.

### Cell culture

HEK293T cells (DSMZ, cat. no. ACC 635) were cultured routinely in Dulbecco’s modified Eagle’s medium (DMEM, Gibco, cat. no. 31966047), supplemented with 10% heat-inactivated fetal bovine serum (FBS, Sigma-Aldrich, cat. No. F9665). Cells were grown in a humidified incubator at 37 °C with 5% CO_2_.

### Electroporation

Electroporation experiments were carried out according to the Manufacturer’s protocol (Neon™ transfection protocol, Thermo Fisher). Briefly, HEK293T cells were washed with phosphate-buffered saline solution (PBS) and collected using TryplE. Cells were pelleted via centrifugation (5 min, 300 × *g*), resuspended in PBS and counted. Cells were centrifuged again (5 min, 300 × *g*), and then resuspended in Buffer R (Neon™ Transfection System) at a concentration of 5 × 10^6^ cells/mL. Mixtures for each condition were prepared so that each electroporation event contained 0.5 µg total DNA, and the volume was supplemented to a total of 1 µL with 1 × FoB5 buffer (folding buffer, 1 mM Tris, 1 mM EDTA, 5 mM NaCl, 5 mM MgCl_2_), which was mixed with 9 µL of the cell suspension. For all screening conditions, electroporation occurred in the 10 µL transfection tips, with two pulses at pulse voltage of 1150 V and width of 20 ms. For cotransfection experiments, the process was identical except for the fact that the electroporation occurred with one pulse at pulse voltage of 1600 V and width of 20 ms. After electroporation, cells were immediately transferred to a 48-well plate which had been pre-prepared with a poly-L-lysine coating, and 240 µL of complete DMEM growth media.

After 48 h, samples were imaged using the EVOS™ M7000 Imaging System, and the transfection efficiency was quantified via flow cytometry. Briefly, samples were acquired using Attune Nxt Flow Cytometer and software (Thermo Fisher). In total, 20,000 single cell events, gated on side scatter area versus height, were recorded for analysis. EGFP was excited with a 488 nm laser, and emission was measured with a 530/30 nm bandpass filter. Untreated cells, and cells electroporated with buffer only, were used as negative controls. Cells electroporated with the corresponding EGFP plasmid was used as a positive control. Data were analyzed post-acquisition using FlowJo software (v10.7.1, v10.8.1); exemplary gates are given in Supplementary Fig. 19.

### Statistics and reproducibility

Statistical analyses were performed with GraphPad Prism (GraphPad Software Inc. v9). The data is illustrated as the mean ± standard deviation, and the individual data points representing biological replicates are shown. The specific analysis performed is detailed in the corresponding figure caption. For all tests, differences were considered significant at *p ≤ 0.05, **p ≤ 0.01, ***p ≤ 0.001, ****p ≤ 0.0001. All samples presented in AGE gels are representative of *n* ≥ 2 independent AGE gel repeats. All TEM images are representative of samples imaged on *n* ≥ 2 independent TEM grid preparations.

### Reporting summary

Further information on research design is available in the Nature Portfolio Reporting Summary linked to this article.

## Supplementary information

Supplementary Information

Reporting Summary

## Data Availability

Data supporting the findings of this study are available within the paper and its Supplementary Information files Source data for each graph has been provided as Source Data 1 (excel), and uncropped gel images have been provided as Source Data 2 (pdf) in the Source Data file. Additional information including detailed methods for custom scaffold design and DNA origami folding, and all DNA scaffold and staple sequences are included in Supporting Information (PDF). The Supporting Information also includes supplementary characterization such as AGE, representative TEM, and further transfection experiments. Source data are provided with this paper.

## Source data

Source Data
