# Supplementary material for: Gene-encoding DNA origami for mammalian cell expression
Source: Nat Commun. 2023 Feb 23;14:1017. doi: 10.1038/s41467-023-36601-1 (PMC9950468; doi:10.1038/s41467-023-36601-1)
Supplement: Supplementary file 1 — Supplementary Information [file 41467_2023_36601_MOESM1_ESM.pdf]

Supplemental Information

Gene-encoding DNA origami for mammalian cell expression

Jessica A. Kretzmann[1,2], Anna Liedl[1,2], Alba Monferrer[1,2], Volodymyr Mykhailiuk[1,2], Samuel Beerkens[1,2], and Hendrik Dietz[1,2,*]

[1] Department of Biosciences, School of Natural Sciences, Technical University of Munich, Am Coulombwall 4a, 85748 Garching, Germany.

[2] Munich Institute of Biomedical Engineering, Technical University of Munich, Boltzmannstraße 11, 85748 Garching, Germany.

Contents

1. Materials and Methods 2
1.1 Scaffold cloning 2
1.2 Scaffold production 3
1.3 DNA origami folding 3
2. Supplemental Data 5
2.1 Characterisation and supporting information 5
2.2 Scaffold sequences and origami design 16
2.2.1 sc_EGFP1 16
2.2.1.1 sc_EGFP1 20HB-ext and 20HB-ext-W 17
2.2.1.2 sc_EGFP1 20HB-int and 20HB-int-W 20
2.2.1.3 sc_EGFP1 32HB 24
2.2.1.4 sc_EGFP1 12HB 26
2.2.1.5 sc_EGFP1 20HB-LS 28
2.2.1.6 sc_EGFP1 20HB-LP 30
2.2.1.7 sc_EGFP1 20HB-LPv2 32
2.2.1.8 sc_EGFP1 20HB-Circ 34
2.2.2 sc_EGFP2 36
2.2.2.1 sc_EGFP2 20HB 37
2.2.2.2 sc_EGFP2 20HB-LPv2 39
2.2.2.3 sc_EGFP2 20HB-Circ 41
2.2.3 sc_EGFP3 43
2.2.3.1 sc_EGFP3 20HB 44
2.2.4 sc_EGFP4 47
2.2.4.1 sc_EGFP4 20HB 48
2.2.5 sc_EGFP5 51
2.2.5.1 sc_EGFP5 20HB 52
2.2.5.2 sc_EGFP5 20HB-exL 54
2.2.5.3 sc_EGFP5 20HB-exLP 56
2.2.5.4 sc_EGFP5 16HB_2 58
2.2.6 sc_EGFP6 67
2.2.6.1 sc_EGFP6 20HB 68
2.2.6.2 sc_EGFP6 20HB-exLP 70
2.2.7 sc_mCherry5 73
2.2.7.1 sc_mCherry5 16HB_1 74

1. Materials and Methods

1.1 Scaffold cloning

Plasmids encoding for custom scaffolds were created via standard cloning techniques. All plasmids, with the exception of sc_EGFP3, were created via Golden gate assembly using either Esp3I (NEB cat. no. R0734), or BsaI-HF®v2 (NEB cat. no. R3733), together with T4 DNA ligase (NEB cat. no. M0202). For each plasmid, appropriate cute sites were introduced with PCR, and the assembly was conducted as per manufacturer's protocol.

The exception, sc_EGFP3, was assembled via digestion ligation, and compatible enzyme cut sites were added to the fragment of interest by means of PCR. Enzymes EcoNI and PacI (NEB cat. no. R0521 and R0547 respectively) were used to digest fragments of interest, and then ligated using NEB T4 DNA ligase, as above.

Touchdown PCR with primers for all constructs are detailed in Table S1. In all cases, PCR products were confirmed by agarose gel electrophoresis (AGE), bands were excised, and fragments were extracted (Qiagen QIAquick Gel Extraction Kit) as per manufacturer's protocol.

Table S1 I Primer sequences used for the construction of plasmids for custom scaffold production.

Plasmid	Template	Primer	Sequence 5' - 3'
sc_EGFP1	Addgene plasmid #126854	FWD	CTGGATGGTCTCCgtgacattaagcgcggcgggtg
		REV	CTGGATGGTCTCCaatgagtgagcaaaaggccagca
	Addgene plasmid #13031	FWD	CTGGATGGTCTCCcattgatatacgcgttgacattga
		REV	CTGGATGGTCTCCtcacattccgcctcagaagccat
sc_EGFP2	Addgene plasmid #126854	FWD	CACTGACGTCTCTgtgacattaagcgcggcgggtg
		REV	CACTGACGTCTCTaatgagtgagcaaaaggccagca
	Addgene plasmid #13031	FWD	CACTGACGTCTCTcattcgcctcagaagccataga
		REV	CACTGACGTCTCTtcacagatatacgcgttgacattga
sc_EGFP3	Addgene plasmid #105530	FWD	TTAATTAAcattaagcgcggcgggtgt
		REV	CCTAATTAAGGgtgagcaaaaggccagcaaa
sc_EGFP4	sc_EGFP3	FWD	CACTGACGTCTCTcattatgctctaggaagatcggaa
		REV	CGTGATCGTCTCTaatgtaagggtgagcaaaaggcca
sc_EGFP5	sc_EGFP3	FWD	CACTGACGTCTCTcattgccttaattaacattaagcgc
		REV	CACTGACGTCTCTaatgcgccatgctacttatctacg
sc_EGFP6	sc_EGFP3	FWD	AGATGGCGTCTCCttaggggcctcagtgagcgagcg
		REV	AGATGGCGTCTCCaggggcctcagtgagcgagcg
		FWD	AGATGGCGTCTCCcccttaattaacattaagcgcg
		REV	AGATGGCGTCTCCctaatTAAGGgtgagcaaaagg
sc_mCherry5	sc_EGFP5	FWD	TGGCTACGTCTCGgtgattggatccaatcaacctctg
		REV	TGGCTACGTCTCGaatgcctggacacctgtggaga
	Addgene plasmid #127813	FWD	TGGCTACGTCTCGcattattcgccaccatggtgag
		REV	TGGCTACGTCTCGtcacccgctcacttgtacagct

1.2 Scaffold production

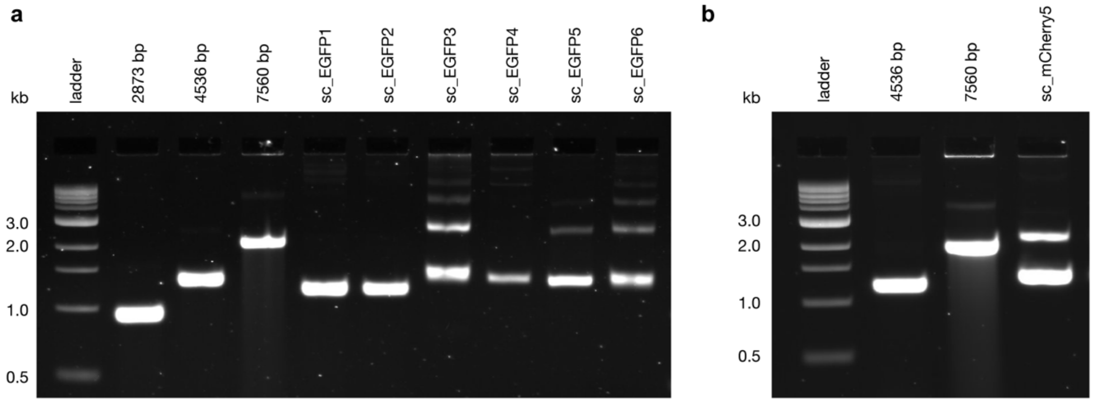

Fig. S1 I AGE showing all custom scaffolds created in this study. ssDNA markers at 2873 bp, 4535 bp and 7560 bp were used as molecular weight markers, while ‘ladder’ depicts NEB 1 kb dsDNA ladder.

1.3 DNA origami folding

Table S2 I Folding conditions summary for each of the structures and scaffolds used.

Scaffold	Structure	Program	10× Folding Buffer	v(scaffold, 100 nM,) μL*	v(staples, 100 μM), μL*
sc_EGFP1	20HB-ext	3	FoB10	10	8
	20HB-int	1	FoB10	10	8
	20HB-ext-W	1	FoB15	10	8
	20HB-int-W	1	FoB15	10	8
	32HB	1	FoB20	10	8
	12HB	2	FoB10	10	8
	20HB LS	3	FoB15	10	8
	20HB LP	1	FoB20	10	8
	20HB LPv2	1	FoB20	10	8
	20HB Circ	3	FoB10	10	8
sc_EGFP2	20HB	1	FoB10	10	8
	20HB LPv2	3	FoB15	10	8
	20HB Circ	3	FoB10	10	8
sc_EGFP3	20HB	5	FoB20	2.5	8‡
sc_EGFP4	20HB	2	FoB15	10	8
sc_EGFP5	20HB	3	FoB15	4	14
	20HB loop	5	FoB10	4	8
	20HB loop LPv2	4	FoB15	4	8
	16HB_2	7	FoB12.5	10	8
	16HB_3	7	FoB25	10	8
	16HB_4	7	FoB7.5	10	8
sc_EGFP6	20HB	2	FoB15	4	14
	20HB LPv2	6	FoB10	4	8
sc_mCherry5	16HB_1	1	FoB10	10	8

*For 20 μL folding reaction, where necessary volume supplemented to 20 μL total with ddH_2O.
‡ Staples used here were at 500 μM concentration.

Table S3 | Folding programs used for the folding reactions detailed in Table S2.

Program	1. Denaturation time 30 s	15 min	2. Temperature ramp	3. Storage temperature
**1**	-	65 °C	60–44 °C, at 1 °C/1 h	20 °C
**2**	-	65 °C	60–44 °C, at 1 °C/2 h	20 °C
**3**	70 °C	65 °C	60–35 °C, at 1 °C/1 h	20 °C
**4**	70 °C	65 °C	60–35 °C, at 1 °C/2 h	20 °C
**5**	70 °C	65 °C	60–35 °C, at 1 °C/4 h	20 °C
**6**	70 °C	65 °C	60–30 °C, at 1 °C/1 h	20 °C
**7**	-	65 °C	60–44 °C, at 1 °C/4 h	20 °C

2. Supplemental Data

2.1 Characterisation and supporting information

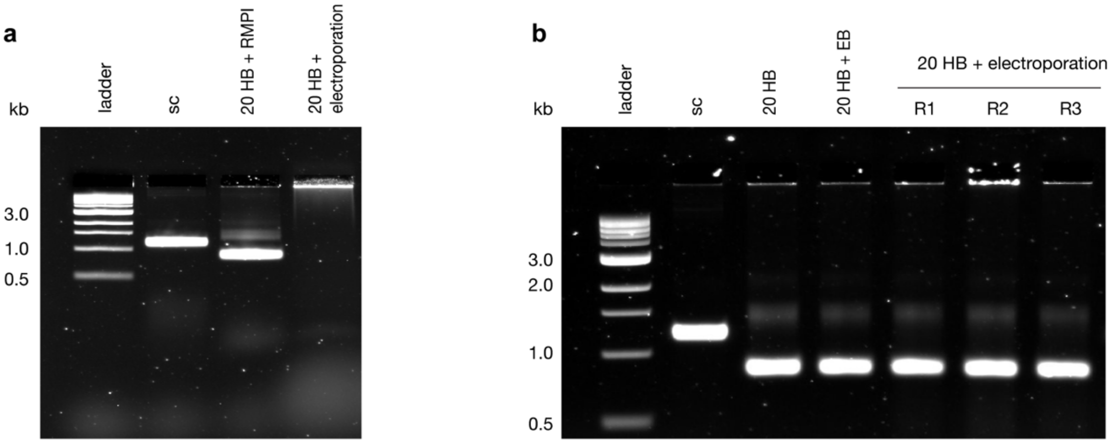

Fig. S2 l 20 HB structure integrity after electroporation. a, Structural integrity of the 20HB was maintained when diluted in RPMI 1640 media but demonstrated complete aggregation after electroporation in a traditional cuvette. **b**, 20HBs were stable in both the electroporation buffer (EB, buffer R in kit) and after electroporation using the Neon™ transfection system. R1-R3 depicts 3 independent replicates. 'Sc' is the corresponding sc_EGFP1 ssDNA scaffold strand, while 'ladder' depicts NEB 1 kb dsDNA ladder.

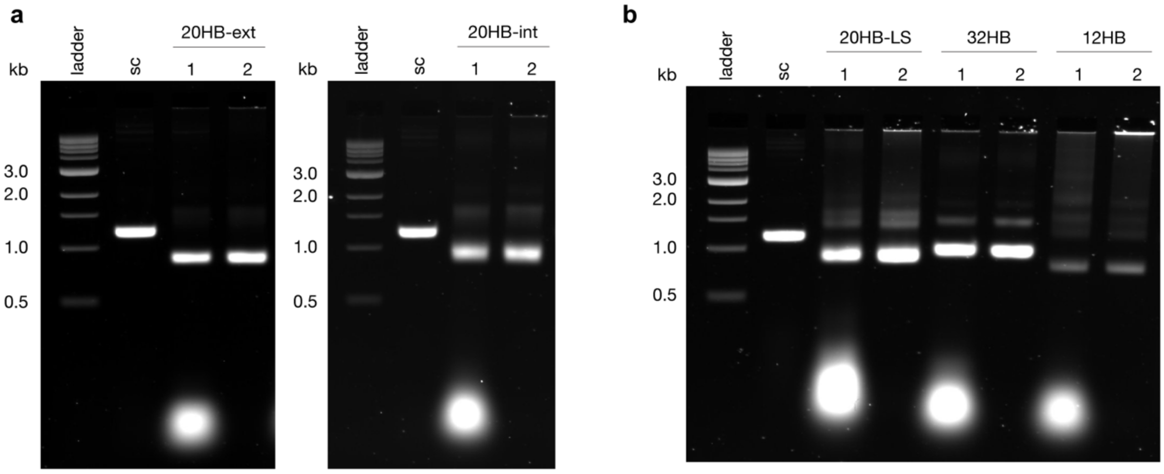

Fig. S3 l Purified DNA origami objects based on sc_EGFP1 scaffold. a, Crude and PEG-purified DNA origami objects are given in lane 1 and 2 for 20HB-ext and 20HB-int respectively. **b**, Crude and PEG-purified DNA origami objects are given in lane 1 and 2 for 20HB-LS, 32HB and 12HB respectively. The corresponding sc_EGFP1 ssDNA scaffold strand is given by 'sc', while 'ladder' depicts NEB 1 kb dsDNA ladder.

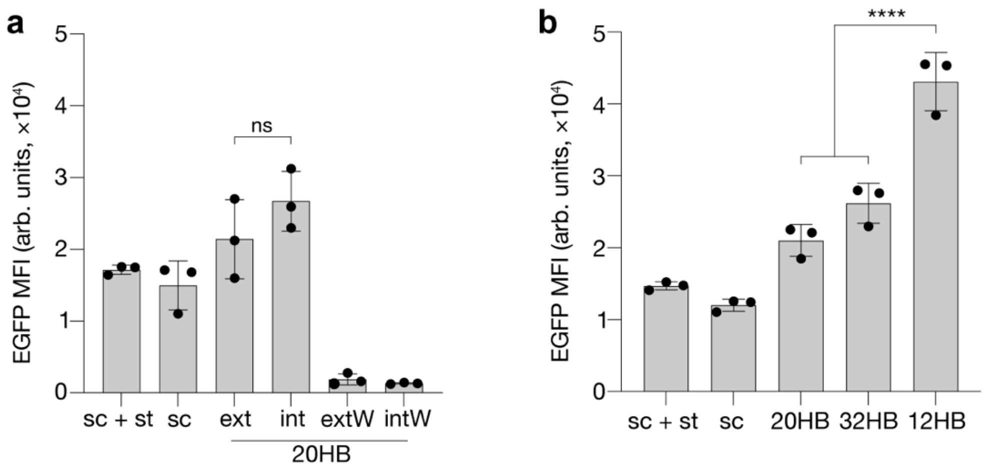

Fig. S4 | Mean fluorescence intensity of EGFP depending on gene orientation. a, HEK293T cells transfected with either 20HB-ext or 20HB-int demonstrated no significant difference in the EGFP MFI. **b**, HEK293T cells transfected with either 20HB(-ext) or 32HB demonstrated no statistically significant difference in EGFP MFI. Transfection with 12HB demonstrated significantly higher EGFP MFI compared to both 20HB(-ext) and 32HB. Data was collected quantified using flow cytometry and are presented as mean ± s.d. for n = 3 biologically independent experiments, individual data points are overlaid. Statistical analysis in was performed using one-way ANOVA with Tukey's multiple comparison (****p ≤ 0.0001, ns p > 0.05).

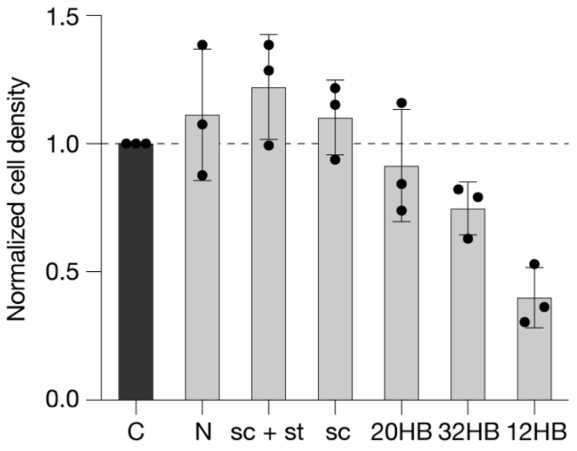

Fig. S5 | Cell density after electroporation of HEK293T cells. Single cell density was counted via AttuneNxt Flow Cytometer 48 h after electroporation. 'C' represents untreated control, 'N' represents control electroporated with blank buffer. Cell densities were normalized against control C. The data is presented as mean ± s.d. with individual data points overlaid, the data given is from n = 3 biologically independent experiments.

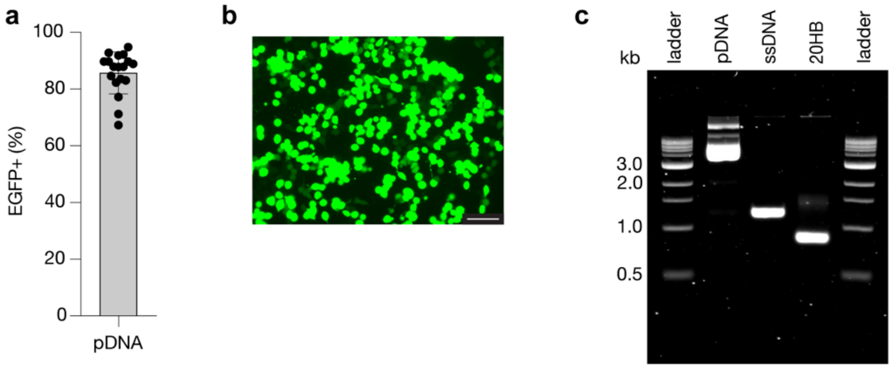

Fig. S6 l Electroporation in HEK293T cells using plasmid EGFP-encoding plasmid DNA (pDNA). a, Transfection efficiency in HEK293T cells, with representative epifluorescent image given in **b**. The data is presented as mean ± s.d. with individual data points overlaid, the data given is from n = 18 biologically independent experiments, where similar results were observed each time. Scale bar is 100 μm. Plasmid DNA was used as a positive control, however cannot be directly compared to the electroporation of ssDNA and folded DNA origami objects due to differences in electrophoretic mobility, as demonstrated in **c**. Plasmid DNA is given as pDNA, and the corresponding EGFP-encoding ssDNA scaffold strand and 20HB origami object is given as ssDNA and 20HB respectively, while 'ladder' depicts NEB 1 kb dsDNA ladder.

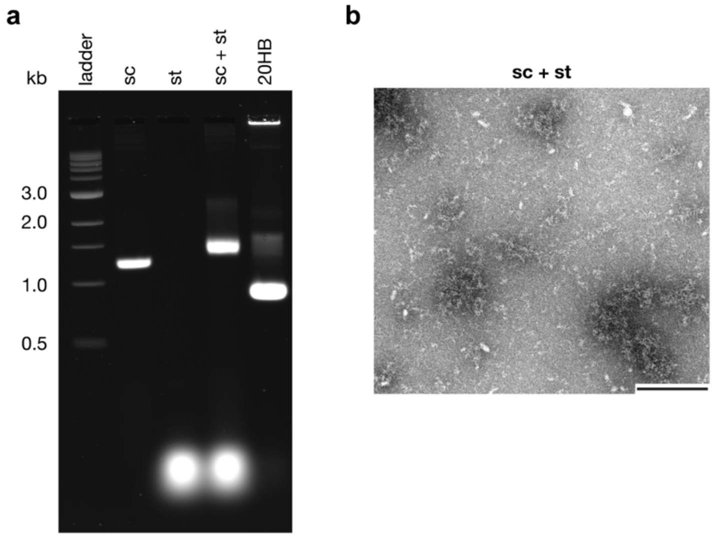

Fig. S7 l Staples associate with scaffold after mixing. a, Staples and scaffold were mixed ('sc + st') and incubated at rt for 10 min, while 20HB represents the corresponding purified folded object. A slight shift in the 'sc + st' band compared to scaffold only ('sc') demonstrates some staple association has occurred, but no structure has folded. 'st' is the corresponding mixture of staples, and 'ladder' depicts NEB 1 kb dsDNA ladder. **b**, Representative negative stain TEM image of the corresponding sc + st mixture. Scale bar 100 nm.

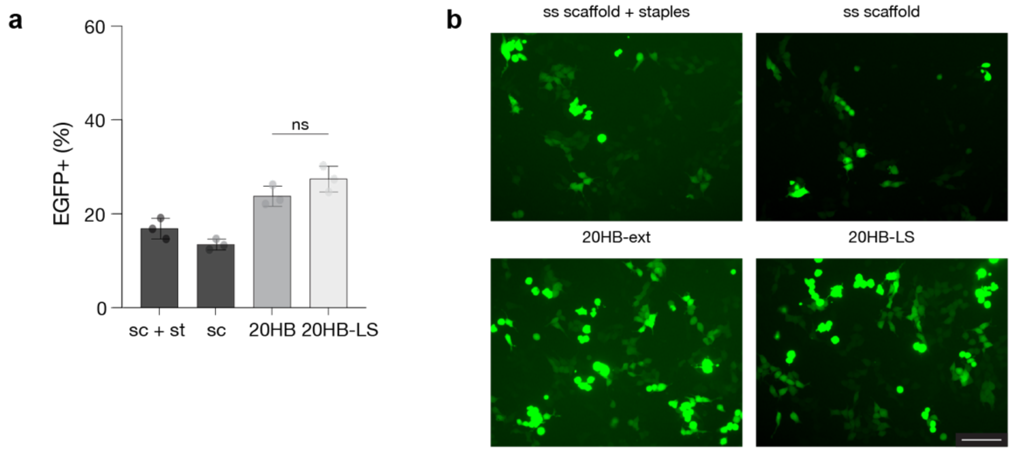

Fig. S8 | Electroporation of 20HB designs with different staple routings. 20HB with typical staple routings, as tested previously (20HB-ext, average staple length of ~ 42 bases) was compared to a 20HB design with longer staples and fewer staple crossovers (20HB-LS, average staple length of ~ 57 bases). **a**, Quantification of EGFP positive cells by flow cytometry is given in **a**. The data is presented as mean ± s.d. with individual data points overlaid. One-way ANOVA with Tukey's multiple comparison was performed to test statistically significant differences between samples. Both 20HB-ext and 20HB-LS demonstrated statistically significant increases in EGFP expression compared to ss scaffold + staples ($p < 0.05$, $p < 0.01$ respectively) and ss scaffold only ($p < 0.01$, $p < 0.001$ respectively). No statistically significant difference was observed between 20HB-ext and 20HB-LS (ns). Representative epifluorescent microscopy images demonstrating EGFP expression are given in **b**. The data in both **a** and **b** is representative of n = 3 biologically independent experiments, where similar results were observed each time. Scale bar is 100 μm.

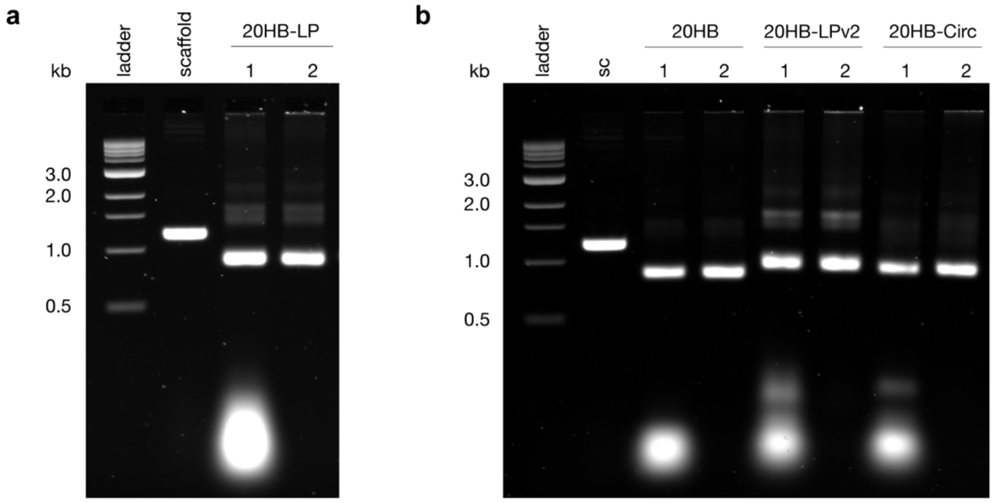

Fig. S9 | Purified DNA origami objects based on sc_EGFP1 scaffold with alternative staple designs. a, Crude and PEG-purified DNA origami objects are given in lane 1 and 2, respectively, for 20HB-LP. **b**, Crude and PEG-purified DNA origami objects are given in lane 1 and 2, respectively, for 20HB, 20HB-LPv2 and 20HB-Circ. The corresponding sc_EGFP1 ssDNA scaffold strand is given by 'sc', while 'ladder' depicts NEB 1 kb dsDNA ladder.

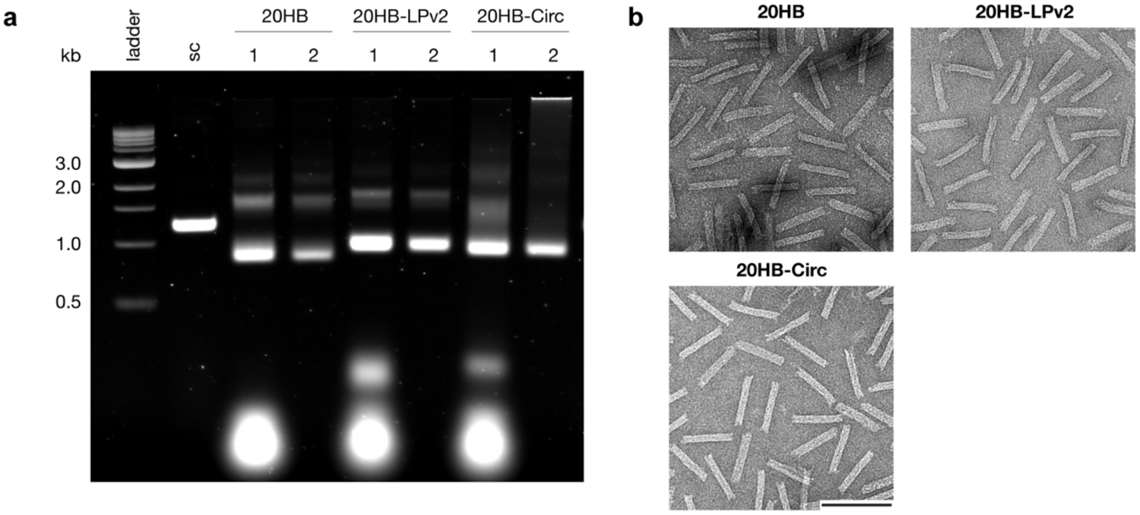

Fig. S10 l Purified DNA origami objects based on sc_EGFP2 scaffold. a, Crude and PEG-purified DNA origami objects are given in lane 1 and 2 for 20HB, 20HB-LPv2 and 20HB-Circ. The corresponding sc_EGFP2 ssDNA scaffold strand is given by 'sc', while 'ladder' depicts NEB 1 kb dsDNA ladder. **b**, Representative negative stain TEM images of the corresponding purified DNA origami objects. Scale bar 100 nm.

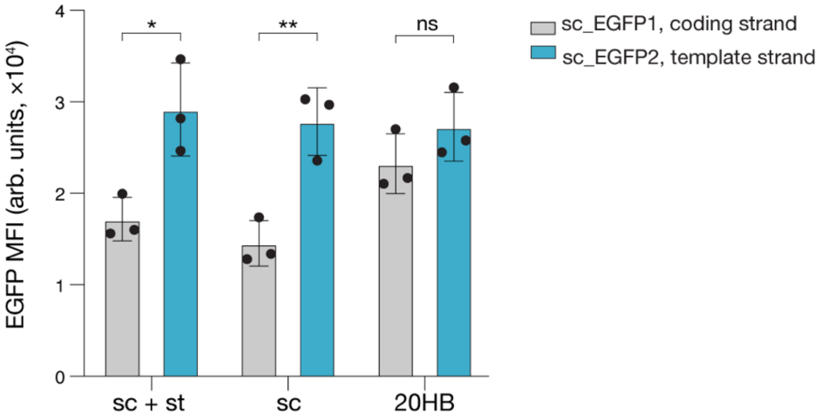

Fig. S11 l Mean fluorescence intensity of EGFP depending on scaffold orientation. HEK293T cells transfected with either sc_EGFP2 scaffold + staple mixture, or scaffold only, demonstrated significantly higher EGFP MFI than that of the sc_EGFP1 counterpart. No significant difference in the transfection efficiency from 20HB structures was observed. Data was collected quantified using flow cytometry and are presented as mean ± s.d. for n = 3 biologically independent experiments, individual data points are overlaid. Statistical analysis in was performed using Student's t-test (*p ≤ 0.05, **p ≤ 0.01, ***p ≤ 0.001, ns p > 0.05).

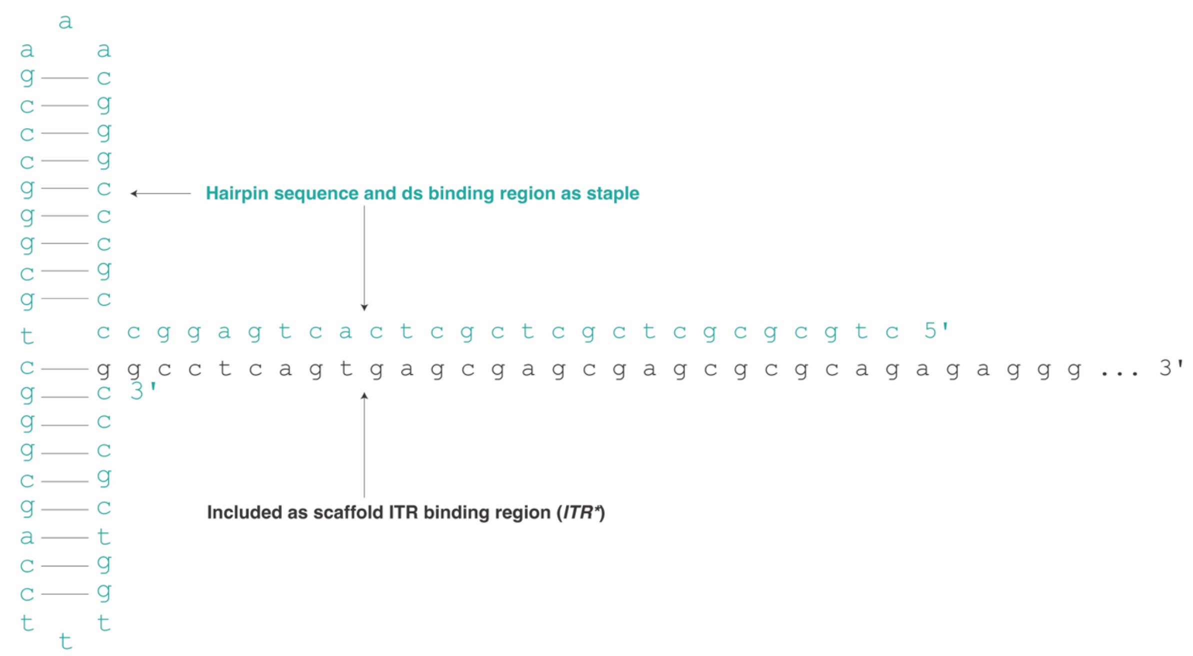

Fig. S12 l Exemplary schematic of ITR design for sc_EGFP6 scaffold. ITR hairpin and complementary binding sequence (green) was removed from the scaffold sequence, to be included as a separate staple. The sequence remaining in the scaffold (black) is given as the ITR binding region (*ITR**).

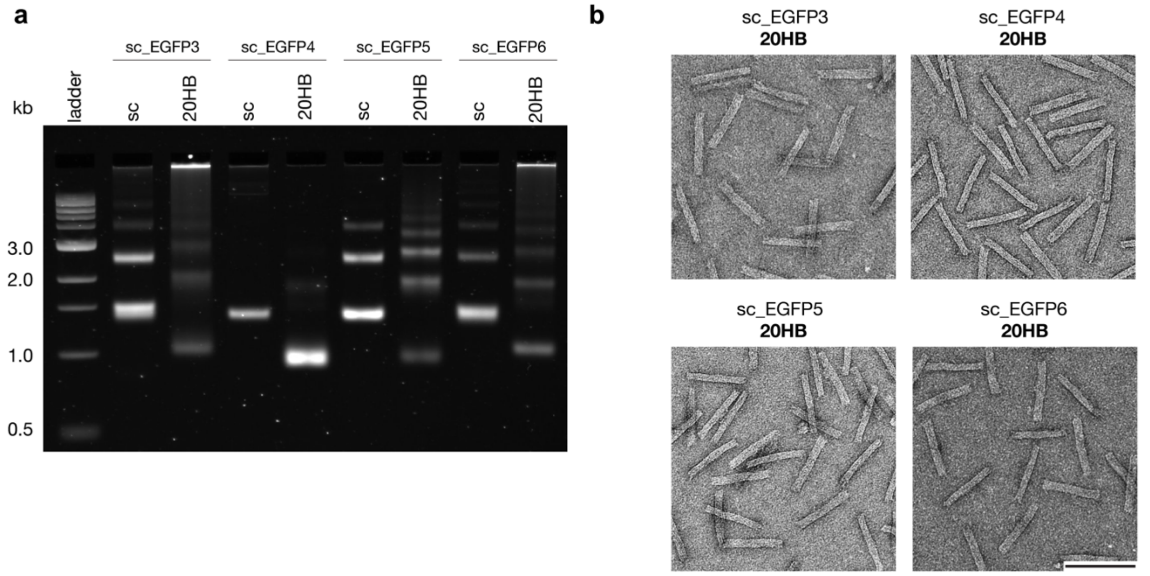

Fig. S13 l Purified DNA origami objects based on alternative scaffolds sc_EGFP3, sc_EGFP4, sc_EGFP5 and sc_EGFP6. a, Gel-purified origami objects. For each scaffold, the first lane ('sc') depicts the scaffold only MW control, while the second lane depicts the folded 20HB object. Higher order bands can be seen some of the scaffold samples, and in folded origami samples. In **a** 'ladder' depicts NEB 1 kb dsDNA ladder. **b**, Representative negative stain TEM images corresponding to each of the samples. Scale bar is 100 nm.

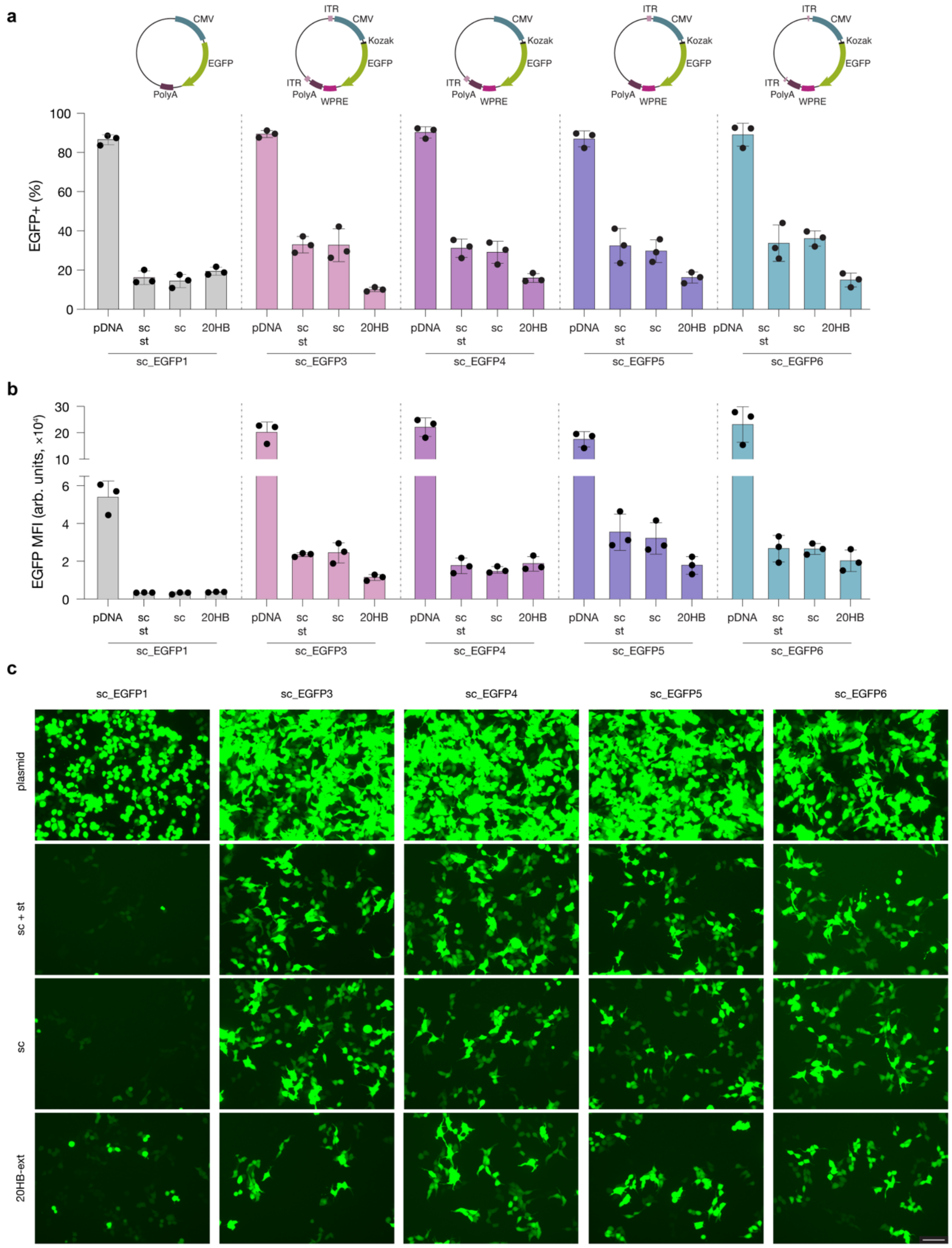

Fig. S14 I Screening expression from different scaffold sequence designs. To improve expression of ssDNA several sequence features, encoding for chimeric intron, kozak, WPRE, and ITRs were incorporated in the scaffold. Scaffolds with several ITR sequence derivatives were also investigated. Each ss scaffold DNA was expressed and folded into a 20HB. **a**, Plasmid/scaffold design schematics, CMV promoter (pink), kozak sequence (black), EGFP (green), WPRE (yellow), polyA (blue) and ITRs (purple) are shown. The ITR components are present in full at both upstream and downstream of the expression cassette (sc_EGFP3), only downstream (sc_EGFP4) and only upstream (sc_EGFP5). The final design (sc_EGFP6) has been constructed so that the only D-D' and A-A' ITR sequences are present on the scaffold strand, and the hairpin B-B', C-C', and corresponding A-A' sequence was supplied on staples strands. Plasmid, ss scaffold + staples (sc + st), ss scaffold (sc) and 20HB origami with the various sequences were electroporated into HEK293T cells and quantified by flow cytometry, looking at population of EGFP positive cells **a**, and mean fluorescent intensity (arb. units) **b**. Data is presented as mean ± s.d. with

individual data points overlaid. Representative epifluorescent microscopy images for each of the conditions is given in **c**. All data **a**–**c** is representative of n = 3 biologically independent experiments, where similar results were observed each time. Scale bar is 100 μm.

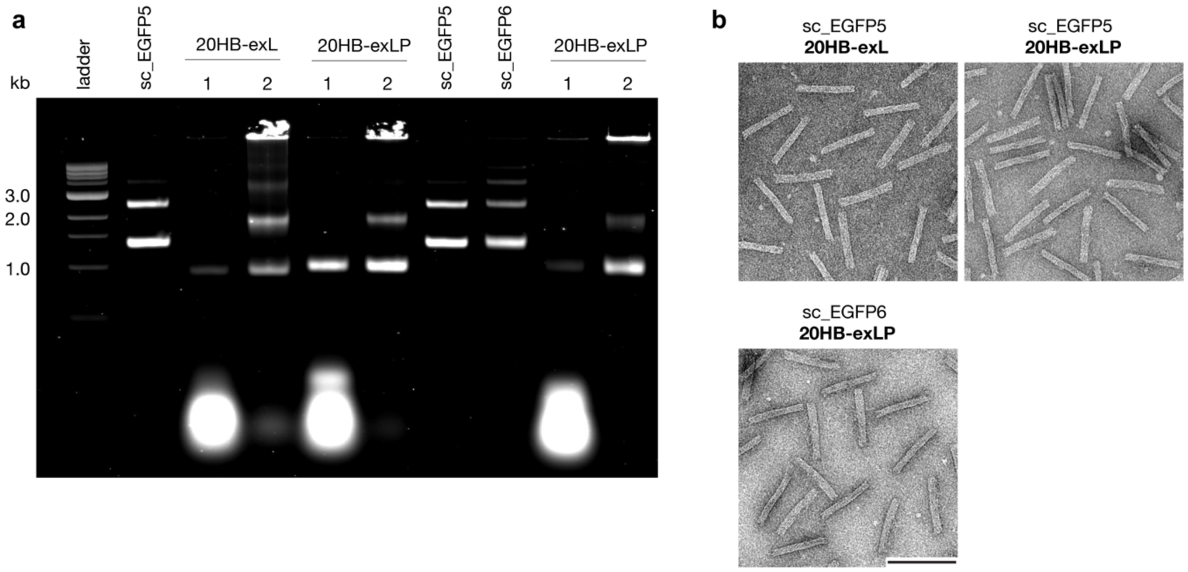

Fig. S15 | Purified DNA origami objects based on alternative scaffolds sc_EGFP5 and sc_EGFP6 with external loops and long promoter staple. a, Crude and PEG-purified DNA origami objects are given in lane 1 and 2 respectively for 20HB-exL, 20HB-exLP (sc_EGFP5) and 20HB-exLP (sc_EGFP6). Respective ssDNA scaffolds are included as MW controls, while 'ladder' depicts NEB 1 kb dsDNA ladder. **b**, Representative negative stain TEM images corresponding to each of the samples. Scale bar is 100 nm.

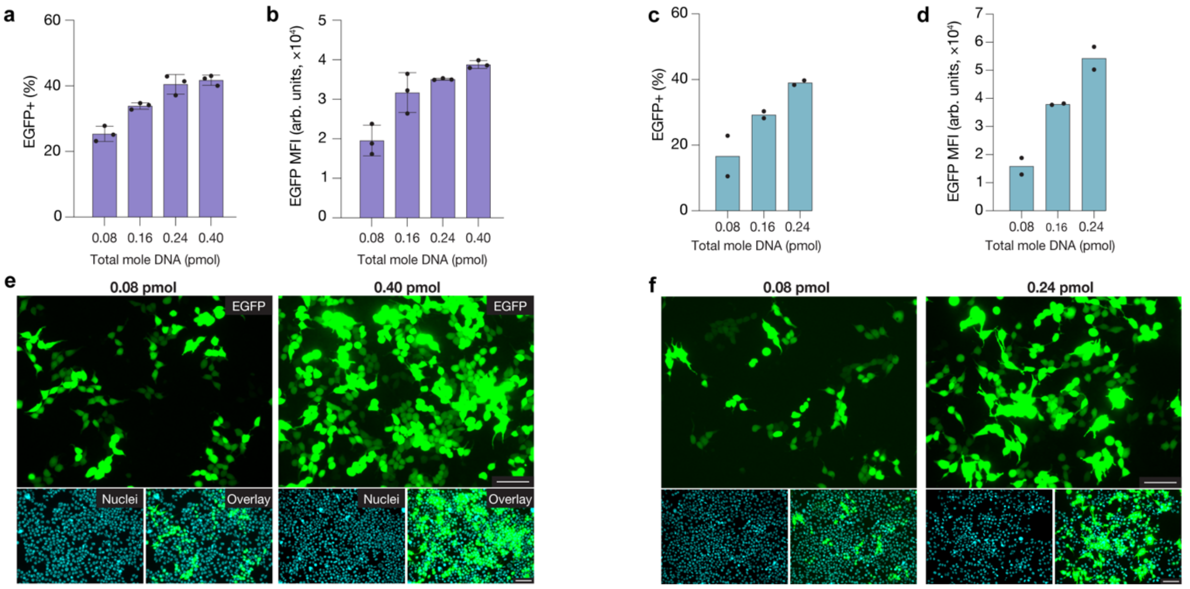

Fig. S16 | Titration of 20HB achieves high transfection efficiency. Titrating the 20HB-exLP folded from the sc_EGFP5 (**a** and **b**) and sc_EGFP6 (**c** and **d**) scaffolds increased both the transfection efficiency and MFI, respectively. Data is presented as mean ± s.d. (**a**, **b**) or mean (**c**, **d**) with individual data points overlaid. **e** and **f**, Representative epifluorescent microscopy images demonstrated the expression of EGFP by successfully transfected cells. For each of the conditions, EGFP expression (green), cell nuclei (blue) and the overlay are given. The images are representative of one of n = 3 and n = 2 biologically independent experiments for **e** and **f** respectively; similar results were observed each time. Scale bar 100 μm.

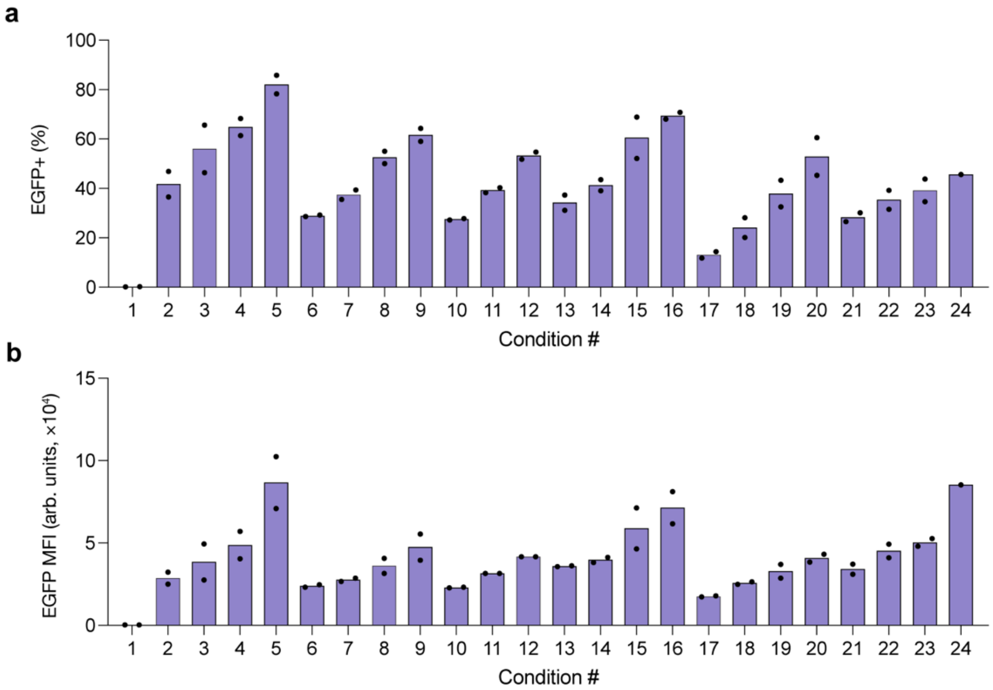

Fig. S17 I Electroporation condition screen in HEK293T cells. Electroporation conditions were screened to optimise delivery of 20HB DNA origami objects. 20HB-exLP sample from scaffold sc_EGFP5 was used in all conditions. Numbers refer to programs detailed in Table S4. Data is presented as mean with individual data points overlaid representing biologically independent experiments.

Table S4 I Electroporation conditions screened as featured in Fig S17.

Sample	Pulse voltage (V)	Pulse width (ms)	Pulse no.
**1**	-	-	-
**2**	1400	20	1
**3**	1500	20	1
**4**	1600	20	1
**5**	1700	20	1
**6**	1100	30	1
**7**	1200	30	1
**8**	1300	30	1
**9**	1400	30	1
**10**	1000	40	1
**11**	1100	40	1
**12**	1200	40	1
**13**	1100	20	2
**14**	1200	20	2
**15**	1300	20	2
**16**	1400	20	2
**17**	850	30	2
**18**	950	30	2
**19**	1050	30	2
**20**	1150	30	2
**21**	1300	10	3
**22**	1400	10	3
**23**	1500	10	3
**24**	1600	10	3

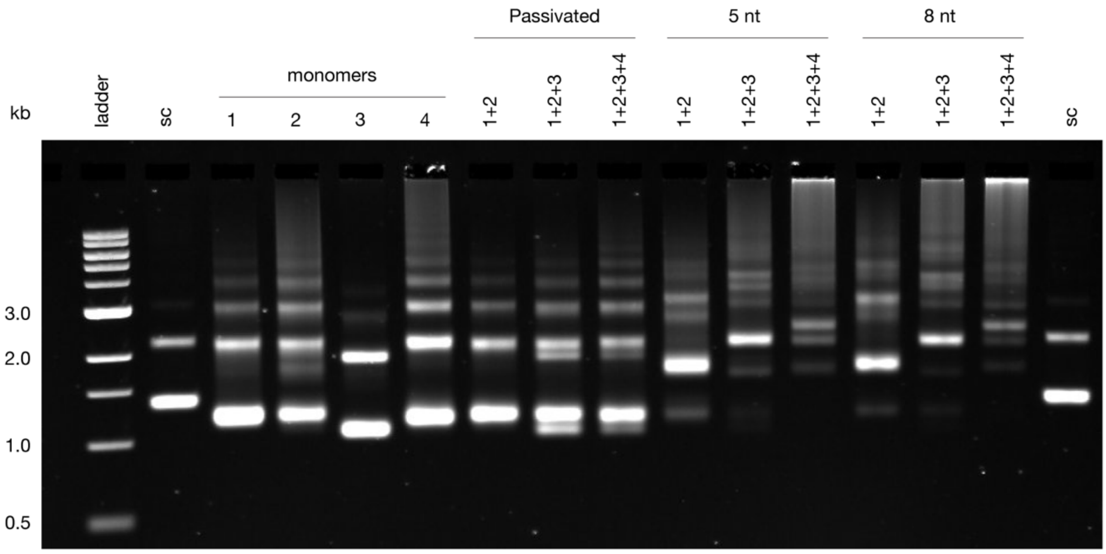

Fig. S18 | AGE of purified DNA origami objects and assembly products. Lanes labelled 1 – 4 demonstrate purified monomer samples. Lanes 1+2, 1+2+3 and 1+2+3+4 demonstrate the monomer addition to create dimer, trimer, and tetramer higher-order structures respectively. No assembly can be observed in the passivated samples. sc_EGFP5 was used as MW control, while 'ladder' depicts NEB 1 kb dsDNA ladder.

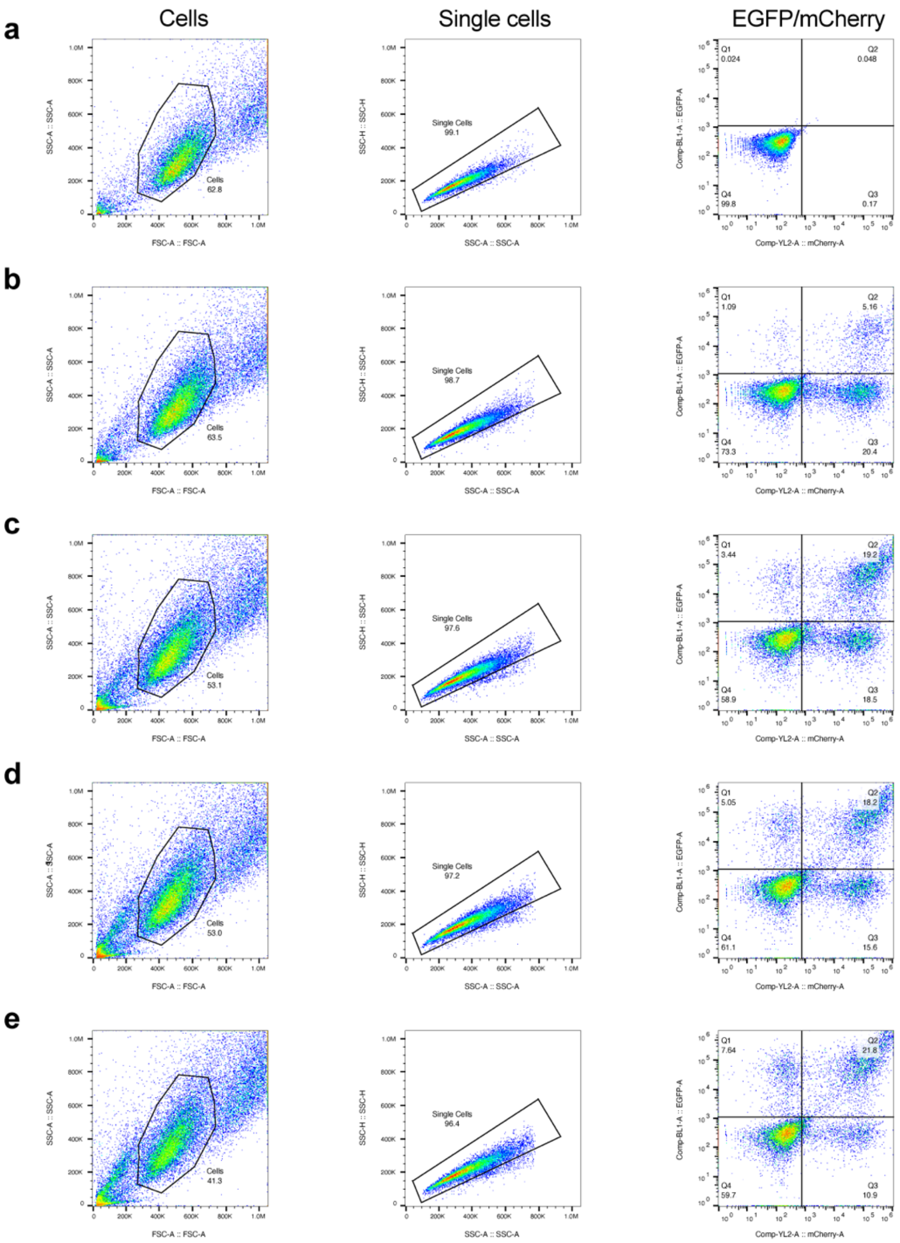

Fig. S19 l Exemplary flow cytometry gating. From left to right: cell populations were first gated on forward scatter-area (FCS-A) versus side scatter-area (SSC-A), gate ‘cells’; single cells were selected by gating SSC-A versus side scatter-height (SSC-H), gate ‘single cells’; cells were then assessed for EGFP and/or mCherry expression. In this example, cell populations were investigated for expression of both mCherry (x-axis) and EGFP (y-axis) expression, where cells can be either EGFP+/mCherry- (Q1), EGFP+/mCherry+ (Q2), EGFP-/mCherry+ (Q3) or EGFP-/mCherry- (Q4). **a** gives representative gating for untreated cells, **b** demonstrates delivery of individual passivated mCherry and EGFP origami objects at a 1:1 ratio mCherry:EGFP. Delivery of objects assembled with 8 nt overhangs at mCherry:EGFP ratios of 1:1, 1:2 and 1:3 are given in **c**–**e**, respectively.

2.2 Scaffold sequences and origami design

2.2.1 sc_EGFP1

Scaffold sequence for sc_EGFP1:
cgcgttgacattgattattgactagttattaatagtaatcaattacggggtcattagttcatagcccatatatggagttccgcgttacataacttacggt
aaatggcccgcctggctgaccgcccaacgacccccgcccattgacgtcaataatgacgtatgttcccatagtaacgccaatagggactttcca
ttgacgtcaatgggtggagtatttacggtaaactgcccacttggcagtacatcaagtgtatcatatgccaagtacgccccctattgacgtcaatga
cggtaaatggcccgcctggcattatgcccagtacatgaccttatgggactttcctacttggcagtacatctacgtattagtcatcgctattaccatgg
tgatgcggttttggcagtacatcaatgggcgtggatagcggtttgactcacggggatttccaagtctccaccccattgacgtcaatgggagtttgttt
tggcaccaaaatcaacgggactttccaaaatgtcgtaacaactccgccccattgacgcaaatgggcggtaggcgtgtacggtgggaggtcta
tataagcagagctctctggctaactagagaacccactgcttactggcttatcgaaattaatacgactcactatagggagacccaagcttggtacc
gagctcggatccactagtaacggccgccagtgtgctggaattctgcagatatccatcacactggcggccgctcgagatggtgagcaagggcg
aggagctgttcaccggggtggtgcccatcctggtcgagctggacggcgacgtaaacggccacaagttcagcgtgtccggcgagggcgagg
gcgatgccacctacggcaagctgaccctgaagttcatctgcaccaccggcaagctgcccgtgccctggcccaccctcgtgaccaccctgacc
tacggcgtgcagtgcttcagccgctaccccgaccacatgaagcagcacgacttcttcaagtccgccatgcccgaaggctacgtccaggagc
gcaccatcttcttcaaggacgacggcaactacaagacccgcgccgaggtgaagttcgagggcgacaccctggtgaaccgcatcgagctga
agggcatcgacttcaaggaggacggcaacatcctggggcacaagctggagtacaactacaacagccacaacgtctatatcatggccgaca
agcagaagaacggcatcaaggtgaacttcaagatccgccacaacatcgaggacggcagcgtgcagctcgccgaccactaccagcagaa
cacccccatcggcgacggccccgtgctgctgcccgacaaccactacctgagcacccagtccgccctgagcaaagaccccaacgagaagc
gcgatcacatggtcctgctggagttcgtgaccgccgccgggatcactctcggcatggacgagctgtacaagtaatctagagggccctattctat
agtgtcacctaaatgctagagctcgctgatcagcctcgactgtgccttctagttgccagccatctgttgtttgcccctcccccgtgccttccttgaccc
tggaaggtgccactcccactgtcctttcctaataaaatgaggaaattgcatcgcattgtctgagtaggtgtcattctattctggggggtggggtggg
gcaggacagcaagggggaggattgggaagacaatagcaggcatgctggggatgcggtgggctctatggcttctgaggcggaatgtgacatt
aagcgcggcgggtgtggtggttacgcgcagcgtgaccgctacacttgccagcgccctagcgcccgctcctttcgctttcttcccttcctttctcgcc
acgttcgccggctttccccgtcaagctctaaatcgggggctccctttagggttccgatttagtgctttacggcacctcgaccccaaaaaacttgatt
agggtgatggttcacgtagtgggccatcgccctgatagacggtttttcgccctttgacgttggagtccacgttctttaatagtggactcttgttccaaa
ctggaacaacactcaaccctatctcggtctattcttttgatttataagggattttgccgatttcggcctattggttaaaaaatgagctgatttaacaaaa
atttaacgcgaattttaacaaaatattaacgcttacaatttaggtggcacttttcggggaaatgtgcgcggaacccctatttgtttatttttctaaatac
attcaaatatgtatccgctcatgagacaataaccctgataaatgcttcaataatattgaaaaaggaagagtatgagtattcaacatttccgtgtcg
cccttattcccttttttgcggcattttgccttcctgtttttgctcacccagaaacgctggtgaaagtaaaagatgctgaagatcagttgggtgcacgag
tgggttacatcgaactggatctcaacagcggtaagatccttgagagttttcgccccgaagaacgttttccaatgatgagcacttttaaagttctgct
atgtggcgcggtattatcccgtattgacgccgggcaagagcaactcggtcgccgcatacactattctcagaatgacttggttgagtactcaccag
tcacagaaaagcatcttacggatggcatgacagtaagagaattatgcagtgctgccataaccatgagtgataacactgcggccaacttacttct
gacaacgatcggaggaccgaaggagctaaccgcttttttgcacaacatgggggatcatgtaactcgccttgatcgttgggaaccggagctga
atgaagccataccaaacgacgagcgtgacaccacgatgcctgtagcaatggcaacaacgttgcgcaaactattaactggcgaactacttact
ctagcttcccggcaacaattaatagactggatggaggcggataaagttgcaggaccacttctgcgctcggcccttccggctggctggtttattgct
gataaatctggagccggtgagcgtgggtctcgcggtatcattgcagcactggggccagatggtaagccctcccgtatcgtagttatctacacga
cggggagtcaggcaactatggatgaacgaaatagacagatcgctgagataggtgcctcactgattaagcattggtaactgtcagaccaagttt
actcatatatactttagattgatttaaaacttcatttttaatttaaaaggatctaggtgaagatcctttttgataatctcatgaccaaaatcccttaacgtg
agttttcgttccactgagcgtcagaccccgtagaaaagatcaaaggatcttcttgagatcctttttttctgcgcgtaatctgctgcttgcaaacaaaa
aaaccaccgctaccagcggtggtttgtttgccggatcaagagctaccaactctttttccgaaggtaactggcttcagcagagcgcagataccaa
atactgttcttctagtgtagccgtagttaggccaccacttcaagaactctgtagcaccgcctacatacctcgctctgctaatcctgttaccagtggct
gctgccagtggcgataagtcgtgtcttaccgggttggactcaagacgatagttaccggataaggcgcagcggtcgggctgaacggggggttc
gtgcacacagcccagcttggagcgaacgacctacaccgaactgagatacctacagcgtgagctatgagaaagcgccacgcttcccgaag
ggagaaaggcggacaggtatccggtaagcggcagggtcggaacaggagagcgcacgagggagcttccagggggaaacgcctggtatc
tttatagtcctgtcgggtttcgccacctctgacttgagcgtcgatttttgtgatgctcgtcaggggggcggagcctatggaaaaacgccagcaacg
cggcctttttacggttcctggccttttgctggccttttgctcactcattgatata

Corresponding DNA origami designs and staple lists:

2.2.1.1 sc_EGFP1 20HB-ext and 20HB-ext-W

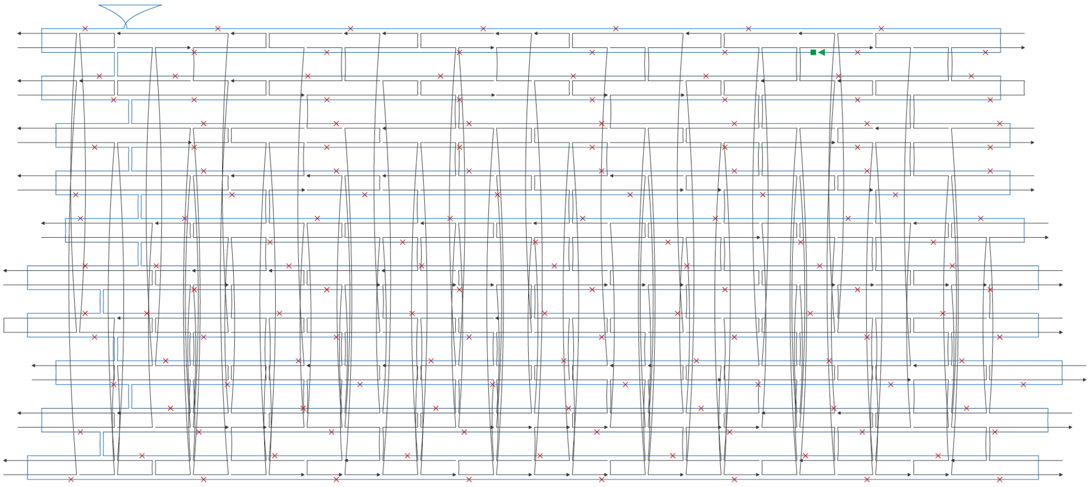

Fig. S20 I Scaffold routing and staple design for sc_EGFP1 20HB-ext and 20HB-ext-W. Scaffold routing is shown in blue, staples are given in black, breakpoint for addition of sequence is marked by a green square (5') and arrow (3'). Design was prepared using caDNAno v0.1.

Table S5 I Individual staple sequences for sc_EGFP1 20HB-ext.

Name	Sequence 5' - 3'
core_1	GGAAGCTCCCTCGTGCGGCGGGCCGGGCGACAACCACCG
core_2	GGACTATAAAGATACGGAACTCCGGAAGGCTGCAAGCA
core_3	TCCGCCCCCCTGACGAATAATCGTGCACCCGGGGTCT
core_4	GCGTTTTTAATTCGCGTTAAATTTTATAAATAGCAGAA
core_5	ATTTACCAAGTCCCTATTGGCGTTGACGTC
core_6	ATTGATTACACTTGATGTACTGCGATGTAC
core_7	AAGGCCAGTAATGCCAGGCGGGCCCAAGTAGGGGTCTCCC
core_8	CGTCATTATTGACGTGTATTGATTTTGGTGCCAAAACAAACTCCCATTACTATG
core_9	TCAATGGAGTAAGTTATGTAACGCCAGGCGTT
core_10	CGTAAATACTCCACCCCCGTGAGACCGTACACGCCTACC
core_11	CAGTTTACGGCTATGAACTAATGAGTGGCGAA
core_12	ATATGATACTATTAATAACTAGTCAGCATCAC
core_13	GGGGGCGTATAGCGAATAAGCCAGTAAGCAGAGCGGCCGTAAACTTCCGCCTCC
core_14	GCTATCCACGCCCATTCAAGTGGGAAAAAAAG
core_15	CCATGGTAACTTGGCTTTTCTACAACTGATTCTTGCCC
core_16	TGGGGCGGAGTTGTTACACGCTGACCGTCGTGTACCGCGA
core_17	GCGTCAAAATGGGGTGGAGACTTGGAAATCCATTGACGTTTTTGTTAAAATGCC
core_18	GCCAGAGAGCTCTGCTGAACAGCATCAGTGGCCGAGCG
core_19	AGTCGTATGTGGAACGGTCATTGACGCGTATATCAATGA
core_20	TATAGTGAATTCCAGAAGTTTTGGAAGCTTCGTCGTT
core_21	CGCCGGACGACATTTTGATCCGGAACATATTGGGCGGTCAGCCAGCTCTCCT
core_22	CCGTTTACGTCGCCGGCCCATTTCGGTGGTT
core_23	CCTTGCTCACCATCTCGTGGGTTCCTTTGATC
core_24	CGAGGGTTGGGCACCACCCCGGTTATATAGACGCGCAG
core_25	AGCGGCTGAACTTTATGGTCTGACGAGTTGCCTTCAGCA
core_26	TGTGGCTTCACCTTGATGCCGTTGGGGCAAAAGTGTTATAATCGGA
core_27	TGTACTCCAGCTTGTAGCTTGCCGCTCCAGATTCATCCA
core_28	GCCCCAGGCCGTCCTCGATGTTGTGGCACAGTCTCCGATCAGAGCTTG
core_29	ATGTTGCCGTCCTCCTTGAAGTCGGTGGTCACCGGAAGG
core_30	GGGTGTTTCAGCTCGATGCGGTTCACCAGG
core_31	CAGCACGGGGCCGTCTAGATTAACGATCAAGGGCGCTA
core_32	TCGGGCAGCTCGAACTTCACCTCGGCGCGGGAAGAAGTCTGTTGCCGAAATCAA
core_33	ACTGGGTGCTCAGGTAGTGATCTGGTATGACGCTGCG
core_34	GGCTGGCAACTAGAAGGCGGATCTCACCGGGTGGTG
core_35	ATGCCGAGAGTGGTTGCTATTAATGTGCTGCTTGATGGATATCTGCAG

core_36	CCGGCGGCGCCTGCTATTGTCTTCCCAATCCTAGCGGTCGCTTCATT
core_37	GGTCAAGAAGCACTACACTCATGGTTACTA
core_38	ATTTTATTAGGAAAGGCCGATTTGTTGTCAGCCGTAAGATAGTTGCCCTGGTAG
core_39	CGATGCAGATCAGCGAGCTCTAGGCGAGCTGCAGCCAG
core_40	CCAGAATAATAGAATAGGGCCCTCGCCGATGGGGTCCTGCAAGCACTG
core_41	ACCACACCCCCAGCATGGTCACGAACTCCAGTCTTTGCT
core_42	TCACCCTAATCAAGTTTTATTGAACTCATACGGGGGTCG
core_43	CTATCAGGGCGATGGCATGAGCGGGGAATAA
core_44	AACGTCAAAAAATAACAAAAACAATATATG
core_45	TTTGGAACAAGAGTCTTCCCCGATCTTTTAC
core_46	AATCGGCAAAATCCCTTTGTTAATCTTACCG
core_47	TTTGAATGTATTTAGAAGGGCGAAGGTGAGTATTAGCTCCAATAAACCACGCTG
core_48	GGGTTCCGCGCACATCACTATTAAGTGTATGACATGATCCAGAAGT
core_49	AAAGTGCCAAAAATCGACGCTCAAGTCAGAGCCCCGTATTTCACCAGATCTCA
core_50	ACCTAAATTGTAAGCTTGAGTGTGGCGTCACAGCTCCGATCCAGT
core_51	GCGTTTCCTGAGAATAAGAACGGAAGGGAAGAAAGCG
core_52	CTGTGACTAAACCGTACGGGGAAAGCCGGCG
core_53	ATACGGGATCTAAAGTGACGCTCATAATTTCGTGACTAATACGTAGATGTACTGCATTTACC
core_54	CCCCATGTGACACTGAATGACACCTACTCAGACAATGAACGTGG
core_55	GTTCCCACTTGTACAGCTGTCCTGCCCCACCCCACCCCAAAGGAGC
core_56	CATCGTGGCTTTAAAATTTTAAAGGATTTTG
core_57	TAGATAACTAATTCTGTTGAATAGCATTTAGTTCCGACCCTGCCGCTTACCCT
core_58	TGACTCCACTTGTGGCAGATGAACTTCAGGG
core_59	GATCTGTCGACCAGGAGGGCCAGGGCACGGGC
core_60	ATATATGAGCCAGTGTCATGTGGTCGGGGTGTGTCGCC
core_61	TTAAAAATGCACACTGGGCATGGCGGACTTGTCTTGTAG
core_62	GGCAAACAACGGAAATCTTACTGTGTGAACCAACCCTAAAGGGAGCCCACAGTGG
core_63	CAATGGGCTCTTCCTTAAAGAGTTGGGAGGGCACTCGCCCTCGCCCT
core_64	TCCAGCTCTATTTCGTTTATCAGCTTCGGTCCGAGGCTATTTCCTC
core_65	AGTGGTCGCATTTAGGTTGTGCAACGAGAAAGTGGACTCC
core_66	TGTTCCAGGGGCGCTGGCAAGTGTCCCCCTTGCTCGTCC
core_67	TAGACCGAGATAGGGGTTAATATACCCACTCAATGTCAACGTCAATA
core_68	TCAGGGTTATTGTCTCCCACTACCATGCCATAAGTAAGGACCCACGCTTGAAGTGTTGTAGT
core_69	TGCTTTTGCAAAAAAGATACATATCCCCCT
core_70	TAATACCGCGATGTATTTGTTAACCATAGGC
core_71	TTGGCCGCCAACAGATGAGTGGCACCTTCCAG
core_72	AAAAGCGGCTCAACCATCTCAGCGCAGATTACCTCCCTCAAACC
core_73	GGCGAGTTCGGCGACCAGTTACCAGAAGATCTCTAGTTATGCCAAAACCGCATCA
core_74	AGGCACCTAAGTCATTTGGGTGAGACAAATAGACCCGACA
core_75	AATGCTTATCCTCGCCACGCCGTAGGTCAGGATGCCCTCTGCTGGT
core_76	AAAACTCATCCAGTTCGCCACATCAAAAGAACGTAACC
polyT_1	TTTTTGGAACCGTAAAAAGGCCGCGTTGCTGGTGAGCAACTGTTGAGACGTTAAG
polyT_2	TTTTTGCGTGGCGCTTTCTCATTCTCCCGGCGGTGTTTCAATATTTTGGG
polyT_3	TTTTTGTATCTCAGTTCGGTTAGCTCA
polyT_4	CAAGCTTGAAAGTCCCATAAGGTCATTTTTTTTTTTGTACTGGGCACAAAAGGCCATTTTT
polyT_5	TTTTTCGAGCTCGGTACGTCATGAGATTATCAAAATTTTT
polyT_6	TTTTTTTTTTGGGCTGTGTGCTTATCTGCGCTCTGCTGATTTTT
polyT_7	TTTTTGCTCCTGGACGTCAGTTAATCTAGATCC
polyT_8	TTTTTTTTTTGACCGCTGCGCCTTATCGGCCATGATATAGACGTTCAGCTTGCAATGA
polyT_9	TTTTTCGCTTCTCGTTGGGGCAGGACCACTACAGGTTAATGTC
polyT_10	TTTTTCTTGAGTCCAACCCGGGGCACGGGGGACTTCTGC
polyT_11	TTTTTTCAGAAGCCATAGAGCCCACCGCATCCGCCGCGC
polyT_12	TTTTTGCAGCAGCCACTGGTAGAGGTGCCGTAGAATAAGACACGACTTATCGCCACTGTTTTT
polyT_13	TTTTTCAATAGGCCGAACATTCCGCCTTTTT
polyT_14	TTTTTTTCGGGGCGAAAACTCCATTGGAAAACGTTCTTTTT
polyT_15	TTTTTGAAGTGGTGGCCGTCACAGGATTAGCAGAGTTTTTTTTTTCGAGGTATGTATTCGGGAATTTTT
polyT_16	TAATGGCAGCACTGCATACGATACGGTAGCTCTTGGAAAGTCCCGGGTCGTTCGCTCCAAGTTTTT
polyT_17	GTGCTCATTCAAGGAATCAGCTCATTTTTTAACTTTTT
polyT_18	TGTCACGCAGAGTAAGCAGGGCGGTTGCCGTCGTCCTTGAAGAAGATGGTGCTTTTT
polyT_19	TTTTTGTTGTTGCCATTGTGTGATCGTTTTT
polyT_20	TTTTTACAGTATTTGGTACCCGGCTACACTAGAAGATTTTT
polyT_21	ATCTGGCCTTGTCCGGTAACTATCGTTTTTT
polyT_22	CCAGTGCTGCCGTAGGTGGCAGAACCCCCCGTTCAGCTTTTT
polyT_23	TAGTTCGCAGCCTTCGGCGGCCGTTACTAGTGGATCTTTTT
polyT_24	TTTTTAGGATCTTCACAGTTTGCGCAACTTTTT
polyT_25	TTTTTAGCCAGTTACCGCTGTAGTTTTT
polyT_26	CTTCGGAACTACAGAGTTCTTTTTTT

Table S6 | Individual staple sequences for sc_EGFP1 20HB-ext-W.

Name	Sequence 5' - 3'
core_1	tGGAAGCTCCCTCGTGCttGGCGGGCCttGGGCGACttAACCACCGt
core_2	tGGACTATAAAGATACttGGAACTCCttGGAAGGCttTGCAAGCAt
core_3	tTCCGCCCCCCTGACGttAATAATCttGTGCACCCttGGGGTCTt
core_4	tGCGTTTTTttAATTCGCGTTAAATTttTTATAAATttAGCAGAAt
core_5	tATTTACCttAAGTCCCTATTGGCGttTTGACGTCt
core_6	tATTGATTAttCACTTGATGTACTGCttGATGTACt
core_7	tAAGGCCAGttTAATGCCAGGCGGGCCttCAAGTAGGttGGTCTCCCt
core_8	tCGTCATTATTGACGTttGTAttTTGATTTTGGTGCCAAAACAAACTCCCAttTTACTATGt
core_9	tTCAATGGAttGTAAGTTATGTAACGCttCAGGCGTTt
core_10	tCGTAAATACTCCACCttCCCGTGAGttACCGTACACGCCTACCt
core_11	tCAGTTTACttGGCTATGAACTAATGAttGTGGCGAAt
core_12	tATATGATAttCTATTAATAACTAGTCttAGCATCACt
core_13	tGGGGGCGTttATAGCGAttATAAGCCAGTAAGCAttGAGCGGCCttGTAAACTTttCCGCCTCCt
core_14	tGCTATCCACGCCCATTttCAAGTGGGttAAAAAAAGt
core_15	tCCATGGTAttACTTGGCttTTTTCTACttAACTGATttTCTTGCCCt
core_16	tTGGGGCGGAGTTGTTAttCACGCTGAttCCGTCGTGttTACCGCGAt
core_17	tGCGTCAAttAATGGGGTGGAGACTTGGAAATCttCATTGACGttTTTTTGTTttAAAATGCCt
core_18	tGCCAGAGAGCTCTGCttTGAACAGCttATCAGTGttGCCGAGCGt
core_19	tAGTCGTATttGTGGAACGttGTCATTGAttCGCGttTATATCAATGAt
core_20	tTATAGTGttAATTCCAttGAAGTTTTttGGAAGCTttTCGTCGTTt
core_21	tCGCCGGAttCGACATTttTTGATCCttGGAACATAttTTGGGCGGTCAGCCAttGCTCTCCTt
core_22	tCCGTTTACGTCGCCGttGCCCATTTttCGGTGGTTt
core_23	tCCTTGCTCACCATCTCttGTGGGTTCttCTTTGATCt
core_24	tCGAGGGTttTGGGCACCACCCCGGttTTATATAGttACGCGCAGt
core_25	tAGCGGCTGttAACTTTATttGGTCTGAttCGAGTTGCttCTTCAGCAt
core_26	tTGTGGCTttTCACCTTGATGCCGTTttGGGGCAAAttAGTGTTATttAATCGGAt
core_27	tTGTACTCCAGCTTGTttAGCTTGCCttGCTCCAGAttTTCATCCAt
core_28	tGCCCCAGGttCCGTCCTCGATGTTGTttGGCACAGTttCTCCGATCttAGAGCTTGt
core_29	tATGTTGCCGTCCTCCTTGAAGTCttGGTGGTCAttCCGGAAGGt
core_30	tGGGTGTTttTCAGCTCGATGCGGTTCACCAGGt
core_31	tCAGCACGGGGCCGTCttTAGATTAttACGATCAAttGGGCGCTAt
core_32	tTCGGGCAGttCTCGAACTTCACCTCGGCGCGGGttAAGAAGTCttTGTTGCCGttAAATCAAt
core_33	tACTGGGTGCTCAGGTttAGTGATCttTGGTATGttACGCTGCGt
core_34	tGGCTGGCAACTAGAAttGGCGGATttCTCACCGttGGTGGTGt
core_35	tATGCCGAGttAGTGGTTGttCTATTAATttGTGCTGCTttTGATGGATATCTGCAGt
core_36	tCCGGCGGCttGCCTGCTATTGTCTTCCCAATCCttTAGCGGTCttGCTTCATTt
core_37	tGGTCAAGttAAGCACTAttCACTCATGGTTAttCTAt
core_38	tATTTTATTAGGAAAGGttCCGATTTttGTTGTCAGttCCGTAAGAttTAGTTGCCttCTGGTAGt
core_39	tCGATGCAttGATCAGCGAGCTCTAGttGCGAGCTGttCAGCCAGt
core_40	tCCAGAATAttATAGAATAGGGCCCTCttGCCGATGGttGGTCCTGCttAAGCACTGt
core_41	tACCACACCttCCCAGCATttGGTCACGAACTCCAGttTCTTTGCTt
core_42	tTCACCCTAATCAAGTTttTTATTGAAttCTCATACttGGGGGTCGt
core_43	tCTATCAGGGCGATGGCttATGAGCGttGGGAATAAt
core_44	tAACGTCAAttAAAATAAttCAAAAACAttATATATGt
core_45	tTTTGGAACAAGAGTCttTTCCCCGAttTCTTTTACt
core_46	tAATCGGCAAAATCCCttTTTGTTAAttTCTTACCGt
core_47	tTTTGAATGTATTTAGAttAGGGCGAAttGGTGAGTAttTTAGCTCttCAATAAACttCACGCTGt
core_48	tGGGTTCCGCGCACATttCACTATTAttAGTGTATGttACATGATCttCAGAAGTt
core_49	tAAAGTGCCttAAAAATCGACGCTCAAGTCAGAGttCCCCGTAttTTTCACCAttGATCTCAt
core_50	tACCTAAATTGTAAGCttTTGAGTGTttGGCGTCAttCAGCTCCGttATCCAGTt
core_51	tGCGTTTCttCTGAGAATttAAGAACGttGAAGGGAAGAAAGCGt
core_52	tCTGTGACTttAAACCGTttACGGGGAAAGCCGGCGt
core_53	tATACGGGAttTCTAAAGTttGACGCTCAttTAATTTCGttTGACTAATACGTAGATGTACTGCttATTTACCt
core_54	tCCCCATGttTGACACTttGAATGACACCTACTCAGACAATGttAACGTGGt
core_55	tGTTCCCAttCTTGTACAttGCTGTCCTGCCCCACCCCACCCCttAAAGGAGCt
core_56	tCATCGTGGttCTTTAAAAttTTTTAAAttGGATTTTGt
core_57	tTAGATAACttTAATTCTttGTTGAATAttGCATTTAttGTTCCGACCCTGCCGCTTACCttCTt
core_58	tTGACTCCttACTTGTGGttCAGATGAACTTCAGGGt
core_59	tGATCTGTCttGACCAGGAttGGGCCAGGGCACGGGCt
core_60	tATATATGAttGCCAGTGttTCATGTGGTCGGGGTttGTGTCGCCt
core_61	tTTAAAAATttGCACACTGttGGCATGGCGGACTTGttTCTTGTAGt
core_62	tGGCAAACAttACGGAAATttCTTACTGTttGTGAACCAttACCCTAAAGGGAGCCCttACAGTGGt
core_63	tCAATGGGCttTCTTCCTTttAAAGAGTTttGGGAGGGttCACttTCGCCCTCGCCCTt

core_64	tTCCAGCTCttTATTTCGttTTTATCAGttCTTCGGTCttCGAGGCTttATTTCCTCt
core_65	tAGTGGTCGttCATTTAGGttTTGTGCAAttCGAGAAAGttTGGACTCCt
core_66	tTGTTCCAGttGGGCGCTGGCAAGTGttTCCCCCTTttGCTCGTCCt
core_67	tTAGACCGAGATAGGGttGTTAATATttACCCACTCttAATGTCAAttCGTCAATAt
core_68	tTCAGGGTTATTGTCTCttCCACTACttCATGCCATttAAGTAAGttGACCCACGttCTTGAAGTttGTTGTAGTt
core_69	tTGCTTTTttGCAAAAAAttGATACATAttTCCCCCTt
core_70	tTAATACCGttCGATGTAttTTTGTTAAttCCATAGGCt
core_71	tTTGGCCGCttCAACAGATttGAGTGGCACCTTCCAGt
core_72	tAAAAGCGGttCTCAACCttATCTCAGCttGCAGATTttACCTCCCttTCAAACCt
core_73	tGGCGAGTTttCGGCGACttCAGTTACCttAGAAGATCttTCTAGTTAttTGCCAAAACCGCATCAt
core_74	tAGGCACCTttAAGTCATTttTGGGTGAGttACAAATAGttACCCGACAt
core_75	tAATGCTTAttTCCTCGCttCACGCCGTAGGTCAGttGATGCCCTttCTGCTGGTt
core_76	tAAAACTCttATCCAGTTttCGCCACATttCAAAAGAAttCGTAACCt
polyT_1	tttttGGAACCGTAAAAAGGCCGCGTTGCTGttGTGAGCAAttCTGTTGAGttACGTTAAGt
polyT_2	tttttGCGTGGCGCTTTCTCAttTTCTCCCttGGCGGTGttTTTCAATAttTTTTGGGt
polyT_3	tttttGTATCTCAGTTCGGTttTAGCTCAt
polyT_4	tCAAGCTTGttAAAGTCCCATAAGGTCATttttttttttGTACTGGGCAttCAAAAGGCCAttttt
polyT_5	tttttCGAGCTCGGTACttGTCATGAGATTATCAAAAttttt
polyT_6	ttttttttCTGGGCTGTGTGttCTTttATCTGCGCTCTGCTGAttttt
polyT_7	tttttGCTCCTGGACGTttCAGTTAATttCTAGATCCt
polyT_8	ttttttttCCGACCGCTGCGCCTTAttTCGGCCATGATATAGACGTttTCAGCTTttGCAATGAt
polyT_9	tttttCGCTTCTCGTTGGGGttCAGGACCAttCTACAGGttTTAATGTCt
polyT_10	tttttCTTGAGTCCAACCCGGttGGCACGGGGGAttCTTCTGCt
polyT_11	tttttTCAGAAGCCATAGAGCCCACCGCATCttCGCCGCGCt
polyT_12	tttttGCAGCAGCCACTGGTAttGAGGTGCCGTAttGAAttTAAGACACGACTTATCGCCACTGttttt
polyT_13	tttttCAATAGGCCGAttACATTCCGCCttttt
polyT_14	tttttTTCGGGGCGAAAACTCttCATTGGAAAACGTTCttttt
polyT_15	tttttGAAGTGGTGGCCttGTCttACAGGATTAGCAGAGttttttttttCGAGGTATGTAttTTCGGGAAttttt
polyT_16	tTAAttTGGCAGCACTGCAttTACGATACttGGTAGCTCttTTGGAAAGTCCCGttGGTCGTTCGCTCCAAGttttt
polyT_17	tGTGCTCATttTCAAGGAttATCAGCTCATTTTTTAACttttt
polyT_18	tTGTCACGCttAGAGTAAGttCAGGGCGGttTTGCCGTCGTCCTTGAAGAAGATGGTGCttttt
polyT_19	tttttGTTGTTGCCATTGttTGTGATCGttttt
polyT_20	tttttACAGTATTTGGTttACCttCGGCTACACTAGAAGAttttt
polyT_21	tATCTGGCCttTTGttTCCGGTAACTATCGTttttt
polyT_22	tCCAGTGCTttGCCGTAGGTGGCAttGAACCCCCCGTTCAGCttttt
polyT_23	tTAGTTCGCttAGCCTTCGttGCGGCCGTTACTAGTGGATCttttt
polyT_24	tttttAGGATCTTCACttAGTTTGCGCAACttttt
polyT_25	tttttAGCCAGTTACttCGCTGTAGttttt
polyT_26	tCTTCGGAAttCTACAGAGTTCTTttttt

2.2.1.2 sc_EGFP1 20HB-int and 20HB-int-W

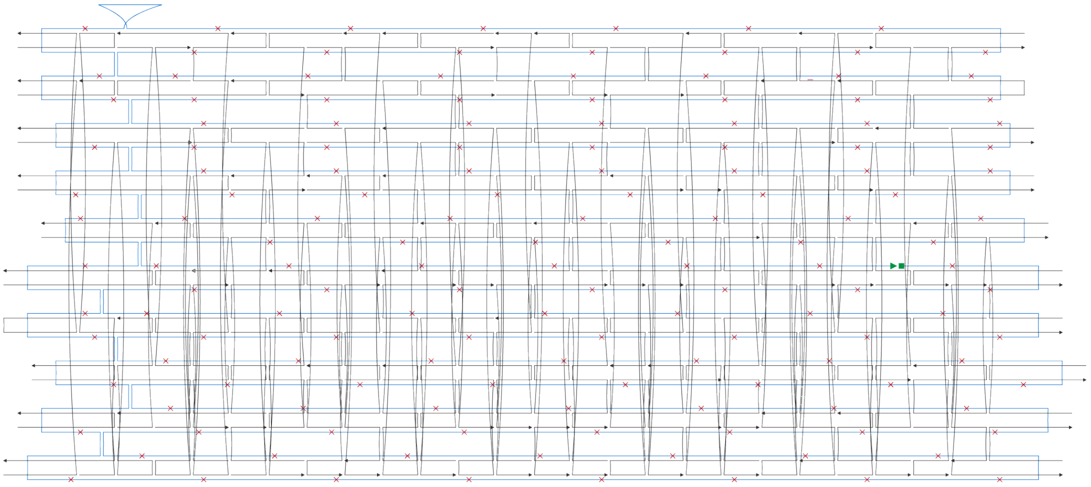

Fig. S21 l Scaffold routing and staple design for sc_EGFP1 20HB-int and 20HB-it-W. Scaffold routing is shown in blue, staples are given in black, breakpoint for addition of sequence is marked by a green square (5') and arrow (3'). Design was prepared using caDNAno v0.1.

Table S7 | Individual staple sequences for sc_EGFP1 20HB-int.

Name	Sequence 5' - 3'
core_1	GGCAAAATCCCTTATAGAGCAAAATTAATTTCCCAATCC
core_2	AGCTCATTTTTTAACAAAGGGAACCAAGCTCCCACCCC
core_3	GTGCCACCTAAATTGGAAGCATTGATGGATAGGAAAG
core_4	CACATTTCACTTGGAAATCCCCGATGGTAATACTTGTG
core_5	ACAGGAAGAGATCCAGTTCGATTCGGTCCT
core_6	TACTCTTCAAAGTGCTCATCATTGCACTGC
core_7	ATATTTGAGACCGAGTTGCTCTTGACTCAACCATTAATTG
core_8	CCCAACTGATCTTCATCAATGTTGTGCAAAAAAGCGGTTAGCTCCTGTAACCCA
core_9	CCGCTGTTGGCAAAATGCCGCAAACAATAGGC
core_10	GGCGAAAACTCTCAAGGCCGCAGCTCGTCGTTTGGTATG
core_11	TTCTTCGGGACACGGAAATGTTGAAAATTTTT
core_12	GAACTTTACTTTTTCAATATTATTTAAGCGTT
core_13	CCGCGCCACCGTAAGGTTCGCCAGTTAATACTCCAGATATAGAATAGTTCACCT
core_14	ACTCATGGTTATGGCAGGAAAACGACCTACTC
core_15	CATGCCATCATAGCAATTTTATTATCTGCACGTAGGTG
core_16	GGTTCCCAACGATCAACCTGACTCGCGCTTCTCAGCAGCA
core_17	CAGCTCCCCGATCGTTGTCAGAAGTAAGTTGGATCTTATGCCCCACTGGGTCTC
core_18	TGCCATTGCTACAGGGCAATGATGCTCGTCTGCCGTCC
core_19	GAAGCTAGGAGTGGCATCAATACGGTTATTGTCTCATGA
core_20	TTGCCGGCGGAAGGGATCAGCGGCCATGATGCCCTTC
core_21	ATAGTTGGGCGAGTGCCTGCTCTCGTGCACCAGCGTTTCTGGGTAATCAAAA
core_22	GTAGATAACTACGATGCTTCATTTGCTGTCC
core_23	GACCCACGCTCACCGGGTTTGCGCATTTCCTC
core_24	AGTATATTCTGGCCCCAGTGCTCATCGTGGAGAATGAC
core_25	ATCCTTTTATCTTGAAGGGCCCTCAGCTTGCGAATTCCA
core_26	CTGAAGCAACTACGGCTACACTACCCCCTGGAAGAAGTCGACGTCA
core_27	TTCGGAAAAAGAGTTTGACAGTTTGGGGGTGAACTCCAG
core_28	GGTAGCTCCGGTGCTACAGAGTTCTTCCGACCCTGGACGTCTATGGGA
core_29	TTGATCCGGCAAACAAACCACCGCAATCTAACTGCACGC
core_30	GCCACTGGGTGGTTTTTTTGTTTGCAAGCA
core_31	ACGACTTATCGCCACTCTCATATCGAACTTGGGCCATT
core_32	GGTAAGACCGCGCAGAAAAAAAGGATCTCAACATGAGATGCTTGTCGAGCTCTA
core_33	AACTATCGTCTTGAGCGGTGTAAGCTCGAACTCCATA
core_34	TCGTGCGCTCTCCTGTTGAAGTTCGCCGAACCAATG
core_35	TCTCAGTTTCCAACCCGTTCTTCTTATCAAAACAATAAACCAGCCAGC
core_36	GGTCGTTCCAATGAGTGAGCAAAAGGCCAGCAACGCGGATGCGGTTC
core_37	CTATAAACACCCATTGTGCTGCTTCATGCT
core_38	AATCGACGCTCAAGTCGGCGTTAAGCCTTCGGGTCAGGGCAGGACCATCCCCCT
core_39	GACGAGCTTACCGGATACCTGTCAGCGAGGTCGGCGAG
core_40	GCTGGCGTGGGAAGCGTGGCGCTTTGGCAGCATGTGGCGGAAATTAAA
core_41	TATGAACTGCGTATATGCTCCAAGCTGGGCTCTGCGCCT
core_42	GATGTACTGCCAAGTGGAGCTCTGAGTGGGTTACTTTCA
core_43	ATAGGGGGCGTACTTGACGCCTAGAGTCGTA
core_44	GCCATTTAGAGTTGTGCTCGGTATAAGGGC
core_45	GTCCCATAAGGTCATTGATTTTGGCACACTG
core_46	CAAAACCGCATCACCTGAGTCAACTCCTCGC
core_47	TTGCGTCAATGGGGCGCCGTCATTGCCAGGGCGAAGATGGTAGTGGTATGTAGG
core_48	TTTGGAAAGTCCCGTGTACTGGGGATGAACTCGCGGGTCTCGATGT
core_49	GTGCCAAAAATATTTTGTTAAAATTCGCGTTATACTCAGCGGCCGTAGACAAT
core_50	ACAAACTCCCATTGAACTGCCAAGCATCGCACCAGGGTTGATGCC
core_51	TACTAGTGTGGTGCACATAATGGGTCGTTGGGCGGTC
core_52	GAGGGTGGGACGTCAACATACGTCATTATTG
core_53	CCTCGCCCGCATTTAGGACAGTGGAGTAAGTAATGCTTTTCTGTGACTGGTGAGTCCCGGCG
core_54	TTGTAGTTCCCTTCTTTTCCATAGGCTCCGCCCCCCTACGTCAA
core_55	GTCGCCCGCTCACGCAGGAACCGTAAAAAGGCCGCGTTAGCCAGGC
core_56	TCCTCCTTGCCGTTTAGGCACAGGAAGGCAC
core_57	CGTTGGGGCGGCTGACAGTAAGCCTTATATGAATAGACCGAGATAGGGTTGTT
core_58	TGTGATCCCCGTCGTCTTAATCAGTGAGGCA
core_59	CCGGCGGCGCTTACCAATGAGTAAACTTGGTC
core_60	GTGACACTTTATCAGAGGATCTTCACCTAGGCAGATTA
core_61	TCGAGGCTGCCGAGCGTTAAGGGATTTTGGTGAAGATCC
core_62	ATTGTCTTCGATAAGCAGCACTGCATACACTTATGGAAAGTCCCTATTAGAGGTG
core_63	GCATCTTTTCTCTAGTACCGCATCCAGGGCGCCCATTTCGTTCATCC
core_64	ACGGGAGGGGTCACGTTCTGCTGGTGCGCTCCTGCCGCATCACAAA
core_65	ATTAGCAGCGCCTTTCTGCCGTCGTGGGCGGGCCAGGCGG

core_66	GTAGGAAATACCGTAAGTTATGTAAAAGGCCTGTAGGTA
core_67	CTAATACGTAGATGTCGTCAATGCGCCAGTGTTATCAGGGGATAATA
core_68	AGACCTCCCACCGTACGCATATGACGCCGTAGGCATGGCGGGGCCGGGTGGCCTCAGTTACC
core_69	TGGTCACCCTATAGTCCGCCCATCGAAATC
core_70	TCGCCGGAGAGCGGCGGGTGGAGCCCGAAAA
core_71	CGGACTTGAAGCTCCCGCGAAACCCGACAGGA
core_72	TCCTTGAAACGGGCAGAGTGATCCCAGAATTGTCACGTGTTATC
core_73	CACCTCGGTCAGGGTCTAGATTAGCGATGCAAACGTTGTATAATTCTCTTACTGT
core_74	CATGCCGAGCTTGCCGGGATCCGATACGACATGTTAAATC
core_75	CTTGTACAACCGCGAAATGAAGTTTTAAATCTGGTAGCGTAACAGG
core_76	CCTTCCAACCATCTCCACGCTGAAGCGATGATATGGGC
polyT_1	TTTTTAAAAATAAACAAATAGGGGTTCCGCGGCGGATACCCTTGCTCGGGTCAAG
polyT_2	TTTTTTATTAAAGAACGTGGAGGAACAAGCGGGCGTAGCCAGAGGCAGTT
polyT_3	TTTTTGCGAAAAACCGTCTACTCCAAC
polyT_4	TCCAGTCTAAGTCATTCTGAGAATAGTTTTTTTTTTTGTATGCGGCATGTATTTAGTTTTT
polyT_5	TTTTTTATCCGCCTCCAGGGGGAGGGGCAAACAACTTTTT
polyT_6	TTTTTCGTGAACCATCAGACAATGTCACATTCCGCCTTTTT
polyT_7	TTTTTCAGTGGAACGAAGTTGTAGTAACTAGAA
polyT_8	TTTTTGGTCGAGGTGCCGTAAAATTTGGTATCTGCGCTCTGCCTATCTTGTCGGG
polyT_9	TTTTTCGTTCAGCCCGACCGGTGTGCACGTTGCCGCGTAATTG
polyT_10	TTTTTCCTAAAGGGAGCCCCCACCAGGCGTTTGAAGAAC
polyT_11	TTTTTATAACTAGTCAATAATCAATGTCAACAATGACCC
polyT_12	TTTTTCCGGCGAACGTGGCGACGTAAATACTCGATGATTTAGAGCTTGACGGGGAAAGTTTTT
polyT_13	TTTTTTGATGTACTGCATTACTATTATTTTT
polyT_14	TTTTTATGGGCACCACCCCGGTCCAGCTCGACCAGGTTTTT
polyT_15	TTTTTAAGTGTAGCGGTTACGAAAGGAAGGGAAGATTTTTTTTTTAAGCGAAAGGAGAGTCCACTTTTT
polyT_16	CACGTGGTCGGGGTAGTCTTTGCTCCCAGCATTACATGATCCCCCGGGCGATGGCCCACTATTTTT
polyT_17	CGTCGCCGTGAACAGACCGCTATCCACGCCCATTTTTT
polyT_18	GAAGTCGATATAGACGTATCCGGTTTTGATCTTTTCTACGGGGTCTGACGCTTTTTT
polyT_19	TTTTTTGTGCCCCAGGATGAACCCCCTTTTT
polyT_20	TTTTTCCCGCCGCGCTTTGGGCGCGTAACCACCACATTTTT
polyT_21	GTGCTCAGAGTGCACTAAATCGGAACTTTTT
polyT_22	GTAGTGGTCAGCGATCTGTCTTAATCAAGTTTTTTGGTTTTT
polyT_23	TTGTGGCTAACTCACGCAGAAGTGGTCCTGCAACTTTTTTT
polyT_24	TTTTTAGATGGCTGGCTGTACTCCAGCTTTTTT
polyT_25	TTTTTTCAGAAGCCAGTCAAAGGTTTTT
polyT_26	TAGAGCCCCTAGGGCGCTGGCTTTTT

Table S8 l Individual staple sequences for sc_EGFP1 20HB-int-W.

Name	Sequence 5' - 3'
core_1	tGGCAAAATCCCTTATAttGAGCAAAAttTTAATTTttCCCAATCCt
core_2	tAGCTCATTTTTTAACttAAAGGGAAttCCAAGCTttCCCACCCCt
core_3	tGTGCCACCTAAATTGttGAAGCATttTGATGGATttAGGAAAGt
core_4	tCACATTTCttACTTGGAAATCCCCGttATGGTAATttACTTGTGt
core_5	tACAGGAAttGAGATCCAGTTCGATttTCGGTCCTt
core_6	tTACTCTTCttAAAGTGCTCATCATTttGCACTGCt
core_7	tATATTTGAttGACCGAGTTGCTCTTGttACTCAACCttATTAATTGt
core_8	tCCCAACTGATCTTCAttTCAttATGTTGTGCAAAAAAGCGGTTAGCTCCTttGTAACCCAt
core_9	tCCGCTGTTttGGCAAAATGCCGCAAAttCAATAGGCt
core_10	tGGCGAAAACTCTCAAttGGCCGCAGttCTCGTCGTTTGGTATGt
core_11	tTTCTTCGGttGACACGGAAATGTTGAttAAATTTTTt
core_12	tGAACTTTAttCTTTTTCAATATTATTttTAAGCGTTt
core_13	tCCGCGCCAttCCGTAAGttGTTCGCCAGTTAATAttCTCCAGATttATAGAATAttGTTCACCTt
core_14	tACTCATGGTTATGGCAttGGAAAACGttACCTACTCt
core_15	tCATGCCATttCATAGCAttATTTTATTttATCTGCAttCGTAGGTGt
core_16	tGGTTCCCAACGATCAAttCCTGACTCttGCGCTTCTttCAGCAGCAt
core_17	tCAGCTCCttCCGATCGTTGTCAGAAGTAAGTTttGGATCTTAttTGCCCCACttTGGGTCTCt
core_18	tTGCCATTGCTACAGGttGCAATGATttGCTCGTCttTGCCGTCCt
core_19	tGAAGCTAGttGAGTGGCAttTCAATACGttGTTATTGTCTCATGAt
core_20	tTTGCCGGttCGGAAGGttGATCAGCGttGCCATGAttTGCCCTTCt
core_21	tATAGTTGttGGCGAGTttGCCTGCTttCTCGTGCAttCCAGCGTTTCTGGGTttAATCAAAAt
core_22	tGTAGATAACTACGATttGCTTCATTttTGCTGTCCt
core_23	tGACCCACGCTCACCGGttGTTTGCGCttATTTCCTCt
core_24	tAGTATATttTCTGGCCCCAGTGCTttCATCGTGGttAGAATGACt
core_25	tATCCTTTTttATCTTGAAttGGGCCCTttCAGCTTGCttGAATTCCAt
core_26	tCTGAAGCttAACTACGGCTACACTAttCCCCCTGGttAAGAAGTCttGACGTCAt

core_27	tTTCGGAAAAAGAGTTttTGACAGTTttTGGGGGTGttAACTCCAGt
core_28	tGGTAGCTCttCGGTGCTACAGAGTTCttTTCCGACCttCTGGACGTttCTATGGGAt
core_29	tTTGATCCGGCAAACAAACCACCGttCAATCTAAttCTGCACGCt
core_30	tGCCACTGttGGTGGTTTTTTTGTTTGCAAGCAt
core_31	tACGACTTATCGCCACttTCTCATAttTCGAACTTttGGGCCATTt
core_32	tGGTAAGACttCGCGCAGAAAAAAAGGATCTCAAttCATGAGATttGCTTGTCGttAGCTCTAt
core_33	tAACTATCGTCTTGAGttCGGTGTAttAGCTCGAttACTCCATAt
core_34	tTCGTGCGCTCTCCTGttTTGAAGTttTCGCCGAttACCAATGt
core_35	tTCTCAGTTttTCCAACCCttGTTCTTCTttTATCAAAAttCAATAAACCAGCCAGCt
core_36	tGGTCGTTCttCAATGAGTGAGCAAAAGGCCAGCttAACGCGGAttTGCGGTTCt
core_37	tCTATAAAttCACCCATTttGTGCTGCTTCATttGCTt
core_38	tAATCGACGCTCAAGTCttGGCGTTAttAGCCTTCGttGGTCAGGGttCAGGACCAttTCCCCCTt
core_39	tGACGAGCttTTACCGGATACCTGTCttAGCGAGGTttCGGCGAGt
core_40	tGCTGGCGTttGGGAAGCGTGGCGCTTttTGGCAGCAttTGTGGCGGttAAATTAAAt
core_41	tTATGAACTttGCGttTATATttGCTCCAAGCTGGGCTttCTGCGCCTt
core_42	tGATGTACTGCCAAGTGttGAGCTCTGttAGTGGGTttTACTTTCAt
core_43	tATAGGGGGCGTACTTGttACGCCTAttGAGTCGTAt
core_44	tGCCATTTAttGAGTTGTttGCTCGGTAttTAAGGGCt
core_45	tGTCCCATAAGGTCATttTGATTTTGttGCACACTGt
core_46	tCAAAACCGCATCACCttTGAGTCAAttCTCCTCGCt
core_47	tTTGCGTCAATGGGGCGttCCGTCATTttGCCAGGGCttGAAGATGttGTAGTGGTttATGTAGGt
core_48	tTTTGGAAAGTCCCGTttGTACTGGGttGATGAACTttCGCGGGTCttTCGATGTt
core_49	tGTGCCAAAttAATATTTTGTTAAAATTCGCGTTttATACTCAttGCGGCCGTttAGACAATt
core_50	tACAAACTCCCATTGAttACTGCCAAttGCATCGCttACCAGGGTttTGATGCCt
core_51	tTACTAGTttGTGGTGCAttCATAATGttGGTCGTTGGGCGGTCt
core_52	tGAGGGTGGttGACGTCAttACATACGTCATTATTGt
core_53	tCCTCGCCCttGCATTTAGttGACAGTGGttAGTAAGTAttATGCTTTTCTGTGACTGGTGAGTttCCCGGCGt
core_54	tTTGTAGTttTCCCTTCttTTTTCCATAGGCTCCGCCCCCCTttACGTCAAt
core_55	tGTCGCCCttGCTCACGCttAGGAACCGTAAAAAGGCCGCGTTttAGCCAGGCt
core_56	tTCCTCCTTttGCCGTTTAttGGCACAGttGAAGGCACt
core_57	tCGTTGGGGttCGGCTGAttCAGTAAGCttCTTATATttGAATAGACCGAGATAGGGTTGttTTt
core_58	tTGTGATCttCCCGTCGTttCTTAATCAGTGAGGCAt
core_59	tCCGGCGGCttGCTTACCAttATGAGTAAACTTGGTCt
core_60	tGTGACACTttTTATCAGttAGGATCTTCACCTAGttGCAGATTAt
core_61	tTCGAGGCTttGCCGAGCGttTTAAGGGATTTTGGTttGAAGATCCt
core_62	tATTGTCTTttCGATAAGCttAGCACTGCttATACACTTttATGGAAAGTCCCTATTttAGAGGTGt
core_63	tGCATCTTTttTCTCTAGTttACCGCATCttCAGGGCGttCCCttATTTCGTTCATCCtt
core_64	tACGGGAGGttGGTCACGttTTCTGCTGttGTGCGCTCttCTGCCGCttATCACAAAtt
core_65	tATTAGCAGttCGCCTTTCttTGCCGTCGttTGGGCGGGttCCAGGCGGtt
core_66	tGTAGGAAAttTACCGTAAGTTATGTttAAAAGGCCttTGTAGGTAtt
core_67	tCTAATACGTAGATGTttCGTCAATGttCGCCAGTGttTTATCAGGttGGATAATAtt
core_68	tAGACCTCCCACCGTACttGCATATGttACGCCGTAttGGCATGGttCGGGGCCGttGGTGGCCTttCAGTTACCtt
core_69	tTGGTCACttCCTATAGTttCCGCCCATttCGAAATCtt
core_70	tTCGCCGGAttGAGCGGCttGGGTGGAGttCCCGAAAAtt
core_71	tCGGACTTGttAAGCTCCCttGCGAAACCCGACAGGAtt
core_72	tTCCTTGAAttACGGGCAttGAGTGATCttCCAGAATttTGTCACGttTGTTATCtt
core_73	tCACCTCGGttTCAGGGTttCTAGATTAttGCGATGCAttAACGTTGTttATAATTCTCTTACTGTtt
core_74	tCATGCCGAttGCTTGCCGttGGATCCGAttTACGACATttGTTAAATCtt
core_75	tCTTGTACAttACCGCGAttAATGAAGTTTTAAATttCTGGTAGCttGTAACAGGtt
core_76	tCCTTCCAttACCATCTCttCACGCTGAttAGCGATGAttTATGGGCtt
polyT_1	tttttAAAAATAAACAAATAGGGGTTCCGCGttGCGGATACttCCTTGCTCttGGGTCAAGt
polyT_2	tttttTATTAAAGAACGTGGAttGGAACAAttGCGGGCGttTAGCCAGAttGGCAGTTt
polyT_3	tttttGCGAAAAACCGTCTAttCTCCAACt
polyT_4	tTCCAGTCTttAAGTCATTCTGAGAATAGtttttttttTGTATGCGGCttATGTATTTAGttttt
polyT_5	tttttTATCCGCCTCCAttGGGGGAGGGGCAAACAACttttt
polyT_6	tttttttCGTGAACCATCAttGACttAATGTCACATTCCGCCttttt
polyT_7	tttttCAGTGGAACGAAttGTTGTAGTttAACTAGAAt
polyT_8	tttttttGGTCGAGGTGCCGTAAAttATTTGGTATCTGCGCTCTGttCCTATCTttTGTCGGGt
polyT_9	tttttCGTTCAGCCCGACCGttGTGTGCACttGTTGCCGttCGTAATTGt
polyT_10	tttttCCTAAAGGGAGCCCCCttACCAGGCGTTTttGAAGAACt
polyT_11	tttttATAACTAGTCAATAATCAATGTCAACttAATGACCCt
polyT_12	tttttCCGGCGAACGTGGCGAttCGTAAATACTCttGATttGATTTAGAGCTTGACGGGGAAAGttttt
polyT_13	tttttTGATGTACTGCttATTACTATTAttttt
polyT_14	tttttATGGGCACCACCCCGGttTCCAGCTCGACCAGGttttt
polyT_15	tttttAAGTGTAGCGGTttTACttGAAAGGAAGGGAAGAttttttttttAAGCGAAAGGAttGAGTCCACttttt
polyT_16	tCACttGTGGTCGGGGTAGttTCTTTGCTttCCCAGCATttTACATGATCCCCCttGGGCGATGGCCCACTAttttt
polyT_17	tCGTCGCCGttTGAACAGttACCGCTATCCACGCCCATttttt
polyT_18	tGAAGTCGAttTATAGACGttTATCCGGTttTTTGATCTTTTCTACGGGGTCTGACGCTttttt
polyT_19	tttttTGTGCCCCAGGATttGAACCCCCttttt

polyT_20	ttttCCCGCCGCGCTTttTGGttGCGCGTAACCACCACAtttt
polyT_21	tGTGCTCAGttAGTttGCACTAAATCGGAACtttt
polyT_22	tGTAGTGGTttCAGCGATCTGTCTttTAATCAAGTTTTTTGGtttt
polyT_23	tTTGTGGCTttAACTCACGttCAGAAGTGGTCCTGCAACTTtttt
polyT_24	ttttAGATGGCTGGCttTGTACTCCAGCTtttt
polyT_25	ttttTCAGAAGCCAttGTCAAAGGtttt
polyT_26	tTAGAGCCCttCTAGGGCGCTGGCtttt

2.2.1.3 sc_EGFP1 32HB

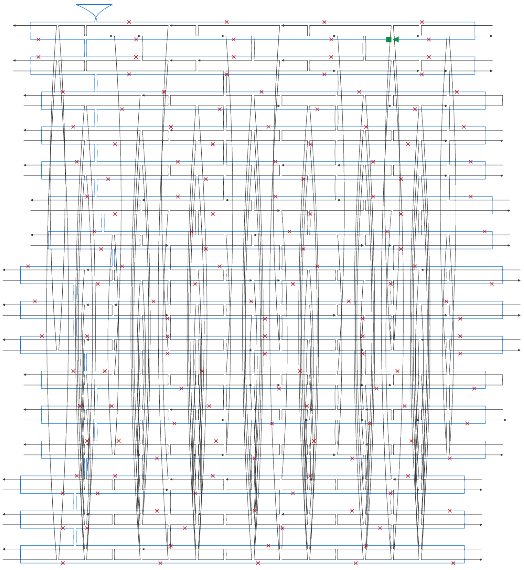

Fig. S22 l Scaffold routing and staple design for sc_EGFP1 32HB. Scaffold routing is shown in blue, staples are given in black, breakpoint for addition of sequence is marked by a green square (5') and arrow (3'). Design was prepared using caDNAno v0.1.

Table S9 l Individual staple sequences for sc_EGFP1 32HB.

Name	Sequence 5' - 3'
core_1	GAACCGTACCACTACGTGAACCATGCCGTAAA
core_2	ACCGTAAGTTATGTACTGCATTTACCGTCATTGACGTCAATAGGGGGCCAGGCG
core_3	GGCGTTACCAAAAAGGTTTTGTTACCTAAATGCACTAAAAGCCATAGAGCCCACC
core_4	GCGTACTTCATAAGGTCATGTACTTTGGTGCCTGCTTAATGACTCCTATTATTG
core_5	CAAGTAGCGAAAACGGGCGGGGATTACTATTAATAACTTTCCATAG
core_6	TACGTAGATGTACTGCATGGGGTGATATGAGTGGGAGGGTGTTGAAGTGCACCC
core_7	AATAGCGAGAGATTATTATGGGAATCAACGCGTATATCAA
core_8	ACCGCATCACCATGGTCAAACCGAAAAATGACAATGATACGCAAAATTTCACCA
core_9	TGGGTTCTCTAGTTAGAGTGAGTCAGATTTATTGGTATG
core_10	CTCTGATGGATATCTGCAGAATTGGGGCGGATAGTTGCCTCAGTGAG
core_11	CCAGCACATTATCCGCGTTAATAATCATTGGGCACTGCA
core_12	CTGGCGGCTCGCCCTTGCTCACTGGGCCAGCCGATCGT
core_13	GCTTGGGTCTCCCTATCCAGAGAAGTGCTGAGTTTTAAAGTGCC
core_14	TTGTGGCCGTTTACGTGTGGCATATCAAGG
core_15	GCAGCTTGCCGGTGGTCCCGGTGTGCCATTGAGCGCAGCCCACTCTACTCATA
core_16	GCAGATGGTGCAAAAGGTTCACCTGCTTGTCGGCCATG
core_17	AACTTCAGTGGCGGACTTGAAGAAGTCGTGCCTCGATGC
core_18	GGTCAGCTTGCCGTAGCGCCGTCTCGTCGTTCAGCAATCTTTTACAAGGGAAT
core_19	ATGGTGCGCTCCTGGACGTAGCCAACTTCACCGAGTTACGGTGAGTACCGGCGTC
core_20	TGCCGTCCTGTCAGAATATGGCAAAAACGTTGGTCAAGGTCCTCATTTTATTAG
core_21	TCCTTGAATGTTGTAGTTGTACTGGGCGGAC
core_22	CTCGGCGGATCTTGAAGTTCACGTGTTCTG
core_23	GTTGTGGCGTCGATGCCCTTCAGTGCTTCAT
core_24	CGTTCTTCAGGGTGTCGCCCTCGTTCGGGCA
core_25	CTTGATGCTGTGACTATGATCCCTGTCACGCCAGCTCGA
core_26	GGGCAGCATCACGAACTCCAGCAGGGCAAACA
core_27	CTTCTCGTTGGGGTCCCTTCCAGCTTCGGGGCGAAGC

core_28	TGATCGCGTGGGTGCTCAGGTAGTGGTTGTCATATAGACTTACTGTCAGCTCCTT
core_29	TCGTCCATGCCGAGAGCACAGTCGAATACGGGTTCAGCAT
core_30	AGGCTGATGTCCTGCCCCACCCACATTCCAAGGGCGATCCCCGAA
core_31	ATGACACCTACTCAGCGTAACCACTCTTCCTAAAATAAA
core_32	AGAATAGACTGGCAACTAGAAGGTGATCCCTGCTTTTC
core_33	CACCCCCCAACTGATCATAATACCATCGTGGCCATGTT
core_34	CCCCTTGCTCAGCGAGCTCTAGCAGATTACTT
core_35	GGGCGCTGATCAGGGTTATTCTATATTTCGTAATTGTT
core_36	CCACACCGCTTGACGGGGAAAGGGACTCCA
core_37	GCCTCAGATCGGAACCCTAAAGGTATCAGG
core_38	GAAAGGAAGGGAAGATTTGGAACGATAGGGTCTCCATA
core_39	CACTATTAAAGAACGTCCGGCGAATATTTAGATTTTCAACCGTCGTGCTGCAACT
core_40	AGGGCGAAAAACCGTCGAGCCCCCCGCACATTCACGGAAA
core_41	TGAGTGTCAAGAAGACTCAGCGATCCTTTGGAAAGTCCCGTTGATTGGGCATA
core_42	TATAAATCTGACGCTGTTACCAAAAAACAACCCATTTGCGTCAAT
core_43	AAATCCCTGTAATTGGTCGTTGGGCGGTCA
core_44	AATCGGCAACGTCAAGCTCCGCCCCCCTGACGAGCATCATGACCCC
core_45	TAACCAATAGGGATTTTAAAGTATGAGACTTTATATAGACCTCCCACCGAGCTCGAAGGGCCG
core_46	TCATTTTTAATCAATGCATACGTCATTATTGTGCCAAG
core_47	AAATCAGCGCGATGGCAAAAGGCCGCGTTGCTGGCGTTTAGTCAAT
core_48	ATACATATTAGACCGAAAGAGTCCGACGCTCAAGTCAGAGGTGCAGACGCGGAA
core_49	TGTTGAGATGCAATTAAGGCACGGGGGAGGGACCATGTAATTCTC
core_50	AGTGCTCGTTTGCGCCGGTCCTGGCACGGGTGGTCGGGGTAGCGGCTGAAGC
core_51	ACTTTAAACGATGTAAAAGTGGTCTAGATAACGCCTACCGACTCCCATTGACGTCA
core_52	AAGCGGTTATGCCATATAGCAGAACAGATGG
core_53	AAGCTAGGGCCGCAGGTCGGTCAGGGTGGTCACGAGGGCATCTCGAGTTCGCCA
core_54	AACGTTGTAACAGCTCCCGTTACTAGTGGATCCGTACACTACGATACAAACTTGCAAATAG
core_55	CTACAGGCGCGCCACCCGTAAGAGGCGGCGGGCACGGGGCCGTCGCCGATGGGG
core_56	CTCCATCCTTACCGCAAGCATTTGCAAGTG
core_57	AAACCAGCTGGCCCCGCTCTGCTGGAAATCCCCGTGAGT
core_58	CTTACCATCCAGCCGGGTACCAACCAGGATGGGCACCAC
core_59	GTCTGACACAGTGGAAGAAAGTCCGGCATATGATACACTTGATGTACACGTCAAT
core_60	AATCAATCTGGTCATTGACTAATGGGCAGTTTACCGTAAATACTCC
core_61	TCTTTTCGGCCATTTTATGGGCTATGAACTAACAAAAAT
core_62	TACGGGGTCAAAAGAATTGAATGCGTGGCGATAGCGGTCACGCTGCGACAATGCGATCCAGTT
core_63	TCACGTTAAGGCCGAGGGTTCCGGATTTAGACGCCGCGCTTAATGTC
polyT_1	CTCTTGCCTCAACCAGTACAGCCTGGTAGTGGTCGGCGAGCTGCACGCTGTTTTT
polyT_2	TTTTTCCAGCAAAAGGCCAGTGAGTGACGTTAAATATCTTCACCTAGTTTTT
polyT_3	TTTTTGGCGTTTCCCCCTGGGACTATAATGCTACAGTGTTCCAGAAGCGAA
polyT_4	TTTTTTGGAAAGTCCCTATTACCCATT
polyT_5	TTTTTCTCTCCTGTTCCGACCAAGCTCCCAGGATCTAGTTCTTGAAGTTTTTT
polyT_6	TTTTTCTGTCCGCCTTTCTCTCAAGCAGCAGATTACGTTTTT
polyT_7	TTTTTCAGTAAGCAGGCCCATTGATGTTTTTTTTTTTACTGCCAAAGACGTCAATTTTT
polyT_8	TTTTTCGCTTTCTCATAGGCCCCACCGCTGGTAGCGGTTTTT
polyT_9	TTTTTCACGCTGAACTTTCGATAAGCTTTTT
polyT_10	TTTTTCAGTTCGGTGTAGAAAGAGTTGGTAGCTCTTTTTTT
polyT_11	TTTTTTCCTTGAAGAAGCCCTCGCCGGATTTTT
polyT_12	TTTTTTGGGCTGTGTGCACGACCCAGGATGTACTGCACGCCGTAGTTCGCTCCAAGCTTTTT
polyT_13	TTTTTCCGTCCTCGATGTGAGAATAGTTTTTT
polyT_14	TTTTTCGACCGCTGCGCCTTATCTTTGCTCACCAGCTTGCTCATGGT
polyT_15	TTTTTAGAATAGGGCCCTCTATTTAGGTGACACTATTTTTT
polyT_16	TTTTTCGGTAAGACACGACTTGGGAGTGGCACGGTAACT
polyT_17	AATCCTCGCGTTTCTCCGAGTTGGCTTCATTCAGCTTTTTT
polyT_18	TTTTTCTGCTATTGTCTTCCCGCATCCCCAGCATGCTTTTT
polyT_19	TTTTTAGCCACTGGTAACAGGAGCGGGCGCTAGAAAGGAC
polyT_20	TTTTTTTTTGGGGTCGAGGTCACCCTAATCAAGTTTTTTT
polyT_21	TTTTTTATGTAGGCGGAGATACCATTTTT
polyT_22	TTTTTGGTGGCCTAACTAAGGATTAGCAGAGCGAGGTTTTT
polyT_23	CGGGTCTCATGAGCGGGCACCTATTCCTTTGAATGCCAGGCGGGCCCGCTTACCGGATACTTTTT
polyT_24	TGTAAGCGCAAAATGCCCGCGAGACCCACTTTTT
polyT_25	TTTTTAAACAGGAAGGTTAATATTTTTTTTTTTTTTGTTAAAATTCGGCAAAAGGTTTTT
polyT_26	TTTTTAGTATTTGGTATCAGTATCGCCACTGGCAGCTTTTT
polyT_27	TGCAACTCTCAAGGATCAGTCTATTTCATCCAGTTGTTACGACATCTTCGGGAAGCGTGGTTTTT
polyT_28	TTTTTGTATGCGGCGAGGGTGAGCAATTTTT
polyT_29	TTTTTGTTACCTTCGGAAATCGTCTTGAGTCCAACCTTTTT
polyT_30	TGTTATCATGCACCCCCCGTTCAGCCTTTTT
polyT_31	AGTCATTCTTGTGGCGCGGGTCTTGTAGTTGCCGTCGTTTTT
polyT_32	GTAAGTTAGTAAGTAGCGGCCGCCAGTGACGCTGTAGGTATCTTTTTT
polyT_33	TTTTTCCGGTTCCCAACGCGCCCTCG

polyT_34	TTTTTGATCCGGCAAACAAAGGGTCTGCTGAAGCCATTTTT
polyT_35	TTTTTGCTCACCGGCTCCGTATTAA
polyT_36	TTTTTTGGTTTTTTTGTTTGGTCCACTAGAAGAACTTTTT
polyT_37	TTTTTATCCTTTTAAATTCTATCCAC
polyT_38	TTTTTCGCAGAAAAAATCGTGCGTTTTT

2.2.1.4 sc_EGFP1 12HB

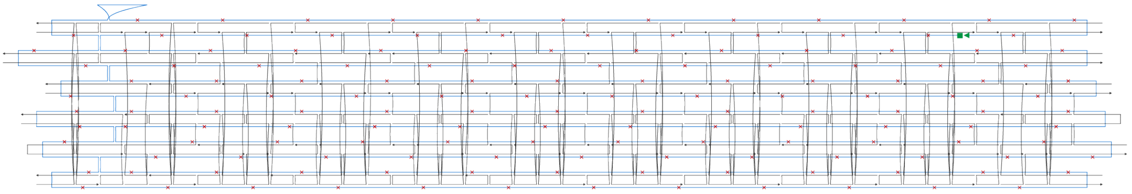

Fig. S23 l Scaffold routing and staple design for sc_EGFP1 12HB. Scaffold routing is shown in blue, staples are given in black, breakpoint for addition of sequence is marked by a green square (5') and arrow (3'). Design was prepared using caDNAno v0.1.

Table S10 l Individual staple sequences for sc_EGFP1 12HB.

Name	Sequence 5' - 3'
core_1	TGCACGAAGTCCTCCGATCGTTGTTAAAATTC
core_2	CGTTCGCTCCAAGCTGGGCTGTGGTACTGCC
core_3	GTGTAGGTCGCAGTGTTATCACTCGCCACCTA
core_4	CACGCTGTAGGTATCTCAGTTCGCTCCACCC
core_5	TCATAGCTCTGCATAATTCTCTTTAGGGGTT
core_6	CCTTCGGGAAGCGTGGCGCTTTCATTGGCGT
core_7	CTTTCTCTAAGATGCTTTTCTGATATTTGA
core_8	GCCGCTTACCGGATACCTGTCCGCTTGACGT
core_9	CGACCCTAACCAAGTCATTCTGTTATCAGG
core_10	CTCCCTCGTGCGCTCTCCTGTTCTCAGCCA
core_11	CCTGGAAGCGACCGAGTTGCTCTACTCTTC
core_12	TATAAAGATACCAGGCGTTTCCCTGTAACGC
core_13	GACAGGACCGGGATAATACCGCGCTAAGGGC
core_14	TCAAGTCAGAGGTGGCGAAACCCACTAATGA
core_15	ATCGACGCTAAAAGTGCTCATCATACAGGAAG
core_16	CCCCCCTGACGAGCATCACAAAAAACTAGTC
core_17	AGGCTCCGGGGGCGAAAACTCTCA
core_18	CCGCGTTGCTGGCGTTTTTCCATTCAATGAG
core_19	TCATTGACGTCAATAGTAGGTGCAGATGAACTTCAGGGTCAGCTTGGGCGGGCCCGCGCAGA
core_20	CCGTAGGTACGAGGGTGGGCCAGGCATTTA
core_21	GGCATCGCCCTCGCCCTCGCCGGCCCATAAGTTTGATCTATGGCTTC
core_22	ACACGCTGGAAGCACTGCACGCCCTAGATT
core_23	AACTTGTGGCCGTTTACGTCGCCATACGTA
core_24	GTCCAGCTCGTGCTGCTTCATGTAGAGTGAT
core_25	CGACCAGGATGGGCACCACCCCGAACCGCATCATGAGATAGTTTG
core_26	GTGAACAGTAGCCTTCGGGCATGGCAGGACC
core_27	CTCCTCGCCCTTGCTCACCATCTCCGCTATCGATCCTTT
core_28	CGAGCGGGTCCTTGAAGAAGATGGTCTTTGC
core_29	CCGCCAGTGTGATGGATATCTGCAGGTGGAGATCAATCT
core_30	GAATTCCTCACCTCGGCGCGGGTTAGTGGTT
core_31	AGCACACTGGCGGCCGTTACTAGTGCCAAAACTGACAG
core_32	TGGATCCGATGCGGTTCACCAGGGCGCCGATG
core_33	AGCTCGGTACCAAGCTTGGGTCTACGACATTCACCTATC
core_34	CCCTATAGTCCTCCTTGAAGTCGGGCGAGC
core_35	TGAGTCGTATTAATTTCGATAAGCGCCCATTATCCATAG
core_36	CCAGTAAGGTACTCCAGCTTGTGTGGCGGA
core_37	GTAGGTCAAAGATCCGTCATGTAAGTTTACCGTAAATA
core_38	GGTCGGGGACGCTCAGGATGTACTTCAATGGAAAGTCCCT
core_39	GCGGACTTGATTTTGGTCACCATGGGAACATACGTCATTA
core_40	GTGCGCTTTCACCTACACGCCCGGGGGTCGTTGGGCGG
core_41	CTTGTAGGTTTTAAACTTGGAAATTTACCGTAAGTTA
core_42	TGTCGCCCAACTTGGTCAAACTCCATATATGGGCTATGA
core_43	ATGCCCTTAGTGAGGTTGGAAAGTTGATTACTATTAAT

core_44	CCCCAGGATTCGTTCTGCGTCAAATGTCAACGCGTATA
core_45	GGCTGGCAACTAGAAGCAATAGGCGTTACATG
core_46	GGAAGGCACGGGGGAGAATCAAAAATTCAGCTAAGTTGGC
core_47	GACAGTGGGAGTGGCTGTTGTTCTGGTGTCATGGCAGCA
core_48	GCGATGCAATTTCCTTTAAAGACGCAACGTGCCATCCG
core_49	CCCAGAATAGAATGAGAAAAACAGAGTAAGAGTACTC
core_50	CTCCCCCTTGCTGTCTACGTGAACATCCAGGTATGCGG
core_51	CCCCAGCATGCCTGCTGGGGTCGAGCAGAAGTGTCAATA
core_52	TCCGCCTCAGAAGCCAAACCCTAAGCAATAAAAGAACTT
core_53	ACCACCACACCCGCCGGACGGGGAAGACCCACCGTTCTTC
core_54	CTGGCAAGTGTAGCGGGAAGGGACATCTGGCTACCGCTG
core_55	GCAAAATCCCTTATAGGGCAAACATCTCAAGGGGTGGTC
core_56	GAATAGAAATTGTAAGCGTTAATATTTTGTCAGAAGT
core_57	CCGAGATAGGGTTGAGACCTTCCAGGGTCTGTAGCGGCT
core_58	GAACAAGAGTCCACTACATTTTATGTTAAGGGAAGAAGT
core_59	ACGTGGACATGTATTTAGAAAAATAAACAAA
core_60	TCCAACGTCAAAGGGCCACCTACTAAAGGATCCCTGGACG
core_61	AGGGCGATGGCCCACCTGCCCCAAAAATGAATTGCCGTC
core_62	CTAATCAAGTTTTTTATTGTCTTATGAGTATCGAACT
core_63	AAAGCACTAAATCGGTAGAGCCGCTTAATCCAGCTCG
core_64	CCCGATTTAGAGCTTCGCTTAATCTGTCTATTGTTGCCG
core_65	TACTTTCACGAACGTGGCGAGAAAGTCACGCT
core_66	TCCTTCGCCCCCCGTTCAGCCCGACCGACTGGGGGCGT
core_67	ATGGTTACGCTCGTCATTGACGGCCAAGTAGGAAAGT
core_68	ACTGTCATTGTTGCCATACTATGGTAATAGCGATGACTA
core_69	TGACTGGTGTAGTTCGCAATGGGCATTGATGTACTGCCAA
core_70	AGAATAGTTCTATTAAGGCGGGCCATCCCCGTGAGTCAAA
core_71	TGCCCGGCGGTCCTGGGAACTCCCATTGACGTCAATGG
core_72	CACATAGCCCAGCCACCCCGTAATCCCGTTGATTTTGG
core_73	TGGAAAAGCTCACCGAATAATCATGGGGCGGAGTTGTT
core_74	CAAACAACGCTCTGCTGAAATTTTTTAACGCACAGT
core_75	CAAGGCGACGAAATCGGCGTTAAATTTTTGTTAAATCAGCGGTTAGCATCCCCCACTTGGCA
core_76	CAACGATAAAAAAGGAACAGATGGTGACACTATAGAATAGGGCCCT
core_77	CCGGTTCCAAGTGGGCCTGGGCATAATGCCA
core_78	GTTTGGTTTTCTACGGGGTCAAACTTGTACAGCTCGTCCATGCCG
core_79	TTGCTACAAAACTCACTAGGAAAGCCCGGCGGCGGTCACGAACTCCA
core_80	CCAGTTAATTATCAACAGACAATATGTGATCGCGCTTCTCGTTGGG
core_81	GGGAAGCTCGTCTATCGTTATTGTCTCATGAGCGGATAC
core_82	TTGTTGCCTAAATTACCCCACCCTCAGGGCGGACTGGGTGCTCAGG
core_83	TCCGCCTCCCATCACCCTTTTTCAATATTATTGAAGCAT
core_84	CAACTTTAAAAGTATATCCCAATCGTCGGGCAGCAGCACGGGGCCGT
core_85	GGCCGAGCGGTGCCGTGACACGGAAATGTTGAATACTCAT
core_86	GCCGGAAGTTACCAATCACCGCATGGGGTGTTCTGCTGGTAGTGGTC
core_87	ATTTATCAAGGGAGCCGCAAAATGCCGCAAAAAAGGGAA
core_88	GCTCCAGTCAGCGATGTCACATTGCACGCTGCCGTCCTCGATGTTG
core_89	ATACCGCGAAGCCGGCCAGCGTTTCTGGGTGAGCAAAA
core_90	TGCAATGTTGCCTGAGCGCGTATCTTGAAGTTCACCTTGATGCCGTGTGGCTG
core_91	CCCAGTGCTGAGCAAACCGTACACGCCTAC
core_92	CAGATTAATTTACCGTATGATACACTTGAT
core_93	TGGAACGAGGCATCGCAGTTTGCCGCGCACATTTCCCCGAAAAGT
polyT_1	TTTTTACCGTAAAAAGGTTGAGATCCAGTTCGATGTATTTTT
polyT_2	TTTTTCCCGGTAAGACACGAATCGTCTTTGATCCGGCAAAAAAG
polyT_3	TTTTTTCTGCTTATATAATAACTACGATACTTTTT
polyT_4	TTTTTGTAACAGGATTAGCAGAGCGAGGTATCTTATCGCGTGGTTT
polyT_5	TTTTTCCATGATATAGACGTTTCTTCTG
polyT_6	TTTTTTCTTGAAGTGGTGGCCTGGCTGATCAGCGAGCTCTAGGCACGGG
polyT_7	TTTTTTATTTGGTATCTGACCACCGCTTTTT
polyT_8	CTCGCCAGTTACCTTCGGAAAAAGAGTTTTTTTTTTTTGGTAGCTCTGAGTCCAATTTTT
polyT_9	TTTTTACCCACTCGTGCAGAAAGGAGCGGTTTTTTTTTTGCGCTAGGGCGCTTGTCGGTTTTT
polyT_10	ATGTTGTGTTTTGTTTCGAAACTACGGCTACACTAGAAGAACAGTTTTT
polyT_11	TTTTTGGGAGGGCTTACAGAAAGCCCCAACTGATCTTCAGCATCTTTAGGATCT
polyT_12	TTTTTTGGTAGCGCACTGGCAGCAGCCACTGTTTTT
polyT_13	GCAAGCAGCAGCTTGCCGGTGGCGGTGCTACAGAGTTTTTT
polyT_14	CTCCCCGTTGTAGTTCAGTGGGTTCTCTAGTTAGCCAGAGAGCTTTTT
polyT_15	TCGTGTAGGACCTCCCAAGGCCAGCAAAAGGCCAGGATTTTT

2.2.1.5 sc_EGFP1 20HB-LS

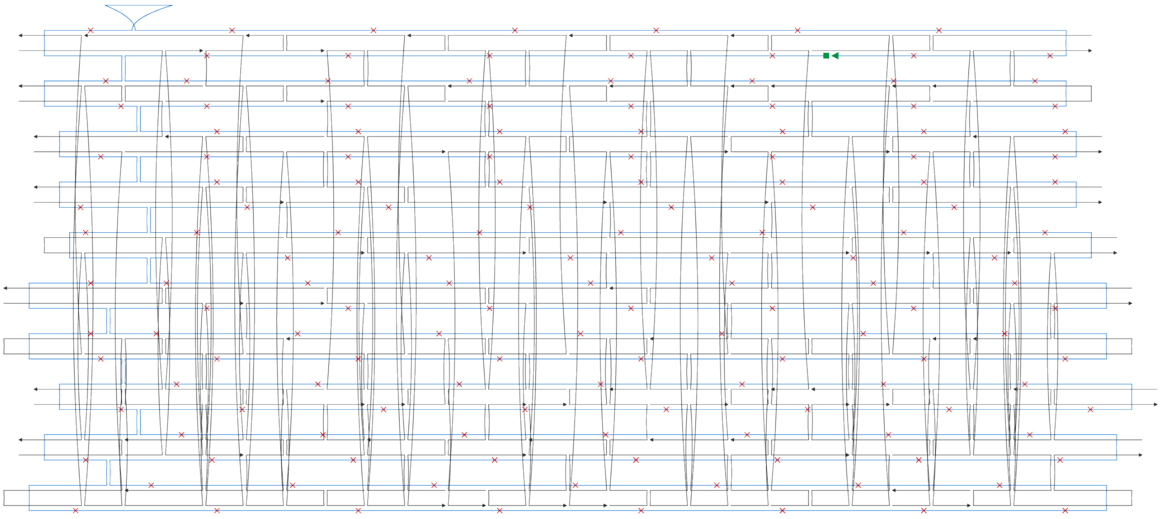

Fig. S24 | Scaffold routing and staple design for sc_EGFP1 20HB-LS. Scaffold routing is shown in blue, staples are given in black, breakpoint for addition of sequence is marked by a green square (5') and arrow (3'). Design was prepared using caDNAno v0.1.

Table S11 | Individual staple sequences for sc_EGFP1 20HB-LS.

Name	Sequence 5' - 3'
core_1	CGCGTATATCAATGAGTGAGCAACCATAGGCTTAAATTTTTGTTAACAAAAGAA
core_2	CGTAAATAACTAATGACCCCGTAAACCCGACA
core_3	CAAAACAAACTCCCATTGACGTCGGAACATAAACCACCGGTTGAATACATGCCATTCACCCTAGGGAGCCCCCGATTTA
core_4	TCAAACCGCGCCTACCGCCCATTTGCGTCAATTTTTTGTTAAGTCCCTTGTAACGCGGAACTCCTCCCCCTG
core_5	GCCAAAAGCTCTGCTTATATAGTCCTCGCCGGCACCTCAGAAGTGTGTGCAAAAAAGCGG
core_6	GACTAATGTAAGCAGTGGGTTCGCCAGTGTGGTCTGACATCCAGTCCGATCAA
core_7	AAAGTCCCTATAGTGAGTCGTATCACACTGAAATCAAT
core_8	AGTTGTTACGACATTTACTTGTGGTAGATAACGACCCACGAGTGTTATGGCTGGCAACTAGAAGGCACAGT
core_9	TCTAGTTATTTTCTACCACTTGATAACTAGTCAATAATCAAAAAATCG
core_10	TAATTTCGAAAACTCACGTCAATAGGGGGCGTACTTGGCA
core_11	CCCGCCCTCGCCGGACACGCTGATGGAAAGGCAAACACGTCATTATCAGCCAGGCGGGCCGTTCCGAC
core_12	GGCCAGGACCCCGGTGAACAGCACCTCCCAAAAAAAAG
core_13	TCATGTGGTCGGGGTAGCGGCTGAAGCACTGCACGCCGTACCATCTCGAGCGGCC
core_14	TTGAAGTTCACCTTGTGTACTCCAGCTTGTGCCCCAGGGGTGGTGCTTTATCAGTAGTTGCCTGTGACT
core_15	CACGCTGCCGTCCTCGATGTTGTGGCGGATCCGAGGCTGGTTGTCAG
core_16	AGTGGTCGGCGAGCTGTGACACTATAGAATACCTACTCAGACAATGCGATGCAAGAGAAAGTTAGCTCCAGTCATT
core_17	GGCCGTCGCCGATGGGGGTGTTCTGCTGGTATGCGGTTCACCAGGGTGTCGCCCTCGAACT
core_18	TCGGGCAGCAGCACGGTCACCTCGGCGCGGGTCTTGTAGGTGCTGCTGGAAGCTACTAAAGTCGCCACAT
core_19	GGGCCCTCTAGATTACTTGTACAGGCGAGTTACATGATC
core_20	GCTCGTCCGCCCCACCCCACCCCCCAGAATAGGGCGCTAGGGCGCTGAAGAGTCCACTATTA
core_21	AGTGGCACCTTCCAGGGTCAAGGAAGGCACAATCGGAAGTTATGGCAGCAACA
core_22	TTGTCTTCCCAATCCTCCCCCTTGCTGTCCTATGCCGAGAGTGATCC
core_23	ACGGGGAAAGCCGGCGAACGTGGCTTTCCTCATTTTATTAGGAAAGGACAGTGGGGAGCTTGAAGTAAGTTGCTTTTC
core_24	GAAGGGAAGAAAGCGAAAGGAGCGAATGACA
core_25	ATCAAGTTTTTTGGGGGCATTTATCTTCCTTTTGGGCGG
core_26	TACAGAGTTATTGAATCGAGGTGCTCTGCATAATTCTCGGGAGGGCTTACCAT
core_27	ATTGTCTCATGAGCGGATACATAGGGCGACATAAGTTAATTGGCGTTACTATGAATGGGGT
core_28	TATTTAGAAAAATAAAAAACCGTCCTCAACCATTCGGTCCAGCCAGCCGGAAGG
core_29	CGCACATTTCCCCGAAAGAACGTCGGCGACCCCCCATGTGTCCTGCAACTTTATCCGCCTCCAGTTACCTCTTGCCC
core_30	AAAGTGCCTTTCACCACTATTAATGTACTGCCAAGTGGG
core_31	TGTAAGCGTTAATATTGTTCCAGTACGGGAGTTCCCAATATTAATTGTTGCCG

core_32	ATCAGCTCCTGTTGAGCAAAAGGCGGCGGGCCATTTACCG
core_33	CGGAAATCTGGTAGCCGTCGTGCCGTTTACCTTCAGGGTCAGCTTGTTGTAGT
core_34	GGGAATAATTTGAATGGAAGCTCCCTCGTGCGCTCTCCTATTTACCG
core_35	GCAAAAAAGGTGAGTATATCAGGGCGATGGCCCACTACGTGAACCA
core_36	CAAAAACAGGGTTCCGGGACTATAAAGATACCAGGCGTTATATATGGAAATGCCGCAGATTA
core_37	GGGTGAGAGTGTATGGGACTCCAACGTCAAAGGGCGAACAAATAGGGAAGGCAGCTATGACTCCACCCATTGACG
core_38	TCTTTTACACCTAAATACGCTCAAGTCAGAGGTGGCGAATTGATTAGCGTTTCT
core_39	CCCACTCAATTCGCGTCCGCCCCCCTGACGAGCATCACATGTCAAAACTGATC
core_40	CCGTAAGATGGCCGCCTCACCGGCTCCAGAAGATGAAGTCGCCGTCCAGCTCGACCAGGA
core_41	GAGTTGCAATGCTTACTTTGATCGCCAGAGACCGCATCACCATGGTAATAGCGATTATGATA
core_42	TTTAAAATCGTCGTTTAGTTCGCACTGGGTGCTCAGGTAGTGGTTGCGGCGGCGGTCACGA
core_43	GAGTAAGTGGTATGGCTTCATTCGTAACCACCACACCCGCCGCGCGCCTGCTAACTCCAGCAGGACCA
core_44	TACGATACTTACTGTCTCATACTCAGGGTTCCTGCCGCTTACCGGATACCTCTTCGGGAA
core_45	TGACTCCCGGTGGTTGGGGCGGGGAGACTTGGAAATCCCCGTGAGTCAATGGATGCAAGCA
core_46	TCATCCACAATAAACCTCCGATCATCAGCGAGCTCTAGCATTTAGG
core_47	TATTTCGTTGGGCACCGCACGGGCAGCTTGCCATGTTGCCGTCCTCCTTGAAGTCGATGCCCTTCAGCTCG
core_48	GATCTGTCCGCGCAGCCGTACACTATCCACGCCCATTGATGTACTCAGTTTACGATCTCAA
core_49	ATATATGAGTGGAACGATAAGCCAACGTAGATGTACTGCCAAGTAGGTCATTGA
core_50	GAAGATCATCAGTGACTTGCTCAGGTCAGGGTGGTCACGAGGGTGGCCGAGCGATCTCAGCCTGAGAAT
core_51	GGGGTCTGTTCAGCAGGCGTCAATTTGGAACGCAAGTGTAGCGGTCACGCTGCGCAGCTCCGTAATACCG
core_52	ACGCTCAGTAAACTTGATGGATATCTGCAGAATTCCAG
core_53	CGTTAAGCGATGTAAAGCAGAACTAGACCGAGATAGGGTTGAGTGTTTTGTTAAGTGCACCC
polyT_1	TTTTTAAAAAGGCCGCGTTGCTGGCGTTTTTAAGGCCAGATCCAGTTGGATTTTG
polyT_2	TTTTTCTTTCTCATAGCTCACGGGGGTCGTTTCAATAGGTAGCTCTTGATCCGTCCCGTTG
polyT_3	TTGACGTCAATGGGCTTCGGTGCATTTTGCTCCAAGCTGGGCTGTTTTT
polyT_4	TTTTTGTTCGGTGTAGGTCGGCTGTAGAAAGAGTTTTCTTGAAGTGGTTTTTT
polyT_5	ATAAGGTCATGTACTGGGTTTTTTTTTTCATAATGCCACAGGAACCGTTTTTT
polyT_6	TTTTTGTACCAAGCTTGATTATCAAAAAGGATCTTTTTTTTTTTCACCTAGATCCCAACGTTGTTGCTTTTT
polyT_7	TTTTTTGTGCACGAACCCTGTCTGCTGAAGCCAGTTTTTTTTTTTTACCTTCGGAAGTATCTCATTTTT
polyT_8	TTTTTACGTAGCCTTCGAGTTTGCGTTTTAAATCATTGGAACTTACCG
polyT_9	TTTTTTGCGCCTTATCCGGTAAGATATAGACGTTGTGGCTGCCGTAGGACCGCGA
polyT_10	ATGCCGTTCTTCTGCTTGTCGGCGCAATGATTGGCATCGCCCTCCGTTCAGCCCGACCGCTTTTT
polyT_11	CAGGGCGGCAGTTAATGGCATGGCTACTAGTGGATCCGAGCTCGTTTTT
polyT_12	TTTTTGTTGGGGTCTTTGCTTGTGATCGATCGTGGACATTCCGAAATCCCTTATAAAT
polyT_13	TTTTTCATAGAGCCCACCGCATCCCCAGCATTTAATGTCTGTCACGCGTGCTCATTAAAAATGTCATGAGGGTCTCCC
polyT_14	TTTTTCACTGGTAACAGGATTGTAAAGCACTAGGGGACTTATCGCCACTGGCAGCAGCTTTTT
polyT_15	ATTTTTTAACCAATAGGCTTTTTTTTTTCGAAATCGGCACCTCAGAAGCTTTTT
polyT_16	TTTTTGAAAACTCTCAAGGATAACGTTCTTCGGGGCTTTTT
polyT_17	TTTTTGGCCTAACTACGGCCAGCAGAGCGAGGTATTTTTTTTTTGTAGGCGGTGCGCGTGGCGTTTTT
polyT_18	TTTTTCATTGCTACAGGCCGCTTCTCTTTTT
polyT_19	TTTTTTGGTATCTGCGCGCCCTAGAAGACCCGGTAAGACACGGAGGGGCAAACAACAGATCACTCATGCCCTAAA
polyT_20	CCAGTGCTCATCTATCGTCTTGAGTCTTTTTTTTTTCAAACAGTATTTTTTT
polyT_21	GAAGTTTTGCGGCCGTGGACTTGAAGAAGTCTTGCCGTCGTCCTTGAAGAAGATGGTGCGCTCCTGGTTTTT

2.2.1.6 sc_EGFP1 20HB-LP

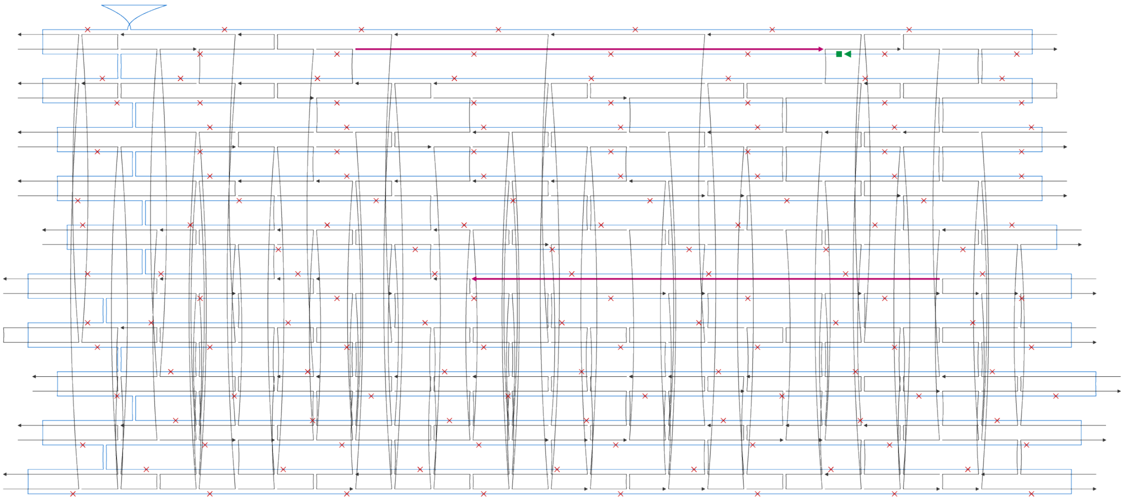

Fig. S25 | Scaffold routing and staple design for sc_EGFP1 20HB-LP. Scaffold routing is shown in blue, staples are given in black, breakpoint for addition of sequence is marked by a green square (5') and arrow (3'). Long 93-mer 'primer' sequences at the 5' promoter and 3' polyA regions flanking the EGFP gene are given in pink. Design was prepared using caDNAno v0.1.

Table S12 | Individual staple sequences for sc_EGFP1 20HB-LP.

Name	Sequence 5' - 3'
core_1	GCGTTTTTAATTCGCGTTAAATTTTATAAATAGCAGAA
core_2	CAATGGGCTCTTCCTTAAAGAGTTGGGAGGGCACTCGCCCTCGCCCTCGCCGGA
core_3	ATTTACCAAGTCCCTATTGGCGTTGACGTC
core_4	AAGGCCAGTAATGCCAGGCGGGCCCAAGTAGGGGTCTCCC
core_5	CGTCATTATTGACGTGTATTGATTTTGGTGCCAAAACAAACTCCCATTACTATG
core_6	ATATGATACACTTGATGTACTGCGATGTAC
core_7	CGTCAATAGGGGGCGTATAGCGAATAAGCCAGTAAGCA
core_8	CCCGTGAGACCGTACACGCCTACCTCCAGCTC
core_9	TCAAACCGCTATCCACGCCCATTCAAGTGGGAAAAAAAG
core_10	CGACATTTTGATCCGGAACATATTGGGCGGTCAGCCAGCTCTCCT
core_11	TGGGGCGGAGTTGTTACACGCTGACCGTCGTGTACCGCGA
core_12	GCGTCAAAATGGGGTGGAGACTTGGAAATCCATTGACGTTTTTGTTAAAATGCC
core_13	GCCAGAGAGCTCTGCTGAACAGCATCAGTGGCCGAGCG
core_14	TCTAGTTATGCCAAAACCGCATCACCATGGTAACTTGGCTTTTCTACAACTGATTCTTGCCC
core_15	TATAGTGAATTCCAGAAGTTTTGGAAGCTTCGTCGTT
core_16	CCGTTTACGTCGCCGGCCCATTTCGGTGGTTTCAATGGA
core_17	TGGGCACCACCCCGGTTATATAGACGCGCAGCAGTTTACCGTAAATACTCCACC
core_18	CCTTGCTCACCATCTCGTGGGTTCCTTTGATC
core_19	GAGCGGCCGTAAACTTCCGCCTCCGTTCCCA
core_20	AGCGGCTGAACTTTATGGTCTGACGAGTTGCCTTCAGCA
core_21	GTGCTGCTTGATGGATATCTGCAGAGTCGTAT
core_22	TGTGGCTTCACCTTGATGCCGTTGGGGCAAAAGTGTTATAATCGGA
core_23	GTTGTAGTTGTACTCCAGCTTGTAGCTTGCCGCTCCAGATTCATCCA
core_24	GCCCCAGGCCGTCCTCGATGTTGTGGCACAGTCTCCGATCAGAGCTTG
core_25	ATGTTGCCGTCCTCCTTGAAGTCGGTGGTCACCGGAAGG
core_26	GATGCCCTCTGCTGGTAGTGGTCGCATTTAGG
core_27	GGGTGTTTCAGCTCGATGCGGTTCACCAGG
core_28	CAGCACGGGGCCGTCTAGATTAACGATCAAGGGCGCTA
core_29	TCGGGCAGCTCGAACTTCACCTCGGCGCGGGAAGAAGTCTGTTGCCGAAATCAA
core_30	ACTGGGTGCTCAGGTAGTGATCTGGTATGACGCTGCG
core_31	GGCTGGCAACTAGAAGGCGGATCTCACCGGGTGGTG
core_32	GATCAGCGAGCTCTAGGCGAGCTGCAGCCAGCGAGGGT
core_33	CTTGTACAGCTCGTCCATGCCGAGAGTGGTTGCTATTAAT
core_34	CCGGCGGCGGTCACGAACTCCAGTCTTTGCT
core_35	AAGCACTACACTCATGGTTACTA
core_36	CCGATTTGTTGTCAGCCGTAAGATAGTTGCCCTGGTAG
core_37	AACGTGGAAAAGCGGCTCAACCATCTCAGCGCAGATTACCTCCC

core_38	TCACCCTAATCAAGTTTTATTGAACTCATACGGGGGTCG
core_39	CTATCAGGGCGATGGCATGAGCGGGGAATAA
core_40	TTTGGAACAAGAGTCTTCCCCGATCTTTTAC
core_41	TGTTCCAGGGGCGCTGGCAAGTGTAGCGGTCGCTTCATT
core_42	TAGACCGAGATAGGGGTTAATATACCCACTCGTGCACCC
core_43	AATCGGCAAAATCCCTTTGTTAATCTTACCG
core_44	TCAGGGTTATTGTCTCCCACTACCATGCCATAAGTAAGGACCCACGCTTGAAGT
core_45	TTTGAATGTATTTAGAAGGGCGAAGGTGAGTATTAGCTCCAATAAACCACGCTG
core_46	GGGTTCCGCGCACATCACTATTAAGTGTATGACATGATCCAGAAGT
core_47	AAAGTGCCAAAAATCGACGCTCAAGTCAGAGGTGGCGAA
core_48	ACCTAAATTGTAAGCTTGAGTGTGGCGTCACAGCTCCGATCCAGT
core_49	GGAAGGCTGCAAGCAGATCTGTCGACCAGGAGGGCCAGGGCACGGGC
core_50	GCGTTTCCTGAGAATAAGAACGGAAGGGAAGAAAGCGAAAGGAGC
core_51	TTTCACCAGATCTCAAATGCTTATCCTCGCCACGCCGTAGGTCAG
core_52	TGCTTTTGCAAAAAAGATACATATCCCCCTGGAAGCTCCCTCGTGCGGCGGGCCGGGCGAC
core_53	CTGTGACTAAACCGTACGGGGAAAGCCGGCGCGATGCA
core_54	ATACGGGATCTAAAGTGACGCTCATAATTTCGTGACTAATACGTAGATGTACTGCATTTACC
core_55	TAATACCGCGATGTATTTGTTAACCATAGGCTCCGCCCCCCTGACGAGCATCAC
core_56	TTGGCCGCCAACAGATGAGTGGCACCTTCCAGGGTCAAG
core_57	TTGTGCAACGAGAAAGTGGACTCCAACGTCAAAAAATAACAAAAACA
core_58	CCCCATGTGACACTATAGAATAGGGCCCTCGCCGATGGGGTCCTGCAAGCACTG
core_59	GGCGAGTTCGGCGACCAGTTACCAGAAGATC
core_60	CATCGTGGCTTTAAAATTTTAAAGGATTTTG
core_61	TAGATAACTAATTCTGTTGAATAGCATTTAGTTCCGACCCTGCCGCTTACCCT
core_62	TATTTCGTTTATCAGCTTCGGTCCGAGGCTATTTCCTCATTTTATTAGGAAAGG
core_63	AGGCACCTAAGTCATTTGGGTGAGACAAATAGACCCGACAGGACTATAAAGATACCAGGCGTT
core_64	TTAAAAATGCACACTGGGCATGGCGGACTTGTCTTGTAG
core_65	GGCAAACAACGGAAATCTTACTGTGTGAACCAACCCTAAAGGGAGCCCACAGTGG
core_66	AACCACCGTGACTCCACTTGTGGCAGATGAACTTCAGGG
core_67	GGGGTCTATATATGAGCCAGTGTCATGTGGTCGGGGTGTGTCGCC
core_68	GTGGAACGGTCATTGACGCGTATATCAATGA
core_69	AAAACTCATCCAGTTCGCCACATCAAAAGAACGTAACCACCACACC
polyT_1	TTTTTGGAACCGTAAAAAGGCCGCGTTGCTGGTGAGCAACTGTTGAGACGTTAAG
polyT_2	TTTTTGCGTGGCGCTTTCTCATTCTCCCGGCGGTGTTTCAATATTTTGGG
polyT_3	TTTTTGTATCTCAGTTCGGTTAGCTCA
polyT_4	CAAGCTTGAAAGTCCCATAAGGTCATTTTTTTTTTTGTACTGGGCACAAAAGGCCATTTTT
polyT_5	TTTTTCGAGCTCGGTACGTCATGAGATTATCAAAATTTTT
polyT_6	TTTTTTTTTTGGGCTGTGTGCTTATCTGCGCTCTGCTGATTTTT
polyT_7	TTTTTGCTCCTGGACGTCAGTTAATCTAGATCC
polyT_8	TTTTTTTTTTGACCGCTGCGCCTTATCGGCCATGATATAGACGTTCAGCTTGCAATGA
polyT_9	TTTTTCGCTTCTCGTTGGGGCAGGACCACTACAGGTTAATGTC
polyT_10	TTTTTCTTGAGTCCAACCCGGGGCACGGGGGACTTCTGC
polyT_11	TTTTTTCAGAAGCCATAGAGCCCACCGCATCCGCCGCGC
polyT_12	TTTTTGCAGCAGCCACTGGTAGAGGTGCCGTAGAATAAGACACGACTTATCGCCACTGTTTTT
polyT_13	TTTTTCAATAGGCCGAACATTCCGCCTTTTT
polyT_14	TTTTTTTCGGGGCGAAAACTCCATTGGAAAACGTTCTTTTT
polyT_15	TTTTTGAAGTGGTGGCCGTCACAGGATTAGCAGAGTTTTTTTTTTCGAGGTATGTATTCGGGAATTTTT
polyT_16	TAATGGCAGCACTGCATACGATACGGTAGCTCTTGGAAAGTCCCGGGTCGTTCGCTCCAAGTTTTT
polyT_17	GTGCTCATTCAAGGAATCAGCTCATTTTTTAACTTTTT
polyT_18	TGTCACGCAGAGTAAGCAGGGCGGTTGCCGTCGTCCTTGAAGAAGATGGTGCTTTTT
polyT_19	TTTTTGTTGTTGCCATTGTGTGATCGTTTTT
polyT_20	TTTTTACAGTATTTGGTACCCGGCTACACTAGAAGATTTTT
polyT_21	ATCTGGCCTTGTCCGGTAACTATCGTTTTTT
polyT_22	CCAGTGCTGCCGTAGGTGGCAGAACCCCCCGTTCAGCTTTTT
polyT_23	TAGTTCGCAGCCTTCGGCGGCCGTTACTAGTGGATCTTTTT
polyT_24	TTTTTAGGATCTTCACAGTTTGCGCAACTTTTT
polyT_25	TTTTTAGCCAGTTACCGCTGTAGTTTTT
polyT_26	CTTCGGAACTACAGAGTTCTTTTTTT
LP_1	GTAAGTTATGTAACGCGGAACTCCATATATGGGCTATGAACTAATGACCCCGTAATTGATTACTATTAATAA CTAGTCAATAATCAATGTCAA
LP_2	CCCAGCATGCCTGCTATTGTCTTCCCAATCCTCCCCCTTGCTGTCCTGCCCCACCCCACCCCCCAGAATA GAATGACACCTACTCAGACAATG

2.2.1.7 sc_EGFP1 20HB-LPv2

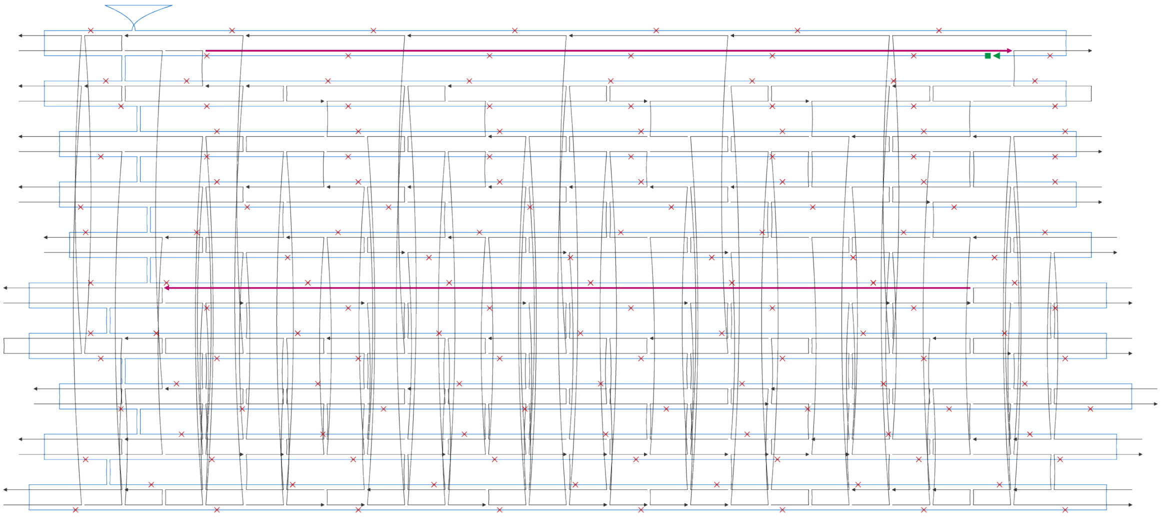

Fig. S26 | Scaffold routing and staple design for sc_EGFP1 20HB-LPv2. Scaffold routing is shown in blue, staples are given in black, breakpoint for addition of sequence is marked by a green square (5') and arrow (3'). Long 154-mer 'primer' sequences at the 5' promoter and 3' polyA regions flanking the EGFP gene are given in pink. Design was prepared using caDNAno v0.1.

Table S13 | Individual staple sequences for sc_EGFP1 20HB-LPv2.

Name	Sequence 5' - 3'
core_1	GGAACATAACGGAAATGCTGAAGCCGTCGTG
core_2	CGTCATTAATTGGCGTTACTATGCGCTTACGACATTTTGGAAAGTCCCGTTGATTCAATGGA
core_3	CGTCAATAGGGGGCGTACTTGGCTCAAACC
core_4	CCCATAAGGTCATGTATGACTAATATCCGAGCTCGGTACGCCAGTGCTAGATCC
core_5	AATGGGGTAGCTCTGCTTATATAGACTTGTGGGGCACCTGACCCACG
core_6	GGAGACTTGGAAATCCCCGTGAGATATGATATGCAAGCA
core_7	CGCCTACCGCCCATTTGCATCGCCTTCATCCAATCTGGCC
core_8	CCGTACACAAAACAAACTCCCATTGACGTCCAGTTTACAACCACCGTGGGTGAG
core_9	GTAAGCAGTGGGTTCGACCAGGAGGTCTGACAATAAAC
core_10	ATAAGCCAGCTATCCACGCCCATTGATGTACTTCATTGAGATCTCAACGATGTAAGTGTATG
core_11	TATAGTGAGTCGTATTCCTCGCCAAATCAATCAGAAGTG
core_12	TTCGGGTCAGCTTGCCGTAGGTGGCGTCAATTCGGAAAAGTCCCT
core_13	CGCCGGACACGCTGAACCTCCCAGGCAAACACGTAAATACTCCACCCATTGACGTTTGGTGC
core_14	GTCGCCGTCCAGCTCTCTAGTTATTTTTGTTCACTTGATGTACTGCCAAGTGGG
core_15	CACCCCGGTGAACAGCTAATTTCGAAAAAAAG
core_16	TGATGGATTGCGCTCCTGGACGTCTCGAACT
core_17	GGCATGGCACCATCTCGAGCGGCCCAAGCTTG
core_18	CTTGTCGGATGTTGTGGCGGATCAGGGTCAAGTTGTCAGGGTCGAG
core_19	GCCATGATATAGACGTTGTGGCTCGAGGGTGTACCGCGAATCTCAGC
core_20	GTTGTAGTAGTGGTCGGCGAGCTGCAACAGAT
core_21	TGTACTCCAGCTTGTGCCCCAGGAAGCACTGTTTATCAG
core_22	ATGTTGCCGGGCCGTCGCCGATGGCGAGGCTG
core_23	TCAGCTCGCTCAGGTAGTGGTTGTGACACT
core_24	CAGCACGGTCCTCCTTGAAGTCGATGCCCTGTGCTGCTGCCGAGCG
core_25	ACTGGGTGATGCGGTTCACCAGGGTGTCGCCAGCCTTCGCCGCCTCCTTTTAAA
core_26	GTTGGGGTCTTTGCTTTGTACAATCGTGGGGGCGCTG
core_27	TTTTTTGGAAGTAAGTTGGCGAG
core_28	CGTAACCATCATTTTTTAACCAATTTGTTAA
core_29	GCGATGGCCCACTACGCTCATACTGGGCGAC
core_30	TAGACCGAGAAAGCGAAAGGAGCGGGCGCTATGTCACGC
core_31	AAATCCCTTATAAATAAAGTGCCCTGTTGAGATCCAGTT
core_32	TTTCAATATTATTGAAAAACCGTGCACTGCATTCGGTCCCAGTGCTCCGTCCTC
core_33	GCATTTAGGAAGGCAAAATGCCGGTAGCTC
core_34	ATTGTCTCATGAGCGGAAGAACGTCCGTAAGACCCCATGCTCACCGGTGCTGGT
core_35	ATACATATTTCACCAGCGTTTCCTGGTAGC
core_36	TATTTAGAAAAATAATGTTCCAGCTCAACCAGTTCCCAACAGCCAG

core_37	ACAAATAGGTGCACCCAACTGATCGCAGATT
core_38	CGCACATTTCCCCGACAAAAGAACGGCGACTCGTCGTTGTCCTGC
core_39	TTCAGCAGGTGAGTATTTGGAAAGCCGGCGAACGTGGCGAGAAAG
core_40	TTCGGGGCGAAAACTCGACGCTCAATCTTCAC
core_41	TTACTGTCAAAAACATCAGGGTTGGACTATAAAGATACCAGGCGTTTCCCCCTG
core_42	CATGCCATGGACTCCGGGAGCCCCCGATTTAGAGCTTG
core_43	CGAGTTGCTTAAAAATCTTTGATCGGTCTCCCCCGCATCACCATGGTAATAGCGACTGGGCA
core_44	TCTTGCCCCTTACCGACCTAAATAAGGCCGCGTTGCTGGCGTTTTTCCATAGGC
core_45	TAATACCGAAAAAGGGTGGAACGACTAGTGG
core_46	CTCCGATCGGAAGGCACGGGGGAGGGGCAAACACGCTGGCAATGAGGCCAGG
core_47	TTAGCTCCTAATTCTCGATCTGTCTTGATCC
core_48	AAAAGCGGACCCTAAAAACGTCAAAGGGCGAA
core_49	TTGTGCAAGGCTGGCAACTAGAAGGCACAGTGGGTGTTCCTCCAGACACGCCG
core_50	ACATGATCTGCTTTTAATGCTTAGGTGGTTGCCAGAG
core_51	GGCGAGTTACGGGGAACAAGAGTCCACTATTA
core_52	CGATCAAATCAGCGAGCTCTAGCATTTAGGTCGGGCAGCCGGAAGGTCATGTGG
core_53	CAGCTCCGAGTCATTATATATGAACGCGCAG
core_54	GCTTCATTGAAGGGAAGATAGGGTTGAGTGT
core_55	TGGTATGATAGAATAGGGCCCTCTAGATTACCAGGGCGGAACTTTAT
core_56	ATCCAGTCTACAGGCGCTCGTCCATGCCGAGAGTGATCTGTGATCG
core_57	TAGTTGCCCTCATGGGGGAATAACTTCCTTGAAGCTCCCTCGTGCGCTCTCCC
core_58	TATTTCGCTCGCCCTGCACGGGCAGCTTGCC
core_59	ATCAGTGACCGTTTACTAGGTCAGGGTGGTCA
core_60	CAGTTACCCTGTGACTTCTTTTACTTTGAATGACGCTCAAGTCAGAGGTGGCGAAACCCGACA
core_61	GTAAACTTTGGGCACTCGGGGTAGCGGCTG
core_62	CTAAAGTCTGAGAATACCCACTCGGGTTCCGTCCGCCCCCCTGACGAGCATCACAAAAATCG
core_63	AAAGAGTTGCAAAAAATTATGGCACTATCAGGGTGCCGTAAAGCACTAAATCGGA
core_64	GAAGATCGAAGTTTTCTTGCTCGGACTTGAAGAAGTC
core_65	TTTTCTACTAATGCCAGGCGGGCCATTTACCGGCCAAAA
core_66	GGGGTCTTCAAGGATGGCGTCAAAATCGGCAGCAAGTGTAGCGGTC
polyT_1	TTTTTAGCAAAAGGCCAGCAAAAGGCCAGGAACCGTAAATGTAAGCGTTAATATTAGGCCGA
polyT_2	TTTTTGTCCGCCTTTCTCCCTGCTTACCGGATTAGGTTGAATATGAACCA
polyT_3	TTTTTCTTTCTCATAGCTCATCGGGAA
polyT_4	ACGTAGATGTACTGCCAATTTTTTTTTTTGTAGGAAAGTTCAATGAGTGTTTTT
polyT_5	TTTTTCTGGCGGCCGTTAAAACTCACGTTAAGGGATTTTT
polyT_6	TTTTTTTTTTGTGTAGGTCGTAGACACTAGAAGAACAGTTTTTT
polyT_7	TTTTTGTCGTCCTTGAAGGAAGCTAAGATTATC
polyT_8	TTTTTTTTTTTGCACGAACCCCCCGCCTTGATGCCGTTCTTCTGGGTGGTGGCTTACC
polyT_9	TTTTTACTCCAGCAGGACCACCGGCGGCTTTGCGC
polyT_10	TTTTTGCGCCTTATCCGGTAATGGCACCTTCCTTGAAGT
polyT_11	TTTTTCGCGCTTAATGTCACATTCCGCCTCAACGCTGCGAACGTTGT
polyT_12	TTTTTTAAGACACGACTTATCCCCTAATCAAGGAGCTATCGTCTTGAGTCCAACCCGGTTTTT
polyT_13	TTTTTGTTAAATCAGCCCACACCCGCTTTTT
polyT_14	TTTTTGTGCTCATCATTGGAAAGCAGAACTTTAAAATTTTT
polyT_15	TTTTTGTAGGCGGTGCTTCAGCCACTGGCAGCAGCTTTTTTTTTTCACTGGTAACAGGATACCTTTTTT
polyT_16	ACACGCAGTGTTATCATGACTCCCCAGTTACCTGGGGCGGAGTTGTGTAGGTATCTCAGTTTTTTT
polyT_17	TACGGGATGCCATTGCTATTAATCGCTTCTCTCACCTCGGCGCGGGTCTTGTAGTTGCCTTTTT
polyT_18	CGCCACATAACGTTCAATTCGCGTTAAATTTTTTTTTT
polyT_19	TTTTTCGCCAGTTAATAGGGTCACGATTTTT
polyT_20	TTTTTCTAACTACGGCTATATTCTTGAAGTGGTGGCTTTTT
polyT_21	ACTACGATTCATTCAGCCCGACCGCTTTTTT
polyT_22	ACGGGAGGCAGATGAACTTCAGCTCCAAGCTGGGCTGTTTTT
polyT_23	TGTTGCCGGAAGATGGATCTGCAGAATTCCAGCACATTTTT
polyT_24	TTTTTTTTTGGTCATGGAGTAAGTAGTTTTTTT
polyT_25	TTTTTATTTGGTATCGCGTGGCGTTTTT
polyT_26	TGCGCTCTCAGAGCGAGGTATTTTTT
LPv2_1	TTGACGTCAATGGGCGGGGGTCGTTGGGCGGTCAGCCAGGCGGGCCATTTACCGTAAGTTATGTAACGC GGAACTCCATATATGGGCTATGAACTAATGACCCCGTAATTGATTACTATTAATAACTAGTCAATAATCAAT GTCAACGCGTATA
LPv2_2	GAAGCCATAGAGCCCACCGCATCCCCAGCATGCCTGCTATTGTCTTCCCAATCCTCCCCCTTGCTGTCCT GCCCCACCCCACCCCCCAGAATAGAATGACACCTACTCAGACAATGCGATGCAATTTCCTCATTTTATTAG GAAAGGACAGTGG

2.2.1.8 sc_EGFP1 20HB-Circ

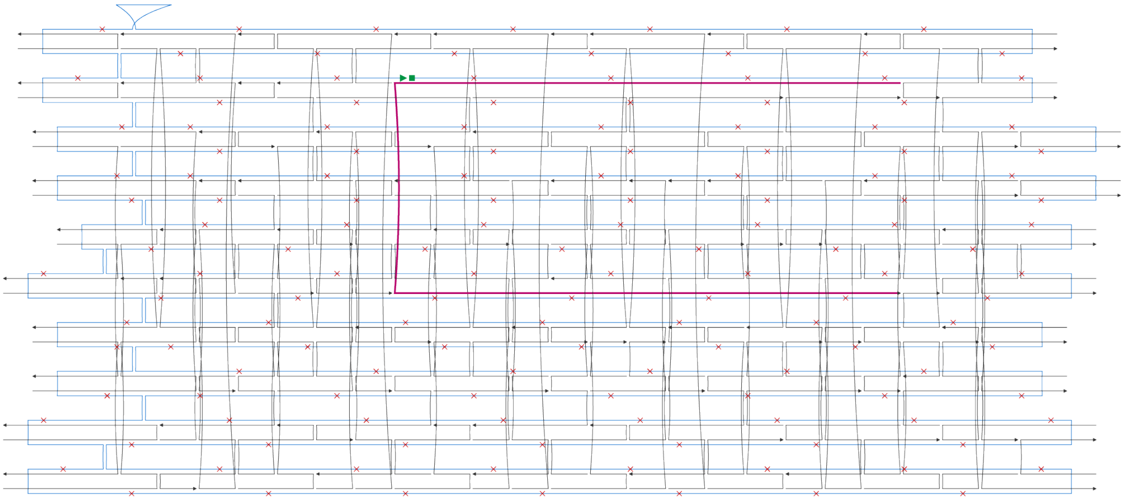

Fig. S27 I Scaffold routing and staple design for sc_EGFP1 20HB-Circ. Scaffold routing is shown in blue, staples are given in black, breakpoint for addition of sequence is marked by a green square (5') and arrow (3'). Long 200-mer 'primer' sequence linking the 5' promoter and 3' polyA regions flanking the EGFP gene is given in pink. Design was prepared using caDNAno v0.1.

Table S14 I Individual staple sequences for sc_EGFP1 20HB-Circ.

Name	Sequence 5' - 3'
core_1	TCAGAGGGGCGAGAAAGGAAGGATAAATCA
core_2	AGATACCTTAGAGCTTGACGGGGGAGTGTT
core_3	TACCTGTCAGGCGGGCCATTTACCTGACGTC
core_4	ATCAATGAAAAATCGACGCTCAAGCTTATCCG
core_5	TACGGCCATTTACCGTCATTGACGTCAATAGGCAAAAGG
core_6	GGGGCGTATCCCATAAGGTCATGTTGGGTCTC
core_7	CTTGGCATATGATACACTTGATGTACTGCCAAATACGTAGATGTACCGATAAGC
core_8	AGTGGGCAGTTTACCGTAAATACTCCACCCAAAACCGCATCACCATTAGCCAGAAGTCGAGG
core_9	ACCGCTATCCACGCCCACCGTACAAACAACAGATGGGGG
core_10	GGGTGGAGACTTGGAATGGGGCGCCAGGGTAGCTGCACGAAGCATTAAAAGGG
core_11	TCTTCCGAGCTCGGTACCAAGCTACTGGGCA
core_12	CTTATATAGACCTCCCATTGATGTACTGCCATTGACGTCAATGGAAAGTCCCTATTGGCGT
core_13	ATATCTGGAGTCGTATTAATTTTGCCAAGTTTCCGCCT
core_14	TCACCATCAGTGGGTTCTCTAGTGGTAATAGCGATGACT
core_15	TACGTCGCCCGCCCATTTGCGTCAAATCCCCGTGAGTCAATACTATGGGAACATACGTCATTAT
core_16	GTGCGCTCCCTCGGCGCGGGTCTACTCTCA
core_17	CTGGACGTAGCCTTCGGGCATGGTGTGATGGGGGGTCTTCTATAGAAAATCCCTTGAAGAAAG
core_18	CGGACTTGCGGTTCACCAGGGTGTCAGTTCGA
core_19	AAGAAGTCGTGCTGCTTCATGTGGCCCTTGCAGGTAGTGCTGATCAGATAGGGTTAAAGCCG
core_20	GTCGGGGTCTCCTTGAAGTCGATGTGATCTTC
core_21	TAGGTCAGACTCCAGCTTGTGCCCTTTCTGGG
core_22	GCACGGGCTGATATAGACGTTGTTGCCGCAA
core_23	TAGTTGTGGTGGTCACGAGGGTGGGCCAGGTGGCCGTTGTCGGCGCAAGGAAGCGTCAAA
core_24	CCAGCAGGCAGCTCGCATTTTTTCTGGCAAGAATTAAAAGACACGACTTATCGCCACTGGGTA
core_25	GTTGTCGAAACAAATAGCATCTTACCGAGTTGCTCTTGCCCGGCGT
core_26	CGTCGCCGATGGCTGAGAGTCCAAAGGGAG
core_27	GCTGCCGTGTCGGCCAAGCTTGCCGGTGGTGCAGATGAA
core_28	CCTCGATCCTCGCCGTACGACATTTTGGAAA
core_29	TACTTGTAACCATGTGATTTTGTAGGATCTTAAGTGCTCATCATTGGAAAACGT
core_30	CTCTAGATCCTATAGTCAGAATTCCAGCACAC
core_31	AGGTGACATGCTCAGGAAAAGTGTGTAACCCGATAATACCGCGCCACATAGCAGGGTATGGC
core_32	GCAACTAGAAGGCACGAGCTCTGGGTGAACAGCTCCTC
core_33	GCACGGGGGAGGGGCACGCCTACGTCCAGCTCGACCA
core_34	GGCACCTTGAGTTGTGACACGCTGAACTTG
core_35	AACCACCACCAGGAACATAGGCTCCGCCCCCCCGGTAA
core_36	CACCCGCCTAGGGCGAACCAATAAGTAGTTCTCACGCTCGTCGTTTAACTTTAA
core_37	TGTAGCGGGTTTTTCCCGTAAAAAGGCCGCG

core_38	GAACCCTACTATTAAAACCAGCCTAGCTCCTTCGGTCCT
core_39	AGGTGCCGGCCTGACTGCTGTAGGTATCTCAGTTCGGTGCTCGTGCGCTAAATCG
core_40	TGGGGTCGGGGCGAAAACGCTCACAGTAAGTTGGCCGCAG
core_41	TAAAATTCTCGTGGTGGCCAGTTAATAGTTTTCCTTTTA
core_42	AATTGTAAATCGGCAATAGGGCCATGTCACAAGGAAAG
core_43	CCACCTATTCATTCAATTGTTGCCGGGAAG
core_44	AGGGGTTCGAGTTAGCAACTTTATCCGCC
core_45	AGAAAAATGGCAGCAGTGCCGTCAGCGGCTGAAGCACTGCACGCCGGGATGGGC
core_46	TATTTGAAAAGCGGTAGCCGGAAGGGCCGA
core_47	CGGATACATGAGCAAACCAAGTCATTCTGAGAATAGTGTGTGCAAAATGTATTTTTGGAACA
core_48	TATCAGGGTTGTCAGACGGCTCCAGATTTATC
core_49	ACCGCTGTTGAGATCCGCCCTCTTCTCGTT
core_50	ACTCGTGCACCCAACCCCTTCAGGGTGCTC
core_51	TTACTTTCACCAGCGCAGGATGTCACGGGGCACCACCCC
core_52	TACTCAAAACAGGAAGGCAAAAGGCTGTTGTGGTAGTG
core_53	AGATGCTTTTCTGTGACTGGTGAGCCGATCGTTATTGTGACTCCAA
core_54	ATCCGTAAATAAGGGCGACACGGTTCTGCTT
core_55	GATCAAGGCCGCGCACAGACCGAGCGAGCTCTAGCATTTCAGTAAGCTCGAGCGGCCGCCAG
core_56	GTCTATTAGCTCCGGTTCCCAACCAATACGG
core_57	GTGGTCCTCATGATCCCCCATGTTATGCGGCG
core_58	GCTGCAATGATACCGCACTACGACCTAATC
core_59	ATGAAGTTTTAAATCCTAGAGTAGGCCGAAGCGTTAATATCGCGCGAACTTCA
core_60	AATCTAAACGAAAGGACATCACAGTGAGCAAAAGGCCA
core_61	GTATATATGTAACTATCGTCTTGAGTCCAACCTGACGAGGCGGGCGCGCGCTTA
core_62	GAGTAAACTTGGTCTTCCATCCAAAAGAATATTTCCCCGGCGGACTGCTCGATG
core_63	GACAGTTAGCGAACGTTGGCGAAACCCGACAGGACTATAAACGAACCC
core_64	CCAATGCCCCGTTCAGCCCGACCGCTGCGC
core_65	TTAATCAGTGAGGCACGCGCAGAAGTTCCAGT
core_66	CTATCTCACCCCCGATAGGCGTTTCCCCCTGGAAGCTCCTAGGTCGT
core_67	GCGATCTTCGCTCCAAGCTGGGCTGTGTGC
core_68	GTCTATTTCGTTCATCAGCAATAAGAACGTGCTCATGAGTGTTCTGC
core_69	CATAGTTTAAAGCACTCTCCTGTTCCGACCCTGCCGCTAGCTCAC
core_70	CCCCGTCGTGTAGATAGAGACCCAACCGTCTTATTATT
polyT_1	TTTTTCCTTCGGGAAGCGTGGCGCTTTCTCATTACCGGAAAGTTTTTACACCTAC
polyT_2	TTTTTCGAGGTATGTAGGCGGACAGGATTAGCAGAGTTTTT
polyT_3	TTGCTGGCTCAGTTTGCAAGCAGCAGATTTTTT
polyT_4	TTTTTGCGGTCAGCCCGCCTTTCTCTTTTT
polyT_5	TTTTTGAAGTGGTGGCCTAACTGCTACAGAGTTCTTTTTTT
polyT_6	CCATTGATGCGATGCAATTTCCTCATTTTT
polyT_7	TTTTTGTGCCAAAACAAACTCGTCCCGTTGATTTTGTTTTT
polyT_8	TTTTTACAGTATTTGGTATCCTCCGGCAAACAAACCTTTTT
polyT_9	CCCTCGCGTTGTGGCAAAGGACAGATGGCCCACTATTTTT
polyT_10	TTTTTGCCGTAGGTGGCATCGCTTCAGGGTCAGCTTTTTTT
polyT_11	TTTTTCAGTTACCTTCGGAAAATCGTCCTTGAAGAAGATGTGGCGGCC
polyT_12	TTTTTTTCACCTTGATGCCGTTCAAATGTTGAATATTTTT
polyT_13	TTTTTCTTGACGGATCTCAAGAAGATTTTTT
polyT_14	CACGAACTGTTACTAGTGGAGCGCTCTGCTGAAGCTTTTT
polyT_15	GTGGGAGTTCAGACAACGTCAATGAATGGGCGGGGGTCGTTGGTTTTT
polyT_16	TTTTTTTTTATTAGGGGATCTTGAAGTTTTT
polyT_17	TTTTTACCGCTGGTAGCGGTGGTTTTTTTCGCGAGAGTGA
polyT_18	TGCGCGTTAATGCCAGGCGGGCTACACTAGAAGATTTTT
polyT_19	TTTTTCGTGAACCATCACTACGGGAGGGCTTACCATCTTTTT
polyT_20	TTTTTTACGCGCAGAAATTGGATTTTGGTCATGAGTTTTT
polyT_21	TTTTTCTCATACTCTTCCACTCATGG
polyT_22	TTTTTCCTTTGATCTTTTCTACTCGGGGCGAAATGTAGTTG
polyT_23	TTTTTCTGCATAATTCTCTTACTGTCATGCCTGTTATCTTTTTCAAATCAGGGC
polyT_24	TTTTTAAAACTCACGTTAAGGTTGCCATTGCTCTGGGGTCTGACGCTCAGTGGAACGTTTTT
polyT_25	TACAGGCAGCGTTAAATTTTTAGGCGGCGGTCCGAGAGTTGGTAGCTTTTTT
polyT_26	TTTTTTGGCCCCAGTTTATGGCAGCATTTTT
polyT_27	TTTTTATTATCAAAAAGGATCTTCACCTAGAGCGCAACGAAAGTTAAATCAGCTTCCATGCC
Circ_1	GTAAGTTATGTAACGCGGAACTCCATATATGGGCTATGAACTAATGACCCCGTAATTGATTACTATTAATAA CTAGTCAATAATCAATGTCAACGCGTATCAGAAGCCATAGAGCCCACCGCATCCCCAGCATGCCTGCTATT GTCTTCCCAATCCTCCCCCTTGCTGTCCTGCCCCACCCCACCCCCCAGAATAGAATG

2.2.2 sc_EGFP2

Scaffold sequence for sc_EGFP2:
gtcgggtttcgccacctctgacttgagcgtcgatttttgtgatgctcgtcaggggggcggagcctatggaaaaacgccagcaacgcggcctttta
cggttcctggccttttgctggccttttgctcactcattcgcctcagaagccatagagcccaccgcatccccagcatgcctgctattgtcttcccaatc
ctccccttgctgtcctgccccaccccaccccccagaatagaatgacacctactcagacaatgcgatgcaatttcctcattttattaggaaagga
cagtgggagtggcaccttccagggtcaaggaaggcacgggggaggggcaaacaacagatggctggcaactagaaggcacagtcgagg
ctgatcagcgagctctagcatttaggtgacactatagaatagggccctctagattacttgtacagctcgtccatgccgagagtgatcccggcggc
ggtcacgaactccagcaggaccatgtgatcgcgcttctcgttggggtctttgctcagggcggactgggtgctcaggtagtggttgtcgggcagc
agcacggggccgtcgccgatgggggtgttctgctggtagtggtcggcgagctgcacgctgccgtcctcgatgttgtggcggatcttgaagttcac
cttgatgccgttcttctgcttgtcggccatgatatagacgttgtggctgttgtagttgtactccagcttgtgccccaggatgttgccgtcctccttgaagt
cgatgcccttcagctcgatgcggttcaccagggtgtcgccctcgaacttcacctcggcgcgggtcttgtagttgccgtcgtccttgaagaagatgg
tgcgctcctggacgtagccttcgggcatggcggacttgaagaagtcgtgctgcttcatgtggtcggggtagcggctgaagcactgcacgccgta
ggtcagggtggtcacgagggtgggccagggcacgggcagcttgccggtggtgcagatgaacttcagggtcagcttgccgtaggtggcatcg
ccctcgccctcgccggacacgctgaacttgtggccgtttacgtcgccgtccagctcgaccaggatgggcaccaccccggtgaacagctcctc
gcccttgctcaccatctcgagcggccgccagtgtgatggatatctgcagaattccagcacactggcggccgttactagtggatccgagctcggt
accaagcttgggtctccctatagtgagtcgtattaatttcgataagccagtaagcagtgggttctctagttagccagagagctctgcttatatagac
ctcccaccgtacacgcctaccgcccatttgcgtcaatggggcggagttgttacgacattttggaaagtcccgttgattttggtgccaaaacaaact
cccattgacgtcaatggggtggagacttggaaatccccgtgagtcaaaccgctatccacgcccattgatgtactgccaaaaccgcatcaccat
ggtaatagcgatgactaatacgtagatgtactgccaagtaggaaagtcccataaggtcatgtactgggcataatgccaggcgggccatttacc
gtcattgacgtcaatagggggcgtacttggcatatgatacacttgatgtactgccaagtgggcagtttaccgtaaatactccacccattgacgtca
atggaaagtccctattggcgttactatgggaacatacgtcattattgacgtcaatgggcgggggtcgttgggcggtcagccaggcgggccattta
ccgtaagttatgtaacgcggaactccatatatgggctatgaactaatgaccccgtaattgattactattaataactagtcaataatcaatgtcaacg
cgtatatctgtgacattaagcgcggcgggtgtggtggttacgcgcagcgtgaccgctacacttgccagcgccctagcgcccgctcctttcgctttc
ttcccttcctttctcgccacgttcgccggctttccccgtcaagctctaaatcgggggctccctttagggttccgatttagtgctttacggcacctcgacc
ccaaaaaacttgattagggtgatggttcacgtagtgggccatcgccctgatagacggtttttcgccctttgacgttggagtccacgttctttaatagt
ggactcttgttccaaactggaacaacactcaaccctatctcggtctattcttttgatttataagggattttgccgatttcggcctattggttaaaaaatg
agctgatttaacaaaaatttaacgcgaattttaacaaaatattaacgcttacaatttaggtggcacttttcggggaaatgtgcgcggaacccctatt
tgtttatttttctaaatacattcaaatatgtatccgctcatgagacaataaccctgataaatgcttcaataatattgaaaaaggaagagtatgagtatt
caacatttccgtgtcgcccttattcccttttttgcggcattttgccttcctgtttttgctcacccagaaacgctggtgaaagtaaaagatgctgaagatc
agttgggtgcacgagtgggttacatcgaactggatctcaacagcggtaagatccttgagagttttcgccccgaagaacgttttccaatgatgagc
acttttaaagttctgctatgtggcgcggtattatcccgtattgacgccgggcaagagcaactcggtcgccgcatacactattctcagaatgacttgg
ttgagtactcaccagtcacagaaaagcatcttacggatggcatgacagtaagagaattatgcagtgctgccataaccatgagtgataacactg
cggccaacttacttctgacaacgatcggaggaccgaaggagctaaccgcttttttgcacaacatgggggatcatgtaactcgccttgatcgttgg
gaaccggagctgaatgaagccataccaaacgacgagcgtgacaccacgatgcctgtagcaatggcaacaacgttgcgcaaactattaact
ggcgaactacttactctagcttcccggcaacaattaatagactggatggaggcggataaagttgcaggaccacttctgcgctcggcccttccgg
ctggctggtttattgctgataaatctggagccggtgagcgtgggtctcgcggtatcattgcagcactggggccagatggtaagccctcccgtatc
gtagttatctacacgacggggagtcaggcaactatggatgaacgaaatagacagatcgctgagataggtgcctcactgattaagcattggtaa
ctgtcagaccaagtttactcatatatactttagattgatttaaaacttcatttttaatttaaaaggatctaggtgaagatcctttttgataatctcatgacc
aaaatcccttaacgtgagttttcgttccactgagcgtcagaccccgtagaaaagatcaaaggatcttcttgagatcctttttttctgcgcgtaatctgc
tgcttgcaaacaaaaaaaccaccgctaccagcggtggtttgtttgccggatcaagagctaccaactctttttccgaaggtaactggcttcagcag
agcgcagataccaaatactgttcttctagtgtagccgtagttaggccaccacttcaagaactctgtagcaccgcctacatacctcgctctgctaat
cctgttaccagtggctgctgccagtggcgataagtcgtgtcttaccgggttggactcaagacgatagttaccggataaggcgcagcggtcggg
ctgaacggggggttcgtgcacacagcccagcttggagcgaacgacctacaccgaactgagatacctacagcgtgagctatgagaaagcgc
cacgcttcccgaagggagaaaggcggacaggtatccggtaagcggcagggtcggaacaggagagcgcacgagggagcttccagggggg
aaacgcctggtatctttatagtcct

Corresponding DNA origami designs and staple lists:

2.2.2.1 sc_EGFP2 20HB

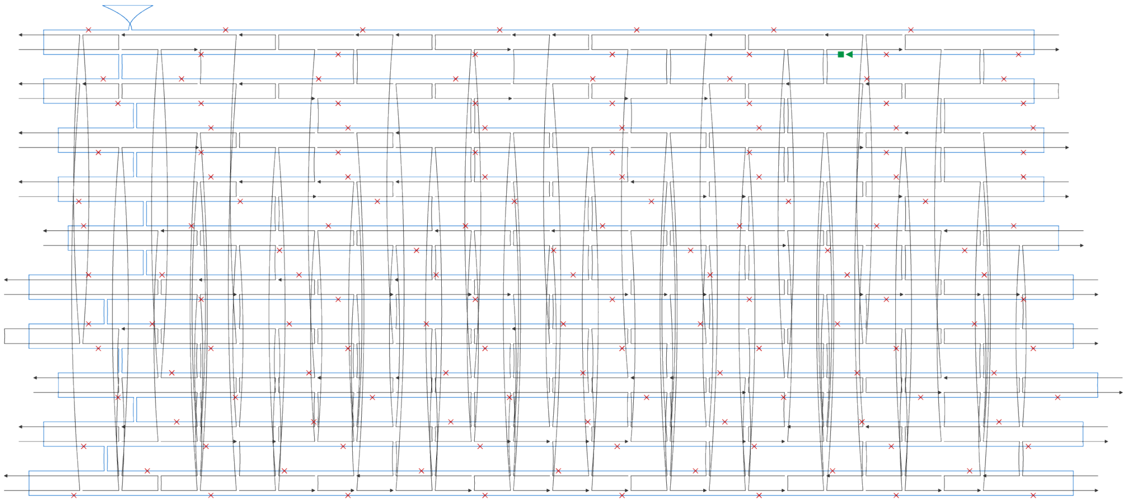

Fig. S28 I Scaffold routing and staple design for sc_EGFP2 20HB. Scaffold routing is shown in blue, staples are given in black, breakpoint for addition of sequence is marked by a green square (5') and arrow (3'). Design was prepared using caDNAno v0.1.

Table S15 I Individual staple sequences for sc_EGFP2 20HB.

Name	Sequence 5' - 3'
core_1	GAAGCTCCCTCGTGCGCTGAGTAGAAGGGCGCAAACCAC
core_2	GACTATAAAGATACCGGGTGGGGCAGGAAGTTTGCAAG
core_3	CCGCCCCCCTGACGAGTGGGCTTCGTGCACACGGGGT
core_4	CGTTTTTCAAAATTCGCGTTAAACCTTATAAATAGCAG
core_5	GTGTCATCCGTGCCTTCCTTGACCGACCAC
core_6	AATAGCAGAGCTCGCTGATCAGCTACCTGA
core_7	AGGCCAGCGCATGGACGAGCTGTATGCTGGAGGACACCCT
core_8	TCCCACTGTCCTTTCGTGAACATCGAGGACGGCAGCGTGCAGCTCGCCCTGGAA
core_9	CCCCTCCCTCTATTCTGGGGGGTGAGGCGTTT
core_10	AGTTGCCAGCCATCTCGACGGCCCCACAACGTCTATATC
core_11	TGCCTTCTGCAAGGGGGAGGATTGTGGCGAAA
core_12	AATGCTAGGCATGCTGGGGATGCGGCATCACA
core_13	CTATAGTGACCCCAAGCATCGACTTCAAGGTGCCCGAAGAGTAAACATCCGCCT
core_14	GCTGCCCGACAACCACCTCGACTGAGAAAAAA
core_15	GAGCAAAGTCACCTATCTTTTCTCCAACTGGCTCTTGC
core_16	AAGAACGGCATCAAGGGGCCCACCCCCCGTCGGATACCGC
core_17	CAAGCAGTACCAGCAGAACACCCCCATCGGGTTGTTTGTTTTTTTGGCAAAATG
core_18	GGGGCACAAGCTGGAATGAAGCATAATCAGGGGCCGAG
core_19	CGCATCGACAGTGGAATCTAGAGGTCTGAGGCGAATGAG
core_20	GGTGAACCTTCTTCATGAAGTTCGGGAAGGCTCGTCG
core_21	GTGCCCTTGAACTTTCTTGATGGTGCCACATTGCATCGCATTGTCTCTCCTG
core_22	CACCCTGACCTACGGATGGCCGAAGCGGTGG
core_23	TTCTTCAAGTCCGCCAAGGACGGCTCCTTTGA
core_24	AAGTTCACGCTACCCCGACCACGTACAACTTTACGCGC
core_25	CCCATCCTGCAACTTTTTGGTCTACCGAGTTATCTTCAG
core_26	ACGGTGGTCGTAACAACTCCGCCTTGACGTCGCAGTGTTTAAATCG
core_27	TATAAGCAGAGCTCTCGAGGGCGCGGCTCCACGTTCATC
core_28	CTGGCTAAGGCACCAAAATCAACGACTGCCCATCCTCCGATTAGAGCT
core_29	CTAGAGAACCCACTGCTTACTGGACGGCCACAGCCGGAA
core_30	AAGTCTCAATTAATACGACTCACTATAGGG
core_31	CGGTTTGACTCACGGTGACGGTCAACGATCGCGGGCGC
core_32	GTGGATAGGCTTGGTACCGAGCTCGGATCCACAAGGGCGATTGTTGCTTAAATC
core_33	GTTTTGGCAGTACATCAGTACATTTGGTATCACGCTG
core_34	GAGTATTTACGGTAAGGACTTTCGCTCACATGCCAC
core_35	ATTATGCCCAATGGGCGTCTATTAAGGAGCTGTCCAGGAGCGCACCAT

core_36	TGACCTTAAGTAATCAATTACGGGGTCATTATGTAGCGGTGGCTTCA
core_37	AACGCCATAAAGCACATCACTCATGGTAAC
core_38	CCGCCCATTGACGTCACCCCGATTCGTTGTCATCCGTAACATAGTTGCGCTGGT
core_39	ACCGCCCGTACATCAAGTGTATCATGGGAGTACCAGCC
core_40	CATAACTTCCCCTATTGACGTCAAGGATTTCCGTGGTCCTGGTCGAGC
core_41	CCACCACATTATTAATTGGGACTTTCCTACTTTACCATG
core_42	CATCACCCTAATCAAGTATTATTGTACTCATATGAGGAA
core_43	GTCTATCAGGGCGATGTCATGAGAAGGGAAT
core_44	CCAACGTCGAAAAATAGCAAAAACAGGACA
core_45	AGTTTGGAACAAGAGATTTCCCCCATCTTTT
core_46	GAAATCGGCAAAATCTTTTTGTTGATCTTAC
core_47	TATTTGAATGTATTTAAAAGGGCGCTGGTGAGGGTTAGCAGCAATAATTGTTTT
core_48	AGGGGTTCCGCGCACTCCACTATATAGTGTATTACATGACGCAGAA
core_49	GAAAAGTGAAAATCGACGCTCAAGTCAGAGGGGAAGACACTTTCACAGGATCT
core_50	CCACCTAAATTGTAAGGTTGAGTCCGGCGTTTCAGCTCCCATCCA
core_51	CAGCGTTTTCTGAGATAAAGAAAGGAAGGGAAGAAAG
core_52	TTCTGTGAAAAAACCTGACGGGGAAAGCCGG
core_53	CAATACGGAATCTAAACTGACGCTGCTGAAGGCGAGAAGCGCGATCACATGGTCCCAAGTAA
core_54	TCCCCCAAGTACGCACGGTAAATGGCCCGCCTGGCTGCGAACGT
core_55	CGGTTCCAAATGGCCCCCATATATGGAGTTCCGCGTTACGAAAGGA
core_56	GGCATCGTAACTTTAACCTTTTAAGGGATTT
core_57	TGTAGATACATAATTATGTTGAAAAGCATTTTCCGACCCTGCCGCTTACCGGC
core_58	CCTGACTCTCGTGACCTACGGCAAGCTGACC
core_59	GCGATCTGGCTTCAGCGCGTGTCCGGCGAGGG
core_60	GTATATATGGCTACGTTCACCGGGGTGGTGAGACCCAA
core_61	AATTAAAAAAGGACGAGCTCGAGATGGTGAGCTAGTAAC
core_62	CCGGCAAAACACGGAACTCTTACTACGTGAACGAACCCTAAAGGGAGCATAATGA
core_63	CTAATAAAACTCTTCCAAAAAGAGACGGGAGTGCCGGCAAGCTGCCC
core_64	CGTGCAGTTCTATTTGATTTATCTCCTTCGGCTTGGCAAACGACCC
core_65	TGACGTCAATATGCCATGTTGTGCGGCGAGAACGTGGACT
core_66	GTTGTTCCTAGGGCGCTGGCAAGGTTCATAGCGCCTGGC
core_67	AATAGACCGAGATAGGCGTTAATTAACCCACCTATGGCTGCCCTATT
core_68	TATCAGGGTTATTGTCGCCCACTGTCATGCCAGAAGTAGAGACCCACCAAAATGGAGGTCTA
core_69	GATGCTTCCGCAAAACGGATACACCCCCTG
core_70	GATAATACTTCGATGATTTTGTTCATAGGCT
core_71	AGTTGGCCAATGGGTGCGTATGTTCCCATAGT
core_72	AAAAAAGCTACTCAACTATCTCACAGCAGAACAACAGCCGTGCT
core_73	AAGGCGAGTGCGGCGGACAGTTACAAGAAGAAACATCCTGCACCCAGTCCGCCCT
core_74	TGAGGCACCCAAGTCATCTGGGTGAAACAAATCCCGACAG
core_75	CCAATGCTGCACGACTGGACGGCGACGTAACTTATCGACACCCCAT
core_76	CGAAAACAGATCCAGCGCGCCACATCAAAAGCGCGTAA
polyT_1	TTTTTGAACCGTAAAAAGGCCGCGTTGCTGGTGAGCAAACGCTGTTGTCACGTTA
polyT_2	TTTTTAAGCGTGGCGCTTTCTCTTTCTCTAGGCGGTTTTTCAATTTTTTG
polyT_3	TTTTTAGGTATCTCAGTTCGCATAGCT
polyT_4	TCGAGGGCTTCGTGACCGCCGCCGGGTTTTTTTTTATCACTCTCGAAAAGGCCAGTTTTT
polyT_5	TTTTTCCGAGGTGAAGTTGGTCATGAGATTATCAATTTTT
polyT_6	TTTTTAGCTGGGCTGTGGGCGTATCTGCGCTCTGCTTTTTT
polyT_7	TTTTTTATCCATCACACGCCAGTTAACCTAGAT
polyT_8	TTTTTGCCCGACCGCTGCGCCTAAATGGGCGGTAGGCGTGTCTGAAGTCTGCAAT
polyT_9	TTTTTTATTAGTCATCGCTATGGCAGTATGCTACAGCTTAATG
polyT_10	TTTTTGTCTTGAGTCCAACCCGGGACTTTCCACCATTGA
polyT_11	TTTTTACGCGTTGACATTGATTATTGACTAGCCCGCCGC
polyT_12	TTTTTTGGCAGCAGCCACTGGTCGAGGTGCCGATAGGTAAGACACGACTTATCGCCACTTTTT
polyT_13	TTTTTACCAATAGGCCTCACAGATATTTTTT
polyT_14	TTTTTTCTTCGGGGCGAAAACATCATTGGAAAACGTTTTTT
polyT_15	TTTTTTTGAAGTGGTGGGGGTAACAGGATTAGCAGTTTTTTTTTTAGCGAGGTATGCCTTCGGGTTTTT
polyT_16	CCTTATGGCAGCACTGACTACGATTTGGTAGCCAAGATCCGCCACTAGGTCGTTCGCTCCATTTTT
polyT_17	AAGTGCTCTCTCAAGAAATCAGCTCATTTTTTATTTTT
polyT_18	GGTGTCACCTAGAGTAGTGATGCGGGCCGCCAGTGTGCTGGAATTCTGCAGATTTTT
polyT_19	TTTTTACGTTGTTGCCATCATCTACGTTTTT
polyT_20	TTTTTGAACAGTATTTGTTATACGGCTACACTAGAATTTTT
polyT_21	CCATCTGGCGCTATCCGGTAACTATCTTTTT
polyT_22	CCCCAGTGTCATCTGCACCACACGAACCCCCCGTTCATTTTT
polyT_23	AGTAGTTCTGGCGGCCCGGCAACTACAAGACCCGCGTTTTT
polyT_24	TTTTTAAAGGATCTTCATAGTTTGCGCATTTTT
polyT_25	TTTTTGAAGCCAGTTCACGCTGTTTTTT
polyT_26	ACCTTCGGTGCTACAGAGTTCTTTTT

2.2.2.2 sc_EGFP2 20HB-LPv2

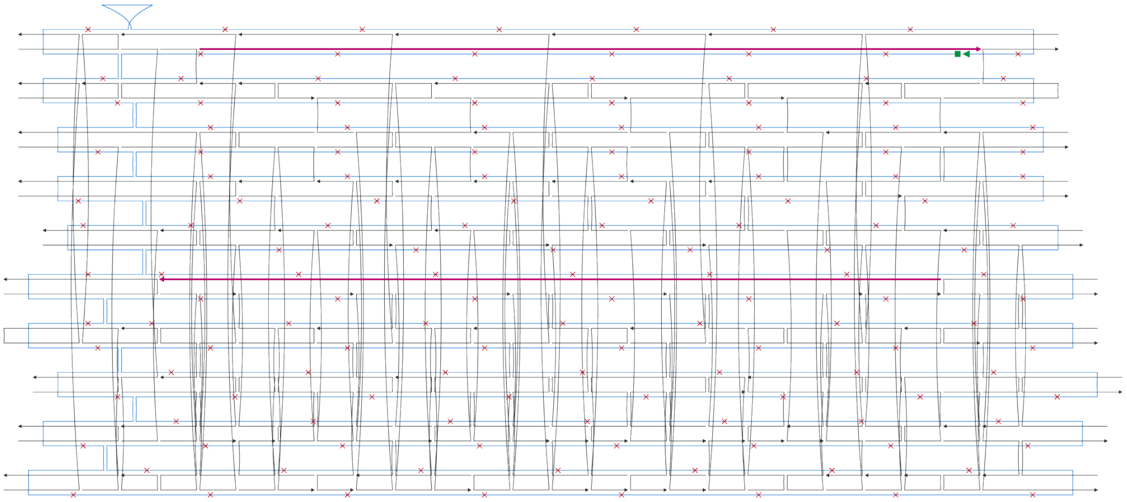

Fig. S29 l Scaffold routing and staple design for sc_EGFP2 20HB-LPv2. Scaffold routing is shown in blue, staples are given in black, breakpoint for addition of sequence is marked by a green square (5') and arrow (3'). Long 154-mer 'primer' sequences at the reverse compliment sequences for the polyA (5') and promoter (3') regions, flanking the EGFP gene are given in pink. Design was prepared using caDNAno v0.1.

Table S16 l Individual staple sequences for sc_EGFP2 20HB-LPv2.

Name	Sequence 5' - 3'
core_1	GGTGCCACACACGGAACTGCTGAACCCGTCG
core_2	TCCCACTGTCCTTGACCCTGGAACACAGGTGAACTTCAAGATCCGCCACAACATCCCCTCCC
core_3	GCCCTATTCTATAGTGTCACCTACCGTGCT
core_4	GACCGCCGCCGGGATCCGAGAAGCGCGCCGAGGTGAAGTGGCTACGACCTAGAT
core_5	TACCAGCAAAGCTGGAGTACAACTCTCGTGACGAGGCACGAGACCCA
core_6	GAACACCCCCATCGGCGACGGCCAATGCTAGTTTGCAAG
core_7	TCTATATCATGGCCGACACCGGCACGTTCATCCCATCTGG
core_8	CACAACGGCAGCGTGCAGCTCGCCGACCACTGCCTTCTCAAACCACTCTGGGTG
core_9	TTCAAGGAGGACGGCGCTTCAGCTTGGTCTAGCAATAA
core_10	GCATCGACGCTGCCCGACAACCACTACCTGAGCTAGAGGAGGATCTCTTCGATGATAGTGTA
core_11	GGTGAACCGCATCGAGCACGACTTTAAATCACGCAGAAG
core_12	CGTACCCTGAAGTTCATCTGCACCAAGCAGCCTTCGGCCGTGCCT
core_13	GTGCCCTGGCCCACCACAACAGCCCGGCAAAAGTTGCCAGCCATCTGTTGTTTGCGAGGACG
core_14	CCTACGGCGTGCAGTAACATCCTTTTTTTTGAGCTCGCTGATCAGCCTCGACTG
core_15	CCGACCACATGAAGCAGCTGAAGGAGAAAAAA
core_16	TCCAGGAGAGATATCCATCACACGCTTGGTA
core_17	GCTCGAGAGTCCGCCATGCCCGAATCGAGGGC
core_18	ACGCAAAAATCAACGGGACTTTCGTAACGCCTCGTTGTCGGGGTCG
core_19	TGGGCGGTAGGCGTGTACGGTGGAAGTTCAGGATACCGCCTATCTCA
core_20	GAGGTCTATGACGTCAATGGGAGTAATGGGTG
core_21	TATAAGCAGAGCTCTCTGGCTAAGGTCGAGCGATTTATC
core_22	CTAGAGAAACTCACGGGGATTTCCCTTGGCAG
core_23	AATTAATAAGTACATCAATGGGCAGTACGC
core_24	CGGTTTGCCCACTGCTTACTGGCTTATCGAAGGAGCTGGGGCCGAG
core_25	GTTTTGGCCGACTCACTATAGGGAGACCCAATGGCGGCCATCCGCCTCCTTTTA
core_26	ATCGCTATTACCATGAATGGCCGCATCGTTAGGGCGC
core_27	AGTTTTTTAGAAGTAAGTTGCAG
core_28	CGCGTAACGCTCATTTTTTAACCATTTTGTT
core_29	GGGCGATGGCCCACTATACTCATAAAGGGCG
core_30	AATAGACCAAGAAAGCGAAAGGAGCGGGCGCGGTGTCAC
core_31	CAAAATCCCTTATAAGAAAAGTGCGCTGTTGAGATCCAG
core_32	TTTTTCAATATTATTGAAAAACCCAGCACTGCCTTCGGCCCCAGTGGGCACCAA
core_33	AAGCATTCAGGAAGGCAAAATGTTGGTAGC
core_34	TTATTGTCTCATGAGCTAAAGAACATCCGTAATCCCCCACGCTCACCACCCCAT
core_35	GGATACAACTTTCACCAGCGTTCGCTGGTA
core_36	TGTATTTAGAAAAATGTTGTTCCTACTCAACCGGTTCCCACCAGCC

core_37	AAACAAATTCGTGCACCCAACTGACAGCAGA
core_38	CGCGCACATTTCCCCATCAAAAGTGCGGCGGCTCGTCGTGGTCCT
core_39	TCTTCAGCTGGTGAGAGTTTGGAAAGCCGGCGAACGTGGCGAGAA
core_40	TCTTCGGGGCGAAAACCTGACGCTGGATCTTC
core_41	TCTTACTAGCAAAAATATCAGGGGACTATAAAGATACCAGGCGTTTCCCCCTGG
core_42	GTCATGCCGTGGACTAAGGGAGCCCCCGATTTAGAGCT
core_43	ACCGAGTTAATTAAAATCCTTTGAGACACCCTTCCGCCCTGAGCAAAGACCCCAAACTCTCG
core_44	GCTCTTGCATCTTACCCACCTAAAGGCCGCGTTGCTGGCGTTTTTCCATAGGCT
core_45	GATAATACTCAAAAACAGTGGAACAAGACCC
core_46	TCCTCCGAAATAGGGACTTTCCATTGACGTCTTGTTTTCTGCAATCGTGTCC
core_47	GGTTAGCTCATAATTCGCGATCTGTCTTGAT
core_48	AAAAAAGCGAACCCTACCAACGTCAAAGGGCG
core_49	TGTTGTGCGAGTATTTACGGTAAACTGCCCAAAGTCTCCGGCTCCATGGACGG
core_50	TTACATGAGATGCTTCCAATGCTGCGGTGGGGGGCAC
core_51	AAGGCGAGTGACGGGGAACAAGAGTCCACTAT
core_52	AACGATCTACATCAAGTGTATCATATGCCAGTGGATAGAGCCGGAATTCACCGG
core_53	TTCAGCTCCAAGTCAGTATATATTTACGCGC
core_54	TGGCTTCAAGGAAGGGGAGATAGGGTTGAGT
core_55	TTTGGTACCCCTATTGACGTCAATGACGGTAGTGATGCGGCAACTTT
core_56	CCATCCATGCTACAGCGCCTGGCATTATGCCCAGTACACATCTACG
core_57	CATAGTTGCACTCATAAGGGAATCTCTTCCAAGCTCCCTCGTGCGCTCTCCTG
core_58	TCTATTTAGCTGCCCGGCGAGGGCGAGGGCG
core_59	TAATCAGTCACCCTGACGACGTAAACGGCCAC
core_60	GACAGTTATTCTGTGACATCTTTTTATTTGAACGCTCAAGTCAGAGGTGGCGAAACCCGACAG
core_61	GAGTAAACCGCTACCGGTGGTGCCCATCCT
core_62	ATCTAAATTCTGAGATAACCCACAGGGGTTCCCGCCCCCCTGACGAGCATCACAAAAATCGA
core_63	AAAAAGAGCCGCAAAAGGTTATGGGTCTATCAAGGTGCCGTAAAGCACTAAATCG
core_64	AAGAAGAATGAAGTTTCTTCAATGGTGAGCAAGGGCG
core_65	TCTTTTCTGCATGGACGAGCTGTACAAGTAATCACCCAG
core_66	ACGGGGTTCTCAAGGCCGGCGTCGAAATCGGTGGCAAGTGTAGCGG
polyT_1	TTTTTGCAAAAGGCCAGCAAAAGGCCAGGAACCGTAAAAATTGTAAGCGTTAATAATAGGCC
polyT_2	TTTTTCTGTCCGCCTTTCTCCCCGCTTACAGGATTATGTTGAACGTGAAC
polyT_3	TTTTTCGCTTTCTCATAGCTCTTCGGG
polyT_4	GCGATCACATGGTCCTGCTTTTTTTTTTTGGAGTTCGTGAATGAGTGATTTTT
polyT_5	TTTTTCGACGGCAACTACGAAAACTCACGTTAAGGTTTTT
polyT_6	TTTTTTTCGGTGTAGGTTGTCTACACTAGAAGAACATTTTT
polyT_7	TTTTTCCAGTGTGCTGGCGGGAAGCTGAGATTA
polyT_8	TTTTTTGTGTGCACGAACCCCCTAACAACTCCGCCCCATTGATGCCACGGGCTTA
polyT_9	TTTTTTCCTACTTGGCAGTATGACCTTAAGTTTGC
polyT_10	TTTTTCTGCGCCTTATCCGGTATGTTCCCATACAAAATG
polyT_11	TTTTTGCCGCGCTTAATGTCACAGATATACGTCACGCTGGCAACGTT
polyT_12	TTTTTGGTAAGACACGACTTACACCCTAATCACGTAACTATCGTCTTGAGTCCAACCCTTTTT
polyT_13	TTTTTTTGTTAAATCACACCACACCCTTTTT
polyT_14	TTTTTAAGTGCTCATCATTGGATAGCAGAACTTTAATTTTT
polyT_15	TTTTTATGTAGGCGGTGCATTCGCCACTGGCAGCATTTTTTTTTTGCCACTGGTAACCGGATACTTTTT
polyT_16	CTAGCCGCAGTGTTATCCTGACTCGCCAGTTAAAGAACGGCATCAGCTGTAGGTATCTCAGTTTTT
polyT_17	AATACGGGTTGCCATGTCTATTATATTAGTCCCGAGCTCGGATCCACTAGTAACGGCCGTTTTT
polyT_18	CGCGCCACAAAACGTAAAATTCGCGTTAAATTTTTTTT
polyT_19	TTTTTTTCGCCAGTTAATTGGGACTTTTTTT
polyT_20	TTTTTGCCTAACTACGGAGAAGTTCTTGAAGTGGTGTTTTT
polyT_21	TAACTACGTCGCGTTCAGCCCGACCGTTTTT
polyT_22	ATACGGGACTACGGCAAGCTGTCGCTCCAAGCTGGGCTTTTT
polyT_23	ATTGTTGCAATTCTGCCGCACCATCTTCTTCAAGGATTTTT
polyT_24	TTTTTGATTTTGGTCATAGAGTAAGTAGTTTTT
polyT_25	TTTTTGTATTTGGTAAAGCGTGGTTTTT
polyT_26	TCTGCGCTAGCAGAGCGAGGTTTTTT
LPv2_1	TCCTTTCCTAATAAAATGAGGAAATTGCATCGCATTGTCTGAGTAGGTGTCATTCTATTCTGGGGGGTGGG GTGGGGCAGGACAGCAAGGGGGAGGATTGGGAAGACAATAGCAGGCATGCTGGGGATGCGGTGGGCTC TATGGCTTCTGAGGC
LPv2_2	CGTTGACATTGATTATTGACTAGTTATTAATAGTAATCAATTACGGGGTCATTAGTTCATAGCCCATATATGG AGTTCCGCGTTACATAACTTACGGTAAATGGCCCGCCTGGCTGACCGCCCAACGACCCCCGCCCATTGAC GTCAATAATGA

2.2.2.3 sc_EGFP2 20HB-Circ

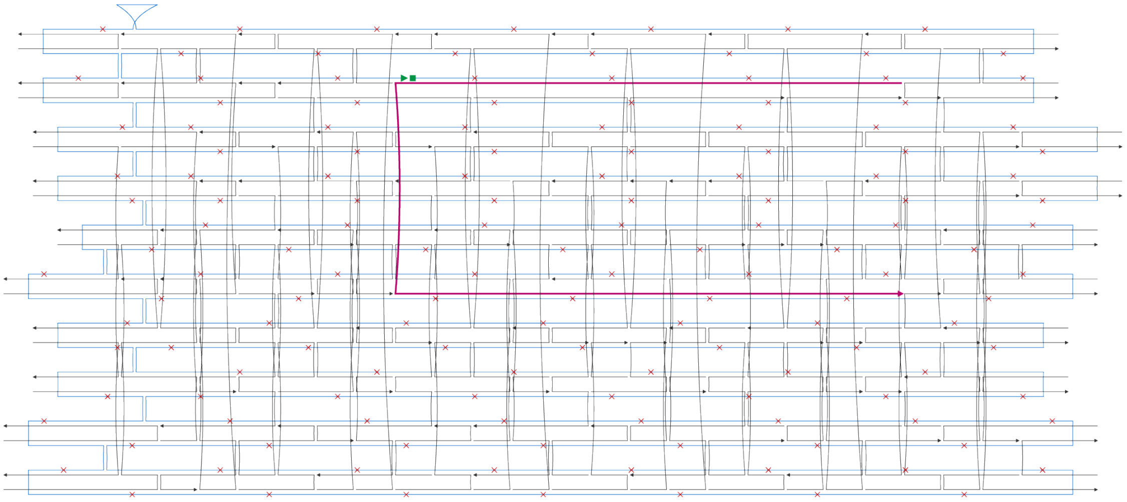

Fig. S 30 I Scaffold routing and staple design for sc_EGFP2 20HB-Circ. Scaffold routing is shown in blue, staples are given in black, breakpoint for addition of sequence is marked by a green square (5') and arrow (3'). Long 200-mer 'primer' sequence linking the reverse compliment sequences for the polyA (5') to the promoter (3') region, flanking the EGFP gene is given in pink. Design was prepared using caDNAno v0.1.

Table S17 I Individual staple sequences for sc_EGFP2 20HB-Circ.

Name	Sequence 5' - 3'
core_1	CAGAGGTGTGGCGAGAAAGGAATTATAAAT
core_2	GATACCAATTTAGAGCTTGACGGTTGAGTG
core_3	ACCTGTCCTCTGAGTAGGTGTCATCCTTTCC
core_4	CGAATGAGAAATCGACGCTCAAGTTTATCCGG
core_5	ACTTGTACAAGTAATCTAGAGGGCCCTATTCCAAAAGGC
core_6	TATAGTGTTGACCGCCGCCGGGATGCGACACC
core_7	CACCTAAATGCTAGAGCTCGCTGATCAGCCTAGCGCGATCACATGGGGGCATCG
core_8	CGACTGTGCCTTCTAGTTGCCAGCCATCTGTCAGTCCGCCCTGAGCCTGGGGCACCACTTGG
core_9	GCTGCTGCCCGACAAGCCACAACGTCAATGGTTTCCAAG
core_10	AGCAGAACACCCCCAGAAGAACGTAGTAACGGAGTTTGTTGAAGCAAAAAAAG
core_11	TATCGCCGAGGTGAAGTTCGAGGCACTCTCG
core_12	AGTACAACTACAACACCACTACCTGAGCACCTGTTTGCCCCTCCCCCGTGCCTTCCTTGAC
core_13	AGCGCACCCGCATCGAGCTGAATCCTGCTGCAGATATA
core_14	AAGTCCGCGAGGACGGCAACATCAAAGACCCCAACGAGA
core_15	TGACCTACTCATGGCCGACAAGCATCGGCGACGGCCCCGTCCTGGAAGGTGCCACTCCCACTGT
core_16	CAGATATCAGCTCGGATCCACTAAAACTCT
core_17	CATCACACTGGCGGCCGCTCGAGCGTCCAGGGCTATTACGCCCCCTAAAAATCCCGGGAAGAA
core_18	ATGGTGAGCTCACTATAGGGAGACTCCAGTTC
core_19	CAAGGGCGAGGAGCTGTTCACCGACTTCTTCACATCAATCAGTACATAGATAGGGGGAAAGC
core_20	GGGTGGTGACTGCTTACTGGCTTAACTGATCT
core_21	CGACGTAAAAGCAGAGCTCTCTGGCGTTTCTG
core_22	GGCGAGGGCGGTAGGCGTGTACGAATGCCGC
core_23	TCTATATACGGCCACAAGTTCAGCGTGTCCGACCACCCGTCAATGGCCAATAGAACGTCA
core_24	TACTTGGCCCCGCCTCTCATTTTCGCTGGCATAAATTAAACACGACTTATCGCCACTGGCTGG
core_25	GGGCGTGATAAACAATCAGCATCCGACCGAGTTGCTCTTGCCCGGC
core_26	ACGGGGAGTGGAGTACAAGAGTCTAAAGGG
core_27	TTTTGGCACAAATGGGCGAGGGCGATGCCACCTACGGCA
core_28	CCAAAATCCCGTGCCGGTGAACTTCAAGATC
core_29	GTAAATGGAGTACATCATATTTTCAAGGATCAAAAGTGCTCATCATTGGAAAAC
core_30	CAATGACGCTGGTGAACATCTTCTTCAAGGAC
core_31	CCAAGTACCATGGTGCCGAAAAGGATGTAACGGGATAATACCGCGCCACATAGCTTGGTATG
core_32	TTTACGGTAAACTGCCAAGCTGGACATGAAGCAGCACG
core_33	GGACTTTCCATTGACGTCTATAGGCGTGCAGTGCTTC
core_34	TGTTCCCAGCATCAACTGGCCCACCCTCGT
core_35	GTAACCACCAGGAACATAGGCTCCGCCCCCCCGGTAAG
core_36	CACACCCGGCTAGGGTTAACCAATAAGTAGTTGTCACGCTCGTCGTAGAACTTT

core_37	AGTGTAGCTTTTTCCCGTAAAAAGGCCGCGT
core_38	CGGAACCCCACTATTTAAACCAGGTTAGCTCCTTCGGTC
core_39	CGAGGTGCTTGCCTGACTGTAGGTATCTCAGTTCGGTGTTCGTGCGCCACTAAAT
core_40	TTTGGGGTAAGGGCGACCACGCTCGAAGTAAGTTGGCCGC
core_41	GTTAAAATCATCGTGGTCGCCAGTTAATAGTGATCCTTT
core_42	TAAATTGTAAATCGGCTTGACGTTAATGTCAGAGTTCG
core_43	TGCCACCGCTTCATTTAATTGTTGCCGGGA
core_44	ATAGGGGGGCGAGTCTGCAACTTTATCCG
core_45	TTAGAAAAGATAGCGGAGAACCCCCCATCCTGGTCGAGCTGGACGGAGCCGCTA
core_46	CATATTTGAAAAGCGCCAGCCGGAAGGGCC
core_47	AGCGGATAGGTGAGCAAACCAAGTCATTCTGAGAATAGTTTGTGCAAAATGTATGTTTGGAA
core_48	TTTATCAGCGTTGTCAACCGGCTCCAGATTTA
core_49	TTACCGCTGTTGAGACCAAGCTTAGTCATC
core_50	CCACTCGTGCACCCATCGAAATTTGGCAGT
core_51	TTTTACTTTCACCAGCTAACTAGTTTGACTCCCCCGACC
core_52	AGTACTCAAAACAGGAAGGCAAGTGGGAGGCCATTGAC
core_53	TAAGATGCTTTTCTGTGACTGGTGCTCCGATGGTTATTTGGACTCC
core_54	CCATCCGGGAATAAGGGCGACACCATTGACG
core_55	ACGATCAATTCCGCGCATAGACCGCAAGTGTATCATATGACTTCAAGCATGCCCGAAGGCTA
core_56	CAGTCTATCAGCTCCGGTTCCCAGTCAATAC
core_57	AAGTGGTCTACATGATCCCCCATGGTATGCGG
core_58	GTGCTGCAATGATACCTAACTACACCCTAA
core_59	AAATGAAGTTTTAAAAGCTAGAGTAGGCCGAAGCGTTATACGTATTGGTACCG
core_60	TCAATCTAAGCGAAAGATCACAATGAGCAAAAGGCCAG
core_61	AAGTATATTAACTATCGTCTTGAGTCCAACCTGACGAGCGAGCGGGCCCGCGCT
core_62	ATGAGTAAACTTGGTCCTCCATCCAAAAGAACATTTCCATGCGGTTTAATACGA
core_63	CTGACAGTCGGCGAACGGCGAAACCCGACAGGACTATAAACGAACCCC
core_64	TACCAATCCGTTCAGCCCGACCGCTGCGCC
core_65	GCTTAATCAGTGAGGCGAGCGCAGTTGTTCCA
core_66	ACCTATCTAGCCCCCGGGCGTTTCCCCCTGGAAGCTCCCAGGTCGTT
core_67	CAGCGATCGCTCCAAGCTGGGCTGTGTGCA
core_68	CTGTCTATTTCGTTCATCAGCAAAAAGAACGGTCTCATGTCTCCACC
core_69	TCCATAGCGTAAAGTCTCCTGTTCCGACCCTGCCGCTAGCTCACG
core_70	CTCCCCGTCGTGTAGAGCGAGACAAAACCGTAATATTA
polyT_1	TTTTTCTTCGGGAAGCGTGGCGCTTTCTCATTACCGGATTCAAGTTTAAATGGCC
polyT_2	TTTTTAGCGAGGTATGTAGGCTAACAGGATTAGCAGTTTTT
polyT_3	TGCTGGCGGGTTTGTTTGCAAGCAGCAGTTTTT
polyT_4	TTTTTCATCGCATTGGCCTTTCTCCTTTTT
polyT_5	TTTTTTTGAAGTGGTGGCCTAGGTGCTACAGAGTTCTTTTT
polyT_6	TCGCCGATGACCGCCCAACGACCCCTTTTT
polyT_7	TTTTTGACGGCAGCGTGCAGCCGCCACAACATCGAGTTTTT
polyT_8	TTTTTGAACAGTATTTGGCATGATCCGGCAAACAAATTTTT
polyT_9	CAAGCTGCAACGGGACGTCAATAGCGATGGCCCACTTTTT
polyT_10	TTTTTTCATCTGCACCACCGGAGCTGACCCTGAAGTTTTTT
polyT_11	TTTTTGCCAGTTACCTTCGGAACAGTGTGCTGGAATTCTGGACGGCAA
polyT_12	TTTTTGTCGTAACAACTCCGCCCGGAAATGTTGAATTTTT
polyT_13	TTTTTCTCTTGACGGATCTCAAGAAGTTTTT
polyT_14	GACTTTCCCTACAAGACCCGCTGCGCTCTGCTGAATTTTT
polyT_15	ATGACGTACGCCTGGCCCACTACCTAATAAAATGAGGAAATTGTTTTT
polyT_16	TTTTTCGCCCATTGACTTTCCAAAATTTTTT
polyT_17	TTTTTCCACCGCTGGTAGCGGTGGTTTTTCACGCCCAGTA
polyT_18	GCTGCGCGCATGGACGAGCACGGCTACACTAGAATTTTT
polyT_19	TTTTTTACGTGAACCATCGATACGGGAGGGCTTACCATTTTT
polyT_20	TTTTTATTACGCGCAGACGTGGGATTTTGGTCATGTTTTT
polyT_21	TTTTTTACTCATACTCTTTCACTCAT
polyT_22	TTTTTATCCTTTGATCTTTTCTCTTCGGGGCGAGTAACGGC
polyT_23	TTTTTCACTGCATAATTCTCTTACTGTCATGAGTGTTACCTTTTTCCTATCAGG
polyT_24	TTTTTCGAAAACTCACGTTAATGTTGCCATTGTTACGGGGTCTGACGCTCAGTGGAATTTTT
polyT_25	GCTACAGGTCGCGTTAAATTTAAACTTATGGCGCAAAGAGTTGGTAGTTTTT
polyT_26	TTTTTTCTGGCCCCAGGTTATGGCAGTTTTT
polyT_27	TTTTTAGATTATCAAAAAGGATCTTCACCTATTGCGCAAAAATTGTTAAATCAGGGCATTAT
Circ_1	TCTATTCTGGGGGGTGGGGTGGGGCAGGACAGCAAGGGGGAGGATTGGGAAGACAATAGCAGGCATGCT GGGGATGCGGTGGGCTCTATGGCTTCTGAGGCGCGTTGACATTGATTATTGACTAGTTATTAATAGTAATC AATTACGGGGTCATTAGTTCATAGCCCATATATGGAGTTCCGCGTTACATAACTTACGGT

2.2.3 sc_EGFP3

Scaffold sequence for sc_EGFP3:
taggctgcgcgctcgctcgctcactgaggccgcccgggcaaagcccgggcgtcgggcgacctttggtcgcccggcctcagtgagcgagcg
agcgcgcagagagggagtggccaactccatcactaggggttccttgtagttaatgattaacccgccatgctacttatctacgtagccatgctcta
ggaagatcggaattcgcccttaagctagctagttattaatagtaatcaattacggggtcattagttcatagcccatatatggagttccgcgttacat
aacttacggtaaatggcccgcctggctgaccgcccaacgacccccgcccattgacgtcaataatgacgtatgttcccatagtaacgccaatag
ggactttccattgacgtcaatgggtggagtatttacggtaaactgcccacttggcagtacatcaagtgtatcatatgccaagtacgccccctattg
acgtcaatgacggtaaatggcccgcctggcattatgcccagtacatgaccttatgggactttcctacttggcagtacatctacgtattagtcatcgc
tattaccatggtgatgcggttttggcagtacatcaatgggcgtggatagcggtttgactcacggggatttccaagtctccaccccattgacgtcaat
gggagtttgttttggcaccaaaatcaacgggactttccaaaatgtcgtaacaactccgccccattgacgcaaatgggcggtaggcgtgtacggt
gggaggtctatataagcagagctggtttagtgaaccgtcagatcctgcagaagttggtcgtgaggcactgggcaggtaagtatcaaggttaca
agacaggtttaaggagaccaatagaaactgggcttgtcgagacagagaagactcttgcgtttctgataggcacctattggtcttactgacatcca
ctttgcctttctctccacaggtgtccaggcggccgccatggtgagcaagggcgaggagctgttcaccggggtggtgcccatcctggtcgagctg
gacggcgacgtaaacggccacaagttcagcgtgtccggcgagggcgagggcgatgccacctacggcaagctgaccctgaagttcatctgc
accaccggcaagctgcccgtgccctggcccaccctcgtgaccaccctgacctacggcgtgcagtgcttcagccgctaccccgaccacatga
agcagcacgacttcttcaagtccgccatgcccgaaggctacgtccaggagcgcaccatcttcttcaaggacgacggcaactacaagacccg
cgccgaggtgaagttcgagggcgacaccctggtgaaccgcatcgagctgaagggcatcgacttcaaggaggacggcaacatcctggggc
acaagctggagtacaactacaacagccacaacgtctatatcatggccgacaagcagaagaacggcatcaaggtgaacttcaagatccgcc
acaacatcgaggacggcagcgtgcagctcgccgaccactaccagcagaacacccccatcggcgacggccccgtgctgctgcccgacaa
ccactacctgagcacccagtccgccctgagcaaagaccccaacgagaagcgcgatcacatggtcctgctggagttcgtgaccgccgccgg
gatcactctcggcatggacgagctgtacaagtaataagcttggatccaatcaacctctggattacaaaatttgtgaaagattgactggtattctta
actatgttgctccttttacgctatgtggatacgctgctttaatgcctttgtatcatgctattgcttcccgtatggctttcattttctcctccttgtataaatcctg
gttgctgtctctttatgaggagttgtggcccgttgtcaggcaacgtggcgtggtgtgcactgtgtttgctgacgcaacccccactggttggggcattg
ccaccacctgtcagctcctttccgggactttcgctttccccctccctattgccacggcggaactcatcgccgcctgccttgcccgctgctggacag
gggctcggctgttgggcactgacaattccgtggtgttgtcggggaaatcatcgtcctttccttggctgctcgcctgtgttgccacctggattctgcgc
gggacgtccttctgctacgtcccttcggccctcaatccagcggaccttccttcccgcggcctgctgccggctctgcggcctcttccgcgtcttcgag
atctgcctcgactgtgccttctagttgccagccatctgttgtttgcccctcccccgtgccttccttgaccctggaaggtgccactcccactgtcctttcct
aataaaatgaggaaattgcatcgcattgtctgagtaggtgtcattctattctggggggtggggtggggcaggacagcaagggggaggattggg
aagacaatagcaggcatgctggggactcgagttaagggcgaattcccgataaggatcttcctagagcatggctacgtagataagtagcatgg
cgggttaatcattaactacaaggaacccctagtgatggagttggccactccctctctgcgcgctcgctcgctcactgaggccgggcgaccaaa
ggtcgcccgacgcccgggctttgcccgggcggcctcagtgagcgagcgagcgcgcagccttaATTAAcattaagcgcggcgggtgtggt
ggttacgcgcagcgtgaccgctacacttgccagcgccctagcgcccgctcctttcgctttcttcccttcctttctcgccacgttcgccggctttcccc
gtcaagctctaaatcgggggctccctttagggttccgatttagtgctttacggcacctcgaccccaaaaaacttgattagggtgatggttcacgta
gtgggccatcgccctgatagacggtttttcgccctttgacgttggagtccacgttctttaatagtggactcttgttccaaactggaacaacactcaac
cctatctcggtctattcttttgatttataagggattttgccgatttcggcctattggttaaaaaatgagctgatttaacaaaaatttaacgcgaattttaac
aaaatattaacgcttacaatttaggtggcacttttcggggaaatgtgcgcggaacccctatttgtttatttttctaaatacattcaaatatgtatccgctc
atgagacaataaccctgataaatgcttcaataatattgaaaaaggaagagtatgagtattcaacatttccgtgtcgcccttattcccttttttgcggc
attttgccttcctgtttttgctcacccagaaacgctggtgaaagtaaaagatgctgaagatcagttgggtgcacgagtgggttacatcgaactgga
tctcaacagcggtaagatccttgagagttttcgccccgaagaacgttttccaatgatgagcacttttaaagttctgctatgtggcgcggtattatccc
gtattgacgccgggcaagagcaactcggtcgccgcatacactattctcagaatgacttggttgagtactcaccagtcacagaaaagcatcttac
ggatggcatgacagtaagagaattatgcagtgctgccataaccatgagtgataacactgcggccaacttacttctgacaacgatcggaggac
cgaaggagctaaccgcttttttgcacaacatgggggatcatgtaactcgccttgatcgttgggaaccggagctgaatgaagccataccaaacg
acgagcgtgacaccacgatgcctgtagcaatggcaacaacgttgcgcaaactattaactggcgaactacttactctagcttcccggcaacaat
taatagactggatggaggcggataaagttgcaggaccacttctgcgctcggcccttccggctggctggtttattgctgataaatctggagccggt
gagcgtgggtcacgcggtatcattgcagcactggggccagatggtaagccctcccgtatcgtagttatctacacgacggggagtcaggcaact
atggatgaacgaaatagacagatcgctgagataggtgcctcactgattaagcattggtaactgtcagaccaagtttactcatatatactttagatt
gatttaaaacttcatttttaatttaaaaggatctaggtgaagatcctttttgataatctcatgaccaaaatcccttaacgtgagttttcgttccactgagc
gtcagaccccgtagaaaagatcaaaggatcttcttgagatcctttttttctgcgcgtaatctgctgcttgcaaacaaaaaaaccaccgctaccag
cggtggtttgtttgccggatcaagagctaccaactctttttccgaaggtaactggcttcagcagagcgcagataccaaatactgtccttctagtgta
gccgtagttaggccaccacttcaagaactctgtagcaccgcctacatacctcgctctgctaatcctgttaccagtggctgctgccagtggcgata
agtcgtgtcttaccgggttggactcaagacgatagttaccggataaggcgcagcggtcgggctgaacggggggttcgtgcacacagcccag
cttggagcgaacgacctacaccgaactgagatacctacagcgtgagctatgagaaagcgccacgcttcccgaagggagaaaggcggaca
ggtatccggtaagcggcagggtcggaacaggagagcgcacgagggagcttccagggggaaacgcctggtatctttatagtcctgtcgggttt
cgccacctctgacttgagcgtcgatttttgtgatgctcgtcaggggggcggagcctatggaaaaacgccagcaacgcggcctttttacggttcct
ggccttttgctggccttttgctcacCCTTAat

Corresponding DNA origami designs and staple lists:

2.2.3.1 sc_EGFP3 20HB

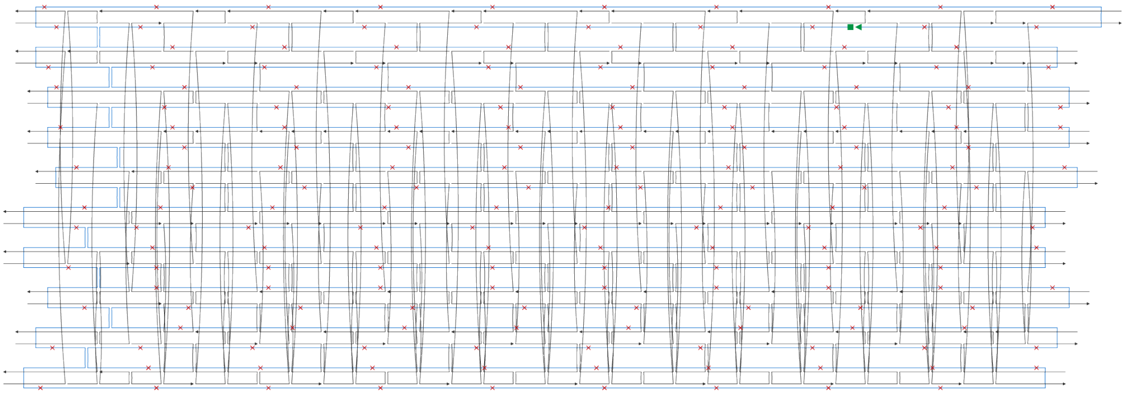

Fig. S31 I Scaffold routing and staple design for sc_EGFP3 20HB. Scaffold routing is shown in blue, staples are given in black, breakpoint for addition of sequence is marked by a green square (5') and arrow (3'). Design was prepared using caDNAno v0.1.

Table S18 I Individual staple sequences for sc_EGFP3 20HB.

Name	Sequence 5' - 3'
core_1	TAGATAAGTCAGGGCGAAAAAAGGTAGAGTAA
core_2	CTACAAGACTCCAACTTTCTACGCCATCCAGCTTGTGGCAGATGAACTTCAGGGT
core_3	GAACCCCTTAGCTAGCTTAAGGGGATGTACT
core_4	ACTCCCTGGAACAAAAACTCAGCAGAAGACCAGGATGGCCAGGGCACGGGCA
core_5	CTCTGCGCAATGACCCCGTAATTATAGCGA
core_6	GAGGCCGGACCGAGATTATCAAGCAATAACCTCGCCACGCCGTAGGTCAGG
core_7	GCGACCAAAACGCGGAACTCCATAAGTAGG
core_8	CGGGCTTTCGGCAAAATTAAATTATGACCCACGACACCTCATGTGGTCGGGGTA
core_9	GCCCGGGCAGCCAGGCGGGCCATGGGCATAA
core_10	GCGAGCGCTCAGCTCAAAAGTATACATCTGGCGTCAGTAAGCATGGCGGACTTGA
core_11	GCAGCCTAGACGTCAATGGGCGGGATTGACGT
core_12	AAGGCCAGTTGTTAAATTACCAATGTGTAGATCGCAAGAGAAGATGGTGCGCTCC
core_13	CAAAAGGTGGCGTTACTATGGGATGATACAC
core_14	CTAGAGCATGGCTACGAGTGTGAGTCAAACCGCTATCCACGCCCATT
core_15	GATCTTCGCGGGTTAATCATTAATTCGGTGT
core_16	CGAATTCCTTTGATCTGTCAAAGGCAGCATCTTATCGGGACCCCCTTGCTGTCCT
core_17	TTAATAACAGTGATGGAGTTGGCCTTTCTCAT
core_18	GATTACTATGGAACGAGAGTCCACGTGAGCAATATCTACGCAGAATAGAATGACA
core_19	TATGAACTGCTCGCTCGCTCACTTCCGCCTT
core_20	ATATGGGCTCATGAGATAGGGTTGAAAAAGGGTTGTAGTTGATGCAATTTCCTCA
core_21	AGTTATGTAGGTCGCCCGACGCCGTTCCGA
core_22	TTACCGTAGATCCTTTCCCTTAGAATACTCGGAGTGGCAGTGGGAGTGGCAC
core_23	GGGCGGTCGGCCTCAGTGAGCGATCCCCCT
core_24	GGTCGTTTCAATCTTTTTTTATTGAAGCAGGCCTCAAAGGCACGGGGGAGGG
core_25	TCATTATTATTAAGGGTGAGCAAACCCGACA
core_26	ACATACGTCTGACAGATTCGCGTAGCGGATAGCCCGGGCTGGCAACTAGAAGGC
core_27	TCCCTATCCAGGAACCGTAAAAA
core_28	CATTGACGTCAATGGGACGCTGAATCTATTAAGTTGTTGCTGTTGAGA
core_29	TCCCGTTGATTTTGGTCCAGCTCGTGGTCCTGCACGCTCGTCGGGGCG
core_30	TGGGGCGGAGTTGTTAGAACAGCTACCAGCCACTCCGGTTTTAAAAG
core_31	CCGTACACGCCTACCGCCGCCTGGCTCACCTGATCCCTACGGGAT
core_32	CTAAACCAGCTCTGCAGTGGATCCCAGTGAGCTCCTTCGGCGACC
core_33	ACGACCAACTTCTGCTCAGAAAAACTACGAGTAAGTTGTCAACCA
core_34	GTAACCTTGATACTTCAAGCCCACCATAGTTTATGGCAGCGTAAGA
core_35	ACTCGCCCTCGCCCTCGCCGGACGTGGAGAGAAGATCC
core_36	CGTTTACGTCGCCGTGCCAAAAACGCTCAG
core_37	GGGCACCACCCCGGTCGACATTTGATTTTGG
core_38	CTTGCTCACCATGGCGGCCCATTTTTCACCTA
core_39	GTGGAGAGAAAGGCAATTATATAGGTTTTAAA

core_40	GACCAATAGGTGCCTAAGGATCTGAACTTGG
core_41	TCTTCTCTGTCTCGAACCTGCCCAGTGAGG
core_42	CTGGTAGTTTGTCGGGCAGCAGCAAGCGAATCCAGTTCCAATCCT
core_43	TCCTCGATGCTCAGGGCGGACTGGGGTGGCAAAAACTCCCACCCCC
core_44	CCTTGATGCCATGTGATCGCGCTTGTCAGCAATGCTCATCGACAATGC
core_45	GATATAGAATCCCGGCGGCGGTCATGCCTGACAATACCGCGGAAAGGA
core_46	TCCAGCTTTACTTGTACAGCTCGGAGACAGCGAGTTGCTGTCAAGG
core_47	CCTTGAATAATCCAGAGGTTGAGAAAATGAAGTCATTCCAGATGG
core_48	GTTCACCAAGAATACCAGTCAATGATACAATGCTTTTCAGGCAGAT
core_49	GGTAGTGGGGTCGGCGAGCTGCACGCTGCCGGCTTGCCG
core_50	GGGTCTTTGTTGTGGCGGATCTTGAAGTTCAGTGGTCAC
core_51	AGCAGGACCGTTCTTCTGCTTGTCGGCCATGCGGCTGA
core_52	GAGAGTGCGTTGTGGCTGTTGTAGTTGTACAGAAGTCG
core_53	AAGCTTATGTGCCCCAGGATGTTGCCGTCCTTGGACGTA
core_54	AAATTTTGGTCGATGCCCTTCAGCTCGATGCGCCGTCGT
core_55	AGTCCCGGGCGGCGATGAGTTCCGCCGTGGCTGTCTTC
core_56	ATGCCCCACCGAGCCCCTGTCCAGCAGCGGGGCCCCACC
core_57	ACACAGTGACAACACCACGGAATTGTCAGTGCCCTACTCA
core_58	AACGGGCCGCAGCCAAGGAAAGGACGATGATTTTTATTA
core_59	AACCAGGACCGCGCAGAATCCAGGTGGCAACCTTCCAGG
core_60	AAGCCATGAGGGCCGAAGGGACGTAGCAGAGCAAACAA
core_61	AGGCATTGCAGGCCGCGGGAAGGAAGGTCCACAGTCG
core_62	CAAGGCAGAAAGGAGCTGACAGGTGTGCTCAACAGGCATGTGGTGC
core_63	CCAACAGACCAGTGGGGGTTGCCTCGTTGGTATGGCTGAGGGTG
core_64	TTCCCCGCACACCACGCCACGTCGAACTCCATCAAGGCAGCACTGC
core_65	ACAGGCGAACAACTCCTCATAAATCCATGCCTGCAAAAATGCTGCTT
core_66	AGGACGTCTTTATACAAGGAGGATTGGATCCCGATCGTTGCCTTCGG
core_67	GCTGGATTACGGGAAGCAATAGCATCTTTCACGTTATCACCTTGAAG
core_68	GAGTCCCCGATTCGTGCACCCAAC
core_69	ATTCGCCCTTAACTCCTACGTGCCGTCTATAGCATG
core_70	TAGCCATGCTCTAGGTGGGGTCAACGTGG
core_71	AATGATTAACCCGCCAGGAACCCTTCCAGTTT
core_72	CCAACTCCATCACTAGTTGACGGGAAGAATAG
core_73	GTGAGCGAGCGAGCGCAAGGAAGGGCCGAAAT
core_74	CGTCGGGCGACCTTTCTAGGGCGTTGTTAAA
core_75	GCTCACTGAGGCCGCGCGCGTAATTAATATT
core_76	CCCTAATCAAGTTTTTAAGATCCTTTTACTTTCTTACCGCCATTGCT
core_77	GTAAAGCACTAAATCTGCTACTAAACAGGAAACGTTCTTCGTTTG
core_78	CCCCCGATTTAGAGCGGGTTCCAATAAGGGAGCAGAACTCCCAACG
core_79	GCGAACGTGGCGAGAGCAGAGAGATACTCTGCGTCAACCATGTTG
core_80	CGAAAGGAGCGGGCGGGTCGCCCTTTATCAGTGTATGCGGTCCTC
core_81	GTGTAGCGGTCACGCTCCGGGCAAACATATTTGTGAGTACGCCGCAGT
core_82	TCAAGGATCACCAGCGTCCGCCTGGGTCTGCAAACTCCGCCAAAACCGCATCACCATGGTA
core_83	ATTGGAAAGGCAAAGGCCGAGCCGTTAAGGTGGAAAGTGACTAATACGTAGATGTACTGCC
core_84	GCCACATCGACACGATTTATCAAAAGGATCGCGTCAAAAAGTCCCATAAGGTCATGTACT
core_85	CTTGCCCGTCCTTTTTATACCGCGAAAATGAAACCTCCCATGCCAGGCGGGCCATTTACCGTC
core_86	TGAGAATAGGGTTATTGGGCTTACTATGAGTAACGGTTCACAATAGGGGGCGTACTTGGCATA
core_87	TGTGACTGGAATGTATTCCCCGTCGCTTAATCAGTGCCTCTTGATGTACTGCCAAGTGGGCAG
core_88	TGCGCAACTTGTTGCCTGATCTTGCGAAAAAAACCATCAAGGTCGTTCGCTCCAAGCTGGGC
core_89	CGTGGTGTCAACTTTATTTCTGGTATTAAAGGAGGTGCCAGCTCACGCTGTAGGTATCTCAG
core_90	TCATTCAGGCCGGAAGATGCCGCAAGTGTTGTAAAGGGAGTCTCCCTTCGGGAAGCGTGGCGC
core_91	GAGTTACAGGCTCCAGGAAATGTTTAAATCAAGAAAGCCGCCCTGCCGCTTACCGGATACCTG
core_92	AGCGGTTCTGCAATGCAATATTAACCAATAGGAAGAAAGGGAAGCTCCCTCGTGCGCTCTCCT
core_93	GTCAGAATACGGGAGTCTCATGTAAATTTCTGGCAAGGACTATAAAGATACCAGGCGTT
core_94	CTCATGGTGCCTGACTTAGAAAAGTAAGCGCCACCACACGCTCAAGTCAGAGGTGGCGAA
polyT_1	AAAAATCGACCCGCCGCGCTTAATGTTAATTTTT
polyT_2	TTTTTGGCTCCGCCCCCCTGACGAGCATCACGGCCGCGT
polyT_3	TTTTTCCCGTTCAGCCCGACTGTGTGCAAGTTACCATGGCCCA
polyT_4	ATGGAAAGCACCTATCCCTAAATTATAAACAGCTCGCTCCTCGAAGACGCGGAAGAGGCTTTTT
polyT_5	TTTTTCCACCCATTGACGTCATTTACCGT
polyT_6	TTTTTGTAACTATCGTCTTGCGCTGCGC
polyT_7	TTTTTAAACCTGTCTTAAATACTTTTTT
polyT_8	TTTTTACGACTTATCGCCGTATTTTTTTGTTTGCAAGTTTTT
polyT_9	TTTTTTCACCTCGGCTGGTCTCCTTTTTTT
polyT_10	TTTTTTAACAGGATTAGCAGAGCGCCGATGGGGGTGTTCTGCAGCTTG
polyT_11	TTTTTGTAAAAGGAGCAAATTCTCTTTTT
polyT_12	TTTTTTGCTACAGAGTTCTTGAGGGAGGGGGAACGGGGCC
polyT_13	TTTTTCGCAGAGCCGGCAAAAGCAGCGTATCCACATAGCTTTTT
polyT_14	TTTTTCACTAGAAGGACAGTATGCCTGCTATAATAAGTGGTGGCCTAACTACGGCTATTTTT

polyT_15	TTTTTTTAAGGCTGCGCAATAGGGGATGCCATC
polyT_16	TTTTTCTGCTGAAGCCCGAACCCTTTTT
polyT_17	AAGTGCCATCAGCGATCTGTCTTTTT
polyT_18	TTTTTTTTCCCCGAATGCTGGCGTTTTTCCATATTTTT
polyT_19	TTTTTTTGGTAGCTCTTAGCATTTGGTATCTGCGCTTTTTT
polyT_20	CGATGTAACCCACCCGCGCCAGTTGTCGAGGTATGTAGGCGGTTTTT
polyT_21	TTTTTTTACTGTCTTCCGCGCACATTTTT
polyT_22	TTTTTTGGTAGCGGTGGGTTGCAAACAAACCACCGCTTTTT
polyT_23	AATAGTTCCGTAGGTGGCATGGCAGCAGCCACTGGTTTTT
polyT_24	CACTGCATACATAGTTAGGGTGTCGCCCTCGAACTTTTTT
polyT_25	GGGAAGCATCTCAACTTGGAAATCCCCCCAACCCGGTAAGACTTTTT
polyT_26	TTTTTTATTTCGTTCATGTTTCTATGCGGGTCTTGTAGTTG
polyT_27	TTTTTCAGCAGATTACTTATCCGTTTTT
polyT_28	CGCGCAGATTCGGAAAAAGAGTTTTT

2.2.4 sc_EGFP4

Scaffold sequence for sc_EGFP4:
gttccgcgttacataacttacggtaaatggcccgcctggctgaccgcccaacgacccccgcccattgacgtcaataatgacgtatgttcccata
gtaacgccaatagggactttccattgacgtcaatgggtggagtatttacggtaaactgcccacttggcagtacatcaagtgtatcatatgccaagt
acgccccctattgacgtcaatgacggtaaatggcccgcctggcattatgcccagtacatgaccttatgggactttcctacttggcagtacatctac
gtattagtcatcgctattaccatggtgatgcggttttggcagtacatcaatgggcgtggatagcggtttgactcacggggatttccaagtctccacc
ccattgacgtcaatgggagtttgttttggcaccaaaatcaacgggactttccaaaatgtcgtaacaactccgccccattgacgcaaatgggcggt
aggcgtgtacggtgggaggtctatataagcagagctggtttagtgaaccgtcagatcctgcagaagttggtcgtgaggcactgggcaggtaag
tatcaaggttacaagacaggtttaaggagaccaatagaaactgggcttgtcgagacagagaagactcttgcgtttctgataggcacctattggt
cttactgacatccactttgcctttctctccacaggtgtccaggcggccgccatggtgagcaagggcgaggagctgttcaccggggtggtgcccat
cctggtcgagctggacggcgacgtaaacggccacaagttcagcgtgtccggcgagggcgagggcgatgccacctacggcaagctgaccc
tgaagttcatctgcaccaccggcaagctgcccgtgccctggcccaccctcgtgaccaccctgacctacggcgtgcagtgcttcagccgctacc
ccgaccacatgaagcagcacgacttcttcaagtccgccatgcccgaaggctacgtccaggagcgcaccatcttcttcaaggacgacggcaa
ctacaagacccgcgccgaggtgaagttcgagggcgacaccctggtgaaccgcatcgagctgaagggcatcgacttcaaggaggacggca
acatcctggggcacaagctggagtacaactacaacagccacaacgtctatatcatggccgacaagcagaagaacggcatcaaggtgaact
tcaagatccgccacaacatcgaggacggcagcgtgcagctcgccgaccactaccagcagaacacccccatcggcgacggccccgtgctg
ctgcccgacaaccactacctgagcacccagtccgccctgagcaaagaccccaacgagaagcgcgatcacatggtcctgctggagttcgtg
accgccgccgggatcactctcggcatggacgagctgtacaagtaataagcttggatccaatcaacctctggattacaaaatttgtgaaagattg
actggtattcttaactatgttgctccttttacgctatgtggatacgctgctttaatgcctttgtatcatgctattgcttcccgtatggctttcattttctcctcctt
gtataaatcctggttgctgtctctttatgaggagttgtggcccgttgtcaggcaacgtggcgtggtgtgcactgtgtttgctgacgcaacccccactg
gttggggcattgccaccacctgtcagctcctttccgggactttcgctttccccctccctattgccacggcggaactcatcgccgcctgccttgcccg
ctgctggacaggggctcggctgttgggcactgacaattccgtggtgttgtcggggaaatcatcgtcctttccttggctgctcgcctgtgttgccacct
ggattctgcgcgggacgtccttctgctacgtcccttcggccctcaatccagcggaccttccttcccgcggcctgctgccggctctgcggcctcttcc
gcgtcttcgagatctgcctcgactgtgccttctagttgccagccatctgttgtttgcccctcccccgtgccttccttgaccctggaaggtgccactccc
actgtcctttcctaataaaatgaggaaattgcatcgcattgtctgagtaggtgtcattctattctggggggtggggtggggcaggacagcaaggg
ggaggattgggaagacaatagcaggcatgctggggactcgagttaagggcgaattcccgataaggatcttcctagagcatggctacgtagat
aagtagcatggcgggttaatcattaactacaaggaacccctagtgatggagttggccactccctctctgcgcgctcgctcgctcactgaggccg
ggcgaccaaaggtcgcccgacgcccgggctttgcccgggcggcctcagtgagcgagcgagcgcgcagccttaATTAAcattaagcgcg
gcgggtgtggtggttacgcgcagcgtgaccgctacacttgccagcgccctagcgcccgctcctttcgctttcttcccttcctttctcgccacgttcgc
cggctttccccgtcaagctctaaatcgggggctccctttagggttccgatttagtgctttacggcacctcgaccccaaaaaacttgattagggtgat
ggttcacgtagtgggccatcgccctgatagacggtttttcgccctttgacgttggagtccacgttctttaatagtggactcttgttccaaactggaaca
acactcaaccctatctcggtctattcttttgatttataagggattttgccgatttcggcctattggttaaaaaatgagctgatttaacaaaaatttaacgc
gaattttaacaaaatattaacgcttacaatttaggtggcacttttcggggaaatgtgcgcggaacccctatttgtttatttttctaaatacattcaaatat
gtatccgctcatgagacaataaccctgataaatgcttcaataatattgaaaaaggaagagtatgagtattcaacatttccgtgtcgcccttattccc
ttttttgcggcattttgccttcctgtttttgctcacccagaaacgctggtgaaagtaaaagatgctgaagatcagttgggtgcacgagtgggttacatc
gaactggatctcaacagcggtaagatccttgagagttttcgccccgaagaacgttttccaatgatgagcacttttaaagttctgctatgtggcgcg
gtattatcccgtattgacgccgggcaagagcaactcggtcgccgcatacactattctcagaatgacttggttgagtactcaccagtcacagaaa
agcatcttacggatggcatgacagtaagagaattatgcagtgctgccataaccatgagtgataacactgcggccaacttacttctgacaacgat
cggaggaccgaaggagctaaccgcttttttgcacaacatgggggatcatgtaactcgccttgatcgttgggaaccggagctgaatgaagccat
accaaacgacgagcgtgacaccacgatgcctgtagcaatggcaacaacgttgcgcaaactattaactggcgaactacttactctagcttccc
ggcaacaattaatagactggatggaggcggataaagttgcaggaccacttctgcgctcggcccttccggctggctggtttattgctgataaatct
ggagccggtgagcgtgggtcacgcggtatcattgcagcactggggccagatggtaagccctcccgtatcgtagttatctacacgacggggag
tcaggcaactatggatgaacgaaatagacagatcgctgagataggtgcctcactgattaagcattggtaactgtcagaccaagtttactcatat
atactttagattgatttaaaacttcatttttaatttaaaaggatctaggtgaagatcctttttgataatctcatgaccaaaatcccttaacgtgagttttcgt
tccactgagcgtcagaccccgtagaaaagatcaaaggatcttcttgagatcctttttttctgcgcgtaatctgctgcttgcaaacaaaaaaaccac
cgctaccagcggtggtttgtttgccggatcaagagctaccaactctttttccgaaggtaactggcttcagcagagcgcagataccaaatactgtc
cttctagtgtagccgtagttaggccaccacttcaagaactctgtagcaccgcctacatacctcgctctgctaatcctgttaccagtggctgctgcca
gtggcgataagtcgtgtcttaccgggttggactcaagacgatagttaccggataaggcgcagcggtcgggctgaacggggggttcgtgcaca
cagcccagcttggagcgaacgacctacaccgaactgagatacctacagcgtgagctatgagaaagcgccacgcttcccgaagggagaaa
ggcggacaggtatccggtaagcggcagggtcggaacaggagagcgcacgagggagcttccagggggaaacgcctggtatctttatagtcc
tgtcgggtttcgccacctctgacttgagcgtcgatttttgtgatgctcgtcaggggggcggagcctatggaaaaacgccagcaacgcggcctttt
acggttcctggccttttgctggccttttgctcacCCTTAcattatgctctaggaagatcggaattcgcccttaagctagctagttattaatagtaatc
aattacggggtcattagttcatagcccatatatgga

Corresponding DNA origami designs and staple lists:

2.2.4.1 sc_EGFP4 20HB

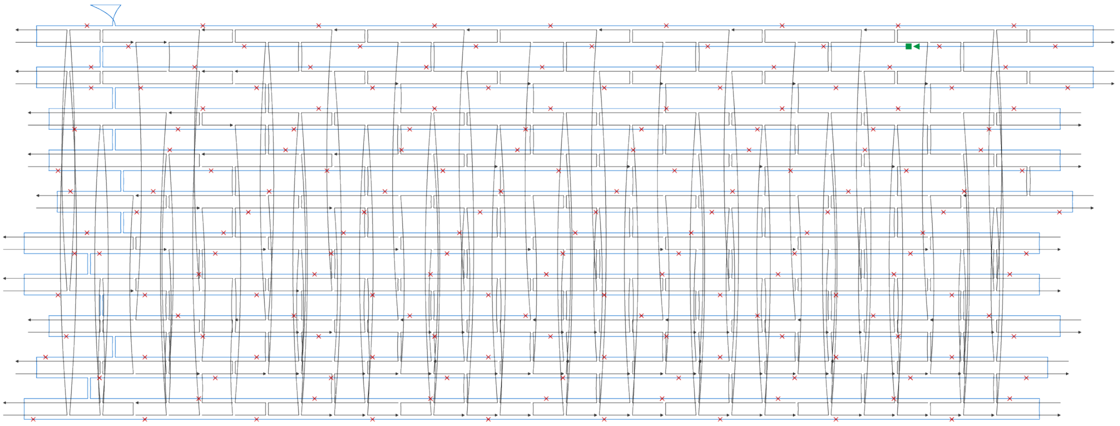

Fig. S32 | Scaffold routing and staple design for sc_EGFP4 20HB. Scaffold routing is shown in blue, staples are given in black, breakpoint for addition of sequence is marked by a green square (5') and arrow (3'). Design was prepared using caDNAno v0.1.

Table S19 | Individual staple sequences for sc_EGFP4 20HB.

Name	Sequence 5' - 3'
core_1	CAATGGAAGAAAGTCCCATAAGGCTGTCTT
core_2	GAACATACATGACTAATACGTAGAGTGCCTC
core_3	GGGGTCGTCTGCCAAAACCGCATACGGTTCA
core_4	TTTACCGAGTCAAACCGCTATCACCTCCCA
core_5	TATATGGTCAATGGGGTGGAGACGCGTCAAT
core_6	GTCAATAGGGGGCGTAGCTCAGAAACGCAAGAGTCTTCTCTGTCTCGTTTACCGT
core_7	TGGGCATGTAAATACTCCACCCAGGTGGCG
core_8	CCAAGTAGAGTCCCTATTGGCGTCGAGCATC
core_9	TAATAGCGGTCATTATTGACGTCTGGCGTTT
core_10	TTGATGTATGGGCGGTCAGCCAGAAGGCCAG
core_11	TCCCCGTGTAAGTTATGTAACGCGCATAATGT
core_12	ACAAGCCGCAAAGTGGATGTCATGTCGCCC
core_13	GGCGGCCGCCTGGACACTTGTAGTCTGACTCCGCCGGAAG
core_14	CGGTGAACAGCTCCTCGTGCGCTCCGATCTGTCAACTTTA
core_15	CCGTCCAGCTCGACCACGGACTTGCAATGCTTTTGTTGCC
core_16	GACACGCTGAACTTGGTCGGGGTATATATGCCAGTTAA
core_17	GCCGTAGGTGGCATCTAGGTCATTAAAAATTGCTACA
core_18	GCCCCAGCACCTCGGCGCGGGTCCTGTGGTGGTAGCT
core_19	TTGTGGCTCCTTGAAGAAGATGGCCCTTGGCTGGTAG
core_20	GTTCTTCTTAGCCTTCGGGCATGGGGATGGGCAGCAGATT
core_21	TGTGGCGGCGTGCTGCTTCATGTGTGGCCGTTAGAAGAT
core_22	TCGGCGAGGAAGCACTGCACGCCGGCCCTCGCGACGCTC
core_23	GTCGCCGAACGAGGGTGGGCCAGACTTCAGGGGGATTTT
core_24	AGGAGCAAGCATGATACAAAGGCGGCAGCAG
core_25	TCTTTCACAGGAGAAAATGAAAGCGATTGAGG
core_26	GAAGCAATACATAGTTAAGAATACCAGTCAACAGCTTGTCCAGCCACCGTCGTATACGGG
core_27	TACAAGGAAATTTTGTAATCCAGAGGTTGATATAGACGGGTCCTGCTATTTCGCTTTAAA
core_28	TAAAGAGACAGCAACCCGTCCCGCGCAGCACT
core_29	CTCCTCAAAGCTTATTACTTGTACAGCTCGTTGATGCCTCTATTAAAATCAGTGCTTCGGGG
core_30	CGTTGCCTGACAACGGCGAGCAGTTGGCCG
core_31	CCACGCCAGAGAGTGATCCCGGCGGCGGTCACTCGATGTGTAGTTCGAGTAAACTGCTGTTGA
core_32	TGCGTCAGCAAACACCCGACAACTTCGGT
core_33	GTGGGGGTAGCAGGACCATGTGATCGCGCTTGGTAGTGGTGTTGCCATGAAGTTTCGTGCACC
core_34	AGGTGGTGGCAATGCACAGCCGACCCCCAT
core_35	GAGCTGACGGTCTTTGCTCAGGGCGGACTGGCGGGGCCCGCTCGTCGATCTTCACTTTCACC
core_36	GCAGGCGGGGTTCCCAAGCATGCCGGTCGAGGTGCCGTAACACTACGCGCAAAAA
core_37	ACAGTCGAAAGGAAGGTCCGCTGCATACGGGCAATAAA
core_38	GCAAACAAGGACGTAGCAGAAGGAAGGATTTAGCAGAAGT

core_39	CTTCCAGGCCAGGTGGCAACACAGGGCCACAACATCCAG
core_40	TTTTATTAAAGGACGATGATTTCCAGTGCACAAGAGTAA
core_41	CCTACTCAAATTGTCAGTGCCCACCCAACCACGCAACGT
core_42	CCCCACCCCAGCAGCGGGCAAGCCGGAAAGTGGTGTCA
core_43	GGAGTGGGGCGCTGGCAAGTGTTATAAATCAAAAATA
core_44	TTGTAGTTAAGGGAAGAAAGCGATGAGTGTTCATGAGCG
core_45	TATCTACGCGGGGAAAGCCGGCGAACTATTAAATTATTGA
core_46	TATCGGGACCCTAAAGGGAGCCCCGGGCGAAATGTTGAAT
core_47	TCGCTCGCGTAGAGAATAGTGTATTCTGGCCC
core_48	AATCAGCTCATTTTTGCCGCGCTCTCTTGCCTTCTGTGATTTATCA
core_49	ATCGGCAAAATCCCTAGCGGTCAGCGCCACACTCTTACGGCCGAGC
core_50	AGACCGAGATAGGGTAAGGAGCGTCATTGGATCACTCATTCCGCCTC
core_51	TTGGAACAAGAGTCCACGTGGCCTCAAGGACGTTGTCAGGGAAGCT
core_52	GGACTCCAACGTCAAACGATTTATCGATGTAAAAAGCGTAGTTTG
core_53	TATCAGGGCGATGGCCAGCACTAACTTCAGCAGGCGAGTGGCATCG
core_54	TTTTGTTATTGGTATCCAGTGCTGAGTCTCGATGCGGTTCACCAGGGGTAAGACC
core_55	TTCCCCGAGTTTACCAATGCCAGGCGGGCCA
core_56	GCGCACATAGGCCGAAAAACCCGACAGGACTATAAAGATAGTGGGCAAAAGTGC
core_57	GGGGTTCCATAATACCCGCTGCGCGTGAGCGAGCGAGCGC
core_58	GTATTTAGAAAAGAATACAAAAATCGACGCTCAAGTCAGATTGACGTAACAAATAGGCAAAC
core_59	ATTTGAATAGTGCTCAGGCGCTAGCCAACTCCATCACTAG
core_60	TATTGTCTGTTCCAGTTTCCATAGGCTCCGCCCCCCTGATACTATGGGATACATTTTTTTGT
core_61	ATCAGGGTCGAAAACTGAGAAAGGAATGATTAACCCGCCA
core_62	TTTTCAATAGAACGTGAACCGTAAAAAGGCCGCGTTGCAATGGGCGAGCATTTGAAAAAAA
core_63	TCTTCCTGATCCAGTGAGCTTGATAGCCATGCTCTAGG
core_64	CACGGAAAAACCGTCAAGGGTGAGCAAAAGGCCAGCAAGCGGGCCAACTCATACCTTTTCTA
core_65	AGGGCGACAACTGATATCGGAAATTCGCCCTTAACTC
core_66	AAAATGCTGAACCATGGCGAATTCCGATCTTCCTAGAGGAACTCCAAGGGAATAGAAAACTC
core_67	GTACTCAAAGCCCGGGATTAAGGCTGCGCGCGTTAAATCGTTAATATATGATAC
core_68	CATCCGTAGGCCTCAGTAACCACCACACCCTAACCAAT
core_69	TGTCATGCGCCGAAGCAGATGGCTGGCAACTAGAAGGCGGTCGCCCAGATGCTTCGGCGTCA
core_70	GGTTATGGCAGAATGTCAAGGAAGGCACGGGGGAGGGGCAGAGAGGCATAATTTAGCAGAA
core_71	GAAGTAAGCCAAGGAGGAAAGGACAGTGGGAGTGGCACGGGTTCCCAGTGTTAAAACGTT
core_72	GTTAGCTCCACCACGGGACAATGCGATGCAATTTCCTCATGCTACTCCTCCGATTCTTACC
core_73	TACATGATGCCCCTGTCCACCCCCCAGAATAGAATGACAAAGATCCTGTTGTGCAAACCCACT
core_74	CTTACCAGCGGCGACTTGTAAGTTTTGTTAGTTTCCCCCTGGAAGCTCCCTCTCCTTGGCA
core_75	CGGGAGGGGCTCCAGACTGGTGAGCCGCGGGGGCAGATCTCGAAGACGCGGAAGCCGGGCAA
core_76	GTAGATAAAAAAGAGTAGAGAAAGCAGTTTCTATTGGTCTCCTTAAACTCATGTACCTTGATCC
core_77	ATAGTTGCTGCCGTCGTGTTGTAGTTGTACTC
core_78	TTCATCCAAACCACCCTCACCATGTAACCTTGATACTTACCTGCCCATGTACTGCGGTGGT
core_79	TATCTCAGCTGGACGGCTTGTCGGCCATGATTGGATCC
core_80	AGGCACCTTGCAAGCACCACCCACGACCAACTTCTGCAGGATCTGCACCATGGACGCGCA
core_81	CAGTTACAAGAAGTATCTTGAAGTTCACCTCCATGCC
core_82	TGGTCTGAGGATCTCATACGTCGCTAAACCAGCTCTGCTTATATAGCACGCCCACCTTTGAT
core_83	TCTAAAGTAGCGGCTCTGCACGCTGCCGTCCGAACTCC
core_84	TAAATCAACGGGGTCTCCTCGCCGCCGTACACGCCTACCGCCCATTTTTGGAAAAGTGGAAC
core_85	CTTTTAAAGGGTGGTCTGGGGGTGTTCTGCTCTCGTTGG
core_86	TGCTGAAGCATTGACACTTGATGTACTGCCAACCAGGC
core_87	CCAGTTACCACCTAAACGAGTTGTAATGTTACGTCGGGCGACCTTT
core_88	CTTCGGACTACGATATCGAACTTGATGTTGCCGTCCTCC
polyT_1	TTTTTTATTAATAACTAGCTACCCGTAATTGATTACTTTTT
polyT_2	TTTTTCGCTTACCGGATACCTCTGTTCCAGCAGCCAAAATTCGC
polyT_3	CATTGACGGCTATGAACTAATGACGCTTAAGCACCCTAATCATTTTT
polyT_4	TTTTTATTTTGGTGCCAAAACTGGAAAGTCCCGTTGTTTTT
polyT_5	TTTTTTTCGGGAAGCGTGGCGTCCGCCGACAGTATCTGGTAACAGGATTTTTT
polyT_6	GTCAGCTTGGGGCGGAGTTGTTACGACATTTAAACTCCGGTCATGAGATTTTTT
polyT_7	TTTTTCGGTGGTGCAGATGAGGCACGGGCAGCTTGCTTTTT
polyT_8	TTTTTTGTAGGTATCTCCAATGGCCTAACTACGGCTTTTTT
polyT_9	TTTTTTTGTCGGGCAGCAGCAGTGCTCAGGGAAAGCGAAAGTC
polyT_10	TTTTTGCTCCAAGCTGGGCTGTCGTATCCACATAGCGTAAATTGAAGTGCTCACCG
polyT_11	TTTTTCAATAGGGAGGGGTAGTGGTTTTT
polyT_12	TTTTTGTTCAGCCCGACCGCTCCGCAGAGCCATTAAAG
polyT_13	TTTTTTTCCCAATCCTCCCCCTTGCTGTCCTGGAGTCCCCACGATCAATCTTTTACCTAGATCACGTTAA
polyT_14	TTTTTCTTGAGTCCAACCCGCTGAGGCCGCAGGGCGCCTTATCCGGTAACTATCGTTTTTT
polyT_15	TTTTTAGTTTTTTGGTGCTATTGTCTTTTT
polyT_16	TTTTTCGCCACTGGCGACCCTGCTTTTT
polyT_17	TTTTTGCAAAAACAGGAAGGCAGCGTTTCTGGGTGATTTTT
polyT_18	TTTTTTAGCAGAGCGAGTCAGTAAGACACGACTTATTTTTT
polyT_19	CCAAGTCATTCTTGTATACCGCGCAGGTGCACGAACCCCCCTTTTT

polyT_20	TTTTTTTCATTCAGCTCCCGATGAGTTCCGCCGTGGTTTTT
polyT_21	TTTTTGTTCTTGAAGTGGTGAGGCGGTGCTACAGATTTTT
polyT_22	TGACCCACCGATGCCCTTCAGTCGGTGTAGGTCGTTCTTTTT
polyT_23	TTTTTTATCAAAAAGGTTTGGTATGGCTTTTT
polyT_24	TTTTTACACTAGAAGTTTCTCCCTTTTT
polyT_25	TGCGCTCAATAGGTGCCTATTTCTCATAGCTCACGCTTTTT

2.2.5 sc_EGFP5

Scaffold sequence for sc_EGFP5:
taggctgcgcgctcgctcgctcactgaggccgcccgggcaaagcccgggcgtcgggcgacctttggtcgcccggcctcagtgagcgagcg
agcgcgcagagagggagtggccaactccatcactaggggttccttgtagttaatgattaacccgccatgctacttatctacgtagccatgctcta
ggaagatcggaattcgcccttaagctagctagttattaatagtaatcaattacggggtcattagttcatagcccatatatggagttccgcgttacat
aacttacggtaaatggcccgcctggctgaccgcccaacgacccccgcccattgacgtcaataatgacgtatgttcccatagtaacgccaatag
ggactttccattgacgtcaatgggtggagtatttacggtaaactgcccacttggcagtacatcaagtgtatcatatgccaagtacgccccctattg
acgtcaatgacggtaaatggcccgcctggcattatgcccagtacatgaccttatgggactttcctacttggcagtacatctacgtattagtcatcgc
tattaccatggtgatgcggttttggcagtacatcaatgggcgtggatagcggtttgactcacggggatttccaagtctccaccccattgacgtcaat
gggagtttgttttggcaccaaaatcaacgggactttccaaaatgtcgtaacaactccgccccattgacgcaaatgggcggtaggcgtgtacggt
gggaggtctatataagcagagctggtttagtgaaccgtcagatcctgcagaagttggtcgtgaggcactgggcaggtaagtatcaaggttaca
agacaggtttaaggagaccaatagaaactgggcttgtcgagacagagaagactcttgcgtttctgataggcacctattggtcttactgacatcca
ctttgcctttctctccacaggtgtccaggcggccgccatggtgagcaagggcgaggagctgttcaccggggtggtgcccatcctggtcgagctg
gacggcgacgtaaacggccacaagttcagcgtgtccggcgagggcgagggcgatgccacctacggcaagctgaccctgaagttcatctgc
accaccggcaagctgcccgtgccctggcccaccctcgtgaccaccctgacctacggcgtgcagtgcttcagccgctaccccgaccacatga
agcagcacgacttcttcaagtccgccatgcccgaaggctacgtccaggagcgcaccatcttcttcaaggacgacggcaactacaagacccg
cgccgaggtgaagttcgagggcgacaccctggtgaaccgcatcgagctgaagggcatcgacttcaaggaggacggcaacatcctggggc
acaagctggagtacaactacaacagccacaacgtctatatcatggccgacaagcagaagaacggcatcaaggtgaacttcaagatccgcc
acaacatcgaggacggcagcgtgcagctcgccgaccactaccagcagaacacccccatcggcgacggccccgtgctgctgcccgacaa
ccactacctgagcacccagtccgccctgagcaaagaccccaacgagaagcgcgatcacatggtcctgctggagttcgtgaccgccgccgg
gatcactctcggcatggacgagctgtacaagtaataagcttggatccaatcaacctctggattacaaaatttgtgaaagattgactggtattctta
actatgttgctccttttacgctatgtggatacgctgctttaatgcctttgtatcatgctattgcttcccgtatggctttcattttctcctccttgtataaatcctg
gttgctgtctctttatgaggagttgtggcccgttgtcaggcaacgtggcgtggtgtgcactgtgtttgctgacgcaacccccactggttggggcattg
ccaccacctgtcagctcctttccgggactttcgctttccccctccctattgccacggcggaactcatcgccgcctgccttgcccgctgctggacag
gggctcggctgttgggcactgacaattccgtggtgttgtcggggaaatcatcgtcctttccttggctgctcgcctgtgttgccacctggattctgcgc
gggacgtccttctgctacgtcccttcggccctcaatccagcggaccttccttcccgcggcctgctgccggctctgcggcctcttccgcgtcttcgag
atctgcctcgactgtgccttctagttgccagccatctgttgtttgcccctcccccgtgccttccttgaccctggaaggtgccactcccactgtcctttcct
aataaaatgaggaaattgcatcgcattgtctgagtaggtgtcattctattctggggggtggggtggggcaggacagcaagggggaggattggg
aagacaatagcaggcatgctggggactcgagttaagggcgaattcccgataaggatcttcctagagcatggctacgtagataagtagcatgg
cgcattgccttaATTAAcattaagcgcggcgggtgtggtggttacgcgcagcgtgaccgctacacttgccagcgccctagcgcccgctccttt
cgctttcttcccttcctttctcgccacgttcgccggctttccccgtcaagctctaaatcgggggctccctttagggttccgatttagtgctttacggcacc
tcgaccccaaaaaacttgattagggtgatggttcacgtagtgggccatcgccctgatagacggtttttcgccctttgacgttggagtccacgttcttt
aatagtggactcttgttccaaactggaacaacactcaaccctatctcggtctattcttttgatttataagggattttgccgatttcggcctattggttaaa
aaatgagctgatttaacaaaaatttaacgcgaattttaacaaaatattaacgcttacaatttaggtggcacttttcggggaaatgtgcgcggaacc
cctatttgtttatttttctaaatacattcaaatatgtatccgctcatgagacaataaccctgataaatgcttcaataatattgaaaaaggaagagtatg
agtattcaacatttccgtgtcgcccttattcccttttttgcggcattttgccttcctgtttttgctcacccagaaacgctggtgaaagtaaaagatgctga
agatcagttgggtgcacgagtgggttacatcgaactggatctcaacagcggtaagatccttgagagttttcgccccgaagaacgttttccaatga
tgagcacttttaaagttctgctatgtggcgcggtattatcccgtattgacgccgggcaagagcaactcggtcgccgcatacactattctcagaatg
acttggttgagtactcaccagtcacagaaaagcatcttacggatggcatgacagtaagagaattatgcagtgctgccataaccatgagtgata
acactgcggccaacttacttctgacaacgatcggaggaccgaaggagctaaccgcttttttgcacaacatgggggatcatgtaactcgccttga
tcgttgggaaccggagctgaatgaagccataccaaacgacgagcgtgacaccacgatgcctgtagcaatggcaacaacgttgcgcaaact
attaactggcgaactacttactctagcttcccggcaacaattaatagactggatggaggcggataaagttgcaggaccacttctgcgctcggcc
cttccggctggctggtttattgctgataaatctggagccggtgagcgtgggtcacgcggtatcattgcagcactggggccagatggtaagccctc
ccgtatcgtagttatctacacgacggggagtcaggcaactatggatgaacgaaatagacagatcgctgagataggtgcctcactgattaagca
ttggtaactgtcagaccaagtttactcatatatactttagattgatttaaaacttcatttttaatttaaaaggatctaggtgaagatcctttttgataatctc
atgaccaaaatcccttaacgtgagttttcgttccactgagcgtcagaccccgtagaaaagatcaaaggatcttcttgagatcctttttttctgcgcgt
aatctgctgcttgcaaacaaaaaaaccaccgctaccagcggtggtttgtttgccggatcaagagctaccaactctttttccgaaggtaactggctt
cagcagagcgcagataccaaatactgtccttctagtgtagccgtagttaggccaccacttcaagaactctgtagcaccgcctacatacctcgct
ctgctaatcctgttaccagtggctgctgccagtggcgataagtcgtgtcttaccgggttggactcaagacgatagttaccggataaggcgcagc
ggtcgggctgaacggggggttcgtgcacacagcccagcttggagcgaacgacctacaccgaactgagatacctacagcgtgagctatgag
aaagcgccacgcttcccgaagggagaaaggcggacaggtatccggtaagcggcagggtcggaacaggagagcgcacgagggagcttc
caggggggaaacgcctggtatctttatagtcctgtcgggtttcgccacctctgacttgagcgtcgatttttgtgatgctcgtcaggggggcggagcct
atggaaaaacgccagcaacgcggcctttttacggttcctggccttttgctggccttttgctcacCCTTAat

Corresponding DNA origami designs and staple lists:

2.2.5.1 sc_EGFP5 20HB

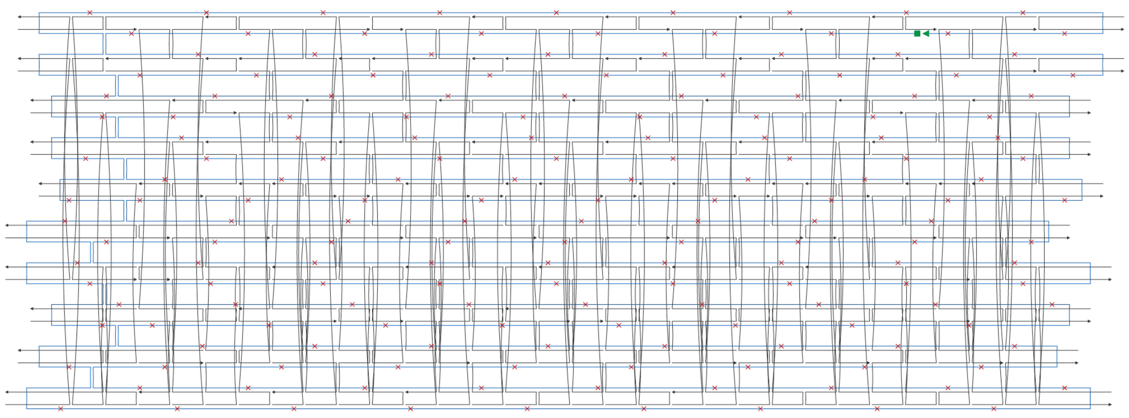

Fig. S33 l Scaffold routing and staple design for sc_EGFP5 20HB. Scaffold routing is shown in blue, staples are given in black, breakpoint for addition of sequence is marked by a green square (5') and arrow (3'). Design was prepared using caDNAno v0.1.

Table S20 l Individual staple sequences for sc_EGFP5 20HB.

Name	Sequence 5' - 3'
core_1	CAATGGAAGAAAGTCCCATAAGGCTGTCTT
core_2	GAACATACATGACTAATACGTAGAGTGCCTC
core_3	GGGGTCGTCTGCCAAAACCGCATACGGTTCA
core_4	TTTACCGAGTCAAACCGCTATCACCTCCCA
core_5	TATATGGTCAATGGGGTGGAGACGCGTCAAT
core_6	GTCAATAGGGGGCGTAGCTCAGAAACGCAAGAGTCTTCTCTGTCTCGTTTACCGT
core_7	TGGGCATGTAAATACTCCACCCAGGTGGCG
core_8	CCAAGTAGAGTCCCTATTGGCGTCGAGCATC
core_9	TAATAGCGGTCATTATTGACGTCTGGCGTTT
core_10	TTGATGTATGGGCGGTCAGCCAGAAGGCCAG
core_11	TCCCCGTGTAAGTTATGTAACGCGCATAATGT
core_12	ACAAGCCGCAAAGTGGATGTCATGTCGCCC
core_13	GGCGGCCGCCTGGACACTTGTAGTCTGACTCCGCCGGAAG
core_14	CGGTGAACAGCTCCTCGTGCGCTCCGATCTGTCAACTTTA
core_15	CCGTCCAGCTCGACCACGGACTTGCAATGCTTTTGTTGCC
core_16	GACACGCTGAACTTGGTCGGGGTATATATGCCAGTTAA
core_17	GCCGTAGGTGGCATCTAGGTCATTAAAAATTGCTACA
core_18	GCCCCAGCACCTCGGCGCGGGTCCTGTGGTGGTAGCT
core_19	TTGTGGCTCCTTGAAGAAGATGGCCCTTGGCTGGTAG
core_20	GTTCTTCTTAGCCTTCGGGCATGGGGATGGGCAGCAGATT
core_21	TGTGGCGGCGTGCTGCTTCATGTGTGGCCGTTAGAAGAT
core_22	TCGGCGAGGAAGCACTGCACGCCGGCCCTCGCGACGCTC
core_23	GTCGCCGAACGAGGGTGGGCCAGACTTCAGGGGGATTTT
core_24	AGGAGCAAGCATGATACAAAGGCGGCAGCAG
core_25	TCTTTCACAGGAGAAAATGAAAGCGATTGAGG
core_26	GAAGCAATACATAGTTAAGAATACCAGTCAACAGCTTGTCCAGCCACCGTCGTATACGGG
core_27	TACAAGGAAATTTTGTAATCCAGAGGTTGATATAGACGGGTCCTGCTATTTCGCTTTAAA
core_28	TAAAGAGACAGCAACCCGTCCCGCGCAGCACT
core_29	CTCCTCAAAGCTTATTACTTGTACAGCTCGTTGATGCCTCTATTAAAATCAGTGCTTCGGGG
core_30	CGTTGCCTGACAACGGCGAGCAGTTGGCCG
core_31	CCACGCCAGAGAGTGATCCCGGCGGCGGTCACTCGATGTGTAGTTCGAGTAAACTGCTGTTGA
core_32	TGCGTCAGCAAACACCCGACAACTTCGGT
core_33	GTGGGGGTAGCAGGACCATGTGATCGCGCTTGGTAGTGGTGTTGCCATGAAGTTTCGTGCACC
core_34	AGGTGGTGGCAATGCACAGCCGACCCCCAT
core_35	GAGCTGACGGTCTTTGCTCAGGGCGGACTGGCGGGGCCCGCTCGTCGATCTTCACTTTCACC
core_36	GCAGGCGGGGTTCCCAAGCATGCCGGTCGAGGTGCCGTAACACTACGCGCAAAAA
core_37	ACAGTCGAAAGGAAGGTCCGCTGCATACGGGCAATAAA
core_38	GCAAACAAGGACGTAGCAGAAGGAAGGATTTAGCAGAAGT

core_39	CTTCCAGGCCAGGTGGCAACACAGGGCCACAACATCCAG
core_40	TTTTATTAAAGGACGATGATTTCCAGTGCACAAGAGTAA
core_41	CCTACTCAAATTGTCAGTGCCCACCCAACCACGCAACGT
core_42	CCCCACCCCAGCAGCGGGCAAGCCGGAAAGTGGTGTCA
core_43	GGAGTGGGGCGCTGGCAAGTGTTATAAATCAAAAATA
core_44	TTGTAGTTAAGGGAAGAAAGCGATGAGTGTTCATGAGCG
core_45	TATCTACGCGGGGAAAGCCGGCGAACTATTAAATTATTGA
core_46	TATCGGGACCCTAAAGGGAGCCCCGGGCGAAATGTTGAAT
core_47	TCGCTCGCGTAGAGAATAGTGTATTCTGGCCC
core_48	AATCAGCTCATTTTTGCCGCGCTCTCTTGCCTTCTGTGATTTATCA
core_49	ATCGGCAAAATCCCTAGCGGTCAGCGCCACACTCTTACGGCCGAGC
core_50	AGACCGAGATAGGGTAAGGAGCGTCATTGGATCACTCATTCCGCCTC
core_51	TTGGAACAAGAGTCCACGTGGCCTCAAGGACGTTGTCAGGGAAGCT
core_52	GGACTCCAACGTCAAACGATTTATCGATGTAAAAAGCGTAGTTTG
core_53	TATCAGGGCGATGGCCAGCACTAACTTCAGCAGGCGAGTGGCATCG
core_54	TTTTGTTATTGGTATCCAGTGCTGAGTCTCGATGCGGTTCACCAGGGGTAAGACC
core_55	TTCCCCGAGTTTACCAATGCCAGGCGGGCCA
core_56	GCGCACATAGGCCGAAAAACCCGACAGGACTATAAAGATAGTGGGCAAAAGTGC
core_57	GGGGTTCCATAATACCCGCTGCGCGTGAGCGAGCGAGCGC
core_58	GTATTTAGAAAAGAATACAAAAATCGACGCTCAAGTCAGATTGACGTAACAAATAGGCAAAC
core_59	ATTTGAATAGTGCTCAGGCGCTAGCCAACTCCATCACTAG
core_60	TATTGTCTGTTCCAGTTTCCATAGGCTCCGCCCCCCTGATACTATGGGATACATTTTTTTGT
core_61	ATCAGGGTCGAAAACTGAGAAAGGAATGATTAACCCGCCA
core_62	TTTTCAATAGAACGTGAACCGTAAAAAGGCCGCGTTGCAATGGGCGAGCATTTGAAAAAAA
core_63	TCTTCCTGATCCAGTGAGCTTGATAGCCATGCTCTAGG
core_64	CACGGAAAAACCGTCAAGGGTGAGCAAAAGGCCAGCAAGCGGGCCAACTCATACCTTTTCTA
core_65	AGGGCGACAACTGATATCGGAAATTCGCCCTTAACTC
core_66	AAAATGCTGAACCATGGCGAATTCCGATCTTCCTAGAGGAACTCCAAGGGAATAGAAAACTC
core_67	GTACTCAAAGCCCGGGATTAAGGCTGCGCGCGTTAAATCGTTAATATATGATAC
core_68	CATCCGTAGGCCTCAGTAACCACCACACCCTAACCAAT
core_69	TGTCATGCGCCGAAGCAGATGGCTGGCAACTAGAAGGCGGTCGCCCAGATGCTTCGGCGTCA
core_70	GGTTATGGCAGAATGTCAAGGAAGGCACGGGGGAGGGGCAGAGAGGCATAATTTAGCAGAA
core_71	GAAGTAAGCCAAGGAGGAAAGGACAGTGGGAGTGGCACGGGTTCCCAGTGTTAAAACGTT
core_72	GTTAGCTCCACCACGGGACAATGCGATGCAATTTCCTCATGCTACTCCTCCGATTCTTACC
core_73	TACATGATGCCCCTGTCCACCCCCCAGAATAGAATGACAAAGATCCTGTTGTGCAAACCCACT
core_74	CTTACCAGCGGCGACTTGTAAGTTTTGTTAGTTTCCCCCTGGAAGCTCCCTCTCCTTGGCA
core_75	CGGGAGGGGCTCCAGACTGGTGAGCCGCGGGGGCAGATCTCGAAGACGCGGAAGCCGGGCAA
core_76	GTAGATAAAAAAGAGTAGAGAAAGCAGTTTCTATTGGTCTCCTTAAACTCATGTACCTTGATCC
core_77	ATAGTTGCTGCCGTCGTGTTGTAGTTGTACTC
core_78	TTCATCCAAACCACCCTCACCATGTAACCTTGATACTTACCTGCCCATGTACTGCGGTGGT
core_79	TATCTCAGCTGGACGGCTTGTCGGCCATGATTGGATCC
core_80	AGGCACCTTGCAAGCACCACCCACGACCAACTTCTGCAGGATCTGCACCATGGACGCGCA
core_81	CAGTTACAAGAAGTATCTTGAAGTTCACCTCCATGCC
core_82	TGGTCTGAGGATCTCATACGTCGCTAAACCAGCTCTGCTTATATAGCACGCCCACCTTTGAT
core_83	TCTAAAGTAGCGGCTCTGCACGCTGCCGTCCGAACTCC
core_84	TAAATCAACGGGGTCTCCTCGCCGCCGTACACGCCTACCGCCCATTTTTGGAAAAGTGGAAC
core_85	CTTTTAAAGGGTGGTCTGGGGGTGTTCTGCTCTCGTTGG
core_86	TGCTGAAGCATTGACACTTGATGTACTGCCAACCAGGC
core_87	CCAGTTACCACCTAAACGAGTTGTAATGTTACGTCGGGCGACCTTT
core_88	CTTCGGACTACGATATCGAACTTGATGTTGCCGTCCTCC
polyT_1	TTTTTTATTAATAACTAGCTACCCGTAATTGATTACTTTTT
polyT_2	TTTTTCGCTTACCGGATACCTCTGTTCCAGCAGCCAAAATTCGC
polyT_3	CATTGACGGCTATGAACTAATGACGCTTAAGCACCCTAATCATTTTT
polyT_4	TTTTTATTTTGGTGCCAAAACTGGAAAGTCCCGTTGTTTTT
polyT_5	TTTTTTTCGGGAAGCGTGGCGTCCGCCGACAGTATCTGGTAACAGGATTTTTT
polyT_6	GTCAGCTTGGGGCGGAGTTGTTACGACATTTAAACTCCGGTCATGAGATTTTTT
polyT_7	TTTTTCGGTGGTGCAGATGAGGCACGGGCAGCTTGCTTTTT
polyT_8	TTTTTTGTAGGTATCTCCAATGGCCTAACTACGGCTTTTTT
polyT_9	TTTTTTTGTCGGGCAGCAGCAGTGCTCAGGGAAAGCGAAAGTC
polyT_10	TTTTTGCTCCAAGCTGGGCTGTCGTATCCACATAGCGTAAATTGAAGTGCTCACCG
polyT_11	TTTTTCAATAGGGAGGGGTAGTGGTTTTT
polyT_12	TTTTTGTTCAGCCCGACCGCTCCGCAGAGCCATTAAAG
polyT_13	TTTTTTTCCCAATCCTCCCCCTTGCTGTCCTGGAGTCCCCACGATCAATCTTTTACCTAGATCACGTTAA
polyT_14	TTTTTCTTGAGTCCAACCCGCTGAGGCCGCAGGGCGCCTTATCCGGTAACTATCGTTTTTT
polyT_15	TTTTTAGTTTTTTGGTGCTATTGTCTTTTT
polyT_16	TTTTTCGCCACTGGCGACCCTGCTTTTT
polyT_17	TTTTTGCAAAAACAGGAAGGCAGCGTTTCTGGGTGATTTTT
polyT_18	TTTTTTAGCAGAGCGAGTCAGTAAGACACGACTTATTTTTT
polyT_19	CCAAGTCATTCTTGTATACCGCGCAGGTGCACGAACCCCCCTTTTT

polyT_20	TTTTTTTCATTCAGCTCCCGATGAGTTCCGCCGTGGTTTTT
polyT_21	TTTTTGTTCTTGAAGTGGTGAGGCGGTGCTACAGATTTTT
polyT_22	TGACCCACCGATGCCCTTCAGTCGGTGTAGGTCGTTCTTTTT
polyT_23	TTTTTTATCAAAAAGGTTTGGTATGGCTTTTT
polyT_24	TTTTTACACTAGAAGTTTCTCCCTTTTT
polyT_25	TGCGCTCAATAGGTGCCTATTTCTCATAGCTCACGCTTTTT

2.2.5.2 sc_EGFP5 20HB-exL

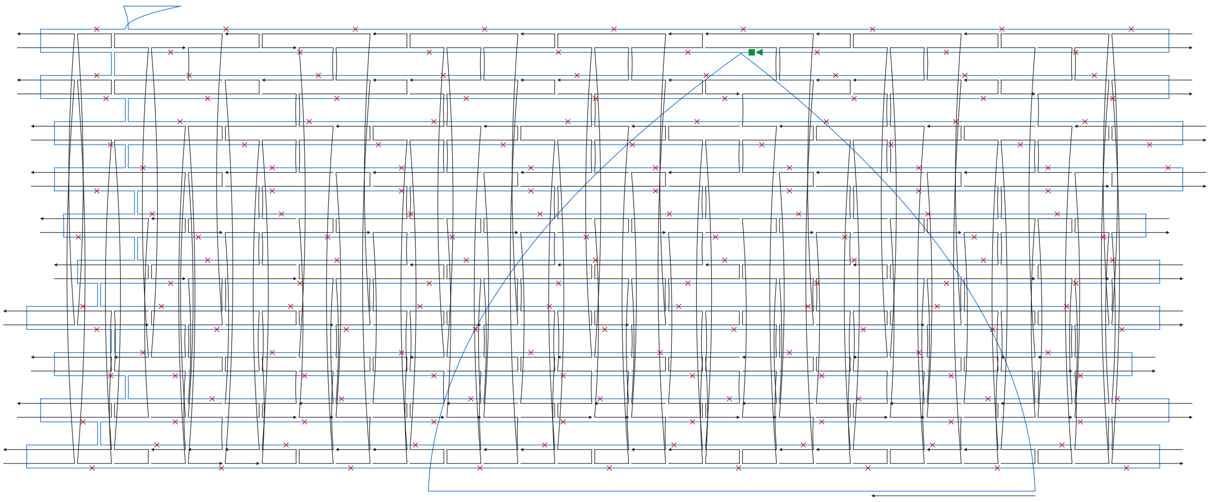

Fig. S34 I Scaffold routing and staple design for sc_EGFP5 20HB-exL. Scaffold routing is shown in blue, staples are given in black, breakpoint for addition of sequence is marked by a green square (5') and arrow (3'). Design was prepared using caDNAno v0.1.

Table S21 I Individual staple sequences for sc_EGFP5 20HB-exL.

Name	Sequence 5' - 3'
core_1	TTAACTACCTAATTAAACTCCACCCATTGACCATCACC
core_2	GCGTTTTTAGGGGGCGTACTTGGCAAGGTCAT
core_3	ATGTAACGCGGAACTCCCCTTGGAAAGTCCCGTTGATTTTGGTGCCACATTTACCTCTCAAGA
core_4	GTAAGTTAACTAATGACCCCGTATCTCAGT
core_5	AAACAAACCCATTTGCGTCAATGGTGAACTTAGTGGTCC
core_6	AGACTTGGTATAGACCTCCCACCATCGCCCAAACCAGC
core_7	ATCCACGCATCTGACGGTTCACTCTTGTGGCACGCTCA
core_8	TAGATGTTTAAACCTGTCTTGTCCTCGCCCATAACTAC
core_9	TGCCCAGTGCCTCACACCAGGATGCCCCAG
core_10	GTCTCGACAAGCCCAGGACACCTGATCCATAG
core_11	ACGCCGTAGCTTGCCGTAGGTGGCGTACACGC
core_12	CATGTGGTGCCGGACACGCTGAAAAACCAGC
core_13	GCATGGCGTCGCCGTCCAGCTCGGACCAACT
core_14	AAGATGGTCCCCGGTGAACAGCTAACCTTGA
core_15	CGGGTCTCATGGCGGCCGCCTGTTTCTAT
core_16	CAGGGTGAAAGGCAAAGTGGATGGCAAGAG
core_17	AGCACGGGTTGCTCAGGGCGGACTGTCCCGG
core_18	GCTGGTAGACCATGTGATCGCGCATGCCCCA
core_19	GTCCTCGGATCCCGGCGGCGGTACACAGTG
core_20	CACCTTGAATTACTTGTACAGCTAACGGGCC
core_21	ATGATATATGTAATCCAGAGGTTAACCAGGA
core_22	ACTCCAGCTTAAGAATACCAGTCAAGCCATA
core_23	GGGGTCTGCCGTCGCCGATGGGGGTGTTCTAGCACTGCTTTGCGCACAGCCGGA
core_24	CCAGCAGGTGGTCGGCGAGCTGCACGCTGCCTGCTGCTTATCGTGGTCCGGCTCC
core_25	CCGAGAGTATGTTGTGGCGGATCTTGAAGTTCCTTCGGCTTCATTCTGCTGCAA
core_26	CCAAGCTTTGCCGTTCTTCTGCTTGTCGGCCCTTGAAGGCGAGTTAGATACGG
core_27	ACAAATTTGACGTTGTGGCTGTTGTAGTTGTCCTCGGCGAAAGCGGTTGCCTG
core_28	GGGTTGCGTCAGCAACACGAACTCTACAGGC
core_29	GCCACGTTGCCTGACCGTCCATGTGGTATGG
core_30	TCATAAAGAGACAGCGATTGGATCGATCAAG

core_31	AAGGAGGAGAAAATGAAATCTTTCTGTGCAAA
core_32	GCGGCGATTGACAGGTGGTGGCATTCTCGTTTTAATAG
core_33	GAGGGCCGCAATAGCATGATACAAAAAGGAGTCCGATCG
core_34	TCCCAATCAGTGCGAAAACTCTCTATCCGC
core_35	GGAAGGGAAGAAAGCGAGCTTGACTCATGA
core_36	AGTTTGGAACAAGAGTCTCGAGTTTGAGATCTTTAAAAGTTCGCCAG
core_37	GTGGACTCCAACGTCAAGGAAGACACCCAATACGGGATGCCATTG
core_38	TCTATCAGGGCGATGGGCCATGCTCACCAGCCGGCGACCCGTCGTT
core_39	ATCACCCTAATCAAGCCGCCGCGAAGGCAAATCAACCAGTTCCCAA
core_40	TGCCGTAAAGCACTAGTAGCGGTGCGACACGCGTAAGACCCCATGT
core_41	GGAGCCCCCGATTTAGAAAGGAGTCCTTTTTCACTGCAT
core_42	CAAAATCCTACCGCTGCCCCAGCACCCCCAGAATAGAATG
core_43	AAATCGGAGATCCTTGCGCAGACAGGGTCAGGTCAGGGTGGTCAC
core_44	ATAGGCCGCTATTAAGTCAGCCAGGCGGGC
core_45	ATTTTTGTTTCCGATCATTGACGTCAATGGG
core_46	AGCGTTAATAAGTAGCTATTGGCGTTACTAT
core_47	CGAAAAGTCACCTAGACCATCTGGGGCACCAGCGCTCCTGGACGTAG
core_48	AGAAAAAGGCCAGGATGTACTGCCAAGTGGG
core_49	AAGGATCTCTTATAAATTGTTCCTCGGTGTAGGTCGTTCGCTCCTGCCATATATGATAGACC
core_50	CAGTTCGAGGGCCGATGATCTTTCTACCGCTCCCATTGACGTCAATGGGGTGGCGGGGGTC
core_51	ATGTAACCTTTAACCAAAAGAACTCTCATAGCTCACGCTGTAGGTAATTGATTA
core_52	CTGATCTTAGATTTATGAACGAATCTGCTTAAAATCCCCGTGAGTCAAACCGCTGGGAACAT
core_53	CAGCATCTCGCGTTAAAAAAACCGCGCCTTTCTCCCTTCGGGAAGCGGGGCGAATAAATCAG
core_54	GTTTCTGGTGATACCGATGAGATTCTGCAGGCCATTGATGTACTGCCAAAACCGGTCAATGG
core_55	GTGAGCAAAATTGTACGTGAACCTTCCGACCCTGCCGCTTACCGGAACGTAGATATTTTGT
core_56	ATGCCGCAGAGGGCTTATCCTTTTTACTTACCATGGTAATAGCGATGACTAATACGCAGTTTA
core_57	AAAAAGGGCCGCGCAGGTCGAGGCCCCCTGGAAGCTCCCTCGTGCG
core_58	GAAATGTTACTCCCCGCAATCTAATGGTCTCCACTGCCAAGTAGGAAAGTCCCATATATGAT
core_59	GAATACTATGTATTTCCCTAAAGCCCGACAGGACTATAAAGATACCCAGCAAAATAAACAAA
core_60	ATTGGAACACCCCACTGCCTGCTATTGTCTGTTGAGTGTCAAAAGAGGCTATG
core_61	GCCACATCTCAGACAGGGAATTCGCCCTTAACCACTATT
core_62	CTTGCCCGATTAGGAATACGTAGCCATGCTCTAAGGGCG
core_63	TGAGAATACAGGGTCATTAATTAAGGCAATGCCCCACTA
core_64	TGTGACTGACAACAGAGCGTAACCACCACACTTTTTTGGCATTTCCC
core_65	TTACTGTCTCGAGGCAGGGCGCTGGCAAGTAATCGGAA
core_66	GTAAGTAGTGCTCATCAAAGGAGCGAGTTCCGCCGTGGCAATAGGGACCTGCCC
core_67	ACGTTGTTAATACCGCACCAGTGGCTGTCCAGCAGCGGGCAAGGCAGACACCTAAGCAGAAC
core_68	GTCACGCTGAGTTGCTCACACCACACGGAATTGTCAGTGCCCAACAGCTCATTTTGCGTCAA
core_69	AGCTCCGAGTCATTCACAACTCCAGGAAAGGACGATGATTTCCCCGGCACCTTCGTGTATG
core_70	CATGATCTGCTTTTCTTTATACGAATCCAGGTGGCAACACAGGCGAAGGGGCAAGTGAGTAC
core_71	TTAGCTCCAATTCTCCGGGAAGAAGGGACGTAGCAGAAGGACGTCAGGCACAGATGCCATC
core_72	TTCGGTCCCAACATAGTTGTGCCCCAGGATGTTGCCGTC
core_73	AGATTACGCTCCATCCGCAGCAGCTTGCCGGTGGTGCAGAGGCGGAGTAAAAGGA
core_74	TCTACGGGTTGGGCGTAACTAGCTAGCTTAATGGCGCTT
core_75	GGTCTGACCTCATTTCACTCGTGTCCTTATCATGCGATGCAATTTCCCGAGCCC
core_76	GCTCAGTGCAGCAATTCGCCCTCCGGGGTAGCGGCTGA
core_77	AACTCACGACGTCATTTTCCTAGAGCATGGCTTACCTGTC
core_78	TTAAGGGATAAAATTTTTACTTTACTTATCAGGACAGTGGGAGTGACAACACC
core_79	TTTTGGTCCGTGACCCCGTTTACGGACTTGAAGAAGTCG
core_80	TATCAAAAAAAGTCCCATGGCGGGTTAATCACTCTCCTG
core_81	AGGATCTTGCCACCTAAAACAGGCTTAATGAGGAAGGCACGGGGGGCAGCCA
core_82	AAATTAAACCGTAAATGGGTGAGCAAAAGGCAGGCGTTT
core_83	AATGAAGTTAGGGGTTAATAAGGCACGCTGCTGGCTGGCAACTAGACCGCGCA
core_84	TTTAAATTCGTGTAGTTGCTCACTGTAGTTGCCGTCGTC
core_85	AGTATATAACACTTGAACCGTAAAAAGGCCGTGGCGAAA
core_86	TGAGTAAATATTTGACATACTCTCGGGCGCTAGATCTCGAAGACGCG
core_87	ACTTGGTCTTTCGTTCTGGAGAGTCGCCCTCGAACTTCA
polyT_1	TTTTTACGAGCATCACAGCCGGCGAACTTTTT
polyT_2	TTTTTAGCCCGACCGCTGCGCACGAACCGCTCTGCGAGATAGG
polyT_3	TTTTTTTACCGTCATTGATTAATCAGTGTTTTT
polyT_4	TTTTTTCGTCTTGAGTCCAACTTATCCAAGCAGCTGAAGCCAGTTACTTTTT
polyT_5	TTTTTGGTGCCTATCAGAAACTCAGTAAGACCAATATTTTT
polyT_6	TTTTTTCGCCACTGGCAAGTGCTGGTAGCGGTGGTTTTTTT
polyT_7	CGGTTCACTTGTCAGTCAGCGATTTGAAGCATTTATTTTT
polyT_8	TTTTTCCCTTCAGCTCGATGCTCCTTGAAGTCGATGTTTTT
polyT_9	TTTTTTTAGCAGAGCGAGGTATGTAGTGGTTGTCGGGCAGCGAGGGTGGAAGCTAGATGCAACTT
polyT_10	TTTTTGTATCCACATAGCGTAAGGCATTCTCATGGTCGCAGAG
polyT_11	TTTTTAGTTCTTGAAGTGGTGGAAAGCGAAAGGGTGCT
polyT_12	TTTTTCCGCGGGAAGGAAGGTCCGCTGGATTGAAGAGGCTATGGCAGCAATATTA

polyT_13	TTTTTCTACACCCCTTGCTGTGGGGGCCTAACTACGGTTTTT
polyT_14	TTTTTGTGGCGAGAAACCGGCAGCAGGTTTTT
polyT_15	TTTTTTTGGTATCTGCCCCCGTTCTTTTT
polyT_16	TTTTTTCAGGGTTATTGTCGGGGAAAAAAATCGACGCTCAAGTCAGAGGCGTTGCTGGCGGATAC
polyT_17	TTTTTCTTCGGAAAAAGCTCCTAGAAGGACAGTATTTTTT
polyT_18	AACGTTCTTCGGGTGGTTAATTGTCAGGTAGGCGGTGCTACAGTTTTT
polyT_19	TTTTTGCAGTGTTATCAAAAGCAGCTTTTT
polyT_20	TTTTTAAACAAACCACCCTATAGCTCTTGATCCGGCTTTTT
polyT_21	TGCCGGGGCCAGGGCACGGGCCACTGGTAACAGGATTTTT
polyT_22	CTGTCTATGACAGTTTCTTCTCTGTACTGGGCATAATGCCAGGCGGGCCATTTTTT
polyT_23	TTTTTAGGCACCTATCAAGTAAGTTGGCCTTTTT
polyT_24	TTTTTTTTTTGTTTGCGGTAACTATTTTT
polyT_25	CGCAGAAATGTTACGACATTGGTAAGACACGACTTATTTTT
polyT_26	ACCAATGCCGTCAATCCATAGGCTCCGCCCCCCTGTTTTT

2.2.5.3 sc_EGFP5 20HB-exLP

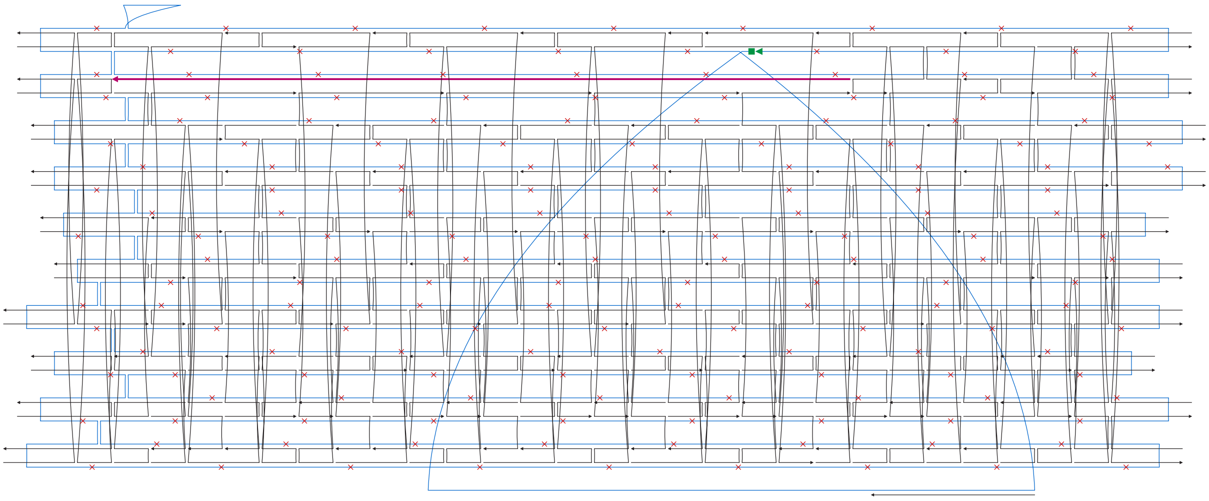

Fig. S35 l Scaffold routing and staple design for sc_EGFP5 20HB-exLP. Scaffold routing is shown in blue, staples are given in black, breakpoint for addition of sequence is marked by a green square (5') and arrow (3'). Long 154-mer 'primer' sequence at the promoter (5') region is given in pink. Design was prepared using caDNAno v0.1.

Table S22 l Individual staple sequences for sc_EGFP5 20HB-exLP.

Name	Sequence 5' – 3'
core_1	TTAACTACCTAATTAAGGGTGAGCAAAAGGCAGGCGTTT
core_2	GCGTTTTTAGGGGGCGTACTTGGCAAGGTCAT
core_3	GGCGGAGTAAAAGGATCTCAAGACAAAATCC
core_4	CCATTTGCGTCAATGGTGAACTTAGTGGTCC
core_5	TATAGACCTCCCACCATCGCCCAAACCAGC
core_6	ATCTGACGGTTCACTCTTGTGGCACGCTCA
core_7	TGCCCAGTGCCTCACACCAGGATGCCCCAG
core_8	TTAAACCTGTCTTGTCCTCGCCCATAACTAC
core_9	GTCTCGACAAGCCCAGGACACCTGATCCATAG
core_10	ACGCCGTAGCTTGCCGTAGGTGGCGTACACGC
core_11	CATGTGGTGCCGGACACGCTGAAAAACCAGC
core_12	GCATGGCGTCGCCGTCCAGCTCGGACCAACT
core_13	AAGATGGTCCCCGGTGAACAGCTAACCTTGAAAATTAAA
core_14	CGGGTCTCATGGCGGCCGCCTGTTTCTAT
core_15	CAGGGTGAAAGGCAAAGTGGATGGCAAGAG
core_16	AGCACGGGTTGCTCAGGGCGGACTGTCCCGG
core_17	GCTGGTAGACCATGTGATCGCGCATGCCCCA
core_18	GTCCTCGGATCCCGGCGGCGGTACACAGTG
core_19	CACCTTGAATTACTTGTACAGCTAACGGGCC
core_20	ATGATATATGTAATCCAGAGGTTAACCAGGA
core_21	ACTCCAGCTTAAGAATACCAGTCAAGCCATA

core_22	GGGGTCTGCCGTCGCCGATGGGGGTGTTCTAGCACTGCTTTGCGCACAGCCGGA
core_23	CCAGCAGGTGGTCGGCGAGCTGCACGCTGCCTGCTGCTTATCGTGGTCCGGCTCC
core_24	CCGAGAGTATGTTGTGGCGGATCTTGAAGTTCCTTCGGCTTCATTCTGCTGCAA
core_25	CCAAGCTTTGCCGTTCTTCTGCTTGTCGGCCCTTGAAGGCGAGTTAGATACGG
core_26	ACAAATTTGACGTTGTGGCTGTTGTAGTTGTCCTCGGCGAAAGCGGTTGCCTG
core_27	GGGTTGCGTCAGCAACACGAACTCTACAGGC
core_28	GCCACGTTGCCTGACCGTCCATGTGGTATGG
core_29	TCATAAAGAGACAGCGATTGGATCGATCAAG
core_30	AAGGAGGAGAAAATGAAATCTTTCTGTGCAAA
core_31	GCGGCGATTGACAGGTGGTGGCATTCTCGTTTTAATAG
core_32	GAGGGCCGCAATAGCATGATACAAAAAGGAGTCCGATCG
core_33	TCCCAATCAGTGCGAAAACTCTCTATCCGC
core_34	TCCTTATCATGCGATGCAATTTCCCGAGCCC
core_35	ACTTATCAGGACAGTGGGAGTGACAACACC
core_36	CTTAATGAGGAAGGCACGGGGGGCAGCCA
core_37	GGAAGGGAAGAAAGCGAGCTTGACTCATGA
core_38	AGTTTGGAACAAGAGTCTCGAGTTTGAGATCTTTAAAAGTTCGCCAG
core_39	GTGGACTCCAACGTCAAGGAAGACACCCAATACGGGATGCCATTG
core_40	TCTATCAGGGCGATGGGCCATGCTCACCAGCCGGCGACCCGTCGTT
core_41	ATCACCCTAATCAAGCCGCCGCGAAGGCAAATCAACCAGTTCCCAA
core_42	TGCCGTAAAGCACTAGTAGCGGTGCGACACGCGTAAGACCCCATGT
core_43	GGAGCCCCCGATTTAGAAAGGAGTCCTTTTTCACTGCAT
core_44	TCAAAAGAGGCTATGAACTAATGACCCCGTATCTCAGT
core_45	AAATCGGAGATCCTTGCGCAGACAGGGTCAGGTCAGGGTGGTCAC
core_46	ATAGGCCGCTATTAATAACTAGCTAGCTTAATGGCGCTT
core_47	ATTTTTGTTTCCGATCTTCCTAGAGCATGGCTTACCTGTC
core_48	AGCGTTAATAAGTAGCATGGCGGGTTAATCACTCTCCTG
core_49	CGAAAAGTCACCTAGACCATCTGGGGCACCAGCGCTCCTGGACGTAG
core_50	AGAAAAAGGCCAGGATGTACTGCCAAGTGGGTAGATGT
core_51	AAGGATCTCTTATAAATTGTTCCTCGGTGTAGGTCGTTCGCTCCTGCCATATATGATAGACC
core_52	TACCGCTGCCCCAGCACCCCCAGAATAGAATG
core_53	CAGTTCGAGGGCCGATGATCTTTCTACCGCTCCCATTGACGTCAATGGGGTGGAGACTTGG
core_54	ATGTAACCTTTAACCAAAAGAACTCTCATAGCTCACGCTGTAGGTAATTGATTA
core_55	CTGATCTTAGATTTATGAACGAATCTGCTTAAAATCCCCGTGAGTCAAACCGCTATCCACGC
core_56	CAGCATCTCGCGTTAAAAAAACCGCGCCTTTCTCCCTTCGGGAAGCGGGGCGAATAAATCAG
core_57	GTTTCTGGTGATACCGATGAGATTCTGCAGGCCATTGATGTACTGCCAAAACCGCATCACC
core_58	GTGAGCAAAATTGTACGTGAACCTTCCGACCCTGCCGCTTACCGGAACGTAGATATTTTGT
core_59	ATGCCGCAGAGGGCTTATCCTTTTTACTTACCATGGTAATAGCGATGACTAATACG
core_60	AAAAAGGGCCGCGCAGGTCGAGGCCCCCTGGAAGCTCCCTCGTGCG
core_61	GAAATGTTACTCCCCGCAATCTAATGGTCTCCACTGCCAAGTAGGAAAGTCCCATATATGAT
core_62	GAATACTATGTATTTCCCTAAAGCCCGACAGGACTATAAAGATACCCAGCAAAATAAACAAA
core_63	ATTGGAACACCCCACTGCCTGCTATTGTCTGTTGAGTG
core_64	GCCACATCTCAGACAGGGAATTCGCCCTTAACCACTATT
core_65	CTTGCCCGATTAGGAATACGTAGCCATGCTCTAAGGGCG
core_66	TGAGAATACAGGGTCATTAATTAAGGCAATGCCCCACTA
core_67	TGTGACTGACAACAGAGCGTAACCACCACACTTTTTTGGCATTTCCC
core_68	TTACTGTCTCGAGGCAGGGCGCTGGCAAGTAATCGGAA
core_69	GTAAGTAGTGCTCATCAAAGGAGCGAGTTCCGCCGTGGCAATAGGGACCTGCCC
core_70	ACGTTGTTAATACCGCACCAGTGGCTGTCCAGCAGCGGGCAAGGCAGACACCTAAGCAGAAC
core_71	GTCACGCTGAGTTGCTCACACCACACGGAATTGTCAGTGCCCAACAGCTCATTTTGCGTCAA
core_72	AGCTCCGAGTCATTCACAACTCCAGGAAAGGACGATGATTTCCCCGGCACCTTCGTGTATG
core_73	CATGATCTGCTTTTCTTTATACGAATCCAGGTGGCAACACAGGCGAAGGGGCAAGTGAGTAC
core_74	TTAGCTCCAATTCTCCGGGAAGAAGGGACGTAGCAGAAGGACGTCAGGCACAGATGCCATC
core_75	TTCGGTCCCAACATAGTTGTGCCCCAGGATGTTGCCGTC
core_76	AGATTACGCTCCATCCGCAGCAGCTTGCCGGTGGTGCAGA
core_77	CGCAGAAATGTTACGAAGTCCCGTTGATTTTGGTGCCAAAACAAAC
core_78	TCTACGGGGTCTGACCTCATTTCACTCGTG
core_79	GCTCAGTGCAGCAATTCGCCCTCCGGGGTAGCGGCTGA
core_80	AACTCACGTTAAGGGATAAAATTTTTACTTT
core_81	TTTTGGTCCGTGACCCCGTTTACGGACTTGAAGAAGTCG
core_82	TATCAAAAAGGATCTTGCCACCTAAAACAGG
core_83	AATGAAGTTAGGGGTTAATAAGGCACGCTGCTGGCTGGCAACTAGACCGCGCA
core_84	TTTAAATTCGTGTAGTTGCTCACTGTAGTTGCCGTCGTC
core_85	AGTATATAACACTTGAACCGTAAAAAGGCCGTGGCGAAA
core_86	TGAGTAAATATTTGACATACTCTCGGGCGCTAGATCTCGAAGACGCG
core_87	ACTTGGTCTTTCGTTCTGGAGAGTCGCCCTCGAACTTCA
core_89	CAGTTTACCGTAAATACTCCACCCATTGACGTCAATGGAAAGTCCCTATTGGCGTTACTATGGGAACATAC GTCATTATTGACGTCAATGGGCGGGGGTCGTTGGGCGGTCAGCCAGGCGGGCCATTTACCGTAAGTTAT GTAACGCGGAACTC

polyT_1	TTTTTACGAGCATCACAGCCGGCGAACTTTTT
polyT_2	TTTTTAGCCCGACCGCTGCGCACGAACCGCTCTGCGAGATAGG
polyT_3	TTTTTTTACCGTCATTGATTAATCAGTGTTTTT
polyT_4	TTTTTTCGTCTTGAGTCCAACTTATCCAAGCAGCTGAAGCCAGTTACTTTTT
polyT_5	CCCTTGGAACATTGGTAAGACACGACTTATTTTT
polyT_6	TTTTTGGTGCCTATCAGAAACTCAGTAAGACCAATATTTTT
polyT_7	TTTTTTCGCCACTGGCAAGTGCTGGTAGCGGTGGTTTTTTT
polyT_8	CGGTTCACTTGTCAGTCAGCGATTTGAAGCATTTATTTTT
polyT_9	TTTTTCCCTTCAGCTCGATGCTCCTTGAAGTCGATGTTTTT
polyT_10	TTTTTTTAGCAGAGCGAGGTATGTAGTGGTTGTCGGGCAGCGAGGGTGGAAGCTAGATGCAACTT
polyT_11	TTTTTGTATCCACATAGCGTAAGGCATTCTCATGGTCGCAGAG
polyT_12	TTTTTAGTTCTTGAAGTGGTGGAAAGCGAAAGGGTGCT
polyT_13	TTTTTCCGCGGGAAGGAAGGTCCGCTGGATTGAAGAGGCTATGGCAGCAATATTA
polyT_14	TTTTTCTACACCCCTTGCTGTGGGGGCCTAACTACGGTTTTT
polyT_15	TTTTTGTGGCGAGAAACCGGCAGCAGGTTTTT
polyT_16	TTTTTTTGGTATCTGCCCCCGTTCTTTTT
polyT_17	TTTTTTCAGGGTTATTGTCGGGGAAAAAAATCGACGCTCAAGTCAGAGGCGTTGCTGGCGGATAC
polyT_18	TTTTTCTTCGGAAAAAGCTCCTAGAAGGACAGTATTTTTT
polyT_19	AACGTTCTTCGGGTGGTTAATTGTCAGGTAGGCGGTGCTACAGTTTTT
polyT_20	TTTTTGCAGTGTTATCAAAAGCAGCTTTTT
polyT_21	TTTTTAAACAAACCACCCTATAGCTCTTGATCCGGCTTTTT
polyT_22	TGCCGGGGCCAGGGCACGGGCCACTGGTAACAGGATTTTT
polyT_23	CTGTCTATGACAGTTTCTTCTCTGTACTGGGCATAATGCCAGGCGGGCCATTTTTT
polyT_24	TTTTTAGGCACCTATCAAGTAAGTTGGCCTTTTT
polyT_25	TTTTTTTTTTGTTTGCGGTAACTATTTTT
polyT_26	ACCAATGCCGTCAATCCATAGGCTCCGCCCCCCTGTTTTT
Loop_1	AAGGAACCCCTAGTGATGGAGTTGGCCACTCCCTCT

2.2.5.4 sc_EGFP5 16HB_2

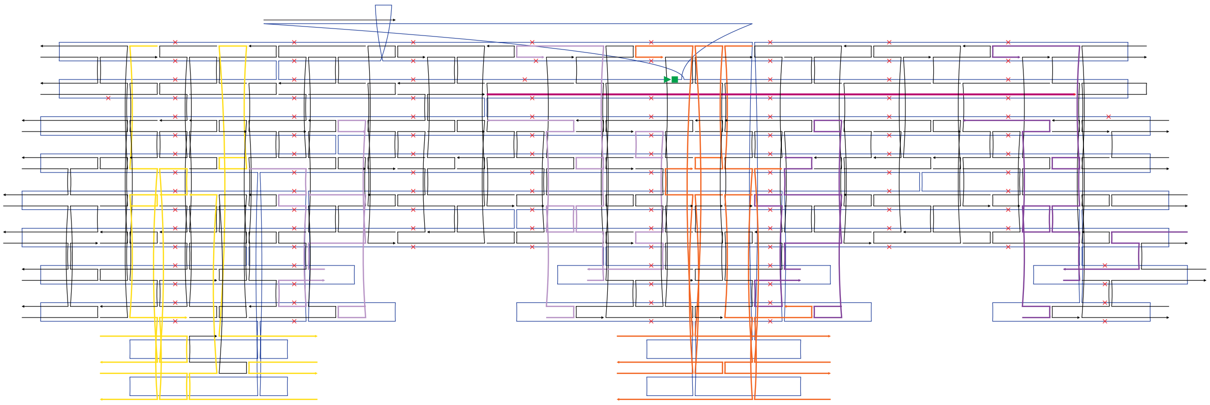

Fig. S36 l Scaffold routing and staple design for sc_EGFP5 16HB_2. Scaffold routing is shown in blue, staples are given in black, breakpoint for addition of sequence is marked by a green square (5') and arrow (3'). Long 154-mer 'primer' sequence at the promoter (5') region is given in pink. Protrusions 1 and 2 are shown in yellow and orange respectively, recess' 1 and 2 are given in light and dark purple, respectively. Design was prepared using caDNAno v0.1.

Table S23 l Individual staple sequences for sc_EGFP5 16HB_2.

Name	Sequence 5' – 3'
core_1	AGAAGATCCTTTGATGGTCATGCACGCTCAATTGTTGGGCAAA
core_2	TTGCAAGCAGCAGATTACGCGCATCGACGCGTTGTTGCGCCAGT
core_3	TTTTTCCGTTCAGCCCGACTCGACCAGGATGGGCACCTTTTT
core_4	TTTTTGAACGAAAACTATATGAGTAAATTTTT
core_5	GGAAGCGTAGATAAGTAGCATGGCGGGTTAATTGGAAAGGAAACGCACACCAGGG
core_6	TTTAAATTAAAAAGGATCTTCACCGAAAAAAA
core_7	CCCCCTGAGAGGTGGCGAAACAGGTTTTTTGTGACTGGTG

core_8	CGCGTTGCGGCGTTTCCCCCTGGACGGCAAAC
core_9	AAGGCCAGGAACCGTAAAAAGGCGACCTCCCTTGTAACCACACCTAC
core_10	GGCTACGTGGCGCTTTCTCATAGCCTTATCGC
core_11	GAATTCCGATCTTCCTAGAGCATCAAACTCCGGATGTCAGCGCGGGTGATCTTAC
core_12	TTAAGGGCCTCAGTTCGGTGTAGGATCGTCTT
core_13	ATTACTATTAATAACTAGCTAGCCTTGGAAAGCCTGGACAGAAGATG
core_14	TTTTTCTTGGTCTGACAGTTACCAATGCTTAAATCAATCAGTCTATTGTCGTTTG
core_15	AGGCACCTATCTCAGCGATCTGTCTAGATCCTAAGTAGTTCCATTGCTCTATTGTCCGTAACCA
core_16	TATTTCGCCACGCTCACCGGCTCGATGAGTAGGGTCAAGGAATTGTTCAAAGGG
core_17	TTCATCCATAGTTGCCTGACTCCCGGCTCCGCGACGGTTCTCACGACCCCTGCCCCAAAGAATA
core_18	CGTCGTGTGGCCCCAGTGCTGCGAAAGCGA
core_19	CATATATGGGCTATTTTTTTTTTTGAACTAATGACCCCACGCCCA
core_20	GCCAGCCGAGCCGGCACAACAGATGAAAGGACTGGCCCACTACGTGAA
core_21	TTTTTCCAAAACCGCATCACCATGGTGCTCATCGCCGAT
core_22	TTTTTCTGCAACTTTATCCGCCTCCATCCTAAAGTATCACGTTAAGGGATTTT
core_23	CAGCTCTGTTACCATCTAGATAACTACGATAC
core_24	CGCCCATTGCCAGCAATCTCCTGTTCCGACCCCCTTCGGAGCCCGGCG
core_25	ACGACATTTCATTAACACCTGTCCGCCTTTCT
core_26	CATTGACCGATGTTATCCAGAGGTTGATTGGATCCAATGCCAAGTGGTGGAGA
core_27	GTCAATGGAGGAAAGTCCCATAAGGTCATGTATTTTGTA
core_28	CCGCTATCCGTAATTGTGGGCTGTGTGCACGAACCCCTTTTT
core_29	CAGTGCCACTAAACCATTTTAAAGTCCCGGAAAGGAGCTGACAGGGGAGGGCCTTATATA
core_30	ACCTGTCACCGTACGCTTGTGAATGCCCCAACCAGTGGGGGTTGATATGATA
core_31	CCCAGTTTTGAAGTCGTTTGTTAATTCCTTTTTCAATATTATTGAAGCAAAAACATCAGCTCG
core_32	CTCTGTCTTACCGGATTACCTAATTAAGGGTGAGCAAAAGTGCGTCAA
core_33	GGCGGCCTCCCCGTTAGTGGTCTTGTACAGCTCGTCCATGCCGAGGTAATAG
core_34	CAGCTCCTCACGAGGGCAGATGAAGCGGCTGAAGCACTGCACTTTTT
core_35	TTTTTACCCCGGTGAATTGATGTACTGTTTTT
core_36	TTTTTCCCAACGATTATCTACGTAGCTTTTT
core_37	GCAAAAAACGTGGTGTAGATTATCAAAAATGAAGTTTTAATCAGTGTAAACCA
core_38	TAAGATGCTGCGCAACTCAAGTCACGAGCATCACAAAAA
core_39	AGTACTCTAAATCAACCCCACCCCCCAGAAATTTCCT
core_40	CATTCTGAAAACCACCGCTGGTAGCGGTGGTACTATAA
core_41	GAATAGTGTATGCGGACCAATACCCCAGG
core_42	TCAATACTTAAATTATGCCCTGGAAGGCCCTGACAACGGGCCA
core_43	TCATCATTCACTGGCAGCAGCCACTGGTAACCCCTTCGCCTATCATCCCGTTGATGCCG
core_44	GGAAAACGTTCTTCGGGGCGGCCAGAGAAAGGTAGGTAT
core_45	GGACACGCGAGTCCAACCCGGTAAGACACGATCACGCTGCAAAGT
core_46	TGAACTTGTGGCCGTCCGTAGGGTGCGCT
core_47	GGCCGAAATCGGCAAAATCCCTTAAACCAAGTTACCTGCCAGATACCATGGCGTTTTTCCATA
core_48	ATCAGCTCATTTTTTACGACCGAGCTCCTTAATCGTGCGC
core_49	TGGCATCGCCCTCGCCCTCAAAACTCTCAAGCTTGTAGTTGCCGTCGCCGTCCT
core_50	CTTCAGGGTCAGCTTGTTACGTCGCTCACCATCTCCAAGC
core_51	TTTTTCAGCTTGCCGGTGGTGTGGGCCAGGGCACGGGTTTTT
core_52	TTTTTCATGCTCTAGGAACTAATCAATTTTT
core_53	TCGGGAATGTATGGCTAGTTACATCGGGGTCTGACGCTCAGTGTTTTT
core_54	AACTCGAAGGGCGAGATGATTTCCCCGAC
core_55	CTCGAACTTTCACCTTGATTTTGGTAATGCCAGGCGGGCCATTTACC
core_56	ACCTTCCTCCGCCGGGGCAAGGCAGGCGGGCTGGATTAACACCACGGAAGGCACATGCCTG
core_57	TTAGGAAAGGACAGTGCTTGCTGTAACTTCTGTTTTCTGT
core_58	TGTGGCTGCCACGTTGAAAATGCCGCAAAAAATCATACTC
core_59	CCATGATAGAGTTGTTCGTCAATAGGGGGCGTACTTGGCCAGCGTAT
core_60	GTGGCGGATCTTGAAGTCACCTCGGTAAGACC
core_61	TTTTTAGATCTCGAAGACAATCCAGGTTTTT
core_62	GGCCGCAGGAAGGGCCGAGCGCAGAAGTGGTCTTTTT
core_63	GCAGGCCAAGGGACGTAGCAGAAGGACGTCCAGCCAAGGGCTGGCA
core_64	GTGGTGGCTCAGACAATGCGATGCATAGAATGTTGATACT
core_65	ATAGTTAATTCTTCTGCATAAAGAAGCGTTTCTGGGTGAGTCCCCGAA
core_66	GAATACCATGAAAGCCATACGGGAAGCAATCAACTCCTCTTGTCGGATGCGGTT
core_67	GCTTATTACGGCGAGCTGCACGCTGTCCTTGAACCTGTGG
core_68	GAGGGCCGGCGGGAAGGAAGGTCCCCAGATTTGCTAGAGT
core_69	AGCATGATGCGTAAAAGGAGCAACGTCATTGA
core_70	TTTTTCGTTGGGGTCTTTTTGTCGGGTTTTT
core_71	TTTTTTGGCAACACAGGCGAGCCGCGCAGGCGGAAGA
core_72	TCCAGCAGGGTGGTGGGCATGGTCGGGGTA
core_73	CGAAAAACGGGAGCCCCCACACCCAAGGAGCGCGGTCCTCCGATCGTTCATGGTTA
core_74	TTTTTGCCGTAGGTCAGGACCATGTGATCGCGCTTCTTTTTT
core_75	TTTTTGTTTTTTGGGGTCGAGCCATCACCGATCCTTAACTAGAAGGCACAGTCGAGGCTTTTT
core_76	GTGCCGTACAATGCGCCATGCTACTCAAGGCGTCATTCAGCTCCGGTTTTTTT

core_77	ACCCTAAACGTCTATCGTCCCCAGCGGGGGAGGCCGGGAAATCAGCAA
core_78	CCGGTGTAGCGGTCAAGTGTTGTACGTGGACTCCTCCCCGGAGTGGCTAATAGTTCGCGTGAC
core_79	AAGGAACCCCTAGTGATGGAGTTGGCCACTCCCTCT
core_80	CAGTTTACCGTAAATACTCCACCCATTGACGTCAATGGAAAGTCCCTATTGGCGTTACTATGGGAACAT ACGTCATTATTGACGTCAATGGGCGGGGGTCGTTGGGCGGTCAGCCAGGCGGGCCATTTACCGTAAG TTATGTAACGCGGAACTC
rec1_1	CGACAAGTGGGGCGTAGACGTCCACATAACAAAGGCATTAAAGCGTCA
rec1_2	CTCTCATGCCATCCGCGCTGCGTTCCCAATCCAACGCAGTGCCCAACA
rec1_3	GCAGCTGGCAATAGGGAGGGGAATGATACCAGGATCT
rec1_4	CCACGTTGTAGTTGTCCTCCTCTATTGGTTTGCTCTTAAAAGAGTTGGTAGCTCTTGATCAGCTCCC
rec1_5	ATTAAAGATCCAGTTTGACCGAGATAGGGTTG
rec1_6	TTGAATACGGGAATAAATGTTGCCGTACTCCAACGCCTACCACTTGATGTACTGCCAAGTGGG
rec2_1	TGTAAGCTGTCGCCTTTCACCGACAGCAACCAG
rec2_2	TTTTTCAGCAGCACGGGGCCGGGTAGTGGGCTCAGGGCGGACTGGAGTG
rec2_3	AGAAAAGTCAATCTTTCACAAACTGGGCATGCCAAAA
rec2_4	CGAACGGGGGTGTTAGCCTTCCGCCCTTGCCGTCCAGCCGCTGCGCCTTATCCGGTAACTTCGTTCG
rec2_5	CTTCAGCAAACCCACTCGCTGTTGAGATCCAGAAAAGTGCAATAGGTG
rec2_6	TCATGTGGCGGACTTGCCTGGACGTCTGCTGGGAGTCAAACGATGACTAATACGTAGATGTAC
pro1_pass_1	CTTTTCTAGATCCCCCTAAGTTGGTTTTT
pro1_pass_2	ACAGGCATGCGGTTAGGGATCTCATGGCAGCACTGCATAATTCTTTTT
pro1_pass_3	TCGCCCTTAATTAAGGAAGCACTAGAACGTGGTTTTT
pro1_pass_4	TTTTTCCGCAGTGTTATCACTGTCAGAAGATGTTGTTAATGTT
pro1_pass_5	TTTTTTCTTACTGCTTGGCGCTAGGGCTTTTT
pro1_pass_6	TTTTTCGAGAAAGGAAGGGAAAAGCCGGCAATCGGA
pro1_pass_7	TTTTTGCTGGCAAATTTAGAGCTTGACGGGGAGAAAGCGAGCCGCGCT
pro2_pass_1	TTTGGTATACATAGCACGGTGCTAATATTTGAATGTATTTAGAAATTTTT
pro2_pass_2	AGAGTCTTACCGCGCCCTGCGCTCCACTAGAAGGACAGTAAGGATTAGCAGAGTTTTT
pro2_pass_3	TTTGTTAAGCGGATACCAGAGTTCTTGAAGTGGTGGCTTTTT
pro2_pass_4	GTTAATATAAGTGCCAGGGTTCCGCGCACATTCATTTATCAGGGTTTTTT
pro2_pass_5	TTTTTCTAACTACGGCTATGCTGAAGCCAGTTATGCCGCT
pro2_pass_6	TTTTTCGAGGTATGTAGGGAACTTT
pro2_pass_7	TTTTTTATTGTCTCATGAAATTCGCGGGGATAAT
pro2_pass_8	TTTTTAATAAACAAATAGCCTAAATTTCGATGTTCTTTTAC
pro1_x8_1	CTTTTCTAGATCCCCCTAAGTTGGAAGCATTT
pro1_x8_2	ACAGGCATGCGGTTAGGGATCTCATGGCAGCACTGCATAATTCTTCGTTCA
pro1_x8_3	TCGCCCTTAATTAAGGAAGCACTAGAACGTGGCGAGTCCC
pro1_x8_4	TATTATTGCCGCAGTGTTATCACTGTCAGAAGATGTTGTTAATGTT
pro1_x8_5	CTGTCTATTCTTACTGCTTGGCGCTAGGGCACCTGCCC
pro1_x8_6	CCTTAACTCGAGAAAGGAAGGGAAAAGCCGGCAATCGGA
pro1_x8_7	TGATACTTGCTGGCAAATTTAGAGCTTGACGGGGAGAAAGCGAGCCGCGCT
pro2_x8_1	TTTGGTATACATAGCACGGTGCTAATATTTGAATGTATTTAGAAAAACTGAGG
pro2_x8_2	AGAGTCTTACCGCGCCCTGCGCTCCACTAGAAGGACAGTAAGGATTAGCAGAGCTACGGCT
pro2_x8_3	TTTGTTAAGCGGATACCAGAGTTCTTGAAGTGGTGGCAGTTTTAA
pro2_x8_4	GTTAATATAAGTGCCAGGGTTCCGCGCACATTCATTTATCAGGGTAATAGGTG
pro2_x8_5	AAAAATGACTAACTACGGCTATGCTGAAGCCAGTTATGCCGCT
pro2_x8_6	TGGCCTAACGAGGTATGTAGGGAACTTT
pro2_x8_7	GTAAGACCTATTGTCTCATGAAATTCGCGGGGATAAT
pro2_x8_8	CGTACATGAATAAACAAATAGCCTAAATTTCGATGTTCTTTTAC
pro1_x5_1	CTTTTCTAGATCCCCCTAAGTTGGAAGCA
pro1_x5_2	ACAGGCATGCGGTTAGGGATCTCATGGCAGCACTGCATAATTCTTCGT
pro1_x5_3	TCGCCCTTAATTAAGGAAGCACTAGAACGTGGCGAGT
pro1_x5_4	TATTGCCGCAGTGTTATCACTGTCAGAAGATGTTGTTAATGTT
pro1_x5_5	TCTATTCTTACTGCTTGGCGCTAGGGCACCTG
pro1_x5_6	TAACTCGAGAAAGGAAGGGAAAAGCCGGCAATCGGA
pro1_x5_7	TACTTGCTGGCAAATTTAGAGCTTGACGGGGAGAAAGCGAGCCGCGCT
pro2_x5_1	TTTGGTATACATAGCACGGTGCTAATATTTGAATGTATTTAGAAAAACTG
pro2_x5_2	AGAGTCTTACCGCGCCCTGCGCTCCACTAGAAGGACAGTAAGGATTAGCAGAGCTACG
pro2_x5_3	TTTGTTAAGCGGATACCAGAGTTCTTGAAGTGGTGGCAGTTT
pro2_x5_4	GTTAATATAAGTGCCAGGGTTCCGCGCACATTCATTTATCAGGGTAATAG
pro2_x5_5	AATGACTAACTACGGCTATGCTGAAGCCAGTTATGCCGCT
pro2_x5_6	CCTAACGAGGTATGTAGGGAACTTT
pro2_x5_7	AGACCTATTGTCTCATGAAATTCGCGGGGATAAT
pro2_x5_8	ACATGAATAAACAAATAGCCTAAATTTCGATGTTCTTTTAC

2.2.5.5 sc_EGFP5 16HB_3

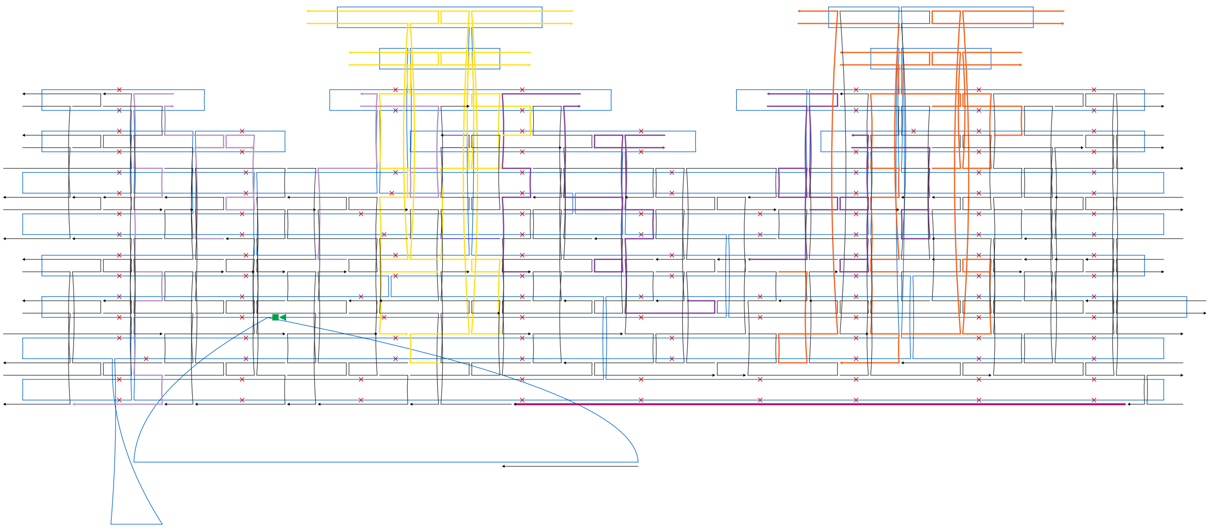

Fig. S37 | Scaffold routing and staple design for sc_EGFP5 16HB_3. Scaffold routing is shown in blue, staples are given in black, breakpoint for addition of sequence is marked by a green square (5') and arrow (3'). Long 154-mer 'primer' sequence at the promoter (5') region is given in pink. Protrusions 1 and 2 are shown in yellow and orange respectively, recess' 1 and 2 are given in light and dark purple, respectively. Design was prepared using caDNAno v0.1.

Table S24 | Individual staple sequences for sc_EGFP5 16HB_3.

Name	Sequence 5' – 3'
core_1	TTTTTAGGACCATAGTTGTACTCCAGTTTTT
core_2	TTTTTCCGAGCGCATCCAGTCTATTATTTTT
core_3	AGAAGTGGCAGCCAGCCGGAAGGGTTTTT
core_4	TCCTGCACCGCGTGAGGCTACACTTCTTGAA
core_5	TTGATTGGATCCAAGCTTCTCTGAGGTCAGG
core_6	CGGCGGCGCGCTTCTCGTTGGGGTCTTTGCTCTCGGCCATGATGCCCTGAGTGGCA
core_7	GTGGATGTCAGTAAGAAGTGATCCTTGAAGTCGATATAGACTAGAAGG
core_8	TTTTTGGAGAGAATTCAGGGTCAGCTTTTTT
core_9	TTTTTGCGCTCTGACAGGATTAGCAGTTTTT
core_10	CTGAAGCCACAGTATTTGGTATCTTTTTT
core_11	CCCGGTGACAGAAACGCAAGAGTCTTATTACTCACCAGG
core_12	TTTTTATTGTTGCCGGGAAGCTAGAGTAAGTAGAATAAAC
core_13	TTAATAGTATCGTGGTGTCACGCTTCTATCAG
core_14	GCCCCAACAAGGAGCTGACAGGTGATCACCCT
core_15	CATACGGGAGAATCCAGGTGGCAGAGCCCC
core_16	CCTTGATGACGCGGAAAATGTTAATATCGGGACGGTCACGCTGCGCGTCGCTGGC
core_17	CGTTGTGGCTGTTGTGTGATCGGTCACGAACTCCAGCTTTTT
core_18	GGTCATGAGGGTCTGATTACCGGAGGCGCTTTCTCATAGCTCACGCT
core_19	TCAGCTCGCCAGGGTGCCTATACAGCTCCTCGCCCTTGCTCACCGCCGGACA
core_20	TTTTTCTTGTGCCCCAGGGGGGGAGGTTTTT
core_21	TTTTTAGCGAGGTATGTAGGCGGTGCCCGCCTCC
core_22	TACAGAGTAGAAGGAGTTACCTTCGGAAAAAGAGTTGGACTTATCGTGGTGGC
core_23	GCAGATTATTGAGTCCTACCTAATTAAGGGTGTAGCTAGC
core_24	CGCTCAGGGGCTGTTGGCGTTTTTCCATAGGCTCCGCGTGCACCC
core_25	TGCTGCTTGTCCTTGAAGAAGATTTAAAGCCGAAGGG
core_26	CACGGGCACGCTGAACAGACCTCCCACCGTACACGCCTACTTGATTTTGTGGTGCACGTCCTCC
core_27	GCTTGCCGGGTGCCAACCTTCCAGACACCTACTGCTATTGTCTTCCCAATCCTCCACAGATG
core_28	GCCACTGGAGATAAGTAGCATGGCTAGAGCAT
core_29	GACCGCTAAGGCCAGGAACCGTAATGACCC
core_30	TTCAGCCCTTTGATCTGCGTTTCTGGGTGCAG
core_31	TCCAAGCTTGGAACGAAACTGATCGAATAAGGGTTAAAATCGATTTAGAGCTTGACGGGGAAAG
core_32	TCGTTCGCGTAGGTATCTCTCCTGTTCCGACCCTAAAGTAACGTTAAG
core_33	CTTGATACTTACGTCAGAGGTGGCGAAACCCAGAAGTCGAAAACCGC
core_34	CAGCTCGATGACGGTTCACTAAACCAGCTCTGACGTCAATGAGGGTGGGATGCGGT
core_35	TTTTTTGCCGTAGGTGGCTTGCGTCAATGGGGCGGAGTTTTT

core_36	CTACAGGCTTGCGCAACGTTGTTGTGGCAATCGGGAGGGAAAGCGGT
core_37	AAGCGAAACCGATCGTCCCGTCGTATCTCAAGAAGATCC
core_38	ACGTAGCACCGGCGAACTAAATTGTAAGCGTTCCAATAGCGATGACTAATACGTAGATGTACT
core_39	GCAGCAGGGGCCGTCGCCGATGGCGGGCAG
core_40	CACAGTCGAGCATGCCTCAGACAATGCGATGTCATTGA
core_41	TTTTTGGCAAACACCCTTGCTGTCCTTTTTT
core_42	TTTTTAGCTCCGGATTAAAGAACGTGTTTTT
core_43	GCGACACGTCCCGCGCAAGCAATAGCATGATACAAAGGCAGGTGCGCT
core_44	GAATACTCATCACCATGGTACTCCCCCCTGAGGTGTAGG
core_45	ATACTCTGTGCCACCGTGGCGAGAAAGGAAGGGAAGACCGCTGGA
core_46	CAATATTATTGCCGTCCATGTGGTCGGGGTA
core_47	TTGAAGCATCCACGCGGAAAGTCCCATAAGGTCATGTAAAATAAACGGTTATTG
core_48	AAGGCACATGTTGCGATGAACAGGCAAACGCCTGGACACCTGTTTTTT
core_49	CATAATTCCATGATCCCGAGATAGGGCGATGGCCCACTACGTGAACCGCCATTGGTGCAAA
core_50	CACTCATGTTTAAAAGTTAAGGGCGAATTCCGATCTTCC
core_51	AGTGTTATTAGCTCCTGCAAAATCAATCAAGTTTTTTGGGGTCGAGGT
core_52	TTCAGCAGTTGAGATCCAGTTCGATGTAACAATATTTT
core_53	CCATTGATCAACTGCCCAGTGCCTCACGACCAACTTCTGGTGAGTCA
core_54	GACTTGGATAATGCCAGGCGGGCCATTTACCGCAATTTCCTCATTATT
core_55	GGGGTGGACATTTATTAGGAAAGGTCTCGAAG
core_56	CCCATTGCTTATATTTGTGGCCGTTTACG
core_57	AACAAACTCGTCAATAGGGGGCGTACTTGGCAATAGAATGGGTCAAGGGCTGGCAA
core_58	TTTTTTTGTTACGACATTTTGGAACACTTGATGTGGG
core_59	TTTTTTCTGTGACATAGTGTATGCGGTTTTT
core_60	TGGTGAGTATCCGTAAGATGCTTTTTTTT
core_61	ACTCAACCAGCAGCCACTGGTA
core_62	GGGTTAATCAGCACTGTTTTGTTTTCTGGCCCCAGTGCTG
core_63	AGCAAAAGTTGGCCGCAAAAAAGGGTAGATAACTACGATA
core_64	GCCAGCAAGCGCCTTATCCGGTAACTATCGTCCGCGCAGA
core_65	CGCCCATATCGCCCTCGCCCTCATGGCGGC
core_66	TTTTTGACTCCAACGTCAAAGGGCGAAAAACCGCGTCGTTGATCAAGACTTTAT
core_67	AAGCGAAAGTTCCGCGCACATTTCGCCAAGTA
core_68	GGTTGAGTGTTGAAGGCGTCAATCATGCC
core_69	CCTTATAAATCAAAAGCAGAACGTTATGGCATTAACAACCCGG
core_70	TTTTAACCAATAGGCACGTTCTAAGTAAG
core_71	TCGCGTTAAATTTTTTTACCGCTTCTTTTACCGCGTTGC
core_72	CCCGAAAATCCTTTTTTTGAGGGCAGCGTATCCACATAGCGTAAAAGGTCTTGTAG
core_73	TTTTTGCCCCACCCCACCCCCCAGATATGATAAGTCCCG
core_74	TTTTTCGACCGAGTTGCTCTTGCCCGAGTCCACTTTCCCAACTGGTATGGCTTCATTCTTTTT
core_75	AATACCGCGCCACATAGAATAGACCCCATGTT
core_76	TGCTCATCATTGGAAACGAAATCGTCGGTCCT
core_77	TCGGGGCGCGTAATTGATTACTATTAATAAC
core_78	AAAACTCTCAAGGATCGTTAAATCAAGGCAAA
core_79	CTGGGCAAATCCCCCAGGATCTCTCCTTAAACCTGTCTTGTAACGCGGCTGAAACCGCTA
core_80	CATATATGGGCTATGAACTAAAAAGGCTTTCACCATTTCTACGGATTATCATAAAAATG
core_81	TTTTTGCCAAGTACTTTTTT
core_82	AAGGAACCCCTAGTGATGGAGTTGGCCACTCCCTCT
core_96	CAGTTTACCGTAAATACTCCACCCATTGACGTCAATGGAAAGTCCCTATTGGCGTTACTATGGGAACA TACGTCATTATTGACGTCAATGGGCGGGGGTCGTTGGGCGGTCAGCCAGGCGGGCCATTTACCGTA AGTTATGTAACGCGGAACTC
rec1_1	GATTTATCAGCTTCGCCAGCAATGATAGCGAGTTATCTTACTGTACGGGATGGCTACGTCAAGTCATT CTGAGA
rec1_2	GACTCTAGATCCTTTTAAATAAAAGGATAGGATTTA
rec1_3	CTTACCAGCAAGCATGGTAGCTGATCCG
rec1_4	TAAGACACGTAGCTCTGGTGGTTTCTAACTACCCC
rec1_5	GTGCACGAACCCCCCGGGAAGCGTTACCTGTC
rec1_6	GTCCCGGACAGTGGGGGTTGCGTCTTCGT
rec2_1	ACAGTTACCAATGCTTAATCTGAAAGCGGATTTTAAAAAGG
rec2_2	AAATTTTGTAAGGCGCGGGAGCAACATCCGCGGGAAGGAAGGT
rec2_3	TGGAAGCTCCGACTTGAGACAGGACGAGCATCACAAAAATCGACGCTGTACTGCC
rec2_4	TTGGCCAGGATGGGCACCACTCGCCGTCGTGGTCAC
rec2_5	AAATTCGGGCATGGCGCTCGTGCGCTCAGTTCCTATAAAGAT
rec2_6	AGCACTGCTTCACCTCTCCAGAGGCAGCACGGAGTTAAGAATA
rec2_7	GAAGGACGGAAATGTTCCTGGACGTAGCCCTCTATAT
rec2_8	AGAGCCGTCTCATGCTCGAACACGCCGTTCTCG
pro1_pass_1	TTTTTGATGATTTCCCCGACAACACCTAAAGGACACAGGCATGCCGCA
pro1_pass_2	TTTTTGTCCAGCAGCGGGCAAGGCAGGGGGAGCCGTAA
pro1_pass_3	TTTTTACTCCTCAGGAGAAAAAGTGAGGCAAGTTTTAAATCAATCTGCCGC
pro1_pass_4	TTTTTCACCACGCTGTCTATAGCAAACCTTCACCTGTAGCAAAAACAGG

pro1_pass_5	ACCTATCTCAGCGATCCACGTTGCCAGCAACCACAGTGCATTTTT
pro1_pass_6	CAATAGGGAGGCGGCGTTGTCAGTGCCCAACAGCCGAGCCCCTTTTTT
pro1_pass_7	CGCCGTGGTACAAGGATAAAGAGACTGACAACGGGCCACATTTTT
pro1_pass_8	AGCTCATTAGCACTAAATCGGAACCCACGGAAATGAGTTCGAGCAGCCAAGGAAAGGACTTTTT
pro2_pass_1	TTTTTTACGTAGCCATGCTCTAGGAGTCCCCAGGCAGAACAGTGG
pro2_pass_2	TTTTTCACCACACCCGCCGCGCTTGAGGCCGCAAGTGTAGTGAATGTATTTAGAA
pro2_pass_3	TTTTTTGTTGTGGCTGCTTGAGGGCGGCTCGTCCATGCCGAGCCAATAGG
pro2_pass_4	TTTTTGTGGTCGGGTGGTTGTGGGTGTTCGTGTCGCCAGCGGATA
pro2_pass_5	TTGTACAGACTGGGTGCTCAGGTACGAGCTGCGAAGTTCATGCTGGTATTTTT
pro2_pass_6	CCGTTCTTCGGATCTTACGCTGCCGTCCTCGATTTTT
pro2_pass_7	TTTATCAGAAATAGGGGGAGCGGGCGCTAGGGAACTTTTT
pro2_pass_8	ATTCGCCCTTAACTCGAAGATCCTTTAAGGCAATGCGCCATGCTACTTATCTTTTT
pro1_x8_1	GCAAACACGATGATTTCCCCGACAACACCTAAAGGACACAGGCATGCCGCA
pro1_x8_2	GCCGAGCCGTCCAGCAGCGGGCAAGGCAGGGGGAGCCGTAA
pro1_x8_3	GGGCGACAACTCCTCAGGAGAAAAAGTGAGGCAAGTTTTAAATCAATCTGCCGC
pro1_x8_4	GGAACAAGCACCACGCTGTCTATAGCAAACCTTCACCTGTAGCAAAAACAGG
pro1_x8_5	ACCTATCTCAGCGATCCACGTTGCCAGCAACCACAGTGCAAGTCCACT
pro1_x8_6	CAATAGGGAGGCGGCGTTGTCAGTGCCCAACAGCCGAGCCCCTCCTGTCCA
pro1_x8_7	CGCCGTGGTACAAGGATAAAGAGACTGACAACGGGCCACACGGAAATG
pro1_x8_8	AGCTCATTAGCACTAAATCGGAACCCACGGAAATGAGTTCGAGCAGCCAAGGAAAGGACAGTGCACA
pro2_x8_1	ATCCCGGCTACGTAGCCATGCTCTAGGAGTCCCCAGGCAGAACAGTGG
pro2_x8_2	GATTTATACACCACACCCGCCGCGCTTGAGGCCGCAAGTGTAGTGAATGTATTTAGAA
pro2_x8_3	AAGAAGTCTGTTGTGGCTGCTTGAGGGCGGCTCGTCCATGCCGAGCCAATAGG
pro2_x8_4	CGTGCACCGTGGTCGGGTGGTTGTGGGTGTTCGTGTCGCCAGCGGATA
pro2_x8_5	TTGTACAGACTGGGTGCTCAGGTACGAGCTGCGAAGTTCATGCTGGTACAACTGAT
pro2_x8_6	CCGTTCTTCGGATCTTACGCTGCCGTCCTCGAGTGCTGCT
pro2_x8_7	TTTATCAGAAATAGGGGGAGCGGGCGCTAGGGAACCAAGGAGG
pro2_x8_8	ATTCGCCCTTAACTCGAAGATCCTTTAAGGCAATGCGCCATGCTACTTATCGGCGGTCA
pro1_x5_1	AACACGATGATTTCCCCGACAACACCTAAAGGACACAGGCATGCCGCA
pro1_x5_2	GAGCCGTCCAGCAGCGGGCAAGGCAGGGGGAGCCGTAA
pro1_x5_3	CGACAACTCCTCAGGAGAAAAAGTGAGGCAAGTTTTAAATCAATCTGCCGC
pro1_x5_4	ACAAGCACCACGCTGTCTATAGCAAACCTTCACCTGTAGCAAAAACAGG
pro1_x5_5	ACCTATCTCAGCGATCCACGTTGCCAGCAACCACAGTGCAAGTCC
pro1_x5_6	CAATAGGGAGGCGGCGTTGTCAGTGCCCAACAGCCGAGCCCCTCCTGT
pro1_x5_7	CGCCGTGGTACAAGGATAAAGAGACTGACAACGGGCCACACGGAA
pro1_x5_8	AGCTCATTAGCACTAAATCGGAACCCACGGAAATGAGTTCGAGCAGCCAAGGAAAGGACAGTGC
pro2_x5_1	CCGGCTACGTAGCCATGCTCTAGGAGTCCCCAGGCAGAACAGTGG
pro2_x5_2	TTATACACCACACCCGCCGCGCTTGAGGCCGCAAGTGTAGTGAATGTATTTAGAA
pro2_x5_3	AAGTCTGTTGTGGCTGCTTGAGGGCGGCTCGTCCATGCCGAGCCAATAGG
pro2_x5_4	GCACCGTGGTCGGGTGGTTGTGGGTGTTCGTGTCGCCAGCGGATA
pro2_x5_5	TTGTACAGACTGGGTGCTCAGGTACGAGCTGCGAAGTTCATGCTGGTACAACT
pro2_x5_6	CCGTTCTTCGGATCTTACGCTGCCGTCCTCGAGTGCT
pro2_x5_7	TTTATCAGAAATAGGGGGAGCGGGCGCTAGGGAACCAAGG
pro2_x5_8	ATTCGCCCTTAACTCGAAGATCCTTTAAGGCAATGCGCCATGCTACTTATCGGCGG

2.2.5.6 sc_EGFP5 16HB_4

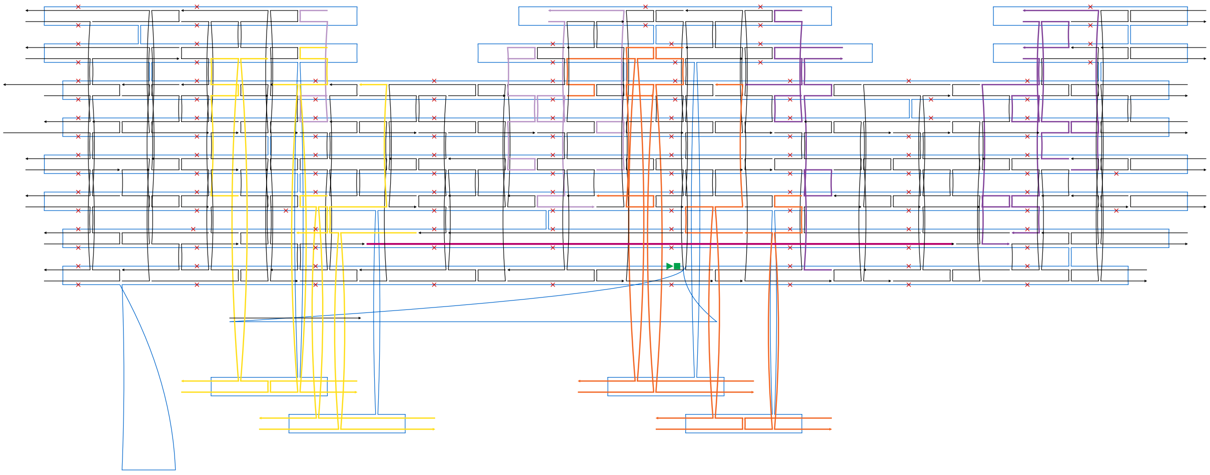

Fig. S38 | Scaffold routing and staple design for sc_EGFP5 16HB_4. Scaffold routing is shown in blue, staples are given in black, breakpoint for addition of sequence is marked by a green square (5') and arrow (3'). Long 154-mer 'primer' sequence at the promoter (5') region is given in pink. Protrusions 1 and 2 are shown in yellow and orange respectively, recess' 1 and 2 are given in light and dark purple, respectively. Design was prepared using caDNAno v0.1.

Table S25 | Individual staple sequences for sc_EGFP5 16HB_4.

Name	Sequence 5' – 3'
core_1	TTTTTATCACCCTAATCAAGTGCCCACTACGTGAACCTTTTT
core_2	ATACTTACGTCCTCGAGGTCGGCGCGATGGGGGTGTTCTGGTGCACAC
core_3	GTCAGCTTTTGTAATCAAGGAGCAACATAGTCACGCCAGCGTCAG
core_4	TTTTTTTTTCTGTGACTGGTGGCCATCCGTAAGATGCTTTTT
core_5	CTCACGCTGTAGGTATCTCAGTTTTGCTGGCCGTTTGGTGGGAAGCT
core_6	TTTTTAAGGCAAAGTGGGCGTTTCTTTTT
core_7	GTCGTTCGCTCCAAGCTGGGCTGAGCAAAAGCCAACGATTCCGCCTC
core_8	TTTTTCGGGGCCGTCGCAGCTGCATTTTTTTTTTTTTTTT
core_9	GCCACAACTATCCACATCTGCTTGGCAGATGACTGTCTCGTACTTTCATCCCTTCG
core_10	GGCAACAGGAAGGTTTTTTCAATATTATT
core_11	CTACTCACTTCCAGGCCCTCGACGGGCATGCGGTTAGC
core_12	CACCACACAATCAAAAGAATAGACTTAACCAAATGGTTATTATGCGGC
core_13	GATGATTAACAGCCGCGATGAGTTCCGCCAATCCCCGTCTATTAA
core_14	TAGCGTAACAGAGGTTGATTGGATCGAGAGTGCCAGCGTT
core_15	CCGCCGCGCTTAATGTTAATTAAAACTTGGTCGCTCACCGGCTCCAG
core_16	TTTTTGTCTATCAGGGCGATGCAAAGGGCGAAAAACCTTTTT
core_17	TTTTTACTATTAAAGAACGTGGACTTCGTTC
core_18	TTTTTCGCTGCCCTGCCCAGTGCCTTTTT
core_19	TGTTGTGGCCAAGGTAGTGGTTGTCGGGCAGCAGCATTTTT
core_20	ACGTTGTGGCTGTTGTTGGGCCAGTGGTGTCACTCGTGCA
core_21	CTCCAGCTAGGGAGGGGGAAAGCGAAAGTCCCCCATTGA
core_22	CGCGGGAAGGCAAACACGTGCTGTCGATGCGCAGAAGAGTGGAA
core_23	ACAAATTGCCGTAGGAAGTTCCTTAAACAACCAGC
core_24	TAAGAATACGGATCTTGTGGCATCTAGGTGCCCTCACCAT
core_25	CAAACACAGTTCACTACTGTCTTGGACGTCAACCGACAGGACTATAAA
core_26	GGCAATGCGGGTCTTGTAGTTGCCGTCGTATCTGACCCACTGACAG
core_27	TTTAGAGCTTGACGGTTTTTGGGATTGTAAGCGTTAATATTTTTTTT
core_28	ACTTCAGGCACCACCCATCCCGGCGGCGGTCACGAACTC
core_29	GGGTTGAGGTAGCGGTCGGGCGCTAGGGCGCTCCAACGTGGAAAGCC
core_30	GGACTGGGTGCTCGTCAATCTTTCCAGCAGGACCGTCCAGCTCGACC
core_31	AATCGGCAAACCTTGAAGAAGATCAGAAGTATCAATACGGTAAGACA
core_32	TTTTTTGTTAAAATTCAACAAGAGTCCTTTTT
core_33	TTTTTTTTTTTTTTTTTGAACTTCCTGTGGAGAGATTTTT
core_34	TCTGGGTCATCTTTTGTTGCCATCACAAATGTACTGCCAAGTGGG
core_35	CCGCAAATAACCCACGCTCGTGTTTTTCCATAGGCTCCGCCCCCC
core_36	AAAGGGAAGGGTGGTCACGAGGGAGTTGTAAGAGTAACGTCAATGTACTGC

core_37	ATGTTGAGATCTTACCGGTTCGCCAGGAACCGTAAAA
core_38	TCATGAGCATAGCATCCTTCGTAAGTAGCATGGCGGGTTAATCATGCGCCTT
core_39	GAAAAATCCCGGCGAGTTGGCTTCCGATCTTCCTAG
core_40	GCCCTTGTATCAGAGCGAAACTAGGGGGCGTACTTG
core_41	GGCGAAAACTCTCAAGATACTCATTAGCGGCTCGCGCAGAGAAAGGAC
core_42	GAAAACGATCCCCCCTAATTAAGGGTGAGCAAAAGGCCTGTGCACG
core_43	AAAGTGCTCATCATTGGAAGCATTGAAGAAGTACAGATGGAAGGCACG
core_44	TTTTTTTTGCTCAGGGCGACACGCTTTTT
core_45	AATAGTGGGCAGCAACTATTAAACTAATGACCCCGTTCTGCTGA
core_46	TTCCGACCGGCGGCCGCCTGGACAGTGGCCGTCCTCGCCG
core_47	CCACTGGTAACAGGATTAGCAGAAATTGATTCTGCATAAGGGCTTAC
core_48	CTGCCAAGTCTGCTTATATAGACCCCAACCAGGCCGTTCT
core_49	CAGTTTGGGCGTTAAACTGTCATAGTACTCATGTAGGCGTTTTT
core_50	ATCGTTGTGGTGCGCTTATTTGAATGTATTTA
core_51	GCCCTCGCTTACGTCGCCATGTGATCGCGCTTCTCGTTGGGGTCTTTTT
core_52	GGAAGCGTGGCGCTTTGACGAGCATTGCTACTAGTTTGCTGATATAGAGCTGACA
core_53	CGACTTATCGCCACTAGGGCGAACGCAGTGTATACCGCGCCTTATA
core_54	AGGGTGTCGGTCAAGGCTGGCAACTAGAAGGAGGCCGCTTATTGTC
core_55	GTAGTTCGCCAGTTAAAGGCATCGGGCACGGGACAGGAAGGCAAAATG
core_56	TTGTTGCCATGGCTTCGTAGGTCATAAGGGCGACACGGAA
core_57	CTTCATGTGTTACATGTTCTTCGGAACCCCCCGTTCAGCCCGACCGC
core_58	TAACTACGGGCCTAACTACGGCTACACTTTTT
core_59	TTTTTCTCCCCGTCGTGTAGAATCCATAGTTGCCTGATTTTT
core_60	TTTTTTCACGACCAACTATAATGCTTTTT
core_61	TCCCACCACAAGCCCTACACTTGAAATCGAC
core_62	GGGGTGGAGACTTGGAGTGGCAATTGTGCCCC
core_63	ACGGGGTCGCGGGCAAGTCGATGCCAGCCAAGATCCAGGT
core_64	GGGATTTTACCAGCCAGCCGGAAGCAAAAAAG
core_65	TCAGCGATCTGTCTATTGGCAAGTTGTTGTTCGGCGAAC
core_66	TAGGAAAGTCCCATAAGGTCATGCCGTCATTTAACCTTGAAGACCAA
core_67	CTGCCGCTTACCGGATACCTGTCCAGAGGTGAACGCAAG
core_68	TAATACGTAGATGTAGCATATGAAGTTTCTAAGTCTTCT
core_69	GCGGACTTTATCAGGGAGAGCCGGCAGCAGGC
core_70	TTACGCGCAGAAAAAAAGGATCTCAAGAAGATCCTGTCAAACCGCTATCCACGCCCATTGAT
core_71	GAACTTTAATCCGGTAACTATCGTCTTGAGT
core_72	CTCTTGATCCGGCAAACAAACCACCGCTGGTAAAGTATATATGAGTA
core_73	TACCTTCGGAAAAAGAGTTGGTAGTTACCAATGCTTAATC
core_74	TTTTTTAGAAGGACAGTATTTGGTATCTGCGCGAAGTGGTATACGGGA
core_75	TTTTTCAGGCGGGCCATTTATACTGGGCTCTGCAGGATCTGACGCTGGTAGT
core_76	TCTCATAGCCCAACTGATCTTCAGGAGCAAAACAGCTTGC
core_77	CATATATCTGCAATGTATCACTCTAGGCCGAAGGGGTTCCGCGCACATTTCCCC
core_78	GCTCAAGTCGCCTTTCCGGTGAACAGCTCCTCAGGATGGG
core_79	AGGCCGCGCGGTGTAGGAGATCCAGTTCGATG
core_80	TTAACTACATGTTGTGGGCCGAGCGGTTCACCAGGAAGATCCCAATAGAATGACAC
core_81	ATAACTAGCTAGCTTAGGCAGCAGGACCGAGTTGCTCTTGAAACAAAT
core_82	AGCATGGCCCAACCCGGGATAATACCGCGCCACGGATACACCTGGACG
core_83	TTTTTGTGCTACAGAGTTCTTGCGAGGTAACCAAGTC
core_84	TTTTTCCCCTGGAAGCTCCCGATACCAGATGTCAGT
core_85	AAGGAACCCCTAGTGATGGAGTTGGCCACTCCCTCT
core_96	CAGTTTACCGTAAATACTCCACCCATTGACGTCAATGGAAAGTCCCTATTGGCGTTACTATGGGAACA TACGTCATTATTGACGTCAATGGGCGGGGGTCGTTGGGCGGTCAGCCAGGCGGGCCATTTACCGTA AGTTATGTAACGCGGAACTC
rec1_1	CAAGGCGAGGTCGGGGACTCTTCCCCGCTGGATTGAGGGCCGAAG
rec1_2	CGTCCGAAGCACGCCGTCCCATCCAGTGATTGATCTTTTCT
rec1_3	CAGTGCCCTCCCCGACAGGATGTTTGCACGCCATTCAGCTCCGCTGTT
rec1_4	GTCCATGCCCAAGCTTCGGTGGTTCGGCCA
rec2_1	ATTCTGAGGAAAAGTGAAATCAGCGCCCCCGAGTGGCGAGAAAGG
rec2_2	AGGACAGTGGGAGTGGCACGACAATGCACTTATCTACGTAGC
rec2_3	CGCGGAAGCACAGTCGTAGCCTTACTTCAC
rec2_4	AGGGATCATTTTCGAGATACATCTGGACCTATCAGCCAGT
rec2_5	TTCTCTTATTTTTGTTCCACCTAAGTCGAGGTGCCGTAAAGCAC
rec2_6	AGGAGCACGCTGCGCGTAACAGTGAGGCCCCCAGTGGGGCTATG
rec2_7	TACGTAGAGTCCTCCGATTTATCACTCGGCGCGCCATGCTGATGCAATTTC
pro1_pass_1	TTGGTCTCACCTTGATTGGGGGTTCGTTGCCTGCAACCAGTTTTT
pro1_pass_2	CAAAACCGCATCACCATGGTATCCCGTTGATTTTGGTGCTTTTT
pro1_pass_3	TTTTTGACATTTTGGAAAGATACTACCGCCCATTTACTCGGAAAGG
pro1_pass_4	TTTTTACGGGAAGTCCTCATAAGAAAATGGCAATGCC
pro1_pass_5	AAGCAGCGCAATAGCAGGTGGTGAAAGCCATTTTTT
pro1_pass_6	TTTTTCAAAACAAGCGTCAATGGGGCGGACGCGCGATGAC

pro1_pass_7	TTTTTGATTTATACAAGGAGGAAGAGACAGACAACGG
pro1_pass_8	GCAACGTGTACAGTTGTTACTTTTT
pro2_pass_1	GCAATAATCAATCTAGCGGTGAAAATGAATTTTT
pro2_pass_2	GGGGAGGCTCGAGTCCTTATCGCTGTCCTGCCCCACCCCATTTTT
pro2_pass_3	TCCTTGAAGGCAGGCGGAGCCCCTGTCCAGCAGCCGTCTTCCCTTTTT
pro2_pass_4	TTTTTCTTCACCTAGATCCTTGATTATCACACGTTAACATGCTCT
pro2_pass_5	TTTTTAATCCTCCCCCTTGGGAATTCTGACGCTCTGGTCCTGCAACTTTA
pro2_pass_6	TTTTTGTTTTAAAGGTCATGATTAAATTAGTTTTTTT
pro2_pass_7	TTTTTCCCCCCAGCAGCATGCCTGCTATTCTTAACAGGCGAGCCTTCAGC
pro2_pass_8	GTTTGCAAGCAGCAGACGAAAACTAAAAGGATTTTTT
pro1_x8_1	TTGGTCTCACCTTGATTGGGGGTTCGTTGCCTGCAACCAGCGGCTCCA
pro1_x8_2	CAAAACCGCATCACCATGGTATCCCGTTGATTTTGGTGCTCCCTTCG
pro1_x8_3	GCAAACAAGACATTTTGGAAAGATACTACCGCCCATTTACTCGGAAAGG
pro1_x8_4	TCATCCATACGGGAAGTCCTCATAAGAAAATGGCAATGCC
pro1_x8_5	AAGCAGCGCAATAGCAGGTGGTGAAAGCCATAGTTGCCT
pro1_x8_6	CGCCTTTCCAAAACAAGCGTCAATGGGGCGGACGCGCGATGAC
pro1_x8_7	ACGCTCACGATTTATACAAGGAGGAAGAGACAGACAACGG
pro1_x8_8	GCAACGTGTACAGTTGTTACACCACCGC
pro2_x8_1	GCAATAATCAATCTAGCGGTGAAAATGAAAGTTTCTA
pro2_x8_2	GGGGAGGCTCGAGTCCTTATCGCTGTCCTGCCCCACCCCATCTTTCAC
pro2_x8_3	TCCTTGAAGGCAGGCGGAGCCCCTGTCCAGCAGCCGTCTTCCCTTGGTCTG
pro2_x8_4	ACCAGGCGCTTCACCTAGATCCTTGATTATCACACGTTAACATGCTCT
pro2_x8_5	GAGTAAACAATCCTCCCCCTTGGGAATTCTGACGCTCTGGTCCTGCAACTTTA
pro2_x8_6	ACAAGCCCGTTTTAAAGGTCATGATTAAATTAGTTTTTTT
pro2_x8_7	CCAGTCAACCCCCCCAGCAGCATGCCTGCTATTCTTAACAGGCGAGCCTTCAGC
pro2_x8_8	GTTTGCAAGCAGCAGACGAAAACTAAAAGGATTTTCCCCC
pro1_x5_1	TTGGTCTCACCTTGATTGGGGGTTCGTTGCCTGCAACCAGCGGCT
pro1_x5_2	CAAAACCGCATCACCATGGTATCCCGTTGATTTTGGTGCTCCCT
pro1_x5_3	AACAAGACATTTTGGAAAGATACTACCGCCCATTTACTCGGAAAGG
pro1_x5_4	TCCATACGGGAAGTCCTCATAAGAAAATGGCAATGCC
pro1_x5_5	AAGCAGCGCAATAGCAGGTGGTGAAAGCCATAGTTG
pro1_x5_6	CTTTCCAAAACAAGCGTCAATGGGGCGGACGCGCGATGAC
pro1_x5_7	CTCACGATTTATACAAGGAGGAAGAGACAGACAACGG
pro1_x5_8	GCAACGTGTACAGTTGTTACACCAC
pro2_x5_1	GCAATAATCAATCTAGCGGTGAAAATGAAAGTTT
pro2_x5_2	GGGGAGGCTCGAGTCCTTATCGCTGTCCTGCCCCACCCCATCTTT
pro2_x5_3	TCCTTGAAGGCAGGCGGAGCCCCTGTCCAGCAGCCGTCTTCCCTTGGT
pro2_x5_4	AGGCGCTTCACCTAGATCCTTGATTATCACACGTTAACATGCTCT
pro2_x5_5	TAAACAATCCTCCCCCTTGGGAATTCTGACGCTCTGGTCCTGCAACTTTA
pro2_x5_6	AGCCCGTTTTAAAGGTCATGATTAAATTAGTTTTTTT
pro2_x5_7	GTCAACCCCCCAGCAGCATGCCTGCTATTCTTAACAGGCGAGCCTTCAGC
pro2_x5_8	GTTTGCAAGCAGCAGACGAAAACTAAAAGGATTTTCC

2.2.6 sc_EGFP6

Scaffold sequence for sc_EGFP6:
attaggggcctcagtgagcgagcgagcgcgcagagagggagtggccaactccatcactaggggttccttgtagttaatgattaacccgccat
gctacttatctacgtagccatgctctaggaagatcggaattcgcccttaagctagctagttattaatagtaatcaattacggggtcattagttcatag
cccatatatggagttccgcgttacataacttacggtaaatggcccgcctggctgaccgcccaacgacccccgcccattgacgtcaataatgac
gtatgttcccatagtaacgccaatagggactttccattgacgtcaatgggtggagtatttacggtaaactgcccacttggcagtacatcaagtgtat
catatgccaagtacgccccctattgacgtcaatgacggtaaatggcccgcctggcattatgcccagtacatgaccttatgggactttcctacttgg
cagtacatctacgtattagtcatcgctattaccatggtgatgcggttttggcagtacatcaatgggcgtggatagcggtttgactcacggggatttcc
aagtctccaccccattgacgtcaatgggagtttgttttggcaccaaaatcaacgggactttccaaaatgtcgtaacaactccgccccattgacgc
aaatgggcggtaggcgtgtacggtgggaggtctatataagcagagctggtttagtgaaccgtcagatcctgcagaagttggtcgtgaggcact
gggcaggtaagtatcaaggttacaagacaggtttaaggagaccaatagaaactgggcttgtcgagacagagaagactcttgcgtttctgatag
gcacctattggtcttactgacatccactttgcctttctctccacaggtgtccaggcggccgccatggtgagcaagggcgaggagctgttcaccgg
ggtggtgcccatcctggtcgagctggacggcgacgtaaacggccacaagttcagcgtgtccggcgagggcgagggcgatgccacctacgg
caagctgaccctgaagttcatctgcaccaccggcaagctgcccgtgccctggcccaccctcgtgaccaccctgacctacggcgtgcagtgctt
cagccgctaccccgaccacatgaagcagcacgacttcttcaagtccgccatgcccgaaggctacgtccaggagcgcaccatcttcttcaagg
acgacggcaactacaagacccgcgccgaggtgaagttcgagggcgacaccctggtgaaccgcatcgagctgaagggcatcgacttcaag
gaggacggcaacatcctggggcacaagctggagtacaactacaacagccacaacgtctatatcatggccgacaagcagaagaacggcat
caaggtgaacttcaagatccgccacaacatcgaggacggcagcgtgcagctcgccgaccactaccagcagaacacccccatcggcgac
ggccccgtgctgctgcccgacaaccactacctgagcacccagtccgccctgagcaaagaccccaacgagaagcgcgatcacatggtcctg
ctggagttcgtgaccgccgccgggatcactctcggcatggacgagctgtacaagtaataagcttggatccaatcaacctctggattacaaaattt
gtgaaagattgactggtattcttaactatgttgctccttttacgctatgtggatacgctgctttaatgcctttgtatcatgctattgcttcccgtatggctttc
attttctcctccttgtataaatcctggttgctgtctctttatgaggagttgtggcccgttgtcaggcaacgtggcgtggtgtgcactgtgtttgctgacgc
aacccccactggttggggcattgccaccacctgtcagctcctttccgggactttcgctttccccctccctattgccacggcggaactcatcgccgc
ctgccttgcccgctgctggacaggggctcggctgttgggcactgacaattccgtggtgttgtcggggaaatcatcgtcctttccttggctgctcgcct
gtgttgccacctggattctgcgcgggacgtccttctgctacgtcccttcggccctcaatccagcggaccttccttcccgcggcctgctgccggctct
gcggcctcttccgcgtcttcgagatctgcctcgactgtgccttctagttgccagccatctgttgtttgcccctcccccgtgccttccttgaccctggaa
ggtgccactcccactgtcctttcctaataaaatgaggaaattgcatcgcattgtctgagtaggtgtcattctattctggggggtggggtggggcagg
acagcaagggggaggattgggaagacaatagcaggcatgctggggactcgagttaagggcgaattcccgataaggatcttcctagagcat
ggctacgtagataagtagcatggcgggttaatcattaactacaaggaacccctagtgatggagttggccactccctctctgcgcgctcgctcgct
cactgaggccccttaATTAAcattaagcgcggcgggtgtggtggttacgcgcagcgtgaccgctacacttgccagcgccctagcgcccgct
cctttcgctttcttcccttcctttctcgccacgttcgccggctttccccgtcaagctctaaatcgggggctccctttagggttccgatttagtgctttacgg
cacctcgaccccaaaaaacttgattagggtgatggttcacgtagtgggccatcgccctgatagacggtttttcgccctttgacgttggagtccacg
ttctttaatagtggactcttgttccaaactggaacaacactcaaccctatctcggtctattcttttgatttataagggattttgccgatttcggcctattggt
taaaaaatgagctgatttaacaaaaatttaacgcgaattttaacaaaatattaacgcttacaatttaggtggcacttttcggggaaatgtgcgcgg
aacccctatttgtttatttttctaaatacattcaaatatgtatccgctcatgagacaataaccctgataaatgcttcaataatattgaaaaaggaaga
gtatgagtattcaacatttccgtgtcgcccttattcccttttttgcggcattttgccttcctgtttttgctcacccagaaacgctggtgaaagtaaaagatg
ctgaagatcagttgggtgcacgagtgggttacatcgaactggatctcaacagcggtaagatccttgagagttttcgccccgaagaacgttttcca
atgatgagcacttttaaagttctgctatgtggcgcggtattatcccgtattgacgccgggcaagagcaactcggtcgccgcatacactattctcag
aatgacttggttgagtactcaccagtcacagaaaagcatcttacggatggcatgacagtaagagaattatgcagtgctgccataaccatgagt
gataacactgcggccaacttacttctgacaacgatcggaggaccgaaggagctaaccgcttttttgcacaacatgggggatcatgtaactcgc
cttgatcgttgggaaccggagctgaatgaagccataccaaacgacgagcgtgacaccacgatgcctgtagcaatggcaacaacgttgcgca
aactattaactggcgaactacttactctagcttcccggcaacaattaatagactggatggaggcggataaagttgcaggaccacttctgcgctc
ggcccttccggctggctggtttattgctgataaatctggagccggtgagcgtgggtcacgcggtatcattgcagcactggggccagatggtaag
ccctcccgtatcgtagttatctacacgacggggagtcaggcaactatggatgaacgaaatagacagatcgctgagataggtgcctcactgatt
aagcattggtaactgtcagaccaagtttactcatatatactttagattgatttaaaacttcatttttaatttaaaaggatctaggtgaagatcctttttgat
aatctcatgaccaaaatcccttaacgtgagttttcgttccactgagcgtcagaccccgtagaaaagatcaaaggatcttcttgagatcctttttttctg
cgcgtaatctgctgcttgcaaacaaaaaaaccaccgctaccagcggtggtttgtttgccggatcaagagctaccaactctttttccgaaggtaac
tggcttcagcagagcgcagataccaaatactgtccttctagtgtagccgtagttaggccaccacttcaagaactctgtagcaccgcctacatacc
tcgctctgctaatcctgttaccagtggctgctgccagtggcgataagtcgtgtcttaccgggttggactcaagacgatagttaccggataaggcgc
agcggtcgggctgaacggggggttcgtgcacacagcccagcttggagcgaacgacctacaccgaactgagatacctacagcgtgagctat
gagaaagcgccacgcttcccgaagggagaaaggcggacaggtatccggtaagcggcagggtcggaacaggagagcgcacgagggag
cttccagggggaaacgcctggtatctttatagtcctgtcgggtttcgccacctctgacttgagcgtcgatttttgtgatgctcgtcaggggggcgga
gcctatggaaaaacgccagcaacgcggcctttttacggttcctggccttttgctggccttttgctcacCCTTA

Corresponding DNA origami designs and staple lists:

2.2.6.1 sc_EGFP6 20HB

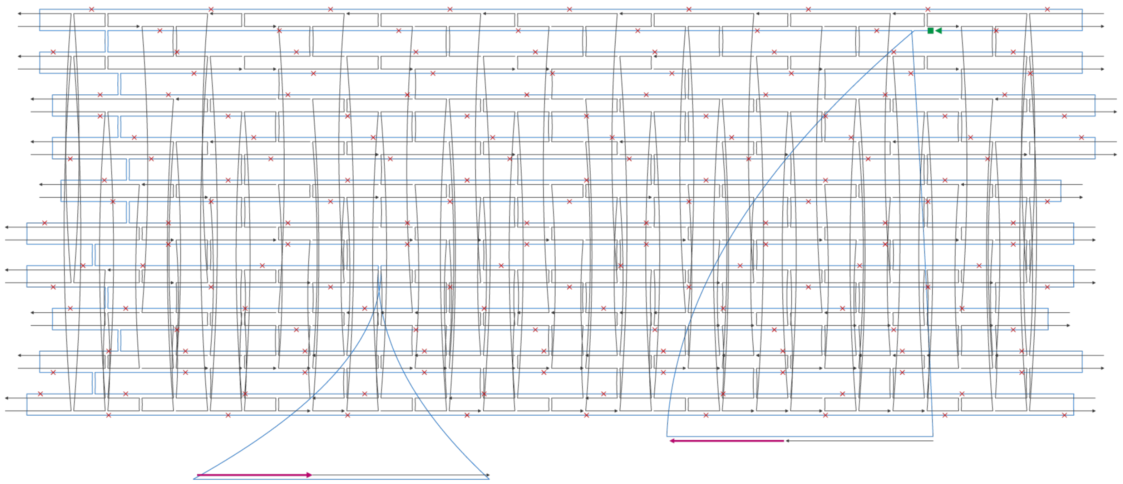

Fig. S39 | Scaffold routing and staple design for sc_EGFP6 20HB. Scaffold routing is shown in blue, staples are given in black, breakpoint for addition of sequence is marked by a green square (5') and arrow (3'). Staple sequences which position the ITR hairpins to flank the 5' promoter region and the 3' polyA region are given in pink (ITR hairpin is position on the left side of each of the loops). Design was prepared using caDNAno v0.1.

Table S26 | Individual staple sequences for sc_EGFP6 20HB.

Name	Sequence 5' - 3'
core_1	AATGACCCGACGTCAATGGAAAGCCCGTTGA
core_2	TAGCTAGCTGGGCAGTTTACCGTATTGACGT
core_3	CGGGTTAATACCGTCATTGACGTCTGATGTA
core_4	CATTATTGGGTTTTTATTTTTGTCTCATCATTAGCCATGCCTGCTATTGTCTTCC
core_5	GGCGTTACACTCCATATATGGGCTCGACCCTG
core_6	CACCCATTCGTAATTGATTACTATCCTGGAAG
core_7	AAATACTCTGATCTTTATTTCCCCAGTTCGAACCACACAATGACACCTACTCA
core_8	CTGCCAAGTTAAGGGCGAATTCCGACAGGAC
core_9	GGCGTACTGGCTACGTAGATAAGATCGACGC
core_10	GGGCCATTTCATTAACTACACTAAGGCTCCG
core_11	CCCCGTGCCAGTTTCTATTGGTCCTCGCCGCTCCCCGTTACAGGCAATGCCATCTGCGTCAG
core_12	CACTAAACCAGCTCTGGGTGGTCAGATTTATTTTATCCG
core_13	GACGGTTGTTGTTACGACATTTTGGAAAGTTCCCTATTGCAGAAAGCGTTAA
core_14	CACGACCAACTTCTGAGCTTGCGATACCGTGCCGGGAGTGTATGCGGGAAAGC
core_15	TGTAACCTTGATACTGTCAGCTTAGGGCTTTTAATAGT
core_16	ACCTGTCTCAATGGGGTGGAGACTTGGAAATTTGATGTAGAACGAAAAGAAAAAT
core_17	AAACGCAAGAGTCTTTACGTCGCCTGTCTATGGTATGGATGGCAG
core_18	GATGTCAGTAAGACCAACCACCCCGCTTAATCGATCAAGTAAGTTG
core_19	TGAAGAAGGGGCCAGGGCACGGGCCAGGATCTGATTACGC
core_20	TCCTGGACCAGATGAACTTCAGGTACCTGCCGATCCTT
core_21	GTTGCCGTTGGCATCGCCCTCGCCTCCTTAACTCAGTG
core_22	CCTCGAACGAACTTGTGGCCGTTCTCTGTCTTTTTGGTC
core_23	TCAGCTCTCGACCAGGATGGGCATAGGTGCACCTAGA
core_24	GGTCTTTGGGTCACGAACTCCAGGGCAGGCG
core_25	CGGCGAGGTCAATCTTTCACAAGTGGGGGTCGTAAGAT
core_26	GTTCTTCTACAAAGGCATTAAAGCCTCCTCATGCCGCAGT
core_27	CCGGCGGCCTCAGGGCGGACTGGGTGCTCAGGGCGGACTTTAATTGTCGTGACCC
core_28	GCTCGTCCATGCCGAAGGGAGGGGCGACCG
core_29	CTTGTACATGTCGGGCAGCAGCACGGGGCCGTGGTGCGCTTCGCCAGACCATCTG
core_30	GTTGATTGGATCCAAAGCTGACTCAACCAACAGAATAG
core_31	ATCCAGAGTGGGGGTGTTCTGCTGGTAGTGGTTCTTGTAGCCATTGCCGTGTAG
core_32	GAATACCACTGCACGCTGCCGTCCTCGATGTGTGTCGCCGTCGTTTTTCGTTCCACCAGCG
core_33	CGTAAAAGGAGCAACACCACGCCACACTGCAT
core_34	CCACATAGATCTTGAAGTTCACCTTGATGCCGATGCCCTTTCCCAACAGTGAGGCAGGCAAAA
core_35	CGTTGCCTTCCGCTGGATTGAGGGCCGAAGGGGAAAGGAAATTCTCTTTTACTTT

core_36	AAAGAGACCAGAGCCGGCAGCAGGCCGCGGGGTCAAGGAGTTATCACAAACAGGA
core_37	AATTGTCAGTTCCGCCGTGGCAATGAGTGATCCAGTCTA
core_38	TGATTTCCCCGACAACACCACGGCAATCCTAGTTGCTGTTCTTCACGCTCACAAGCAGCA
core_39	AGGACGAGAAAGTCCCGGAAAGGGCTTATTATAAGTAG
core_40	CAGGTGGGCAATGCCCCAACCAATTTTGTAACGTTGTT
core_41	AGAAGGACGTCCCGCGCAGAATCGACAATGCGCTTTTCTCACTCGTGATAACTACGTCTGACG
core_42	GACGTAGCCAAACACAGTGCACATAGTTAAGTCACGCT
core_43	AAGGAAGGGACAACGGGCCACAAAGCGTATAGCTCCGG
core_44	AGAGGCCGAGCAACCAGGATTTAAAGCAATA
core_45	CCCCCTTGACCCGCCATGCTACTTATAGACCG
core_46	GATGCAATGTAGCGGTCACGCTGCTGGACTCTAGGGGTT
core_47	CAGTGGGAAAAGGAGCGGGCGCTCTATCAG
core_48	AGGCACGGAACGTGGCGAGAAAATCACCCTTATCAGG
core_49	TGGCAACCCCGATTTAGAGCTTTGCCGTAA
core_50	CCGCCGCGCTTAATGTGTTTGGAAGCCACCTA
core_51	TTATAAATCAAAAGAATCTACGTGGAAAACCTTGCCCGCCTCCATC
core_52	GTTGAGTGTTGTTCCATAATTAAGAGGATCTTTGAGAATAAGCTAGAG
core_53	CCACTATTAAAGAACGGCGTAACCTGTAACCGTGACTGTTGCGCA
core_54	AAGGGCGAAAAACCGTAGGGCGCTAGCATCTTACTGTCTCGTGGT
core_55	CCCACTACGTGAACCGGAAGGGAGTGAGCAATCATGGTTCTTCATTC
core_56	TTTTTGGGGTCGAGGGACGGGGAAAAAAGGGGTCAGAAG
core_57	TTAACCAAACAAACAGCCGGACGCGCACTGCACGCCGTAGGTCAGCTTATATA
core_58	CTCATTTTGCCAGGCTGGGCGGGGGTCGTTGGCATACACGCCTACCGCCCATTTGCGTCAAT
core_59	GCGTTAATTGTTTGCCGGCTCCACGAGGGTTCGTGCTGCTTCATG
core_60	TAAAATTCACGCGGATATGGGAACATACGTGGGGCGGA
core_61	ATATTTGATAGAGCATTGGCATATGATACAC
core_62	CGGATACGGCGATGGTCAAGTCAGAGGTGGCGAAACCCGATCTTCCATGTATTT
core_63	CTCATGAGTTTCTGGAGAAAGCGGTGGCACCTTCCAGG
core_64	GAAGCATTAATCAAGTCCCCCCTGACGAGCATCACAAAATAGCATGGGTTATTGTATCAAAAA
core_65	ATATTATTTGCCGCAAAGCCGGCGGGGAGGGGCAAACAA
core_66	CTTTTTCAAATTAAATTACCAATGGTGAACCGTCCTCCTTGAAGTC
core_67	ACTCTTCTGAGCAAAATGTACTGGGCATAATTAATAGCG
core_68	GGGGCGAATATTTTGTAGATAGGCCGCTTACCGGATACCTGTCCGCGTTATGTA
core_69	AACTCTCAAATGATTACTGTCCTGCCCCACC
core_70	ACCGCTGGCCCCAGTCTCAAGAACAGTGCCTTTTTGGTGCCAAAACAAACTCCC
core_71	TTGAGATCCGAAAAGTCAAGAGTCTCCCTCGTGCGCTCTCCTGTTCATGAACTAATTGTAA
core_72	CACCCAACAAACAAACAACGTCATATAAAGATACCAGGCGTTTCCCTAATAACCCGCGCAC
core_73	TGATCTTCGGCAAGTTTCCTCATTTTATTA
core_74	ACGGGATCCAGCATGCTCTAGGAAGATCCTAAATCGGC
core_75	GCGTCAATGCGATGAGTGCCCAACAGCCGAGCCCCTGTCGAGTCC
core_76	GTGAGTACAGGTGGTGCAACACAGGCGAGCAGCCAAGGAACCACCCCCGTCATTC
core_77	GCGAGTTAGCATGATGCTTGTCGGCCATGATATAGACG
core_78	CATGATCCATGTTGCAGCTCCTCGCCCTTGCGAGAAAG
core_79	CAGCAATAAAAAGTGTAAATCAGAAAATCCCCCTTCGGGAAGCGTGGCGCTTTCTGGCGGTCA
core_80	ATCCATAGTAAGGGACGACAAGCAGTCAAACCGCTATCCACGCCCATAATAGGGATGAGATT
core_81	TCAGCGATCGTCCAGCGATGCGGTTCACCAGGTGTGGCGG
core_82	ACCTATCGGATCTTCCTATCAGCTGCCAAAACCGCATCACCATGGGCCAGGCTCCTTTTA
core_83	GTAGCGGTACGTCAAGGGCCATTTACCGTAACTTTCTC
core_84	AAAAGGATGCTGCAATCGGTGGTGGTAGCCTTCGGGCATGTAGTGGT
core_85	TCTACGGGGATACGGGGCCGTAGGCGTCCTTGAAGAAGATCGCCGA
core_86	ACTCACGTTTGCCTGAGACACGCTTTCACCTCGGCGCGGG
polyT_1	TTTTTACCGTAAAAAGGATCGGAACCCTTTTT
polyT_2	TTTTTAGGTATCTCAGTTCGGCATAGCTTGGCCTAAATAGGCCGTATCGGGA
polyT_3	TTTTTTAGGAAAGTCCCAAATCTAAAGTTTTTT
polyT_4	TTTTTCCAAGCTGGGCTGTGTTGTAGGTCATCCGGCACTACGGCTACACTTTTT
polyT_5	TTTTTCCTGGACACCTGTGGATCACCATGGCGGCCGTTTTT
polyT_6	TTTTTGCCCGACCGCTGAGGTTCGGAAAAAGAGTTTTTTT
polyT_7	GCCCCAGGCCCATGTAACTTGGTCGACACGGAAATTTTTT
polyT_8	TTTTTTGTACTCCAGCTTGTTTGTGGCTGTTGTAGTTTTTT
polyT_9	TTTTTCGTCTTGAGTCCAACCCGATCGCGCTTCTCGTTGGTGGTCGGGCTGCAAC
polyT_10	TTTTTATGAAAGCCATACGGGTACAAGGAGGTCCTC
polyT_11	TTTTTATCGCCACTGGCAGCGCAGCGGGCAACAGGACCA
polyT_12	TTTTTGTCGAGGCAGATCTCGAAGACGCGGACAGATGGCCGATCGTTAATAAGGG
polyT_13	TTTTTGAGCGAGGTATGTAGGCGCCCTTAACTCCAAGCCACTGGTAACAGGATTAGCATTTTT
polyT_14	TTTTTTAAAGGGAGCCTAGAAGGCACATTTTT
polyT_15	TTTTTCTTGAAGTGGCACGCTGTTTTTT
polyT_16	TTTTTGTTGAATACTCATAGCACTAACCGCGTTGCTGGCGTTTTTCCATATTAAGGG
polyT_17	TTTTTTAGAAGGACAGTAATTCGGTGCTACAGAGTTTTTTT
polyT_18	TTTATAGCAGAACTTTAACCAGCCCACCGCTGGACCTCCCACCGCGAACCCCCCGTTCATTTTT

polyT_19	AATACCGCGCCACGGGAGCGCAGTGTGGTAAGACACGACTTTTTTT
polyT_20	TTTTTGGTTAGCTCCTTCGGAGAAATTTTT
polyT_21	TTTTTAAGCCAGTTACCGCCTATCTGCGCTCTGCTGTTTTT
polyT_22	AAGTGGTCGTAGCGGCTGAACTTATCCGGTAACTATTTTTT
polyT_23	CTGACAGAATGAAGTGCAAAGTGATGACTAATACGTAGATGTACTGCCAAGTTTTT
polyT_24	TTTTTATATATGAGTATGTGCAAAAAAGCTTTTT
polyT_25	TTTTTGGTAGCTCTTGGTTCGCTTTTTT
polyT_26	TTTAAATCTAAGGTCAGGCCAGCAAAAGGCCAGGATTTTT
Loop_1	AGGAACCCCTAGTGATGGAGTTGGCCACTCCCTCT
Loop_2	AGAGGGAGTGGCCAACTCCATCACTAGGGGTTCCTTGTAGTT
ITR_1	CTGCGCGCTCGCTCGCTCACTGAGGCCGCCCGGGCAAAGCCCGGGCGTCGGGCGACCTTTGGTCGCC C
ITR_2	GGGCGACCAAAGGTCGCCCGACGCCCGGGCTTTGCCCGGGCGGCCTCAGTGAGCGAGCGAGCGCGC AG

2.2.6.2 sc_EGFP6 20HB-exLP

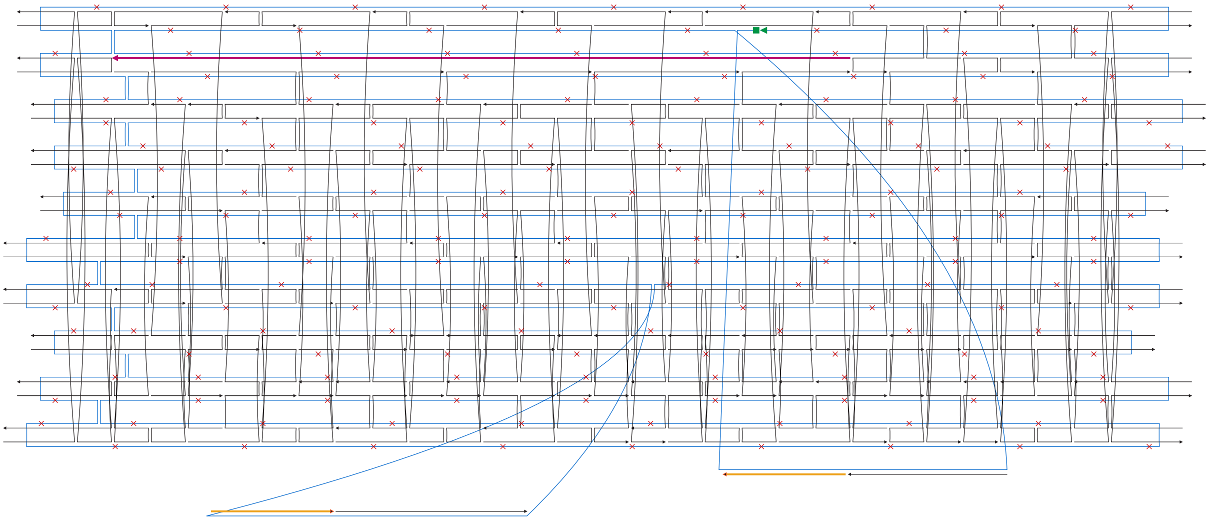

Fig. S40 l Scaffold routing and staple design for sc_EGFP6 20HB-exLP. Scaffold routing is shown in blue, staples are given in black, breakpoint for addition of sequence is marked by a green square (5') and arrow (3'). Staple sequences which position the ITR hairpins to flank the 5' promoter region and the 3' polyA region are given in orange (ITR hairpin is position on the left side of each of the loops). Long 154-mer 'primer' sequence at the promoter (5') region is given in pink. Design was prepared using caDNAno v0.1.

Table S27 l Individual staple sequences for sc_EGFP6 20HB-exLP.

Name	Sequence 5' - 3'
core_1	CCGATCTTCCTAGAGCATGGCTACACCTGTCC
core_2	AGTAGCATGGCGGGTTAATCATTTCTCCTGT
core_3	AACTACACTAATTAAGGGTGAGCAAAAGGCCGGCGTTTC
core_4	GCCAGGAATACTGCCAAGTGGGCAGATGTAC
core_5	ACTTGATGCCGTAAAAAGGCCGCGGCGAAAC
core_6	GTCCCGTTGATTTTGGTGCCAAAACAAACTCATTTGCGTCAATGGGGAACTTCA
core_7	TAGACCTCCCACCGTCGCCCTCCCAGCCGTTGCCATT
core_8	CTGACGGTTCACTAATGTGGCCGACCGGCTCTCGTCGT
core_9	TGCAGGATATTGATGTACTGCCAAAACCGCATCACCAT
core_10	CCCAGTGCCTCACGACAGGATGGGTGCTGCAGGTTCCCATCTGAGAAAACTCCTC
core_11	AAACCTGTCTTGTAATCGCCCTTACGATACGCCCCCATCTGTGAC
core_12	CTCGACAAGCCCAGTTCACCTGTGAGTTGCCTCTTCGGTCTTACTG
core_13	GCCGTAGGTTGCCGTAGGTGGCATACACGCCT
core_14	TGTGGTCGCGGACACGCTGAACTACCAGCTC
core_15	ATGGCGGAGCCGTCCAGCTCGACCCAACTTCAAGGATC
core_16	GATGGTGCCCGGTGAACAGCTCCCCTTGATA
core_17	GGTCTTGTGGCGGCCGCCTGGATCTATTGTATGAGTA
core_18	CACGGGGCCTCAGGGCGGACTGGCCCGGAAA

core_19	CTTGATGTACTTGTACAGCTCGCGGGCCACTAGTGTAT
core_20	TCCAGCTTAAGAATACCAGTCAATGCCATACGTCATGCCA
core_21	GGTCTTTGCGTCGCCGATGGGGGTGTTCTGCCACTGCACCAACGTTGGAAGGGCC
core_22	CATGTGATCGCGCTTCCCCAACTAGCAGAA
core_23	AGCAGGACGTCGGCGAGCTGCACGCTGCCGTCTGCTTCAGTGTCACGCCAGATTT
core_24	TCCCGGCGGCGGTCACAGTGCACGGCGTCATAGGAAAG
core_25	GAGAGTGAGTTGTGGCGGATCTTGAAGTTCACTTCGGGCTCAGCTCCATGATAC
core_26	AAGCTTATCCGTTCTTCTGCTTGTCGGCCATTGAAGAATACATGATGGAGGGCGGAATAAG
core_27	TAATCCAGAGGTTGATCCAGGATTTGGTGAGT
core_28	AAATTTTGCGTTGTGGCTGTTGTAGTTGTACTCGGCGCGGTTAGCTCGACTCCCCTCATACTC
core_29	ACAGGTGGTGGCAATGCTCGTTGGGTTTGCG
core_30	GTTGCGTCAGCAAACACGAACTCCCATCGTG
core_31	CACGTTGCCTGACAATCCATGCCGGCTTCAT
core_32	TATACAAGTCCAGGTGGCAACACAGGCGAGCGGGCAAACACTCAACCCAAAAAAG
core_33	GAGGAGAAAATGAAACTTTCACAAAAAGCG
core_34	GGAAGCAAGGGACGTAGCAGAAGGACGTCCCGCACAGTCTCCGTAAGTTGAATAC
core_35	AAAGGACGATGATTTCCCCGACAACCTTCCAGCGGCGACGGGTGAGCCGCGTGACTTATCAAA
core_36	AGCCAAGGATAAAGAGACAGCAATGGATCCAGGCGAGT
core_37	GGGCCGAATAGCATGATACAAAGAGGAGCAA
core_38	CAGACAATGAATTCGCCCTTAACTTTAAAGAA
core_39	GGGTCAAGAAGAATGATTAACCCGACGTGAAAATATTTT
core_40	AACAGATGCCACCACACCCGCCGGGGTCGACCCGAAAA
core_41	GAGGCAGCTGGCAAGTGTAGCGAACCCTAAATAAACA
core_42	CAGAGCCGAAGAAAGCGAAAGGGACGGGGA
core_43	CCAGCATGCCCAGAATAGAATGACGGCGATGA
core_44	CTTATCGGGCGATGCAATTTCCTAGCCCCT
core_45	ACGTAGCCATGCTCTAGAAAAACCGTTAAATC
core_46	CTTATCTGACAGTGGGAGTGGCACACCAC
core_47	GCGCGTAAGCTGGCAACTAGAAGGCGCAGAA
core_48	AACAAGAGTCCACTACGAGTCCTCCAGTTCAGTGCTCAAGTTAATA
core_49	TCCAACGTCAAAGGGCGGAAGATCACTGATCTATAATACCGCTACAGG
core_50	AGGGCGATGGCCCACTCCATGCTACGTTTCTCGAGTTGTTGGTAT
core_51	CTAATCAAGTTTTTTGCGCTTAATAATGCCGAAGTCATACGATCA
core_52	AAAGCACTAAATCGGGTCACGCTCGGAAATGATGCTTTTGTTGTGCA
core_53	CCCGATTTAGAGCTTAGCGGGCGTTCAATATATAATTCT
core_54	CGAGATAGCGCAGACAGTCTATCGAGCTTGCCGGTGGTGCAGATGCGGAGTTG
core_55	GAATAGACCTATGAACTAATGACCCCGTAATCTCAGTT
core_56	GCAAAATTTTGATCTAAGTGGTGGGTCAGCTCAGGGTGGTCACGA
core_57	CGAAATCGATTAATAACTAGCTAGCTTAAGGGGCGCTTT
core_58	GTGCCACCGATCCTTTTGGCCCCAGCACCACCGCTCCTGGACGTAGCC
core_59	ACATTTCGGTGCCGTCCCCTGGAAGCTCCCTCGTGCGC
core_60	TTAGAAAAAGGGAGCCCCGACAGGACTATAAAGATACCAAGCAAAAGAATAGGGGTCAATCTA
core_61	GAATGTATTTCCTTTCTAGGGCGATCTCGAAGACGCGGA
core_62	ACATATTTCTGACAGTCATCCATGAGAGAAGCCCTCGAACTTCACC
core_63	AGCGGATCGTTTTTCGGGGCGTACTTGGCATGGTCATGT
core_64	GCTACTCTCAAGGATCCCTCCATCAAAAAAGGTTACGAC
core_65	GATGTAAGAGCGCAGTTTCTACGACCGCCCCCATTGACGTCAATGGGGTGGAGACTTGGAA
core_66	CCCACTCGCAATAGGCCGTGGACCTCATAGCTCACGCTGTAGGTATTGATTACT
core_67	TCAGCATATCAGCAAAAACTCACTGCTTATAATCCCCGTGAGTCAAACCGCTATCCACGCCC
core_68	CTTTTACTAAATTTTTGTCTATCGCCTTTCTCCCTTCGGGAAGCGTGCGAATTAGCTCATT
core_69	AAAAACAGAAGCGTTCCATCACCTCCGACCCTGCCGCTTACCGGATGTAGATAGTTAAAAT
core_70	GAAGGCAAGTTAATTGAAGGCACGGGGGAG
core_71	AAACGTTCCCCACCCCCTGCTATTGTCTTCTGTTGTTC
core_72	GCGCCACACAGTGGGGGTCCAGCAGCGGGCAAGGCAGGCACCTACTCTTTAAA
core_73	CTCTTGCCCACCACGCGGAATTGTCAGTGCCCAACAGCCGCATTTTATATACGGG
core_74	CCTCCGATCATAGTTGTGCCCCAGGATGTTGCCGTCCT
core_75	CGTTGTCAGGGTGTCAGGCAAAGTGGATGTCAAGAGTC
core_76	TTTATCCGTTACCGCAATCAAAACAGTTTGGCGGTGTAGGTCGTTCGCTCCAAGCTATATGGG
core_77	CCTGCAACAGTTCGCCTCATTGGAGGAGCTGGTTCCGCCGTGGCAATAGGGAGGTGCCCCA
core_78	TTACCATCTAAATTACTTACCTGGGTAATAGCGATGACTAATACGTA
core_79	AGATAACTGCTCACCATAGTTGCCGTCGTCCTGATATAGA
core_80	GTCGTGTAAGTATAGTCTCCTTTGCCAAGTAGGAAAGTCCCATAAATGATACAACTTGGT
core_81	ATCTCAAGAAGATCCCCCTTATATGTTGAGA
core_82	GGGTCTGACGCTCAGTTTTAACTGCACCCA
core_83	TGGAACGATAAACCAGGCCCTCGCGGGTAGCGGCTGAAGTGGTAGTG
core_84	GTTAAGGGATTTTGGTCGCGTTTTCACCAG
core_85	TCATGAGACCACGCTCTTTACGTCCTTGAAGAAGTCGTGCCTCGAT
core_86	TTCACCTATAAATTGT
core_87	AAAATGAAGTTTTAAATTCCGCGCGGCGACA

core_88	GTTTACCGTAAATACTCCACCCATTGACGTCAATGGAAAGTCCCTATTGGCGTTACTATGGGAACATACG TCATTATTGACGTCAATGGGCGGGGGTCGTTGGGCGGTCAGCCAGGCGGGCCATTTACCGTAAGTTATG TAACGCGGAACTCCA
polyT_1	TTTTTCGAGCATCACAAGAACGTGGCGTTTTT
polyT_2	TTTTTAACCCCCCGTTCAGCCTGGGCTGTGAAGCCAGGGTTGAGCCAATCCT
polyT_3	TTTTTACCGTCATTGACGAGTGAGGCACTTTTT
polyT_4	TTTTTATCCGGTAACTATCGTCGACCGCTCAGATTACGTTACCTTCGGATTTTT
polyT_5	CTTGGAAAATTTTGAGTCCAACCCGGTATTTTT
polyT_6	TTTTTTGCCTATCAGAAACGCAGTAAGACCAATAGGTTTTT
polyT_7	TTTTTAGACACGACTTATTAGCGGTGGTTTTTTTGTTTTT
polyT_8	GTTCACCAGAAGTAAATCTGTCTCATTTATCAGGGTTTTT
polyT_9	TTTTTCTTCAGCTCGATGCGCCTTGAAGTCGATGCCTTTTT
polyT_10	TTTTTCTGGTAACAGGATTAGCGTGGTTGTCGGGCAGCAGGGGTGGGCAGTAAGT
polyT_11	TTTTTATCCACATAGCGTAAAGCATTAAATTATGGC
polyT_12	TTTTTGGCGGTGCTACAGAGAAAGCGAAAGTGTGCTCAG
polyT_13	TTTTTGCGGGAAGGAAGGTCCGCTGGATTGAAGAGGCCGAGCACTGCTATTGAAG
polyT_14	TTTTTCGGCTACACTAGAAGGCCTTGCTGTCCGGGTTCTTGAAGTGGTGGCCTAACTATTTTT
polyT_15	TTTTTAGAAAGGAAGGGGCAGCAGGCCTTTTT
polyT_16	TTTTTTGCGCTCTGCTGTGCACGTTTTT
polyT_17	TTTTTTTATTGTCTCATGAAGCCGGCAAATCGACGCTCAAGTCAGAGGTGTTGCTGG
polyT_18	TTTTTAAAAGAGTTGGTACCCACAGTATTTGGTATCTTTTT
polyT_19	CTTCGGGGCGAAACTGTTGCCGGGTAAGAGCGAGGTATGTATTTTT
polyT_20	TTTTTTTATCACTCATGGGCAGCGTTTTTT
polyT_21	TTTTTCCACCGCTGGTAATTTGATCCGGCAAACAAATTTTT
polyT_22	GAAGCTAGCAGGGCACGGGCCCACTGGCAGCAGCCATTTTT
polyT_23	ATTTCGTTTACCAATTTCTCTGTACTGGGCATAATGCCAGGCGGGCCATTTTTTTT
polyT_24	TTTTTCTATCTCAGCGGTTGGCCGCAGTGTTTTT
polyT_25	TTTTTTTTGCAAGCAGGCGCCTTTTTTT
polyT_26	GCTTAATCTCAATAGCATAGGCTCCGCCCCCCTGATTTTT
Loop_1	AGGAACCCCTAGTGATGGAGTTGGCCACTCCCTCT
Loop_2	AGAGGGAGTGGCCAACTCCATCACTAGGGGTTCCTTGTAGTT

2.2.7 sc_mCherry5

Scaffold sequence for sc_mCherry5:

taggctgcgcgctcgctcgctcactgaggccgcccgggcaaagcccgggcgtcgggcgacctttggtcgcccggcctcagtgagcgagcg
agcgcgcagagagggagtggccaactccatcactaggggttccttgtagttaatgattaacccgccatgctacttatctacgtagccatgctcta
ggaagatcggaattcgcccttaagctagctagttattaatagtaatcaattacggggtcattagttcatagcccatatatggagttccgcgttacat
aacttacggtaaatggcccgcctggctgaccgcccaacgacccccgcccattgacgtcaataatgacgtatgttcccatagtaacgccaatag
ggactttccattgacgtcaatgggtggagtatttacggtaaactgcccacttggcagtacatcaagtgtatcatatgccaagtacgccccctattg
acgtcaatgacggtaaatggcccgcctggcattatgcccagtacatgaccttatgggactttcctacttggcagtacatctacgtattagtcatcgc
tattaccatggtgatgcggttttggcagtacatcaatgggcgtggatagcggtttgactcacggggatttccaagtctccaccccattgacgtcaat
gggagtttgttttggcaccaaaatcaacgggactttccaaaatgtcgtaacaactccgccccattgacgcaaatgggcggtaggcgtgtacggt
gggaggtctatataagcagagctggtttagtgaaccgtcagatcctgcagaagttggtcgtgaggcactgggcaggtaagtatcaaggttaca
agacaggtttaaggagaccaatagaaactgggcttgtcgagacagagaagactcttgcgtttctgataggcacctattggtcttactgacatcca
ctttgcctttctctccacaggtgtccaggcattattcgccaccatggtgagcaagggcgaggaggataacatggccatcatcaaggagttcatgc
gcttcaaggtgcacatggagggctccgtgaacggccacgagttcgagatcgagggcgagggcgagggccgcccctacgagggcaccca
gaccgccaagctgaaggtgaccaagggtggccccctgcccttcgcctgggacatcctgtcccctcagttcatgtacggctccaaggcctacgt
gaagcaccccgccgacatccccgactacttgaagctgtccttccccgagggcttcaagtgggagcgcgtgatgaacttcgaggacggcggc
gtggtgaccgtgacccaggactcctccctgcaggacggcgagttcatctacaaggtgaagctgcgcggcaccaacttcccctccgacggccc
cgtaatgcagaagaagaccatgggctgggaggcctcctccgagcggatgtaccccgaggacggcgccctgaagggcgagatcaagcag
aggctgaagctgaaggacggcggccactacgacgctgaggtcaagaccacctacaaggccaagaagcccgtgcagctgcccggcgcct
acaacgtcaacatcaagttggacatcacctcccacaacgaggactacaccatcgtggaacagtacgaacgcgccgagggccgccactcc
accggcggcatggacgagctgtacaagtgagcgggtgattggatccaatcaacctctggattacaaaatttgtgaaagattgactggtattctta
actatgttgctccttttacgctatgtggatacgctgctttaatgcctttgtatcatgctattgcttcccgtatggctttcattttctcctccttgtataaatcctg
gttgctgtctctttatgaggagttgtggcccgttgtcaggcaacgtggcgtggtgtgcactgtgtttgctgacgcaacccccactggttggggcattg
ccaccacctgtcagctcctttccgggactttcgctttccccctccctattgccacggcggaactcatcgccgcctgccttgcccgctgctggacag
gggctcggctgttgggcactgacaattccgtggtgttgtcggggaaatcatcgtcctttccttggctgctcgcctgtgttgccacctggattctgcgc
gggacgtccttctgctacgtcccttcggccctcaatccagcggaccttccttcccgcggcctgctgccggctctgcggcctcttccgcgtcttcgag
atctgcctcgactgtgccttctagttgccagccatctgttgtttgcccctcccccgtgccttccttgaccctggaaggtgccactcccactgtcctttcct
aataaaatgaggaaattgcatcgcattgtctgagtaggtgtcattctattctggggggtggggtggggcaggacagcaagggggaggattggg
aagacaatagcaggcatgctggggactcgagttaagggcgaattcccgataaggatcttcctagagcatggctacgtagataagtagcatgg
cgcattgccttaATTAAcattaagcgcggcgggtgtggtggttacgcgcagcgtgaccgctacacttgccagcgccctagcgcccgctccttt
cgctttcttcccttcctttctcgccacgttcgccggctttccccgtcaagctctaaatcgggggctccctttagggttccgatttagtgctttacggcacc
tcgaccccaaaaaacttgattagggtgatggttcacgtagtgggccatcgccctgatagacggtttttcgccctttgacgttggagtccacgttcttt
aatagtggactcttgttccaaactggaacaacactcaaccctatctcggtctattcttttgatttataagggattttgccgatttcggcctattggttaaa
aaatgagctgatttaacaaaaatttaacgcgaattttaacaaaatattaacgcttacaatttaggtggcacttttcggggaaatgtgcgcggaacc
cctatttgtttatttttctaaatacattcaaatatgtatccgctcatgagacaataaccctgataaatgcttcaataatattgaaaaaggaagagtatg
agtattcaacatttccgtgtcgcccttattcccttttttgcggcattttgccttcctgtttttgctcacccagaaacgctggtgaaagtaaaagatgctga
agatcagttgggtgcacgagtgggttacatcgaactggatctcaacagcggtaagatccttgagagttttcgccccgaagaacgttttccaatga
tgagcacttttaaagttctgctatgtggcgcggtattatcccgtattgacgccgggcaagagcaactcggtcgccgcatacactattctcagaatg
acttggttgagtactcaccagtcacagaaaagcatcttacggatggcatgacagtaagagaattatgcagtgctgccataaccatgagtgata
acactgcggccaacttacttctgacaacgatcggaggaccgaaggagctaaccgcttttttgcacaacatgggggatcatgtaactcgccttga
tcgttgggaaccggagctgaatgaagccataccaaacgacgagcgtgacaccacgatgcctgtagcaatggcaacaacgttgcgcaaact
attaactggcgaactacttactctagcttcccggcaacaattaatagactggatggaggcggataaagttgcaggaccacttctgcgctcggcc
cttccggctggctggtttattgctgataaatctggagccggtgagcgtgggtcacgcggtatcattgcagcactggggccagatggtaagccctc
ccgtatcgtagttatctacacgacggggagtcaggcaactatggatgaacgaaatagacagatcgctgagataggtgcctcactgattaagca
ttggtaactgtcagaccaagtttactcatatatactttagattgatttaaaacttcatttttaatttaaaaggatctaggtgaagatcctttttgataatctc
atgaccaaaatcccttaacgtgagttttcgttccactgagcgtcagaccccgtagaaaagatcaaaggatcttcttgagatcctttttttctgcgcgt
aatctgctgcttgcaaacaaaaaaaccaccgctaccagcggtggtttgtttgccggatcaagagctaccaactctttttccgaaggtaactggctt
cagcagagcgcagataccaaatactgtccttctagtgtagccgtagttaggccaccacttcaagaactctgtagcaccgcctacatacctcgct
ctgctaatcctgttaccagtggctgctgccagtggcgataagtcgtgtcttaccgggttggactcaagacgatagttaccggataaggcgcagc
ggtcgggctgaacggggggttcgtgcacacagcccagcttggagcgaacgacctacaccgaactgagatacctacagcgtgagctatgag
aaagcgccacgcttcccgaagggagaaaggcggacaggtatccggtaagcggcagggtcggaacaggagagcgcacgagggagcttc
cagggggaaacgcctggtatctttatagtcctgtcgggtttcgccacctctgacttgagcgtcgatttttgtgatgctcgtcaggggggcggagcct
atggaaaaacgccagcaacgcggcctttttacggttcctggccttttgctggccttttgctcacCCTTAat

Corresponding DNA origami designs and staple lists:

2.2.7.1 sc_mCherry5 16HB_1

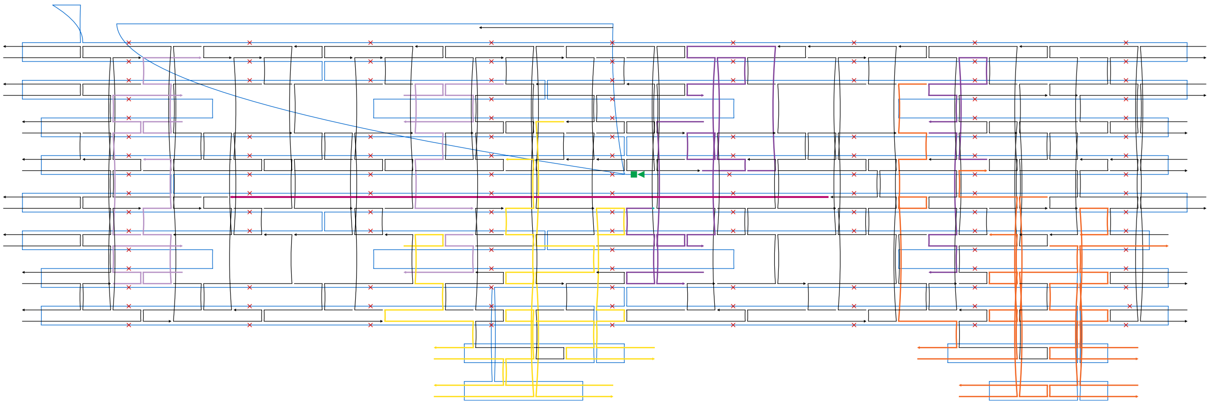

Fig. S41 I Scaffold routing and staple design for sc_EGFP5 16HB_3. Scaffold routing is shown in blue, staples are given in black, breakpoint for addition of sequence is marked by a green square (5') and arrow (3'). Long 154-mer 'primer' sequence at the promoter (5') region is given in pink. Protrusions 1 and 2 are shown in yellow and orange respectively, recess' 1 and 2 are given in light and dark purple, respectively. Design was prepared using caDNAno v0.1.

Table S28 I Individual staple sequences for sc_mCherry5 16HB_1

Name	Sequence 5' - 3'
core_1	TAATACCGAACTAATGACCCCGTAATTGATTGTCAAAGCCACTCG
core_2	ATGCTTTTATGGCGGGTTAATCATTAACTACATCCCTT
core_3	TTTTTCCCAACGATCAAGACCCGACATTTTT
core_4	TTTTTTCGGGGCGAAAACTCTCATTGGAAAACGTTCTTTTTT
core_5	AACTTTACGTTAAGGGATTTTGGTCATCGA
core_6	TCCGCCTCCATCCAGTATAATTCTGGGTGAGCAAAA
core_7	TAGTTCGCCCGCCTTTAACCCCCCGCACCTTGAAGCGCATCCAGCCCA
core_8	TTTGGTATAAACCACCGCTGGTAGTCTGCTGA
core_9	AAGGCAAAATGCCGCGAATACTCTGAGCGGATACATATTTGATTTTT
core_10	TCACCAGCGTTTCTGGGTGAGCACGCACATTACCGTCTAGGAAGAAA
core_11	TTTTACTTTGCACCCAACTGATCTCGAGTTGC
core_12	TACCGCGTGACCCACTCAGCATCTGTAAGCGGAACGTGGGCTGGCAACGTCAATG
core_13	TGCAATGAATTTATCAGCAATAAAAAGTCATT
core_14	TTTTCTACGGGAAGCTAGAGTAAGGTGTTATC
core_15	GATCTCAAGAAGATCCTTTGATCTGTGCACGCTCCCTTCGCTATCCACGCCCATT
core_16	AAAAAAAGTAGTTTGCGCAACGTTTCCGATCG
core_17	TTTTTGTTGGCATCGTGGTGTCACTGTGCAAA
core_18	CGGTGGTTCCAACCCGGAAGCTCCACGTAGAT
core_19	TTTTTTAGCTCTTGATCCGGCAGCAGTTTTT
core_20	TTTTTGCGACACGGAAATGTTAAAAAAGGGAATAAGGTTTTT
core_21	GCGAGGTATGTAGGCGGCAGGGGGCCTCGCCC
core_22	TTTTTCCACTGGTAACAGGACGCCACTGGCAAACGGCTTCATTCAGCTCCGGTTTTTTT
core_23	TTTTTATGTATTTAGAAACCATCACCCTTTTT
core_24	AAAATAAAAAGGCAATTAGGAAGACTTCCCAATCCTCCCCCTTTTTTT
core_25	ACCAATAGAAAGAATACCAAAACAAACTCCCA
core_26	GCCGAAATATGGTGGCGAATACACAAGAGGCC
core_27	CGGCAAAGTAAACTAGGATCTTCACCTAGATCCTTTTGTGGAACGCGGTGTAG
core_28	CGACTTATTTAGCAGAAGCCAGTTACCTTCGGAAAAAGAGTTGGTTTTT
core_29	GGCGAAAATCCCCGAAACCACCACACCCGCCG
core_30	CTATTAAATTAATATTCCCATTTGGTGTAGCG
core_31	TGTAGGTACTAATTAACTTACTGTCATGCCATCCGTAAGGGTCCTGCATAAATCA
core_32	GCTCACGCTGGAGACTTGGAAATCCCTCTGCTTACATCCG
core_33	TTTTTGGACTATAATAAGGTCATGTATTTTT
core_34	TTTTTTAATCAAGTTTTTTGGGGTCTAAAGGGGGCGAACGGAAAGCGACCACCCCA
core_35	CCGTAAAGCGCCACATAGCAGAACTTTAAAACAAGGATCACTACGTG
core_36	TAACTAGCTAGCTTAAGGGCGAAGAGTCCAGCTCCAG
core_37	TTCCTAGAGCATGGCTACGTAGATAGGGTTCCGGAAG

core_38	TAAGTAGCCTGTGACTGGTGAGTACTCAACCCCAGCCAGGAGTGTTGTTGTTAAA
core_39	ACCGTAAAAAGGCCGCGTTGCTGTACCTGTCAGTTAA
core_40	CCATAGGCTCCGCCCCGCATATGACCATGGTATTCTTGGCGGGGAAGT
core_41	AAAAATCGACGCTCAACCGTCATTGTACTGCC
core_42	TGGCGAAGCGAGTTACATGATCCCCCATGTGCTCGTCGTCCCCCTGGTAAGACAGGGGCGGC
core_43	CACTAAATCGGAACCCGAGGTGATGGCCCTTACCGC
core_44	GGGCTATGTGGGGGTTGCGTCAGCAAACACACAGGCGG
core_45	CATATATGCGAAAGGCGATGAGTACTCAGACAATGCGATGCAATTTGTCACGCT
core_46	TTTTTCTGGGCATAATGCACCCGCTCTTTTT
core_47	TTTTTCTTGACGGGGAAAGCCAGCCCCCGATTTAGAGTTTTT
core_48	AGCGGGCGCGCGTAAAGTGCCACCTAAAT
core_49	GCTAGGGCACTCCAACACTATTAATCTTGCCCGGCGTCAATACGGGAGATGTAAC
core_50	GTTGTTACTGGAACAATTCCGATCCTGAGAATAGTGTATGCGGCGACGCTCACCG
core_51	CCCGTGATGATGATCTCCAAGCTGGGCTG
core_52	ATAGCGAGATCTCGACTATCGGAGTTCTAGGACAGTATTTGGTATCTGCGC
core_53	TGACTAATCTCGTGCGAGCATCACAAAGCGGTTAGCTCCTTCGGTCCGTTGCCATGTTCCGAC
core_54	CTCTGCTTCAGCTCATCAGTGCCGTCGTGTAGATAACTAGAGATTATCAAAATGGTCTGA
core_55	GCCGTTCACCGTCGGACTTGTAGGTGGTCTTGAGCAACATATGTATTTTTTTGTCAGAAGTAAGTTGGC CGCA
core_56	TTTTTGGTCTGGGTGAAGTCCCAAGATAC
core_57	TTTTTCTACGTAGCCATGCTCGCGCCATGCTACTTATTTTTT
core_58	CTGCAGGATCTGACGAATGCTTAATTTTTTATACGGGAGGGCCGAGCGCAGAAGT
core_59	TGTGGAGAGAAAGGCATATATGATCTCAGTTAAAACTCA
core_60	TTTTTCCTGGGTCACGGTCACCTGCAGGGAGGAGTTTTTT
core_61	TTTTTGCTGTCCTGCCAAGTCCCGGAATTTTT
core_62	TGACACCTTCCGCCGTGGCAATAGCCAACCAG
core_63	CCTCATTTCCAGCAGCGGGCAAGGGTGCACACCACGCCACGTTGCCTGACAACGG
core_64	CTCGGAGGAGGCCTCGAACTCCTGTCAAACCGGGAAGCG
core_65	TGGTGCCCGTAGGCTGTCCCAGGCGAAGGGTGCTACATCTTGAGT
core_66	TCACCTTGAGGCGCCGTGTCCAACCGTTCGTACTGTTCCACGATGGTGCAATCTTCACGGGC
core_67	GCCCCTGTTATTAGGAAAGGACAGTGGGAGTCGTACACG
core_68	CAGCCGAGGGCGGACCTACCGTTGTTAAAATTCGCG
core_69	CACCACGGCCAGGGTCAAGGCGAGTCTCCT
core_70	AAGGACGAGCAACACAGGCCGAAGGGACGTAGCAGAAGGAGCAACCACCGACAACCGTTGA
core_71	GCCAAGGAGCAGAGCCGGCAGCATTCTCTG
core_72	TGATCTCGCCCTTCAGATACGGGAAGCAATAGCATGATACAAAGGCAGCTTCAG
core_73	TGGCCGCCGTCCTTCATTAAAGCAGCGTATCCACATAGCGTAAAAGG
core_74	CGTCGTAGTGGTCTTCTTCTGCATTACGGGGCGGAGCCCTGCCAAAATTACCGGAGCGCTGCCAAGT GGG
core_75	ACCTCAGGATGTACTCCATGTGTTCAGCCCGACCGC
core_76	TTTTTACTTGTACAGCTCGTTCCAATCCAGGCGGGCCATTTAGTCAGAGGCAGGCGTT
core_77	TTTTTAGGAGCTGACAGGTGGTGGCAATGCCGGAGGGGTGGCGAG
core_78	AAGGAACCCCTAGTGATGGAGTTGGCCACTCCCTCT
core_96	CAGTTTACCGTAAATACTCCACCCATTGACGTCAATGGAAAGTCCCTATTGGCGTTACTATGGGAACAT ACGTCATTATTGACGTCAATGGGCGGGGGTCGTTGGGCGGTCAGCCAGGCGGGCCATTTACCGTAAG TTATGTAACGCGGAACTC
rec1_1	GGCTTACCATCTGGCCTGACTCCAGGCACCTATCTCAGCGAT
rec1_2	TCCATAGTTGCCCCAGTGCTTAAATTTTTCCAGTTGACATTTTGGAAAGTC
rec1_3	ATCAGGGTTATGGGTTCCGAAAACAGGTGTTGAGATCCAGTTC
rec1_4	GTTAATTCAAATAGTGTCTCAATACTCTTCCTTTTTCAA
rec1_5	TATATAGAACCAACTTTAAACCTGTCTTGTAACCT
rec1_6	CAGCATGCCTGGAATAGAACGCTTAATAAAGGAAGTCAGGGCG
rec1_7	CCCCCCACTATTGTTCCTTATCGGGAATTCGC
rec2_1	ATCAATCTAAAGTAAAGTGGATATCCTCCCCTCGGGG
rec2_2	ATCCGGTAAACTCGTGGGACAGGACTTGGAGC
rec2_3	CTCTCCTTGCTACATGCAAGCAGCAGATTACACTAGATGAAGTGG
rec2_4	GGCCAGCAAAATGGCGCTTGTTGCCGGGGTCTGACGCTCAAAATT
rec2_5	GGCCAGGAACTCATGGTTATGGCAGCACTGCCTATTAATTTCTCATAGTCGTTCGGGCCATGTTGTCA
rec2_6	CCTATCAGAAACGCAAGAGTCGGCGCCGTTCGCCCTTCAATGGGG
pro1_pass_1	CAGTTACCGTTATGCCTGGACACCTCTCGACAAGCCCAGTATGGCTGGCAACTTTTTT
pro1_pass_2	AGTGCCTCACGCCTCCCACGGCACCTTAATTGTCAGTGCCCAAGCCACAACTCCTCATAAAGAGACAC GTTTTTT
pro1_pass_3	ACAAGGAGGAGAAAATGGATTGAGGGCGAGCAGGAAGGAATTTTT
pro1_pass_4	TTTTTGGTCCGCTGAAAGCCGGCCGCGTTGACGTGCTCACC
pro1_pass_5	TTTTTCCCGCGCAGAATCTAAGGCACGTTTTT
pro1_pass_6	TTTTTAGAAGGCACAGTCGAGGCAGATCCAGGTGTGATTTCCGGATTTAT
pro1_pass_7	TTTTTGGGGAGGGGCAAACAACAGTTCTATTGAGACGCGGTAAACCAGTTTTGGTGGACCGAGA
pro2_pass_1	ACGCGCAGTGCGCCTTCCTGCCGCCCGCATCATACACTTGAGTTAAGAATACCAGTTAGTTTTT
pro2_pass_2	GACGTCAATTTGTAATCCAGAGGTCCTCGGCGTTGATGTTCCGGTGGATTTTT

pro2_pass_3	CCACCCTTCGGGGATGTCGGCGGGGACAGCTTTCCCACTTGAAGCCCTTTTTT
pro2_pass_4	GACGTTGTTAGATGAATCACGCGCCAAGTAGTCCGTCCTCTTTTT
pro2_pass_5	TTTTTGTGGCGGCTGATTGGACCATGCCGAAGTAGGACCCTCGTA
pro2_pass_6	TTTTTTCCTCGTTGTGGGAGGTGAGGCAGCTGTCACAAATTAGGGGGCGTACTTGCCTGACG
pro2_pass_7	TTTTTGAAGTTCACTCGCCGTCCACGCCGGGTCACCTTCAGCTTGGCTTTTT
pro2_pass_8	TTTTTCGGGGAAGGTGCTTCAGCGCAGCTTCGCCCTC
pro1_x8_1	TGATACAAGGGGAGGGGCAAACAACAGTTCTATTGAGACGCGGTAAACCAGTTTTGGTGGACCGAGA
pro1_x8_2	GGACGTAGGGTCCGCTGAAAGCCGGCCGCGTTGACGTGCTCACC
pro1_x8_3	ATTACTTGCCCGCGCAGAATCTAAGGCACGAGGCATTA
pro1_x8_4	CAGTTACCGTTATGCCTGGACACCTCTCGACAAGCCCAGTATGGCTGGCAACTGGAATTGT
pro1_x8_5	AACACCACAGAAGGCACAGTCGAGGCAGATCCAGGTGTGATTTCCGGATTTAT
pro1_x8_6	AGTGCCTCACGCCTCCCACGGCACCTTAATTGTCAGTGCCCAAGCCACAACTCCTCATAAAGAGACACGTTACAGCTC
pro1_x8_7	ACAAGGAGGAGAAAATGGATTGAGGGCGAGCAGGAAGGAACAGAAGGA
pro2_x8_1	GACGTCAATTTGTAATCCAGAGGTCCTCGGCGTTGATGTTCCGGTGGAAACCCTAA
pro2_x8_2	CTCATTTTCGGGGAAGGTGCTTCAGCGCAGCTTCGCCCTC
pro2_x8_3	GACGTTGTTAGATGAATCACGCGCCAAGTAGTCCGTCCTCAAAGCGAA
pro2_x8_4	AAGGGAAGGAAGTTCACTCGCCGTCCACGCCGGGTCACCTTCAGCTTGGCTTTTT
pro2_x8_5	ACGCGCAGTGCGCCTTCCTGCCGCCCGCATCATACACTTGAGTTAAGAATACCAGTTAGCTCGAAGA
pro2_x8_6	AGGCAGATTCCTCGTTGTGGGAGGTGAGGCAGCTGTCACAAATTAGGGGGCGTACTTGCCTGACG
pro2_x8_7	TAAATCGGGTGGCGGCTGATTGGACCATGCCGAAGTAGGACCCTCGTA
pro2_x8_8	CCACCCTTCGGGGATGTCGGCGGGGACAGCTTTCCCACTTGAAGCCCTATTAGGAA
pro1_x5_1	TACAAGGGGAGGGGCAAACAACAGTTCTATTGAGACGCGGTAAACCAGTTTTGGTGGACCGAGA
pro1_x5_2	CGTAGGGTCCGCTGAAAGCCGGCCGCGTTGACGTGCTCACC
pro1_x5_3	ACTTGCCCGCGCAGAATCTAAGGCACGAGGCA
pro1_x5_4	CAGTTACCGTTATGCCTGGACACCTCTCGACAAGCCCAGTATGGCTGGCAACTGGAAT
pro1_x5_5	ACCACAGAAGGCACAGTCGAGGCAGATCCAGGTGTGATTTCCGGATTTAT
pro1_x5_6	AGTGCCTCACGCCTCCCACGGCACCTTAATTGTCAGTGCCCAAGCCACAACTCCTCATAAAGAGACACGTTACAG
pro1_x5_7	ACAAGGAGGAGAAAATGGATTGAGGGCGAGCAGGAAGGAACAGAA
pro2_x5_1	GACGTCAATTTGTAATCCAGAGGTCCTCGGCGTTGATGTTCCGGTGGAAACCC
pro2_x5_2	ATTTTCGGGGAAGGTGCTTCAGCGCAGCTTCGCCCTC
pro2_x5_3	GACGTTGTTAGATGAATCACGCGCCAAGTAGTCCGTCCTCAAAGC
pro2_x5_4	GGAAGGAAGTTCACTCGCCGTCCACGCCGGGTCACCTTCAGCTTGGCTTTTT
pro2_x5_5	ACGCGCAGTGCGCCTTCCTGCCGCCCGCATCATACACTTGAGTTAAGAATACCAGTTAGCTCGA
pro2_x5_6	CAGATTCCTCGTTGTGGGAGGTGAGGCAGCTGTCACAAATTAGGGGGCGTACTTGCCTGACG
pro2_x5_7	ATCGGGTGGCGGCTGATTGGACCATGCCGAAGTAGGACCCTCGTA
pro2_x5_8	CCACCCTTCGGGGATGTCGGCGGGGACAGCTTTCCCACTTGAAGCCCTATTAG
