# Supplementary material for: Gene-encoding DNA origami for mammalian cell expression
Source: Nat Commun. 2023 Feb 23;14:1017. doi: 10.1038/s41467-023-36601-1 (PMC9950468; doi:10.1038/s41467-023-36601-1)
Supplement: Supplementary file 2 — Reporting Summary [file 41467_2023_36601_MOESM2_ESM.pdf]

nature portfolio

Corresponding author(s): Hendrik Dietz
Last updated by author(s): 03.02.2023

Reporting Summary

Nature Portfolio wishes to improve the reproducibility of the work that we publish. This form provides structure for consistency and transparency in reporting. For further information on Nature Portfolio policies, see our Editorial Policies and the Editorial Policy Checklist.

Statistics

For all statistical analyses, confirm that the following items are present in the figure legend, table legend, main text, or Methods section.

n/a	Confirmed	
☐	☒	The exact sample size (*n*) for each experimental group/condition, given as a discrete number and unit of measurement
☐	☒	A statement on whether measurements were taken from distinct samples or whether the same sample was measured repeatedly
☐	☒	The statistical test(s) used AND whether they are one- or two-sided<br>*Only common tests should be described solely by name; describe more complex techniques in the Methods section.*
☒	☐	A description of all covariates tested
☐	☒	A description of any assumptions or corrections, such as tests of normality and adjustment for multiple comparisons
☐	☒	A full description of the statistical parameters including central tendency (e.g. means) or other basic estimates (e.g. regression coefficient) AND variation (e.g. standard deviation) or associated estimates of uncertainty (e.g. confidence intervals)
☐	☒	For null hypothesis testing, the test statistic (e.g. *F*, *t*, *r*) with confidence intervals, effect sizes, degrees of freedom and *P* value noted<br>*Give P values as exact values whenever suitable.*
☒	☐	For Bayesian analysis, information on the choice of priors and Markov chain Monte Carlo settings
☒	☐	For hierarchical and complex designs, identification of the appropriate level for tests and full reporting of outcomes
☒	☐	Estimates of effect sizes (e.g. Cohen's *d*, Pearson's *r*), indicating how they were calculated

Our web collection on statistics for biologists contains articles on many of the points above.

Software and code

Policy information about availability of computer code

Data collection	SerialEM 3.5.6, Attune Nxt Software v3.1, Invitrogen EVOS M7000 Imaging System v2.0.2094.0
Data analysis	Etomo 4.9.0 (Imod), Graphpad Prism v9.4.1, FlowJo v10.7.1/ v10.8.1, ImageJ v2.9.0/1.53t, caDNAno sq v0.1, caDNAno v2, Adobe Photoshop v24.1.0

For manuscripts utilizing custom algorithms or software that are central to the research but not yet described in published literature, software must be made available to editors and reviewers. We strongly encourage code deposition in a community repository (e.g. GitHub). See the Nature Portfolio guidelines for submitting code & software for further information.

Data

Policy information about availability of data

All manuscripts must include a data availability statement. This statement should provide the following information, where applicable:
- Accession codes, unique identifiers, or web links for publicly available datasets
- A description of any restrictions on data availability
- For clinical datasets or third party data, please ensure that the statement adheres to our policy

The data supporting the findings of this study are available within the paper and its supplementary information files, and are available from the corresponding author upon reasonable request.

Human research participants

Policy information about studies involving human research participants and Sex and Gender in Research.

Reporting on sex and gender	NA
Population characteristics	NA
Recruitment	NA
Ethics oversight	NA

Note that full information on the approval of the study protocol must also be provided in the manuscript.

Field-specific reporting

Please select the one below that is the best fit for your research. If you are not sure, read the appropriate sections before making your selection.

☒ Life sciences ☐ Behavioural & social sciences ☐ Ecological, evolutionary & environmental sciences

For a reference copy of the document with all sections, see nature.com/documents/nr-reporting-summary-flat.pdf

Life sciences study design

All studies must disclose on these points even when the disclosure is negative.

Sample size	A sample size of 3 biological replicates was used to detect a significant difference (p < 0.05) between groups with a signal to noise ratio of 3.0 with 80% power.
Data exclusions	No data was excluded.
Replication	Experiments were performed multiple times to confirm the observations, the number of biological replicates is provided in the caption of the relevant figures. All attempts at replication were successful.
Randomization	Cells used for test experiments were randomly assigned to experimental groups in the process of sample preparation.
Blinding	Blinding was not used for cell experiments due to the use of automated collection and analysis systems, where each entire set of cytometry data was acquired and analyzed using identical parameters. Blinding was not performed for EM-micrographs and fluorescent microscopy experiments as they were not used for direct measurements or statistical analysis to justify blinding, and instead were used as complementary qualitative methods to bulk quantitative methods such as AGE gels and flow cytometry.

Reporting for specific materials, systems and methods

We require information from authors about some types of materials, experimental systems and methods used in many studies. Here, indicate whether each material, system or method listed is relevant to your study. If you are not sure if a list item applies to your research, read the appropriate section before selecting a response.

Materials & experimental systems

n/a	Involved in the study	
☒	☐	Antibodies
☐	☒	Eukaryotic cell lines
☒	☐	Palaeontology and archaeology
☒	☐	Animals and other organisms
☒	☐	Clinical data
☒	☐	Dual use research of concern

Methods

n/a	Involved in the study	
☒	☐	ChIP-seq
☐	☒	Flow cytometry
☒	☐	MRI-based neuroimaging

Eukaryotic cell lines

Policy information about cell lines and Sex and Gender in Research

Cell line source(s)	HEK293T cells were purchased from Leibniz Institute, DSMZ-German Collection of Microorganisms and Cell Cultures GmbH.
Authentication	Cell lines were kept at low passage and were not further authenticated.
Mycoplasma contamination	Cell lines tested negative for mycoplasma contamination.
Commonly misidentified lines (See ICLAC register)	No commonly misidentified cell lines were used.

Flow Cytometry

Plots

Confirm that:

- [x] The axis labels state the marker and fluorochrome used (e.g. CD4-FITC).
- [x] The axis scales are clearly visible. Include numbers along axes only for bottom left plot of group (a 'group' is an analysis of identical markers).
- [x] All plots are contour plots with outliers or pseudocolor plots.
- [x] A numerical value for number of cells or percentage (with statistics) is provided.

Methodology

Sample preparation	HEK293T cells were washed with PBS and trypsinized. Cells were then collected and fixed with 2% formaldehyde before being analyzed on the flow cytometer.
Instrument	Attune Nxt Flow Cytometer
Software	Attune Nxt Flow Cytometer Software
Cell population abundance	20,000 single cell events were analyzed for each condition.
Gating strategy	Cells were gated first by FSC/SSC, and then single cells were gated on SSC-A/SSC-H. Untreated cells were used as a negative control, and plasmids encoding for the fluorescent protein corresponding to each experiment were used as positive controls.

- [x] Tick this box to confirm that a figure exemplifying the gating strategy is provided in the Supplementary Information.
