# Supplementary material for: Gene-encoding DNA origami for mammalian cell expression
Source: Nat Commun. 2023 Feb 23;14:1017. doi: 10.1038/s41467-023-36601-1 (PMC9950468; doi:10.1038/s41467-023-36601-1)

Source data – Fig S1

a

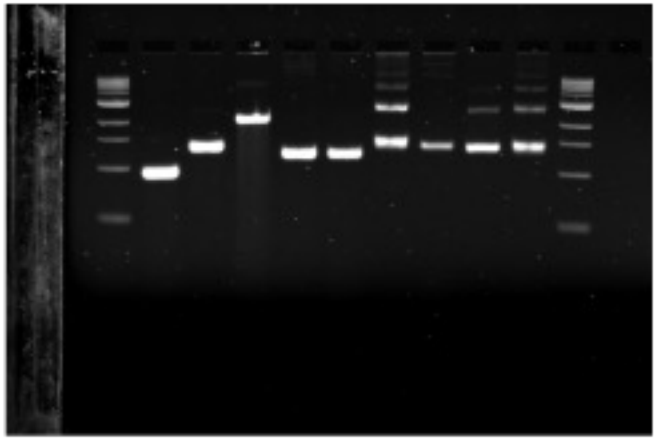

b

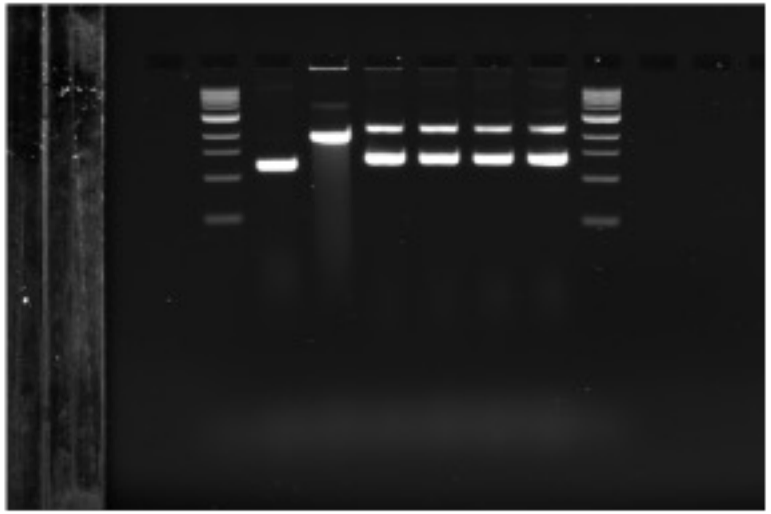

Source data – Fig S2

a

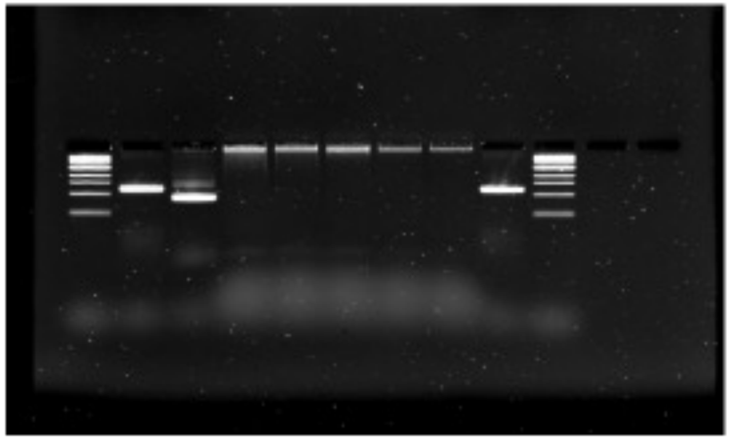

b

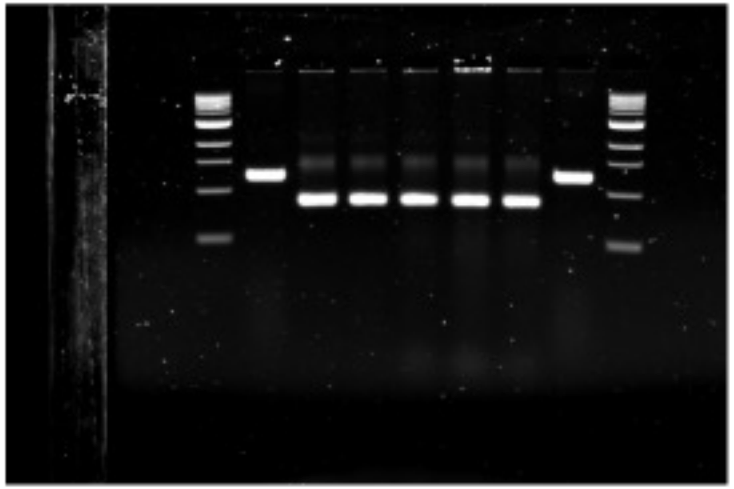

Source data – Fig S3

a

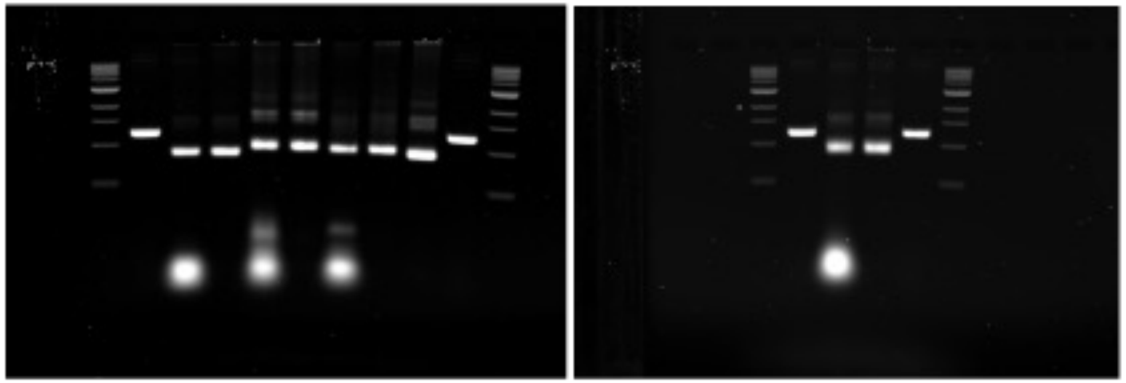

b

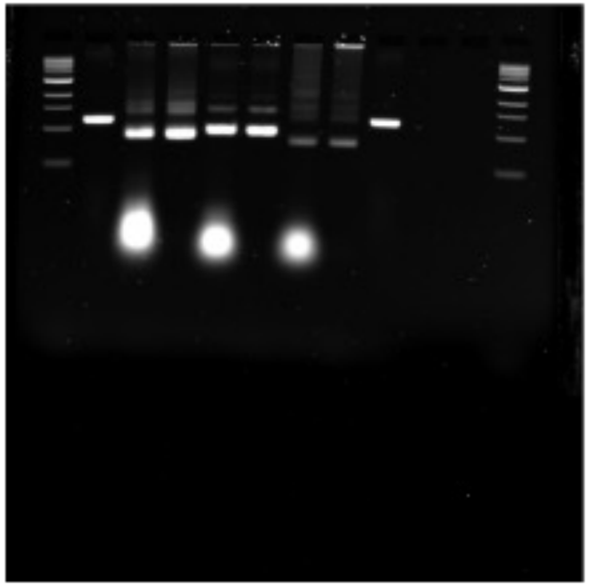

Source data – Fig S6

c

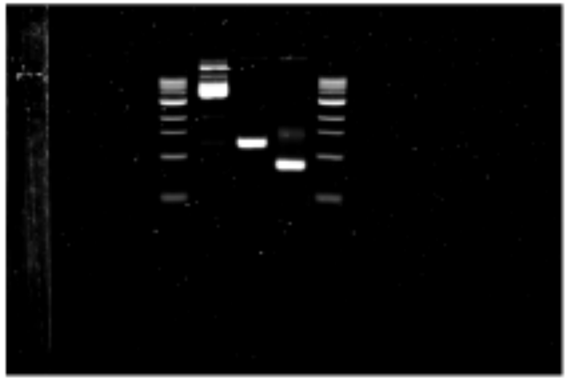

Source data – Fig S7

a

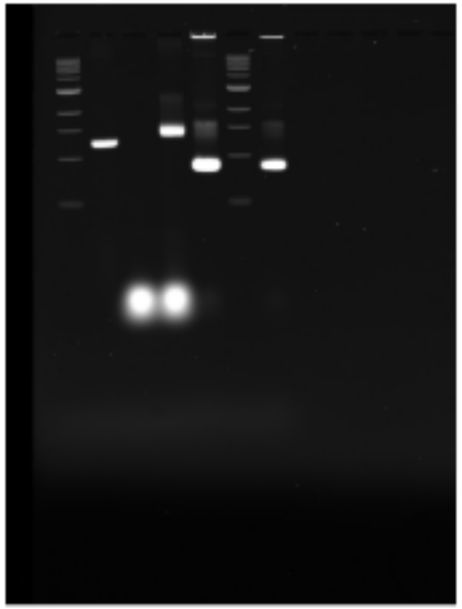

Source data – Fig S9

a

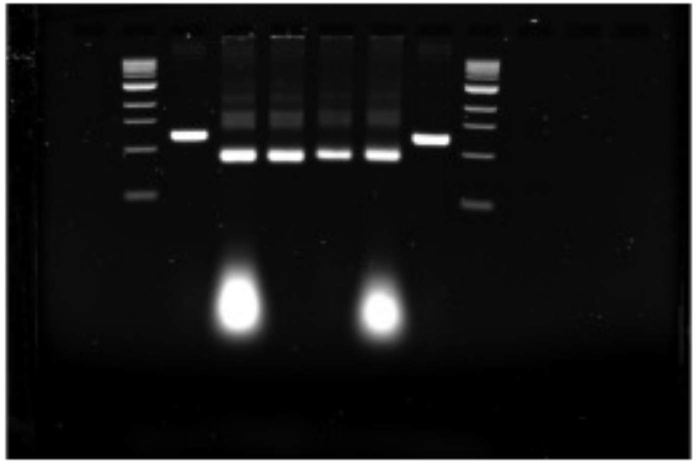

b

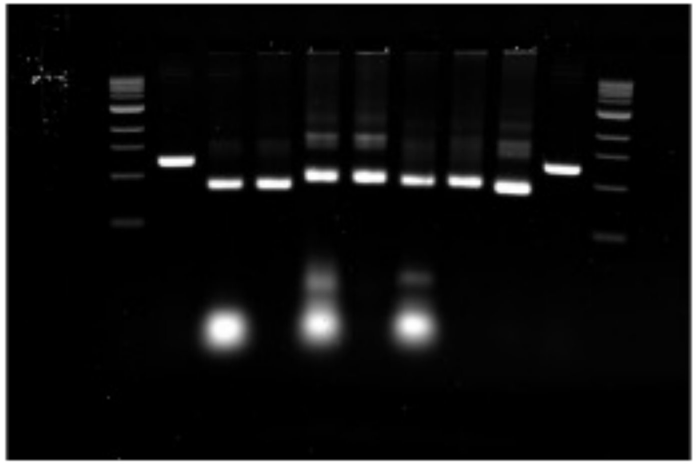

Source data – Fig S10

a

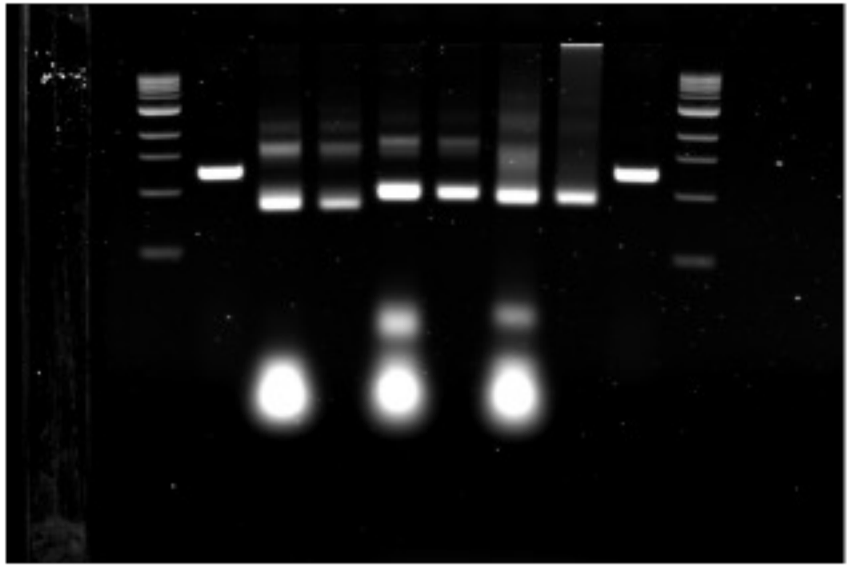

Source data – Fig S13

a

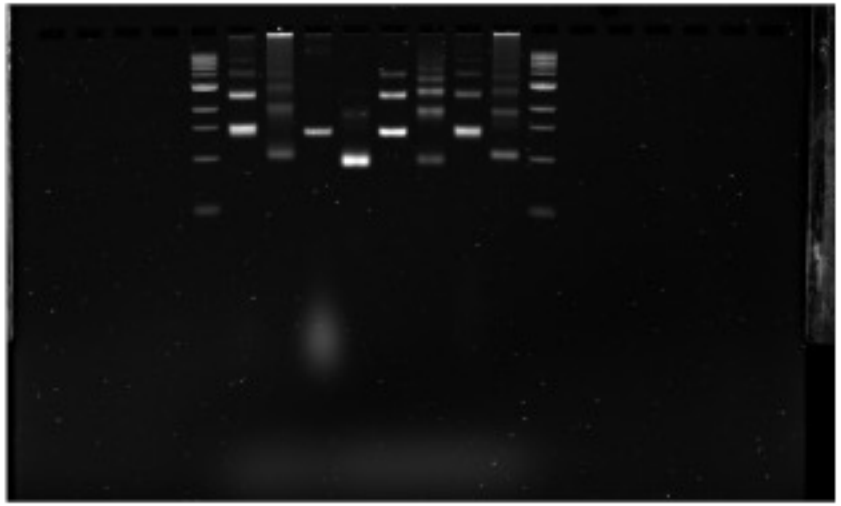

Source data – Fig S15

a

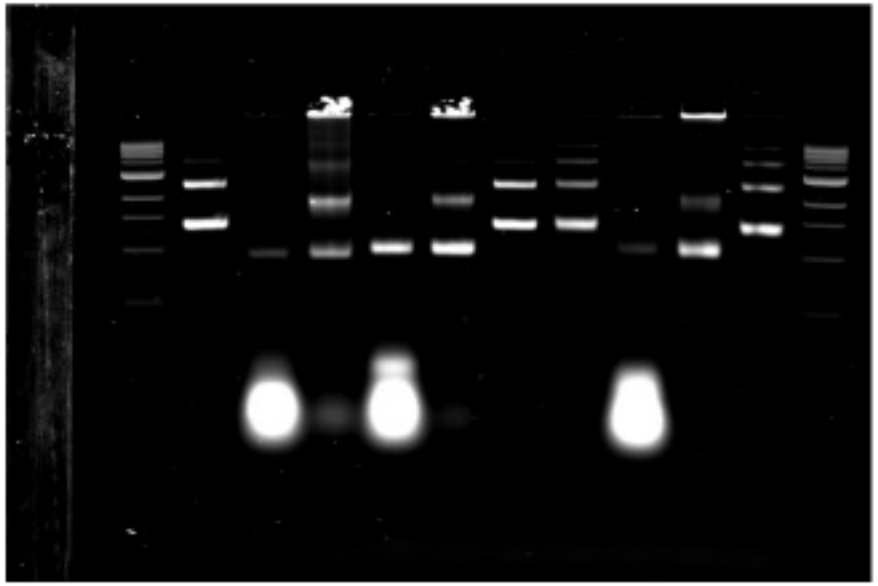

Source data – Fig S18

a

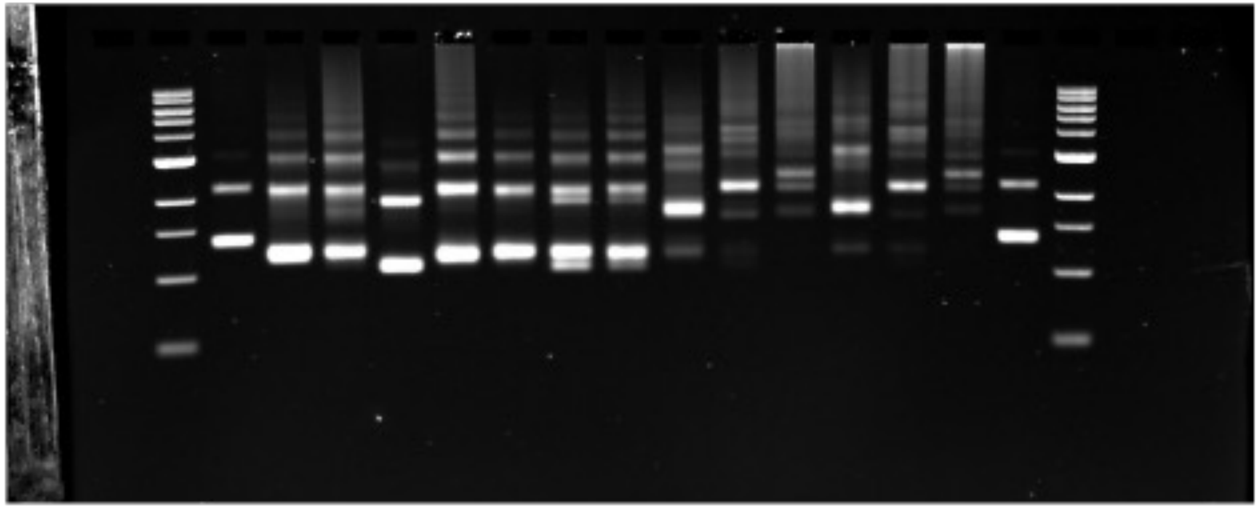

Supplement: Supplementary file 3 — Source Data [file 41467_2023_36601_MOESM3_ESM.zip › Source Data/Source data 2.pdf]
